# Design, synthesis, and high-throughput *in vitro* anti-cancer evaluation of novel 4-aminopyrazolo[3,4-*d*]pyrimidine derivatives: potential anti-cancer candidates against UO-31 renal cancer cells†

**DOI:** 10.1039/d4ra05136j

**Published:** 2024-09-27

**Authors:** Amitananda Dash, Guruswamy Vaddamanu, Mohammed B. Hawsawi, Mustafa S. Alluhaibi, Pavana Kumari Gurijala, Naveen Mulakayala

**Affiliations:** a Sri Sathya Sai Institute of Higher Learning Anantapur – 515 001 Andhra Pradesh India; b SVAK Life Sciences ALEAP Industrial Area, Pragathi Nagar Hyderabad – 500090 India naveen071280@gmail.com; c Department of Chemistry, Faculty of Science, Umm Al-Qura University Makkah 21955 Saudi Arabia

## Abstract

A novel series of 20 compounds containing 4-aminopyrazolo[3,4-*d*]pyrimidine core were synthesized, characterized and their chemical structures confirmed using spectroscopic techniques such as ^1^H NMR, ^13^C NMR, IR, and HRMS. The compound's growth inhibitory activities were evaluated against 60 human tumor cell lines from nine panels: leukemia, non-small cell lung cancer (NSCLC), colon, central nervous system (CNS), melanoma, ovarian, renal, prostate, and breast cancer. Among all the compounds, 11, 12c, 12d, 12f, and 12j are active against different cancer cell lines. Between all the cell lines, compounds 12c, 12d, 12f, 12j, and 11 showed good inhibitory activity against renal cancer cell lines. From the five-dose study, based on IC_50_ values, the order of activity of compounds against renal cancer cell lines was found to be 12c > 12f > 12c > 12j > 11 with 12c being the most potent, was better than sunitinib and sorafenib. Having been recognized as initial hits, these substances need additional pharmacological investigation.

## Abstract 2

Twenty novel 4-aminopyrazolo[3,4-*d*]pyrimidine core compounds were synthesized, characterized and identified as novel anti-cancer compounds. These compounds showed excellent activity against UO-31 Renal Cancer Cells.

## Graphical abstract

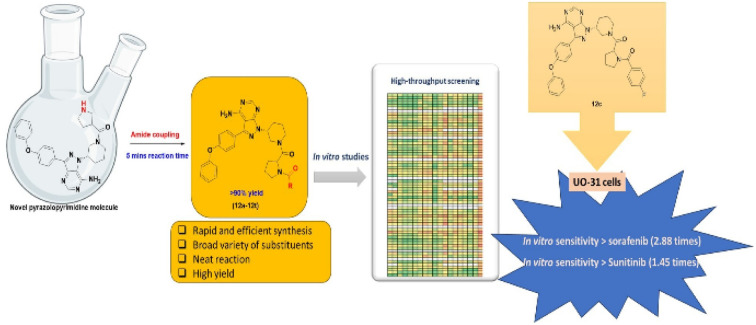

## Introduction

**Fig. 1:**
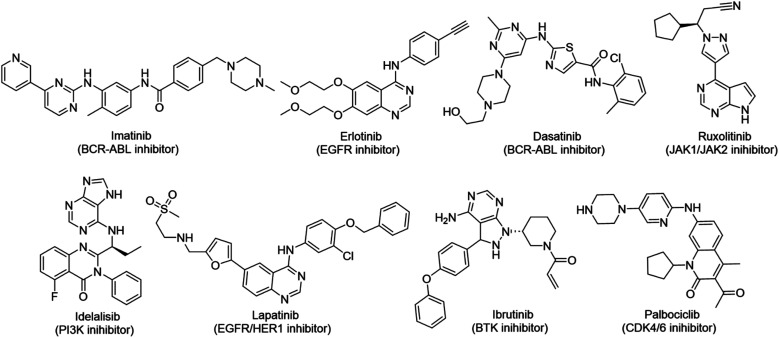
Pyrimidines in small-molecule inhibitors used in selective killing of tumorigenic cells.

Cancer continues to be a significant global challenge. It was responsible for an estimated 9.7 million deaths, with approximately 20 million new cases worldwide in the year 2022.^1^ The World Health Organization (WHO) has predicted a 77% increase in cancer burden globally by the year 2050.^2^ Pyrimidines are a fundamental class of hetero-aromatic compounds that play a crucial role in forming essential components of DNA and RNA, such as cytosine, thymine, and uracil. Their simple yet functionalized structures make them vital scaffolds in synthesizing many drugs and biologically active compounds, including anti-cancer drugs.^3,4^ Derivatives of pyrimidine moiety were known to mimic the natural pyrimidines and interfere with many important biological targets, making them a vital scaffold in the synthesis of novel anti-cancer agents.^5^ Several protein kinase inhibitors (PKIs) are discovered with amine-substituted pyrimidines as their bioactive core (Fig. 1).^6–8^

A diversity-oriented synthesis (DOS) strategy^9–11^ was employed to design a library of novel compounds with potential anti-cancer activity. Since DOS approach in medicinal chemistry aims to create structurally diverse molecules, it enhances the probability of discovering compounds with unique biological activities. Unlike traditional methods, DOS does not restrict itself to specific protein targets, thereby opening up new avenues for uncovering previously unidentified critical pathways involved in tumorigenesis. Recently, we have utilized this technique for the design of a novel quinazoline series where compounds had exhibited interesting properties with excellent anticancer activity against breast cancer cell lines while inflicting minimum effect on non-cancer cells.^12^ In our current study, the 4-aminopyrazolo[3,4-*d*]pyrimidine scaffold was integrated with several structurally diverse biologically active fragments, starting from simple aromatics, halogen-substituted aromatics, heterocycles, polycyclic fragments, *etc.* The choice to combine multiple drug scaffolds into single molecules was driven by the hypothesis that this approach could (1) address drug resistance by bypassing common resistance pathways, (2) reduce the need for combination therapy, and (3) enhance efficacy by simultaneously targeting multiple pathways. Moreover, the compounds were subjected to high throughput *in vitro* screening against 60 human tumour cell lines consisting of the most extensive cancer pharmacology database.^13^

**Fig. 2:**
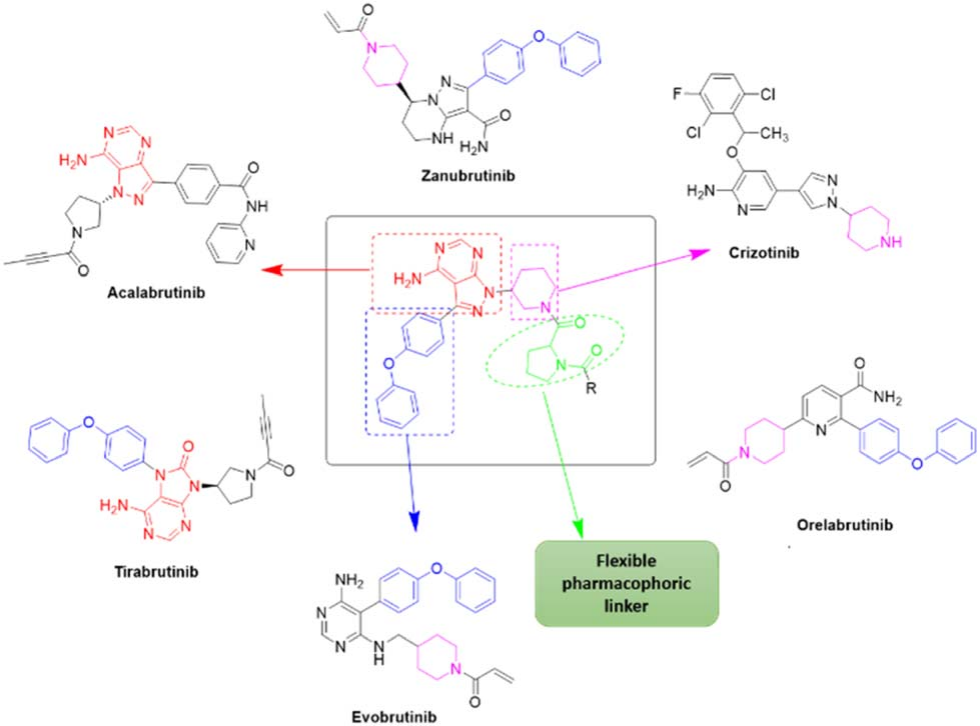
Design model for the novel molecules.

Due to our continuous efforts in identifying novel biologically active compounds,^14^ we have synthesized novel derivatives containing 4-aminopyrazolo[3,4-*d*]pyrimidine (4-APP) scaffold, which is a privileged scaffold found in many biologically active compounds.^15–19^ All the synthesized compounds under the present study were rigorously characterized using spectroscopic techniques such as ^1^H NMR, ^13^C NMR, IR and HRMS. Given our interest in exploring small molecules for their bioactivities,^20–23^ we have considered the molecular skeleton of several small molecule inhibitors (SMIs) to design a new series of small molecules with a 4-APP core (Fig. 2). An evaluation of their growth inhibitory activities was performed against 60 human tumor cell lines belonging to nine different panels including leukaemia, non-small cell lung cancer (NSCLC), colon, central nervous system (CNS), melanoma, ovarian, renal, prostate, and breast cancer cell lines. Additionally, the structure–activity relationships were studied and the activities of the most active compounds were compared with that of the market drugs to understand how well the new compounds perform in relation to existing small molecule drugs.

## Results and discussion

### Chemistry

**Scheme 1:**
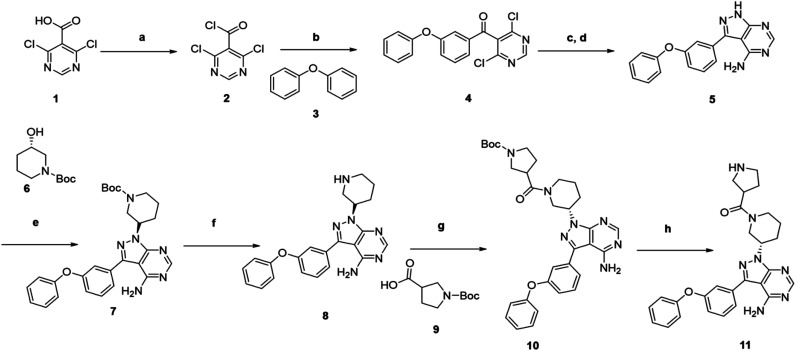
Synthesis of novel 4-aminopyrazolo[3,4-*d*]pyrimidine amine derivative (11) that was used as starting material for the synthesis of the novel library.

**Scheme 2:**
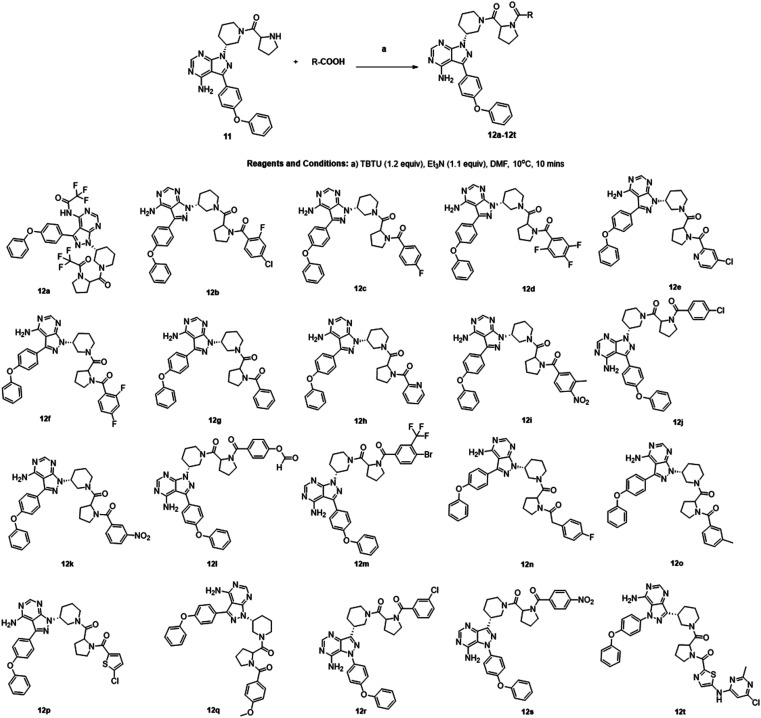
Synthesis of novel pyrazolopyrimidine derivatives (12a–12t).

We followed the literature procedure to synthesize up to compound 8.^24^ Compound 8 was then reacted with Boc-proline to get compound 10, which was deprotected using 1 M HCl to get the target core 11 (Scheme 1). Compound 11 was used as a starting material for synthesizing new derivatives. The synthetic sequences leading to the formation of novel pyrazolopyrimidine derivatives are illustrated in Scheme 2. The synthetic procedure of compound 8 was started from 4,6-dichloropyrimidine-5-carboxylic acid 1. Compound 1 was converted to its corresponding acid chloride, followed by Friedel–Crafts acylation on diphenyl ether, which yielded compound 4. Compound 4 on reaction with ammonia and hydrazine hydrate to produce pyrazolopyrimidine ring 5. The pyrazolopyrimidine 5 was reacted with BOC-protected piperidin-3-ol 6 to give compound 7, which was deprotected with HCl gave compound 8. Compound 8 upon coupling with BOC-protected proline followed by deprotection with 1 M HCl gave a novel intermediate 11. Derivatives were prepared from compound 11 using different acids to get required compounds. To prepare these derivatives, amide coupling was employed using appropriate reagent to carry out reactions between compound 11 with various carboxylic acids including aliphatic, aromatic, heteroaryl, and also polycyclic carboxylic acids.

**Scheme 3:**
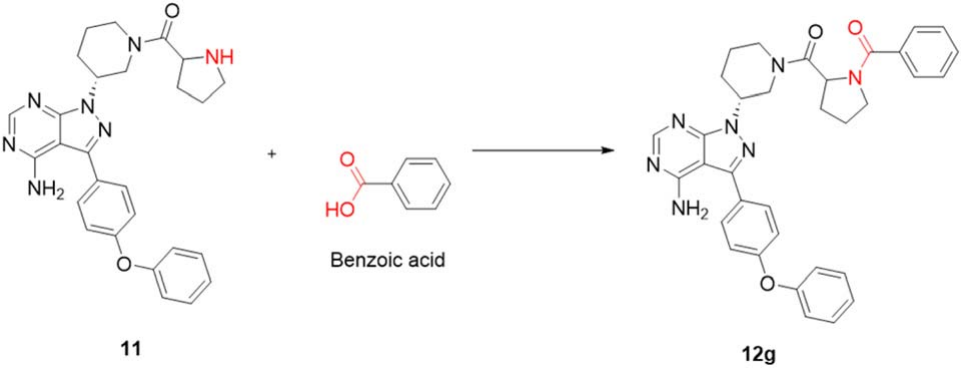
Model reaction for the synthesis of 12g.

The choice of reagent^25–27^ for the amide coupling in the final step was optimized using different reaction conditions summarized in Table 1. The optimization for the choice of amide coupling reagent was performed by taking benzoic acid (g) and compound 11 as starting material. Several reagents such as DCC/HOBT, EDC/HOBT, and triphenylphosphine in the presence of iodine were considered. But the reaction time was either too long or the yield was unsatisfactory or tedious work up was involved during the usage of the above reagents. To optimize the reaction further, we have tested 2-(1*H*-benzotriazole-1-yl)-1,1,3,3-tetramethylammonium tetrafluoroborate^28^ (TBTU) and *N*,*N*,*N*′,*N*′-tetramethyl-*O*-(1*H*-benzotriazol-1-yl)uronium hexafluorophosphate (HBTU) for coupling between benzoic acid (g) and compound 11. TBTU, in the presence of triethylamine, was yielded 96% of the required product when compared with TBTU. So HBTU was found to be the best coupling reagent, which is yielding more than 80% of the required product in each reaction, and was chosen for the amide coupling reaction. The reaction was deemed successful with a wide variety of structurally diverse biologically active fragments, which proved the versatility of the reactions that are possible with the novel intermediate synthesized; depending on the structure of the carboxylic acids, the reaction time varied from five minutes to three hours (Scheme 3).

**Table 1:** Optimization of reaction conditions for the synthesis of 1-(2-((*R*)-3-(4-amino-3-(4-phenoxyphenyl)-1*H*-pyrazolo[3,4-*d*]pyrimidin-1-yl)piperidine-1-carbonyl)pyrrolidin-1-yl)-2-(4-fluorophenyl)ethan-1-one (12g)

Entry	Base	Reagent	Solvent^a^	Temperature (°C)	Reaction time	Yield^b^ (%)
1	—	SOCl_2_	DCM	0 to RT	18 h	48
2	Et_3_N	SOCl_2_	DCM	0 to RT	1 h	67
3	Et_3_N	PPh_3_, I_2_	DCM	0 to RT	90 min	81
4	—	EDC/HOBT	DMF	70	18 h	66
5	Et_3_N	EDC/HOBT	DMF	70	10 h	89
6	—	DCC/HOBT	ACN	20	12 h	69
7	Et_3_N	DCC/HOBT	ACN	50	12 h	83
8	Et_3_N	HBTU	DMF	10	10 min	96
9	NNM	TBTU	DMF	10	5 min	85
10	Et_3_N	TBTU	DMF	10	5 min	95

a Choice of solvent is based on solubility and/or literature survey.

b Yield was calculated after compound isolation and purification.

All the new molecules synthesized under this work were characterized using spectroscopic techniques, such as electron spray ionisation mass spectroscopy (HRMS), ^1^H NMR, and ^13^C NMR. The details are available in the ESI.†

### Biological evaluation

The cells in the NCI-60 panel were extensively profiled at the DNA, RNA, protein, chromosomal, and functional level,^29–31^ providing a diverse genotypic and karyotypic range of cell lines for screening new compounds. The single-dose data of the compounds were represented as a mean growth inhibition percentage (%GI) in Table 2. This preliminary screening allowed for the selection of compounds with exceptional anti-cancer properties for further investigation. Subsequently, compounds exhibiting outstanding anti-proliferative activity against many cell lines were chosen for the next phase. In this phase, the selected compounds were subjected to a dose-dependent study to determine the IC_50_ values across various cancer cell lines at different concentrations (100 μM, 10 μM, 1 μM, 0.1 μM, and 0.01 μM).

**Table 2:** Single-dose NCI-60 screening results exhibiting the %GI in the various tumour cell lines in response to treatment with compounds (12a–12t) at 10 μM concentration^a^

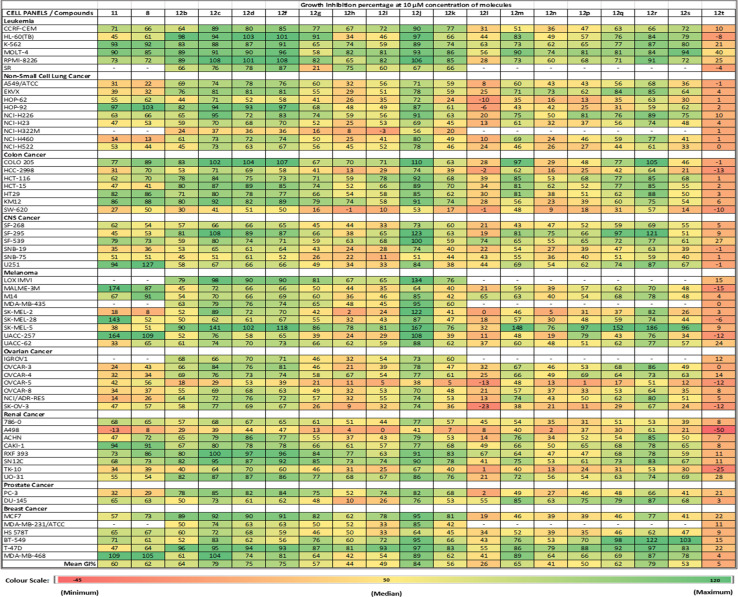

a Values lower than zero (denoted by red) correspond to no effect on the specific cell line at 10 μM. Values greater than 100 (denoted by green) correspond to lethality on the specific cell line at 10 μM. ‘—’ denotes that the compound(s) was not tested against the particular cell line(s).

**Fig. 3:**
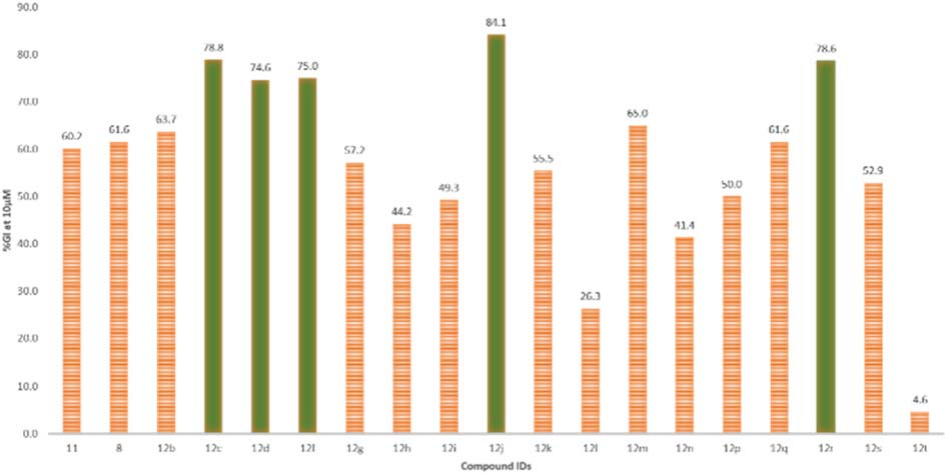
Depiction of average %GI exhibited by all compounds in the study, highlighting the compounds with >75% growth inhibition (in green), at 10 μM concentration.

The single-dose study provided an overview of the anti-cancer activity of all the molecules under this study. Among the tested molecules, five compounds (12c, 12d, 12f, 12j, and 12r) were the most active and exhibited exceptional GI activity across all cell lines (Fig. 3). Compounds 12c, 12d, 12f, 12i, 12j, 12m, 12q, and 12r exhibited the best activity in the human melanoma cell line, *i.e.*, SK-MEL-5 (%GI > 100), whereas compounds 12d and 12f were the most active in HL-60 (TB) (GI% > 101) in the leukemia panel. Compound 11 showed selective growth inhibition in cell lines such as K-562 (%GI = 93) from leukemia, MALME-3M (%GI = 174) from melanoma, and MDA-MB-468 (%GI = 109) from the breast cancer panel. The compounds 12b and 12r displayed significant activity in cell lines including HL-60(TB) (%GI = 98 for 12b), MOLT-4 (%GI = 94 for 12r), and K-562 (%GI = 92 for 8) in the leukaemia panel, HOP-92 (%GI = 82 for 12b) in NSCLC panel, COLO205 (%GI = 105 for 12r) in the colon cancer panel, SK-MEL-5 (%GI = 186 for 12r) and UACC-257 (%GI = 108 for compound 8) in melanoma panel, and BT-549 (%GI = 122 for 12r) from breast cancer panel. Other compounds that exhibited selective activity were 12q and 12s (%GI = 155 and 96, respectively, in SK-MEL-5) in melanoma, 12g (%GI = 91 in HL-60(TB)) in leukemia, 12i (%GI = 93 in T-47D) in breast cancer panel. The compounds with %GI values which were beyond 100 correspond to lethality in the mentioned cell lines were considered as more active compounds against the corresponding cell lines. The average growth inhibition percentage was calculated for each of the compounds and the screening results from the single-dose study were summarized in Table 2.

From the five-dose study, the mean IC_50_ values (Table 3) revealed that 12j was the most active compound, followed by 12c as they exhibited growth inhibitory activity against most of the cell lines in the NCI60 panel. The cell lines in the leukemia cancer panel were found to be sensitive to all five test drugs under the study. Upon considering the individual activities of the molecules, the UO-31 cell line from the renal cancer panel was the most sensitive (12c, IC_50_ = 0.87 μM), followed by HL-60 (TB) (12f, IC_50_ = 1.41 μM) and MOLT-4 (IC_50_ = 1.51 μM) in the leukemia panel. Compound 12d was most active against MOLT-4 (IC_50_ = 2.0 μM) and HL-60(TB) (IC_50_ = 2.63 μM) from leuke mia panel. Compounds 12c and 12j were especially active in MOLT-4 (IC_50_ = 1.58 μM and 1.82, respectively). In the melanoma panel, compound 12j was the most effective in inhibiting tumor growth. Certain cell lines exhibited selective sensitivity. For example, cell lines such as MOLT-4 in the leukemia panel and SK-MEL-5 in the melanoma panel were susceptible to all five drug agents in the study, but cell lines such as M14 in the melanoma cancer panel were affected by selective compounds (11, IC_50_ = 2.40 μM; 12f, IC_50_ = 15.5 μM) only.

**Table 3:** Reduction in 50% of the net protein increase (IC_50_ in μM) as measured by SRB staining experiment by compounds across the tumour cell panels^a^

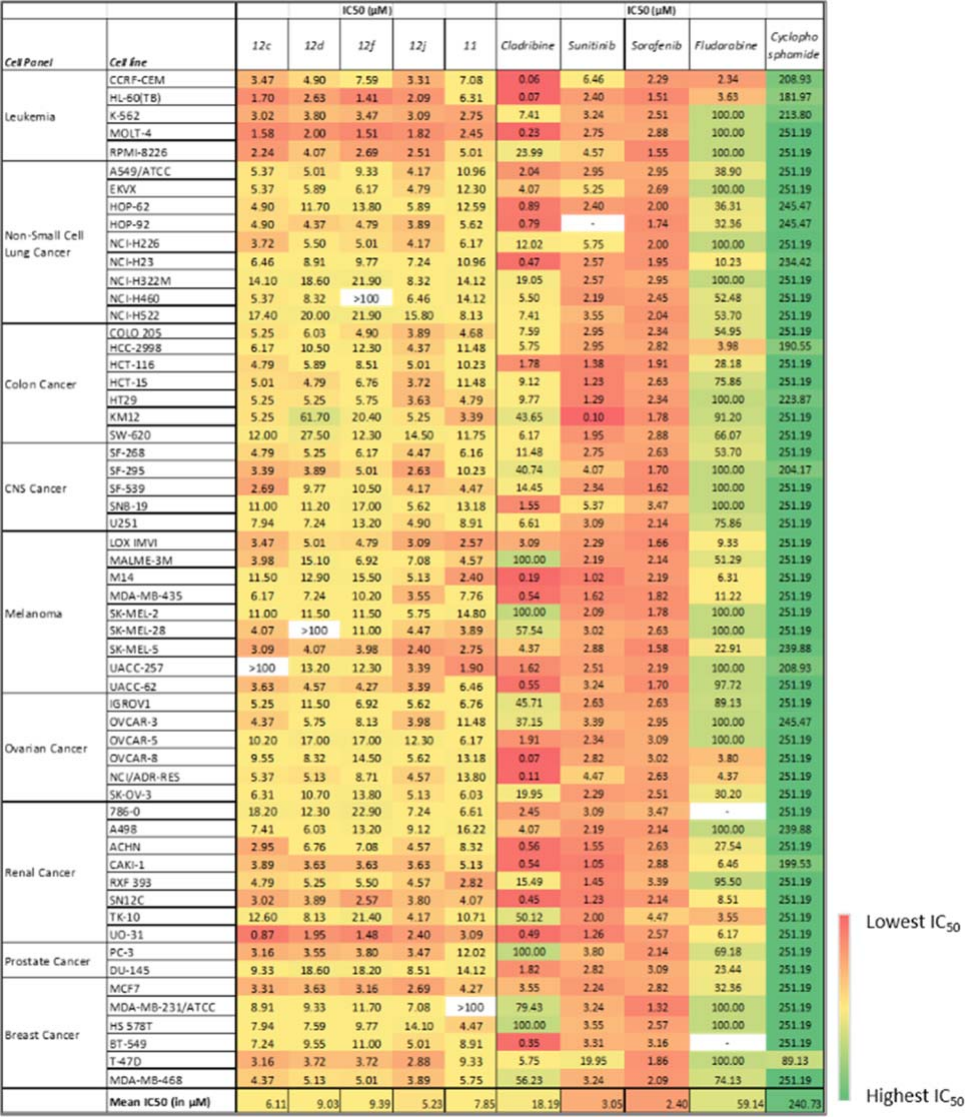

a The IC50 values equal to “>100” mean that the concentration required to achieve 50% activity has exceeded the highest tested concentration range, *i.e.*, 100 μM. Hence, the values for such cases are reported as either equal to or greater than 100 μM.

**Fig. 4:**
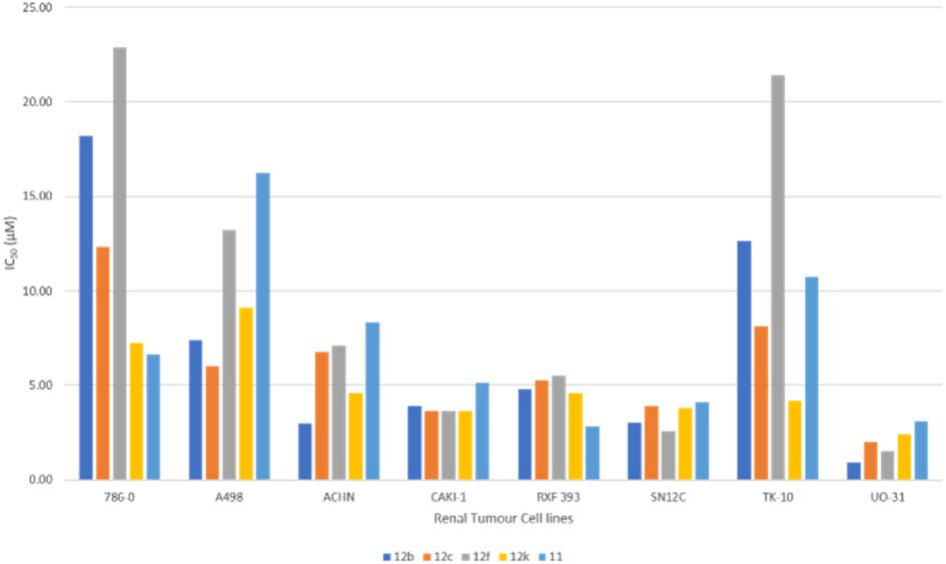
Cytotoxic activity trend in renal cancer sub-panel depicting the IC_50_ value for compounds in the dose-dependent study.

**Fig. 5:**
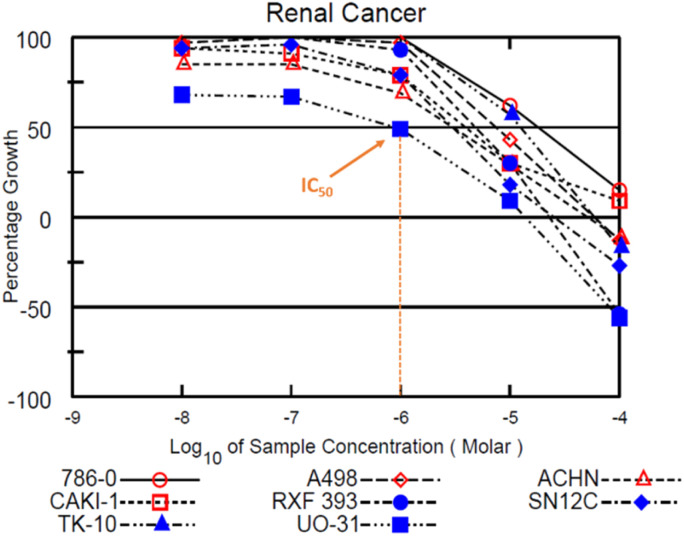
Dose–response curves in the renal cancer sub-panel depicting the IC_50_ value for compound 12b in UO-31 tumour cell line.

Fig. 4 highlights the compound's cytotoxicity trend. Comparing their cytotoxic activity, based on IC_50_ values, against established small-molecule drugs for renal cancer treatment reveals compelling results. Remarkably, compound 12c demonstrated 2.88 times greater activity than sorafenib and 1.45 times greater activity than sunitinib. Detailed data is presented in Table 4, while Fig. 5 showcases a striking dose–response curve for compound 12c in the highly sensitive UO-31 tumor cell line.

**Table 4:** Anti-cancer activity comparison of the drug agents in terms of IC_50_ with market drugs that are currently in use for the treatment of renal cancer

Compound ID (NSC ID)	Renal cancer sub-panel (IC50 in μM)							
	786-0	A498	ACHN	CAKI-1	RXF 393	SN12C	TK-10	UO-31
12b (844710)	18.2	7.41	2.95	3.89	4.79	3.02	12.6	**0.87**
12c (844703)	12.3	6.03	6.76	3.63	5.25	3.89	8.13	1.95
12f (844704)	22.9	13.2	7.08	3.63	5.50	2.57	21.4	1.48
12j (844708)	7.24	9.12	4.57	3.63	4.57	3.8	4.17	2.40
11 (845676)	6.61	16.22	8.32	5.13	2.82	4.07	10.7	3.09
Sunitinib^a^ (750690)	3.09	2.18	1.55	1.05	1.41	1.23	1.99	**1.26**
Sorafenib^a^ (747971)	3.47	2.13	2.63	2.88	3.39	2.51	4.47	**2.57**

a The IC_50_ values for the standard drugs were obtained from the COMPARE database of DTP-NCI using their NSC IDs.

In summary, these findings underscore the potential of the candidate molecules as promising hit compounds, meriting comprehensive investigation to unravel their pharmacokinetics and pharmacodynamics, alongside evaluating their efficacy in animal model studies.

### Structure–activity relationships

**Fig. 6:**
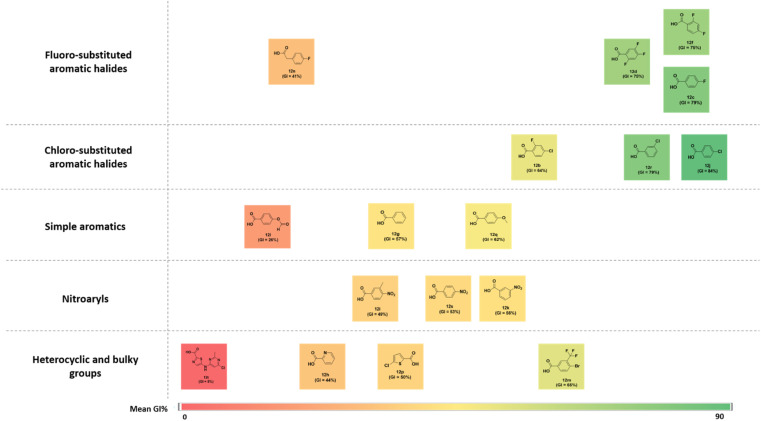
Structure–activity correlation chart for synthesized compounds (12a–12t) based on NCI-60 average growth inhibition percentage (%GI).

**Fig. 7:**
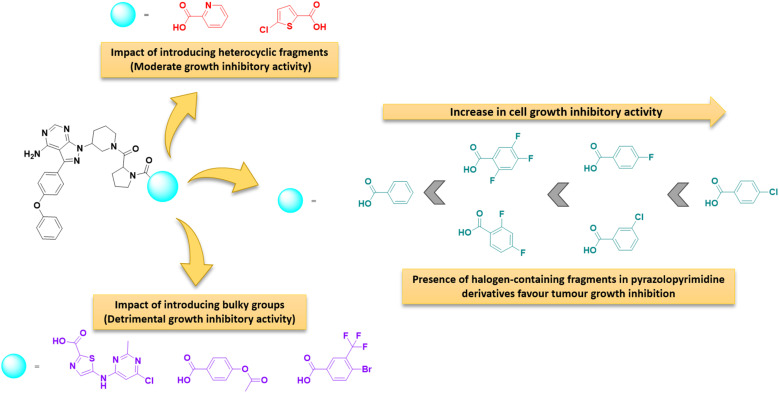
Overview of structure–activity relationship.

The structure–activity correlation of the compounds was possible by comparing the %GI activity data obtained from the single-dose study. The study provided insights into the cytotoxic properties of the compounds in the presence of different substitutions in the pyrazolopyrimidine core. A graphical representation of the structure–activity correlation is depicted in Fig. 6. The introduction of simple heterocyclic acids on compound 8, such as picolinic acid (12h) and 5-chlorothiophene-2-carboxylic acid (12p), showed only moderate activity. The presence of bulky heterocyclic groups such as 5-((6-chloro-2-methylpyrimidin-4-yl)amino)thiazole-2-carboxylic acid (12t) did not improve any activity. This data confirms that the presence of the heterocyclic moiety did not demonstrate any significant activity against cancer cell lines. On the other hand, introducing a simple benzoic acid (12g) provided an average growth inhibition of 57.2%. An activity comparison between 12l (GI = 5%) and 12m (GI = 65%) demonstrated that introducing halogen-substituted aromatic acids significantly improved the activity of the derivatives. Furthermore, introducing halogens at different positions of benzoic acid (as in 12d) showed better activity than a trifluoro –CF_3_ group (12m) substituted benzoic acid. The halogen substitution on benzoic acid showed good to better activity (as in 12c and 12j). Additionally, it was observed that replacing the fluorine with chlorine at the para position (as in compound 12j) resulted in the best activity profile with an average growth inhibition of 84%. Other substitutions such as –CH_3_, –NO_2_, –OPh, –OCH_3_ groups did not show any better activity. Fig. 7 summarizes the structure–activity correlation study.

## Materials and methods

### General procedures

The solvents and chemicals used in the spectroscopic studies were purchased from Sigma-Aldrich, Merck (Darmstadt, Germany). The melting points were determined in open capillary tubes using Labtronics Automatic Digital Melting Point Apparatus (Model: LT-109, Guelph, Canada). The molecular weights of the compounds were determined by understanding the mass spectra obtained using a mass spectrometer (Agilent 6550 tunnel QTOF Mass Spectrometer, California, USA). The purity of the compounds was confirmed by using Waters Binary HPLC System with a 1525 Binary HPLC Pump (Massachusetts, USA). ^1^H NMR and ^13^C NMR spectra were determined at 400 MHz and 100 MHz, respectively, using an NMR spectrometer (Bruker Ascend Avance III HD, Karlsruhe, Germany). Chemical shifts are expressed in *δ* (ppm).

### Chemistry

A five-step synthesis was used for the preparation of compound 8. The details for the same are reported elsewhere.^32^ The progress of the reaction and the formation of products was monitored using thin layer chromatography till the formation of compound 7777.

#### (*S*)-3-(3-Phenoxyphenyl)-1-(piperidin-3-yl)-1*H*-pyrazolo[3,4-*d*]pyrimidin-4-amine (8)


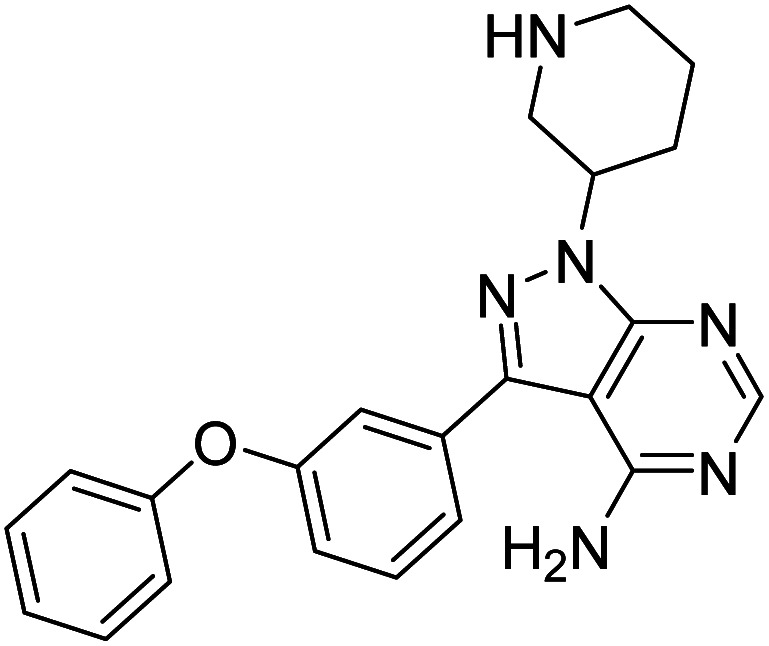
Compound 7 (61.65 mmol, 30 g) was dissolved in ethyl acetate and added with HCl (35–36%) at 50 °C, and stirred for 90 minutes. The solvent was evaporated under vacuum, residue, filtered and washed with ethyl acetate. The organic part was separated and evaporated under pressure to obtain compound 8.

Off-white powder; yield: 22.41 g (94%); m.p.: 142 °C; ^1^H NMR (DMSO-D_6_, 400 MHz): *δ* ppm 8.24 (1H, s, Ar–H), 7.65–7.67 (2H, d, *J* = 8 Hz), 7.42–7.46 (2H, t, *J* = 8 Hz, Ar–H), 7.12–7.21 (5H, m, Ar), 4.64–4.72 (1H, m, NH), 3.05–3.10 (1H, m, CH), 2.89–2.97 (2H, m, CH_2_), 2.45–2.48 (2H, m, CH_2_), 2.01–2.17 (2H, m, CH_2_), 1.52–1.77 (2H, m, CH_2_); ^13^C NMR (DMSO-D_6_, 100 MHz): *δ* ppm 158.6, 157.5, 156.8, 155.9, 154.2, 143.2, 130.6, 128.6, 124.2, 119.4, 97.8 54.4, 51.4, 45.9, 30.8 & 26.5; IR (KBr disc, cm^−1^): 3475 s, 3295 s (N–H), 2952 s (sp^2^ stretch), 2818 s (sp^3^ stretch), 1646 s (C=O stretch), 1589 s (N–H bend), 1519 m, 1489 s, 1489 s (C–H bend), 1232 s (C–N stretch), 1167 w (C–O stretch), 870 w (N–H oop).

#### *tert*-Butyl 3-((*S*)-3-(4-amino-3-(3-phenoxyphenyl)-1*H*-pyrazolo[3,4-*d*]pyrimidin-1-yl)piperidine-1-carbonyl)pyrrolidine-1-carboxylate (10)


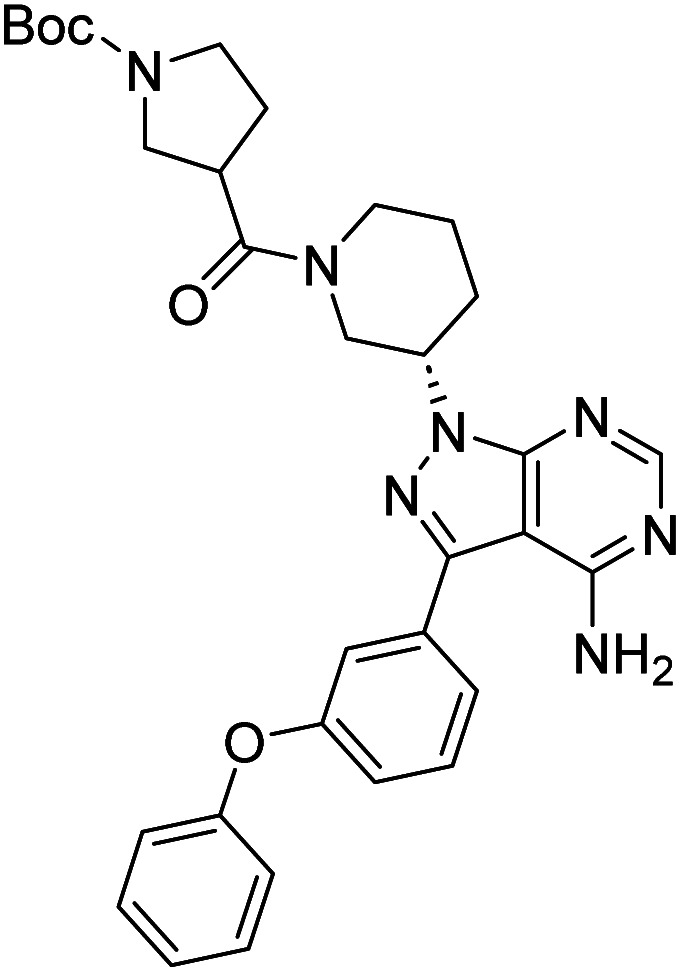
A solution of compound 8 (51.75 mmol, 20.45 g) in DMF was treated with Boc-proline 9 (51.75 mmol, 8 g) in the presence of triethylamine (14.5 mmol, 5.88 g) and TBTU (14.5 mmol, 18.8 g). The reaction mixture was stirred at 10–15 °C for 10 minutes. It was washed with water and ethyl acetate. The organic layer was separated and dried under a vacuum to obtain the title amine.

White powder; yield: 24.97 g (78%); m.p.: 145 °C; ^1^H NMR (DMSO-D_6_, 400 MHz): *δ* ppm 8.24–8.28 (1H, m, Ar), 7.65–7.67 (2H, d, *J* = 8 Hz, Ar–H), 7.42–7.46 (2H, t, *J* = 8 Hz, Ar–H), 7.12–7.21 (5H, m, Ar–H), 4.64–4.70 (1H, m, CH), 4.56–4.63 (1H, m, CH), 4.19–4.41 (2H, m, CH_2_), 3.29–3.32 (2H, m, CH_2_), 3.21–3.27 (2H, m, CH_2_), 2.12–2.26 (2H, m, CH_2_), 1.96–1.99 (2H, brs, CH_2_), 1.70–1.83 (4H, m, CH_2_), 1.40 (9H, s, CH_3_); ^13^C NMR (DMSO-D_6_, 100 MHz): *δ* ppm 170.8, 158.7, 157.5, 156.8, 156.2, 154.4, 153.7, 143.7, 130.6, 128.4, 124.2, 119.4, 97.8, 78.8, 57.2, 56.8, 53.5, 52.4, 46.8, 46.3, 45.2, 30.2, 29.3, 28.6, 25.1 & 23.2; IR (KBr disc, cm^−1^): 3478 s, 3300 s (N–H), 2951 s (sp^2^ stretch), 2864 s (sp^3^ stretch), 1628 s (C=O stretch), 1568 s (N–H bend), 1520 m, 1490 s (C–H bend), 1237 s (C–N stretch), 1165 w (C–O stretch), 872 w (N–H oop); mass: 584.33 [M + H]^+^.

#### ((*S*)-3-(4-Amino-3-(3-phenoxyphenyl)-1*H*-pyrazolo[3,4-*d*]pyrimidin-1-yl)piperidin-1-yl)(pyrrolidin-3-yl)methanone (11)


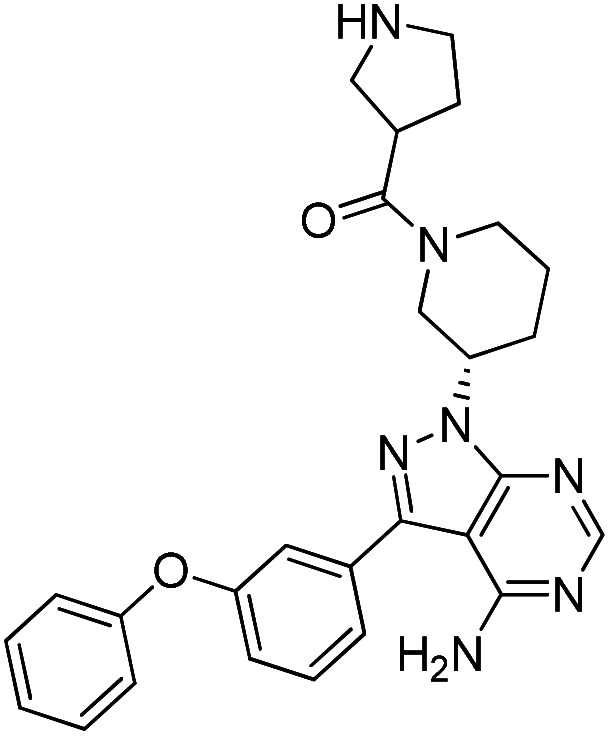
Compound 10 (16.57 mmol, 20 g) was dissolved in 1 M HCl in ethyl acetate (50 mL) and stirred at room temperature for 20 minutes. The reaction mixture was directly evaporated under a vacuum to obtain a white powder of the title amine that was then used for synthesizing the novel derivatives.

White powder; yield: 15 g (95%); m.p.: 148 °C; ^1^H NMR (DMSO-D_6_, 400 MHz): *δ* ppm 8.24–8.28 (1H, m, Ar–H), 7.65–7.68 (2H, m, Ar–H), 7.42–7.46 (2H, t, *J* = 8 Hz, Ar–H), 7.12–7.21 (5H, m, Ar), 4.68–4.75 (1H, m, NH), 4.31–4.41 (1H, m, CH), 4.09–4.12 (1H, m, CH), 3.89–3.93 (2H, m, CH_2_), 3.50–3.53 (2H, m, CH_2_), 3.18–3.33 (2H, m, CH_2_), 2.60–3.00 (2H, m, CH_2_), 2.10–2.33 (2H, m, CH_2_), 1.86–2.01 (2H, m, CH_2_), 1.67 (2H, m, CH_2_); ^13^C NMR (DMSO-D_6_, 100 MHz): *δ* ppm 172.2, 158.7, 157.5, 156.8, 156.1, 154.4, 143.7, 130.6, 128.4, 124.2, 119.5, 97.8, 66.8, 58.0, 52.3, 47.5, 46.2, 44.9, 42.0, 30.7, 29.9, 26.5 & 24.7; IR (KBr disc, cm^−1^): 3471 s, 3304 s (N–H), 2939 s (sp^2^ stretch), 2864 s (sp^3^ stretch), 1639 s (C=O stretch), 1586 s (N–H bend), 1521 s, 1487 s (C–H bend), 1230 s (C–N stretch), 1167 w (C–O stretch), 869 w (N–H oop); Mass: calcd for [C_27_H_29_N_7_O_2_]^+^ 483.56, found 482.26 [M–H].

#### General procedure for the synthesis of 12a–12t

The amine group in compound 11 was treated with various carboxylic acids to obtain derivatives 12a–12t. To a solution of compound 11 (0.62 mmol, 300 mg) in *N*,*N*-dimethyl formamide (3 mL) at 10–15 °C, TBTU (0.68 mmol, 220 mg) and triethylamine (0.68 mmol, 69 mg) were added. The reaction mixture was stirred for about 10 minutes, the amine was added, and stirring was continued for five minutes to one hour. The progress of the reaction was monitored by TLC. The reaction mixture was washed with dilute NaHCO_3_ solution. The precipitate so formed was filtered under vacuum, diluted with DCM, washed with hexane and distilled to get the title compounds.

##### 2,2,2-Trifluoro-*N*-(3-(4-phenoxyphenyl)-1-((3*R*)-1-(1-(2,2,2-trifluoroacetyl)pyrrolidine-2-carbonyl)piperidin-3-yl)-1*H*-pyrazolo[3,4-*d*]pyrimidin-4-yl)acetamide (12a)


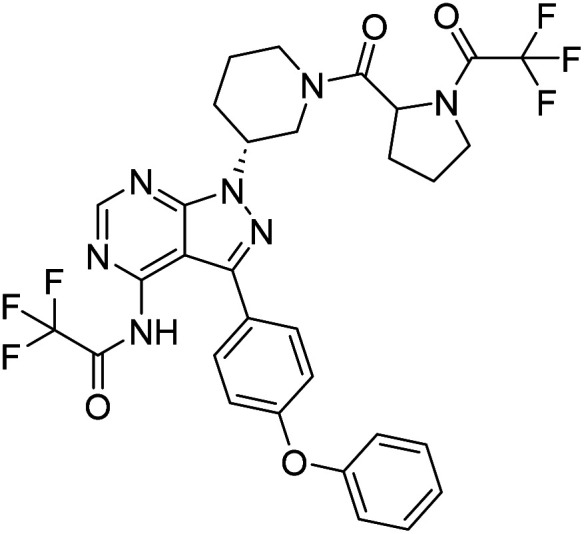
White powder; yield: 383.1 mg (91%); M.P.: 138 °C; ^1^H NMR (DMSO-D_6_, 400 MHz): *δ* ppm 8.23–8.31 (1H, m, Ar–H), 7.65–7.67 (1H, d, *J* = 8 Hz, Ar–H), 7.48–7.52 (1H, m, Ar–H), 7.42–7.46 (2H, t, *J* = 8 Hz, Ar–H), 7.11–7.21 (5H, m, Ar–H), 4.62–4.68 (1H, m, CH), 4.22–4.40 (1H, m, CH), 3.64–3.71 (2H, m, CH_2_), 3.47–3.57 (2H, m, CH_2_), 3.28–3.32 (2H, m, CH_2_), 2.09–2.24 (2H, m, CH_2_), 1.88–1.81 (2H, m, CH_2_), 1.69–1.75 (2H, m, CH_2_), 1.52–1.59 (2H, brs, CH_2_); ^13^C NMR (DMSO-D_6_, 100 MHz): *δ* ppm 172.5, 170.3, 166.9, 158.6, 157.5, 156.8, 156.1, 143.6, 130.6, 128.4, 124.2, 119.4, 97.7, 56.8, 52.2, 33.9, 31.4, 28.9, 25.2, 22.5, 21.5 & 14.4; IR (KBr disc, cm^−1^): 3486 s (N–H), 2929 s (sp^2^ stretch), 2859 s (sp^3^ stretch), 1634 s (C=O stretch), 1586 s (N–H bend), 1520 m, 1490 s, 1490 m, 1440 m (C–H bend), 1233 s (C–N stretch), 1151 w (C–O stretch), 849 w (N–H oop); HRMS: calcd for [C_31_H_27_F_6_N_7_O_4_]^+^ 675.5924, found 678.2410 [M − H + 2]^+^, considering the isotopic fluorine substitution.

##### 4-Amino-1-((3*R*)-1-((4-chloro-2-fluorobenzoyl)prolyl)piperidin-3-yl)-3-(4-phenoxyphenyl)-1*H*-pyrazolo[3,4-*d*]pyrimidine (12b)


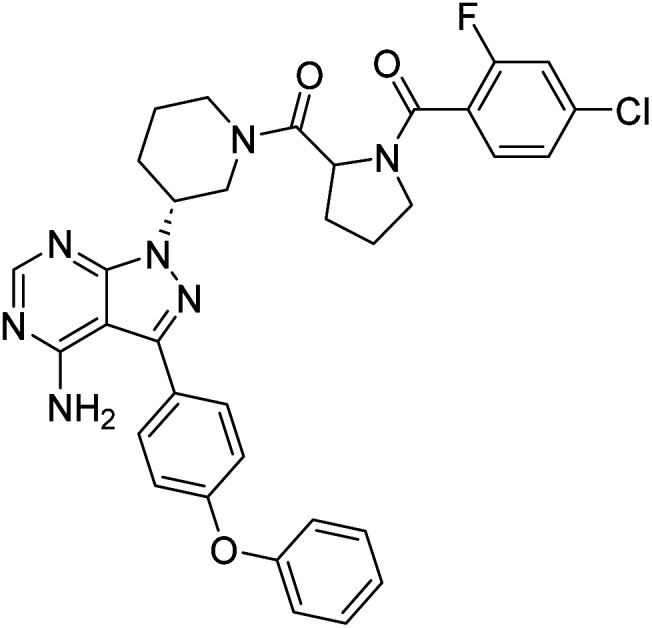
Cream colour powder; yield: 361.15 mg (91%); M.P.: 145 °C; ^1^H NMR (DMSO-D_6_): *δ* = ^1^H NMR (DMSO-D_6_, 400 MHz): *δ* ppm 8.25–8.36 (1H, m, Ar–H), 7.64–7.67 (2H, t, *J* = 8 Hz, Ar–H), 7.55–7.59 (1H, m, Ar–H), 7.39–7.45 (4H, m, Ar–H), 7.11–7.20 (5H, m, Ar), 4.92–5.04 (1H, m, CH), 4.61–4.73 (1H, m, CH), 4.19–4.33 (2H, m, CH), 3.58 (2H, brs, CH_2_), 3.30–3.32 (2H, brs, CH_2_), 2.26–2.32 (2H, brs, CH_2_), 2.00–2.20 (2H, m, CH_2_), 1.99–2.20 (2H, m, CH_2_), 1.72–1.91 (4H, m, CH_2_); ^13^C NMR (DMSO-D_6_, 100 MHz): *δ* ppm 158.6, 156.8, 143.8, 130.6, 128.4, 125.7, 124.2, 119.4, 56.9, 52.2, 32.0, 30.6, 29.5, 29.2, 24.7, 22.6 & 14.4; IR (KBr disc, cm^−1^): 3475 s, 3317 s (N–H), 3206 s (sp^2^ stretch), 2925 s (sp^3^ stretch), 1632 s (C=O stretch), 1588 s (N–H bend), 1521 m, 1478 m, 1438 m (C–H bend), 1222 s (C–N stretch), 1166 w (C–O stretch), 860 w (N–H oop), 801 w (C–Cl stretch); HRMS: calcd for [C_34_H_31_ClFN_7_O_3_]^+^ 640.1164, found 640.2217 [M + 1]^+^ and 662.2043 [M + 1 + Na]^+^.

##### 4-Amino-1-((3*R*)-1-((4-fluorobenzoyl)prolyl)piperidin-3-yl)-3-(4-phenoxyphenyl)-1*H*-pyrazolo[3,4-*d*]pyrimidine (12c)


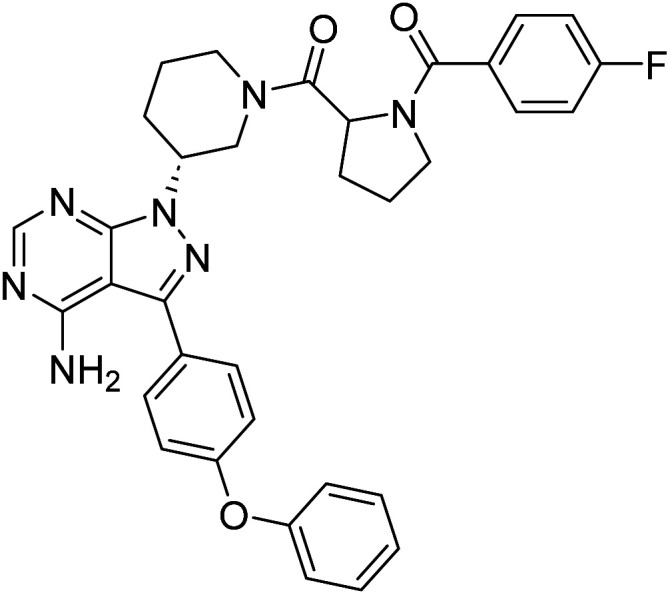
White powder; yield: 337.96 mg (90%); M.P.: 147 °C; ^1^H NMR (DMSO-D_6_, 400 MHz): *δ* ppm 8.24–8.33 (1H, m, Ar–H), 7.55–7.70 (4H, m, Ar–H), 7.42–7.45 (2H, t, *J* = 8 Hz, Ar–H), 7.36–7.39 (1H, m, Ar–H), 7.27–7.31 (1H, t, *J* = 8 Hz, Ar–H), 7.11–7.21 (5H, m, Ar–H), 4.91–5.04 (1H, m, CH), 4.72–4.79 (1H, m, CH), 4.25–4.46 (2H, m, CH_2_), 3.48–3.62 (2H, m, CH_2_), 3.30 (2H, brs, CH_2_), 2.08–2.29 (2H, m, CH_2_), 1.91–2.01 (4H, m, CH_2_), 1.75–1.77 (2H, brs, CH_2_); ^13^C NMR (DMSO-D_6_, 100 MHz): *δ* ppm 172.5, 170.2, 2.5, 170.2, 167.1, 158.7, 156.8, 156.1, 143.7, 133.5, 130.6, 130.2, 129.5, 128.4, 124.2, 119.5, 115.8, 97.7, 60.0, 57.2, 50.2, 45.6, 29.5, 29.0, 25.5 & 21.5; IR (KBr disc, cm^−1^): 3476 s, 3304 s (N–H), 3186 s (sp^2^ stretch), 2924 s (sp^3^ stretch), 1621 s (C=O stretch), 1587 s (N–H bend), 1520 m, 1477 s, 1489 s (C–H bend), 1221 s (C–N stretch), 1141 w (C–O stretch), 862 w (N–H oop); HRMS: calcd for [C_34_H_32_FN_7_O_3_]^+^ 605.6744, found 606.2617 [M + 1]^+^ and 638.2439 [M + 1 + Na]^+^.

##### 4-Amino-3-(4-phenoxyphenyl)-1-((3*R*)-1-((2,4,5-trifluorobenzoyl)prolyl)piperidin-3-yl)-1*H*-pyrazolo[3,4-*d*]pyrimidine (12d)


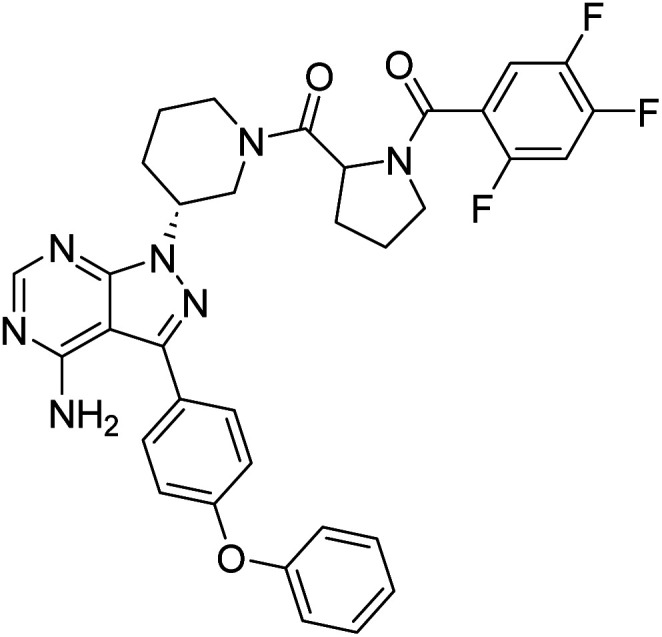
White powder; yield: 366.0 mg (92%); M.P.: 143 °C; ^1^H NMR (DMSO-D_6_, 400 MHz): *δ* ppm 8.25–8.31 (1H, m, Ar–H), 7.66–7.68 (2H, m, Ar–H), 7.44 (3H, brs, Ar–H), 7.28 (1H, brs, Ar–H), 7.14–7.17 (5H, m, Ar–H), 4.95–5.05 (1H, m, CH), 4.75 (1H, brs, CH), 4.15–4.45 (2H, m, CH_2_), 3.51–3.69 (2H, m, CH_2_), 2.95–3.15 (2H, m, CH_2_), 2.26–2.33 (2H, m, CH_2_), 1.90–2.18 (4H, brs, CH_2_), 1.75–1.81 (2H, m, CH_2_); ^13^C NMR (DMSO-D_6_, 100 MHz): *δ* ppm 170.0, 158.7, 157.5, 156.8, 156.2, 154.3, 143.8, 130.6, 128.3, 124.4, 119.4, 52.2, 49.0, 45.5, 30.6, 29.5, 24.7, 22.9, 17.7 & 14.2; IR (KBr disc, cm^−1^): 3476 s, 3317 s (N–H), 3206 s (sp^2^ stretch), 2925 s (sp^3^ stretch), 1632 s (C=O stretch), 1588 s (N–H bend), 1519 m, 1478 s, 1438 s (C–H bend), 1222 s (C–N stretch), 1141 w (C–O stretch), 860 w (N–H oop); HRMS: calcd for [C_34_H_30_F_3_N_7_O_3_]^+^ 641.6552, found 642.2437 [M + H]^+^.

##### 4-Amino-1-((3*R*)-1-((4-chloropicolinoyl)prolyl)piperidin-3-yl)-3-(4-phenoxyphenyl)-1*H*-pyrazolo[3,4-*d*]pyrimidine (12e)


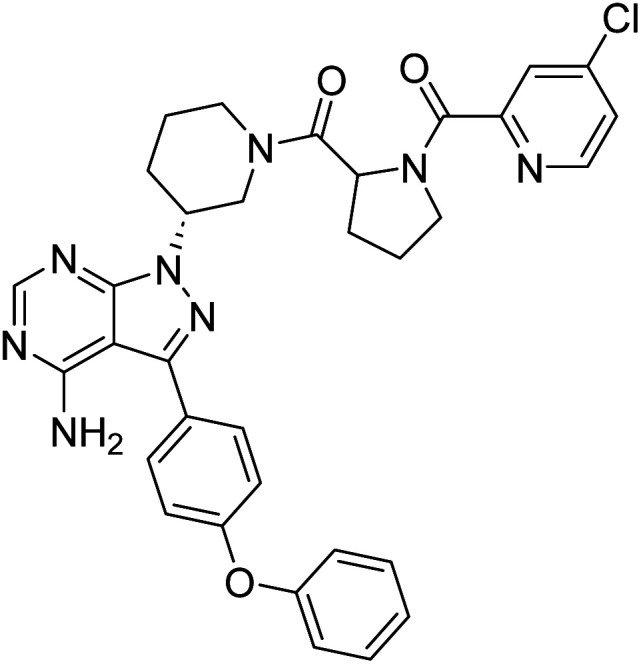
White powder; yield: 328.4 mg (85%); M.P.: 155 °C; ^1^H NMR (DMSO-D_6_, 400 MHz): *δ* ppm 8.60–8.65 (1H, m, Ar–H), 8.25–8.29 (1H, m, Ar–H), 7.82–7.84 (1H, m, Ar), 7.63–7.74 (3H, m, Ar–H), 7.42–7.46 (2H, t, *J* = 8 Hz, Ar–H), 7.11–7.19 (5H, m, Ar), 5.48–5.56 (1H, m, CH), 4.72–5.08 (1H, m, CH), 4.17–4.36 (2H, m, CH_2_), 3.62–3.89 (2H, m, CH_2_), 2.33–2.38 (2H, m, CH_2_), 2.12–2.20 (2H, m, CH_2_), 1.91 (2H, brs, CH_2_), 1.79–1.87 (4H, m, CH_2_); ^13^C NMR (DMSO-D_6_, 100 MHz): *δ* ppm 172.5, 170.3, 164.7, 158.7, 156.8, 156.1, 155.7, 154.3, 150.2, 149.6, 144.5, 143.7, 130.6, 128.4, 126.0, 124.3, 119.5, 97.8, 60.0, 52.2, 49.9, 48.8, 44.9, 31.4, 29.5, 21.5 & 14.4; IR (KBr disc, cm^−1^): 3470 s, 3398 s (N–H), 2922 s (sp^2^ stretch), 2856 s (sp^3^ stretch), 1628 s (C=O stretch), 1570 s (N–H bend), 1489–1438 s (C–H bend), 1236 s (C–N stretch), 1166 w (C–O stretch), 845 w (N–H oop), 802 w (C–Cl); HRMS: calcd for [C_33_H_31_ClN_8_O_3_]^+^ 623.1140, found 623.2252 [M]^+^ and 625.2246 [M + 2]^+^.

##### 4-Amino-1-((3*R*)-1-((2,4-difluorobenzoyl)prolyl)piperidin-3-yl)-3-(4-phenoxyphenyl)-1*H*-pyrazolo[3,4-*d*]pyrimidine (12f)


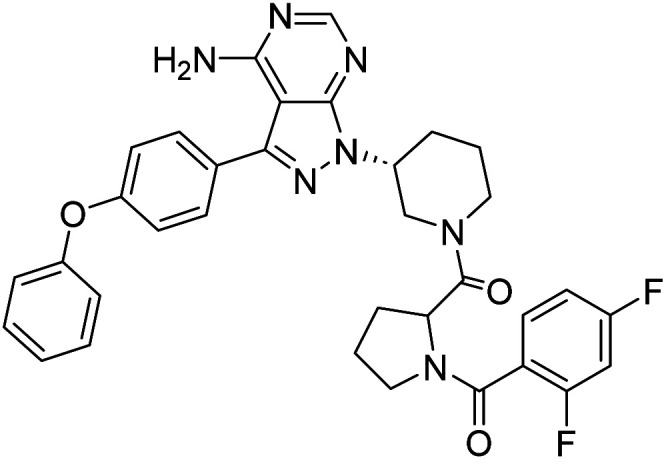
White powder; yield: 367.3 mg (95%); M.P.: 146 °C; ^1^H NMR (DMSO-D_6_, 400 MHz): *δ* ppm 8.25–8.35 (1H, m, Ar–H), 7.64–7.70 (2H, m, Ar–H), 7.41–7.45 (3H, t, *J* = 8 Hz, Ar–H), 7.33–7.38 (1H, m, Ar–H), 7.11–7.21 (6H, m, Ar–H), 4.91–5.05 (1H, m, CH), 4.63–4.74 (1H, m, CH), 4.21–4.46 (2H, m, CH_2_), 3.57–3.62 (2H, m, CH_2_), 3.29–3.32 (2H, m, CH_2_), 2.14–2.32 (2H, m, CH_2_), 1.91–2.03 (2H, m, CH_2_), 1.73–1.85 (4H, m, CH_2_); ^13^C NMR (DMSO-D_6_, 100 MHz): *δ* ppm 170.1, 169.9, 164.1, 163.1, 158.7, 157.7, 156.8, 154.4, 130.6, 128.4, 124.2, 119.5, 105.0, 97.7, 58.9, 56.9, 52.2, 47.1, 45.6, 30.0, 29.1, 24.7 & 22.9; IR (KBr disc, cm^−1^): 3477 s, 3306 s (N–H), 2951 s (sp^2^ stretch), 2855 s (sp^3^ stretch), 1623 s (C=O stretch), 1567 s (N–H bend), 1521 m, 1487 s, 1427 s (C–H bend), 1239 s (C–N stretch), 1089 w (C–O stretch), 842 w (N–H oop); HRMS: calcd for [C_34_H_31_F_2_N_7_O_3_]^+^ 623.6648, found 624.2516 [M + 1]^+^ and 625.2538 [M + 2]^+^.

##### 4-Amino-1-((3*R*)-1-(benzoylprolyl)piperidin-3-yl)-3-(4-phenoxyphenyl)-1*H*-pyrazolo[3,4-*d*]pyrimidine (12g)


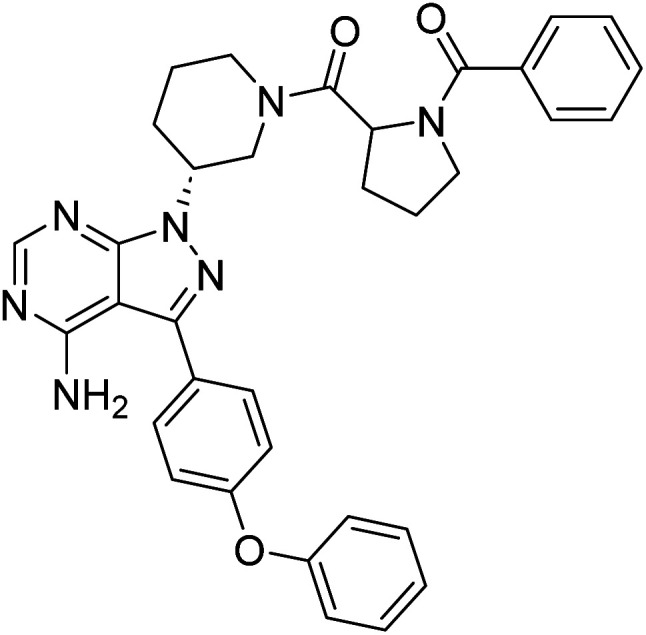
White powder; yield: 302.4 mg (83%); M.P.: 148 °C; ^1^H NMR (DMSO-D_6_, 400 MHz): *δ* ppm 8.24–8.34 (1H, m, Ar–H), 7.64–7.68 (2H, t, *J* = 8 Hz, Ar–H), 7.52–7.53 (2H, m, Ar–H), 7.44–7.47 (4H, t, *J* = 8 Hz, Ar–H), 7.35–7.36 (1H, d, *J* = 8 Hz, Ar–H), 7.14–7.16 (5H, m, Ar–H), 4.93–5.04 (1H, m, CH), 4.74 (1H, brs, CH), 4.02–4.47 (2H, m, CH_2_), 3.47–3.60 (2H, m, CH_2_), 2.95–3.12 (2H, m, CH_2_), 2.26–2.30 (2H, m, CH_2_), 1.88–2.16 (4H, m, CH_2_), 1.76 (2H, brs, CH_2_); ^13^C NMR (DMSO-D_6_, 100 MHz): *δ* ppm 169.8, 168.1, 158.7, 157.5, 156.8, 156.1, 154.3, 137.2, 130.6, 128.7, 127.5, 127.0, 124.2, 119.5, 97.8, 59.5, 57.0, 52.2, 50.2, 45.7, 30.0, 29.0, 25.4 & 24.7; IR (KBr disc, cm^−1^): 3471 s, 3301 s (N–H), 2926 s (sp^2^ stretch), 2959 s (sp^3^ stretch), 1626 s (C=O stretch), 1568 s (N–H bend), 1523 m, 1476 s, 1434 s (C–H bend), 1221 s (C–N stretch), 1141 w (C–O stretch), 861 w (N–H oop); HRMS: calcd for [C_34_H_33_N_7_O_3_] 587.6840, found 588.2705 [M + H]^+^.

##### 4-Amino-3-(4-phenoxyphenyl)-1-((3*R*)-1-(picolinoylprolyl)piperidin-3-yl)-1*H*-pyrazolo[3,4-*d*]pyrimidine (12h)


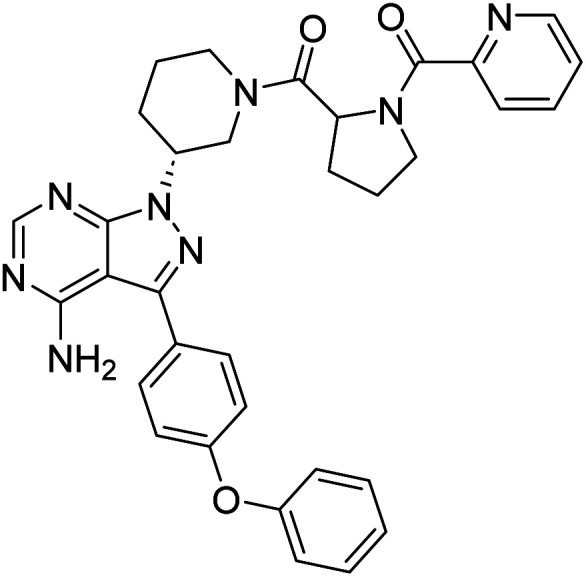
Yellowish-white powder; yield: 302.9 mg (83%); M.P.: 156 °C; ^1^H NMR (DMSO-D_6_, 400 MHz): *δ* ppm 8.60–8.65 (1H, m, Ar–H), 8.24–8.29 (1H, m, Ar–H), 7.93–8.01 (1H, m, Ar–H), 7.76–7.83 (1H, m, Ar–H), 7.64–7.69 (2H, m, Ar–H), 7.50–7.58 (1H, m, Ar–H), 7.42–7.46 (2H, t, *J* = 8 Hz, Ar–H), 7.12–7.21 (5H, m, Ar–H), 5.55–5.56 (1H, m, CH), 4.71–4.76 (1H, m, CH), 4.24–4.38 (2H, m, CH_2_), 3.62–3.87 (2H, m, CH_2_), 3.14–3.33 (2H, m, CH_2_), 2.45–2.29 (2H, m, CH_2_), 2.11–2.21 (2H, m, CH_2_), 1.88–1.99 (2H, m, CH_2_), 1.68–1.79 (2H, m, CH_2_); ^13^C NMR (DMSO-D_6_, 100 MHz): *δ* ppm 170.5, 165.9, 158.7, 157.5, 156.8, 156.1, 154.2, 148.5, 147.9, 143.7, 137.8, 130.6, 128.4, 125.7, 124.6, 124.2, 119.4, 97.7, 59.6, 52.3, 49.9, 48.5, 44.9, 31.4, 30.0, 25.4 & 22.0; IR (KBr disc, cm^−1^): 3466 s, 3397 s (N–H), 2925 s (sp^2^ stretch), 2855 s (sp^3^ stretch), 1623 s (C=O stretch), 1587 s (N–H bend), 1523 m, 1489 s, 1425 s (C–H bend), 1235 s (C–N stretch), 1133 w (C–O stretch), 801 w (N–H oop); HRMS: calcd for [C_33_H_32_N_8_O_3_]^+^ 588.6720, found 589.2654 [M + H]^+^.

##### 4-Amino-1-((3*R*)-1-((3-methyl-4-nitrobenzoyl)prolyl)piperidin-3-yl)-3-(4-phenoxyphenyl)-1*H*-pyrazolo[3,4-*d*]pyrimidine (12i)


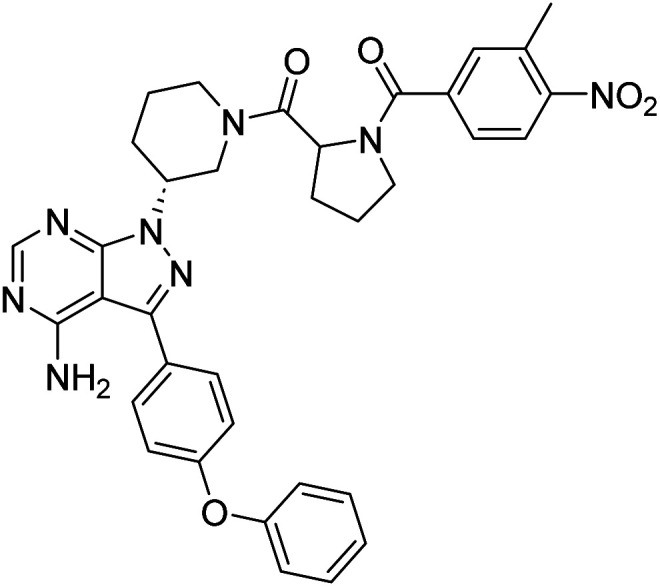
Yellowish-white powder; yield: 316.7 mg (80%); M.P.: 152 °C; ^1^H NMR (DMSO-D_6_, 400 MHz): *δ* ppm 8.24–8.30 (1H, m, Ar), 7.97–8.07 (1H, m, Ar), 7.77–7.83 (1H, m, Ar–H), 7.66–7.71 (2H, m, Ar–H), 7.60–7.65 (1H, m, Ar–H), 7.41–7.45 (2H, t, *J* = 8 Hz, Ar–H), 7.11–7.20 (5H, m, Ar–H), 4.96–5.05 (1H, m, CH), 4.72–4.75 (1H, m, CH), 3.92–4.46 (2H, m, CH_2_), 3.51–3.64 (2H, m, CH_2_), 2.64–2.57 (2H, m, CH_2_), 2.16–2.33 (2H, m, CH_2_), 1.99–2.03 (2H, m, CH_2_), 1.78–1.91 (4H, m, CH_2_), 1.23 (3H, s, CH_3_); ^13^C NMR (DMSO-D_6_, 100 MHz): *δ* ppm 170.1, 165.8, 158.6, 157.5, 156.8, 156.0, 149.1, 143.7, 137.1, 136.0, 134.6, 133.5, 132.1, 130.6, 128.3, 124.4, 123.5, 119.5, 97.7, 60.0, 57.4, 52.1, 50.1, 47.6, 31.4, 22.5, 22.5, 20.0 & 14.4; IR (KBr disc, cm^−1^): 3466 s (N–H), 2925 s (sp^2^ stretch), 2855 s (sp^3^ stretch), 1623 s (C=O stretch), 1587 s (N–H bend), 1523 m, 1489 s (C–H bend), 1235 s (C–N stretch), 1133 w (C–O stretch), 801 w (N–H oop); HRMS: calcd for [C_35_H_34_N_8_O_5_]^+^ 646.7080, found 647.2712 [M + H]^+^.

##### 4-Amino-1-((3*R*)-1-((4-chlorobenzoyl)prolyl)piperidin-3-yl)-3-(4-phenoxyphenyl)-1*H*-pyrazolo[3,4-*d*]pyrimidine (12j)


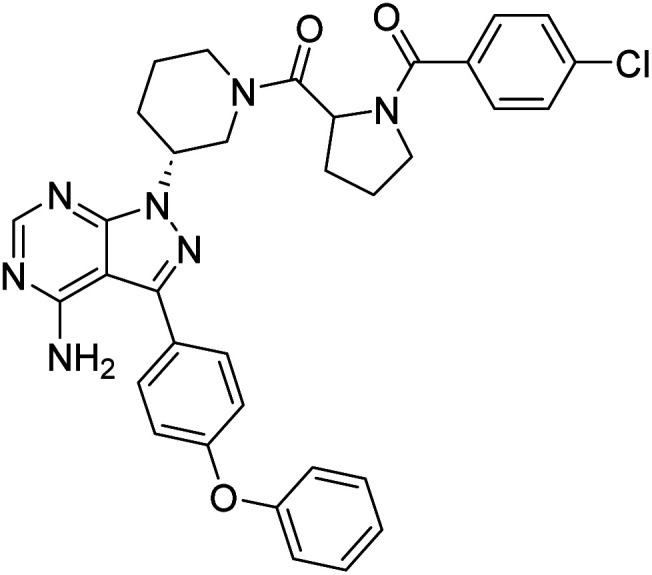
White powder; yield: 339.4 mg (88%); M.P.: 148 °C; ^1^H NMR (DMSO-D_6_, 400 MHz): *δ* ppm 8.23–8.34 (1H, m, Ar–H), 7.61–7.70 (3H, m, Ar–H), 7.51–7.57 (2H, m, Ar–H), 7.42–7.45 (2H, t, *J* = 8 Hz, Ar–H), 7.37–7.39 (1H, d, *J* = 8 Hz, Ar–H), 7.11–7.20 (5H, m, Ar–H), 5.01–5.04 (1H, m, CH), 4.72–4.77 (1H, m, CH), 4.24–4.35 (1H, m, CH), 3.46–3.62 (2H, m, CH_2_), 2.26–2.33 (2H, m, CH_2_), 2.10–2.16 (2H, m, CH_2_), 1.75–1.91 (4H, m, CH_2_), 1.47–1.60 (2H, m, CH_2_); ^13^C NMR (DMSO-D_6_, 100 MHz): *δ* ppm 170.6, 158.7, 156.8, 156.1, 130.6, 129.6, 129.1, 129.0, 128.8, 128.4, 124.4, 119.5, 57.1, 52.2, 34.7, 31.4, 29.0, 26.8, 25.2, 22.5 & 14.4; IR (KBr disc, cm^−1^): 3477 s, 3306 s (N–H), 2951 s (sp^2^ stretch), 2866 s (sp^3^ stretch), 1623 s (C=O stretch), 1566 s (N–H bend), 1521 m, 1427 s (C–H bend), 1239 s (C–N stretch), 1167 w (C–O stretch), 844 w (N–H oop), 803 w (C–Cl stretch); HRMS: calcd for [C_34_H_32_ClN_7_O_3_]^+^ 622.1260, found 622.2297 [M]^+^ and 644.2124 [M + Na]^+^.

##### 4-Amino-1-((3*R*)-1-((3-nitrobenzoyl)prolyl)piperidin-3-yl)-3-(4-phenoxyphenyl)-1*H*-pyrazolo[3,4-*d*]pyrimidine (12k)


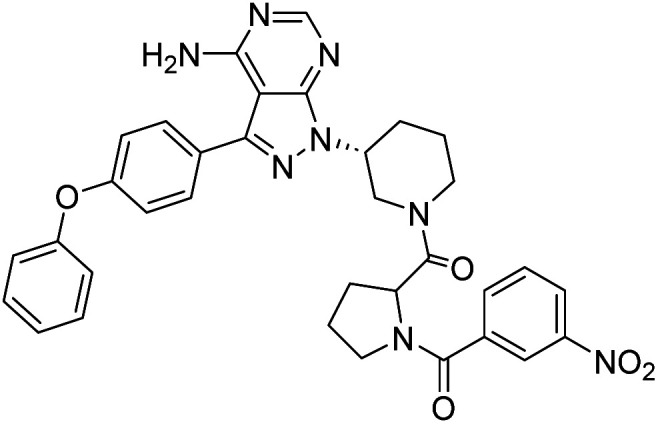
Yellowish-white powder; yield: 321.6 mg (82%); M.P.: 152 °C; ^1^H NMR (DMSO-D_6_, 400 MHz): *δ* ppm 8.34–8.39 (1H, m, Ar), 8.16–8.29 (1H, m, Ar), 7.89–8.03 (1H, m, Ar–H), 7.76–7.84 (1H, m, Ar–H), 7.61–7.70 (2H, m, Ar–H), 7.41–7.44 (2H, t, *J* = 8 Hz, Ar–H), 7.11–7.18 (5H, m, Ar–H), 4.97–5.07 (1H, m, CH), 4.63–4.76 (1H, m, CH), 3.89–4.47 (2H, m, CH_2_), 3.60–3.63 (2H, m, CH_2_), 3.40–3.49 (2H, m, CH_2_), 2.16–2.34 (2H, m, CH_2_), 1.93–2.06 (2H, m, CH_2_), 1.78–1.86 (4H, m, CH_2_); ^13^C NMR (DMSO-D_6_, 100 MHz): *δ* ppm 170.1, 166.0, 158.7, 157.5, 156.8, 156.2, 154.3, 148.0, 143.7, 139.6, 138.5, 133.9, 130.6, 128.3, 125.1, 124.2, 122.3, 119.5, 97.7, 59.6, 57.4, 52.0, 47.6, 46.5, 31.4, 25.4, 22.5 & 14.4; IR (KBr disc, cm^−1^): 3476 s, 3306 s (N–H), 2946 s (sp^2^ stretch), 2869 s (sp^3^ stretch), 1624 s (C=O stretch), 1568 s (N–H bend), 1531 m, 1489 s (C–H bend), 1349 s (N–O stretch), 1236 s (C–N stretch), 1164 w (C–O stretch), 868 w (N–H oop); HRMS: calcd for [C_34_H_32_N_8_O_5_]^+^ 632.6810, found 633.2564 [M + 1]^+^.

##### 4-(2-((*R*)-3-(4-amino-3-(4-phenoxyphenyl)-1*H*-pyrazolo[3,4-*d*]pyrimidin-1-yl)piperidine-1-carbonyl)pyrrolidine-1-carbonyl)phenyl acetate (12l)


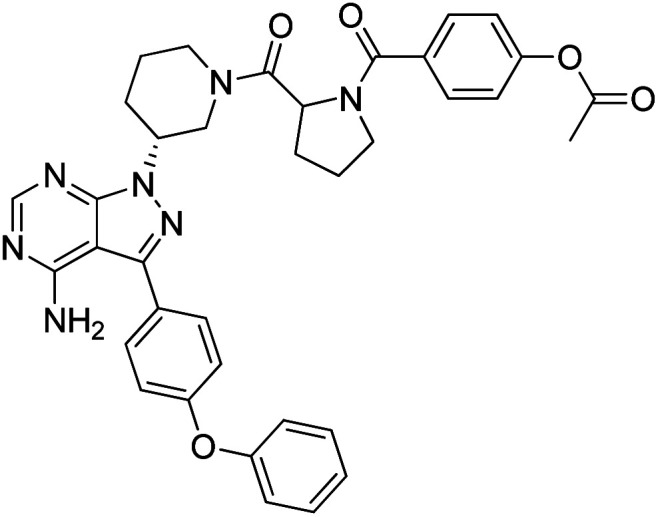
White powder; yield: 86%; M.P.: 344.3 mg (86%); ^1^H NMR (DMSO-D_6_, 400 MHz): *δ* ppm 8.22–8.30 (2H, m, Ar–H), 7.65–7.67 (3H, m, Ar–H), 7.39–7.46 (4H, m, Ar–H), 7.12–7.20 (5H, m, Ar–H), 4.95–5.06 (1H, m, CH), 4.70–4.83 (1H, m, CH), 4.27–4.36 (2H, m, CH_2_), 3.89–4.06 (2H, m, CH_2_), 3.48–3.60 (2H, m, CH_2_), 3.18–3.24 (2H, m, CH_2_), 2.24–2.33 (2H, m, CH_2_), 2.10–2.13 (2H, m, CH_2_), 1.91–1.96 (3H, m, CH_3_), 1.78–1.83 (2H, m, CH_2_), 1.56–1.68 (2H, m, CH_2_); ^13^C NMR (DMSO-D_6_, 100 MHz): *δ* ppm 170.7, 158.7, 157.4, 156.8, 154.4, 132.2, 130.6, 128.4, 124.2, 123.1, 122.3, 119.5, 97.7, 56.2, 47.9, 46.5, 34.6, 31.4, 30.0, 25.2, 24.6 & 22.7; IR (KBr disc, cm^−1^): 3471 s, 3301 s (N–H), 2926 s (sp^2^ stretch), 2859 s (sp^3^ stretch), 1626 s (C=O stretch), 1568 s (N–H bend), 1523 m, 1476 s, 1434 w (C–H bend), 1221 s (C–N stretch), 1141 w (C–O stretch), 861 w (N–H oop); HRMS: calcd for [C_36_H_35_N_7_O_5_]^+^ 645.2760, found 646.2770 [M + H]^+^.

##### 4-Amino-1-((3*R*)-1-((4-bromo-3-(trifluoromethyl)benzoyl)prolyl)piperidin-3-yl)-3-(4-phenoxyphenyl)-1*H*-pyrazolo[3,4-*d*]pyrimidine (12m)


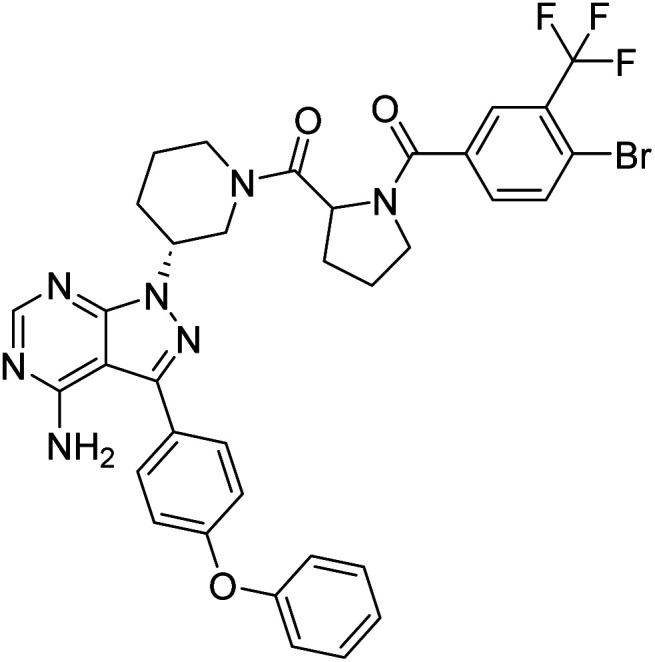
White powder; yield: 437.2 mg (96%); M.P.: 146 °C; ^1^H NMR (DMSO-D_6_, 400 MHz): *δ* ppm 8.23–8.33 (1H, m, Ar–H), 7.98–8.01 (1H, t, *J* = 8 Hz, Ar–H), 7.82–7.87 (1H, m, Ar–H), 7.75–7.79 (1H, m, Ar–H), 7.65–7.70 (2H, m, Ar–H), 7.41–7.46 (2H, t, *J* = 8 Hz, Ar–H), 7.11–7.20 (5H, m, Ar–H), 4.84–4.95 (1H, m, CH), 4.70–4.75 (1H, m, CH), 4.17–4.34 (2H, m, CH_2_), 3.48–3.60 (2H, m, CH_2_), 3.07–3.20 (2H, m, CH_2_), 2.23–2.32 (2H, m, CH_2_), 1.89–2.15 (2H, m, CH_2_), 1.77–1.85 (2H, m, CH_2_); ^13^C NMR (DMSO-D_6_, 100 MHz): *δ* ppm 170.0, 169.6, 158.7, 157.5, 156.8, 156.2, 143.7, 136.9, 135.8, 133.1, 130.6, 128.4, 124.2, 119.5, 97.7, 57.4, 52.2, 46.5, 30.0, 29.5, 29.0, 25.4 & 22.8; IR (KBr disc, cm^−1^): 3468 s (N–H), 2948 s (sp^2^ stretch), 2870 s (sp^3^ stretch), 1629 s (C=O stretch), 1567 s (N–H bend), 1490 s, 1441 m (C–H bend), 1312 m (C–F stretch), 1236 s (C–N stretch), 1135 w (C–O stretch), 853 w (N–H oop); HRMS: calcd for [C_35_H_31_BrF_3_N_7_O_3_]^+^ 734.5782, found 734.1652 [M]^+^ and 756.1491 [M + Na]^+^.

##### 1-(2-((*R*)-3-(4-Amino-3-(4-phenoxyphenyl)-1*H*-pyrazolo[3,4-*d*]pyrimidin-1-yl)piperidine-1-carbonyl)pyrrolidin-1-yl)-2-(4-fluorophenyl)ethanone (12n)


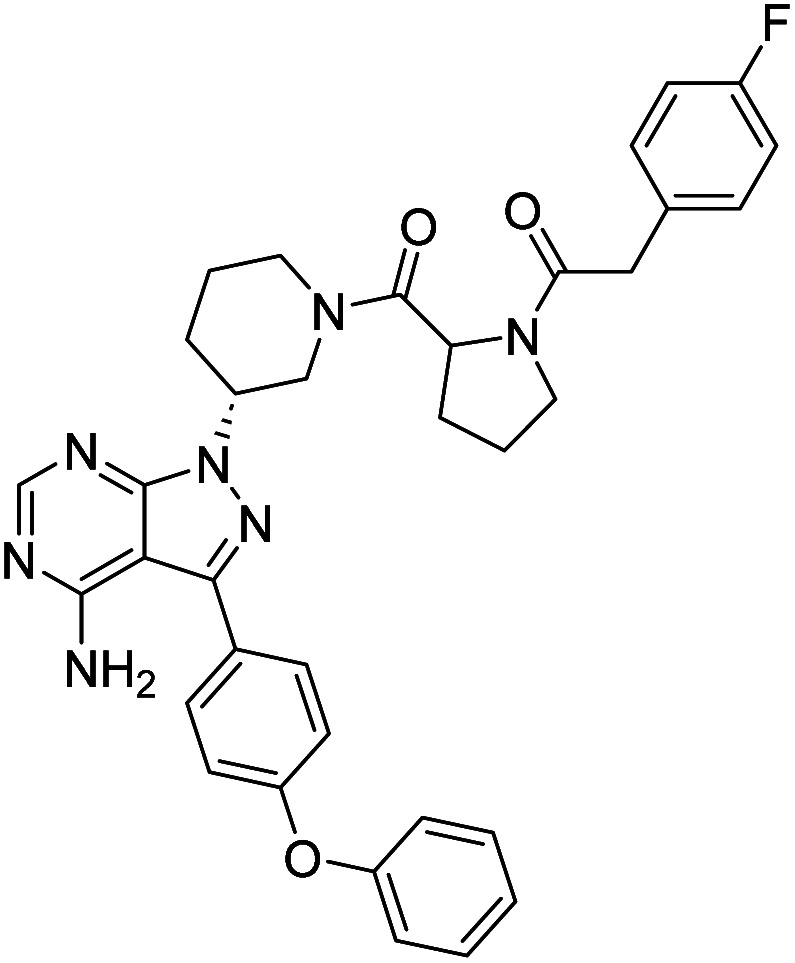
White powder; yield: 361.2 mg (94%); M.P.: 132 °C; ^1^H NMR (DMSO-D_6_, 400 MHz): *δ* ppm 8.23–8.32 (1H, m, Ar–H), 7.91–8.06 (1H, m, Ar–H), 7.62–7.67 (2H, m, Ar–H), 7.42–7.46 (1H, t, *J* = 8 Hz, Ar–H), 7.24–7.34 (2H, m, Ar–H), 7.11–7.21 (6H, m, Ar–H), 6.98–7.07 (1H, m, Ar–H), 4.66–4.76 (1H, m, CH), 4.27–4.37 (1H, m, CH), 3.64–3.81 (2H, m, CH_2_), 3.52–3.56 (2H, m, CH_2_), 3.36–3.37 (2H, m, CH_2_), 2.26 (2H, brs, CH_2_), 2.10–2.13 (2H, m, CH_2_), 1.91–1.97 (2H, m, CH_2_), 1.76–1.80 (2H, m, CH_2_), 1.30–1.35 (2H, m, CH_2_); ^13^C NMR (DMSO-D_6_, 100 MHz): *δ* ppm 170.5, 168.6, 157.6, 156.8, 155.4, 154.1, 143.9, 136.8, 135.5, 134.2, 132.2, 131.6, 130.8, 130.6, 129.2, 127.1, 126.5, 124.3, 119.4, 115.4, 97.7, 70.4, 60.0, 56.6, 52.3, 47.5, 34.8, 32.0, 22.6, 18.4 & 14.4; IR (KBr disc, cm^−1^): 3468 s, 3326 s (N–H), 2924 s (sp^2^ stretch), 2855 s s (sp^3^ stretch), 1639 s (C=O stretch), 1575 s (N–H bend), 1517 m, 1450 s (C–H bend), 1233 s (C–N stretch), 1164 w (C–O stretch), 871 w (N–H oop); HRMS: [M + H]^+^ calcd for [C_35_H_34_FN_7_O_3_]^+^ 619.7014, found 620.38 [M + H]^+^.

##### 4-Amino-1-((3*R*)-1-((3-methylbenzoyl)prolyl)piperidin-3-yl)-3-(4-phenoxyphenyl)-1*H*-pyrazolo[3,4-*d*]pyrimidine (12o)


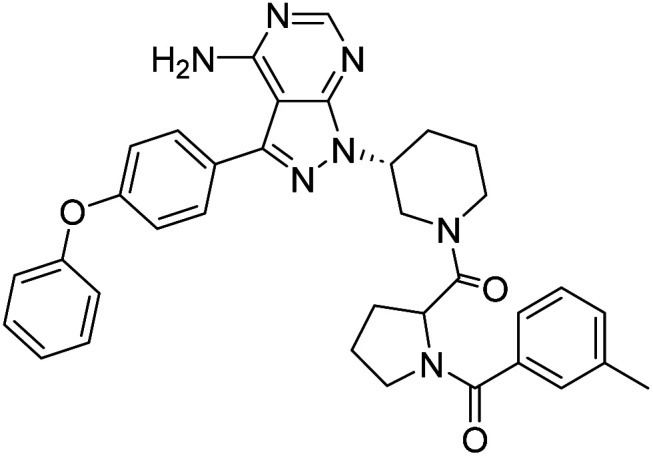
Yellow powder; yield: 302.2 mg (81%); M.P.: 144 °C; ^1^H NMR (DMSO-D_6_, 400 MHz): *δ* ppm 8.17–8.30 (1H, m, Ar–H), 7.64–7.68 (2H, t, *J* = 8 Hz, Ar–H), 7.41–7.45 (2H, t, *J* = 8 Hz, Ar–H), 7.28–7.31 (3H, t, *J* = 8 Hz, Ar–H), 7.11–7.16 (6H, m, Ar–H), 4.93–5.03 (1H, m, CH), 4.75 (1H, brs, CH), 4.02–4.35 (2H, m, CH_2_), 3.40–3.58 (2H, m, CH_2_), 2.41–2.43 (2H, m, CH_2_), 2.27–2.35 (4H, m, CH_2_), 1.87–1.90 (2H, m, CH_2_), 1.76 (2H, brs, CH_2_), 1.23 (3H, s, CH_3_); ^13^C NMR (DMSO-D_6_, 100 MHz): *δ* ppm 158.7, 157.6, 156.8, 156.1, 154.4, 138.0, 130.6, 128.5, 128.0, 124.2, 119.4, 56.9, 52.3, 50.1, 30.1, 29.4, 29.0, 22.7 & 21.3; IR (KBr disc, cm^−1^): 3480 s, 3304 s (N–H), 2922 s (sp^2^ stretch), 2855 s (sp^3^ stretch), 1623 s (C=O stretch), 1568 s (N–H bend), 1521 m, 1480 s, 1438 w (C–H bend), 1222 s (C–N stretch), 1166 w (C–O stretch), 859 w (N–H oop); HRMS: calcd for [C_35_H_35_N_7_O_3_]^+^ 601.7110, found 602.2846 [M + 1]^+^, 624.2673 [M + 1 + Na]^+^ and 626.2737 [M + 3 + Na]^+^.

##### 4-Amino-1-((3*R*)-1-((5-chlorothiophene-2-carbonyl)prolyl)piperidin-3-yl)-3-(4-phenoxyphenyl)-1*H*-pyrazolo[3,4-*d*]pyrimidine (12p)


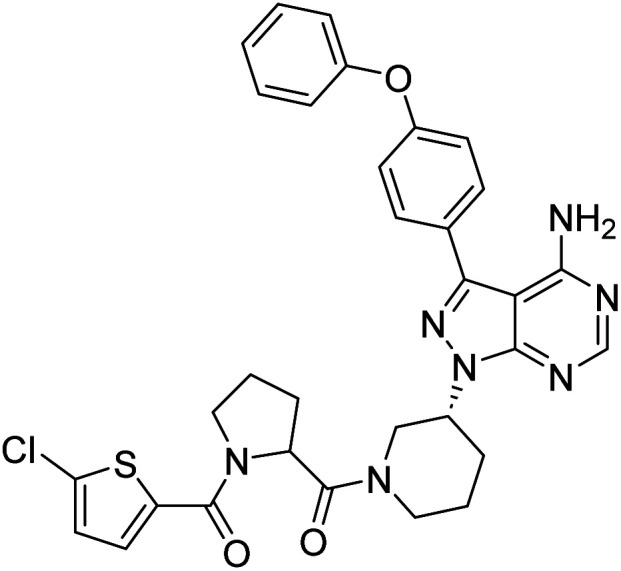
Cream white powder; yield: 307.7 mg (79%); M.P.: 147 °C; ^1^H NMR (DMSO-D_6_, 400 MHz): *δ* ppm 8.24–8.28 (1H, m, Ar–H), 7.67 (2H, brs, Ar–H), 7.54 (1H, brs, Ar–H), 7.44 (2H, brs, Ar–H), 7.15 (6H, brs, Ar–H), 4.94–5.05 (1H, m, CH), 4.70 (1H, brs, CH), 4.31 (2H, brs, CH_2_), 3.82–3.98 (2H, m, CH_2_), 3.55 (2H, brs, CH_2_), 2.15–2.27(2H, m, CH_2_), 1.87–1.97 (4H, m, CH_2_), 1.56 (2H, brs, CH_2_), 1.23 (2H, brs, CH_2_), 0.84–0.94 (2H, m, CH_2_); ^13^C NMR (DMSO-D_6_, 100 MHz): *δ* ppm 170.0, 169.6, 159.0, 157.5, 156.8, 156.1, 154.3, 139.5, 130.6, 128.5, 124.2, 119.5, 58.6, 52.2, 49.3, 31.4, 30.0, 28.4, 25.4, 22.5 & 14.4; IR (KBr disc, cm^−1^): 3468 s, 3309 s (N–H), 2948 s (sp^2^ stretch), 2868 s (sp^3^ stretch), 1613 s (C=O stretch), 1588 s (N–H bend), 1521 m, 1489 s, 1437 w (C–H bend), 1231 s (C–N stretch), 1166 w (C–O stretch), 846 w (N–H oop); HRMS: calcd for [C_32_H_30_ClN_7_O_3_S]^+^ 628.1480, found 628.1876 [M]^+^, 629.1919 [M + 1]^+^ and 630.1845 [M + 2]^+^.

##### 4-Amino-1-((3*R*)-1-((4-methoxybenzoyl)prolyl)piperidin-3-yl)-3-(4-phenoxyphenyl)-1*H*-pyrazolo[3,4-*d*]pyrimidine (12q)


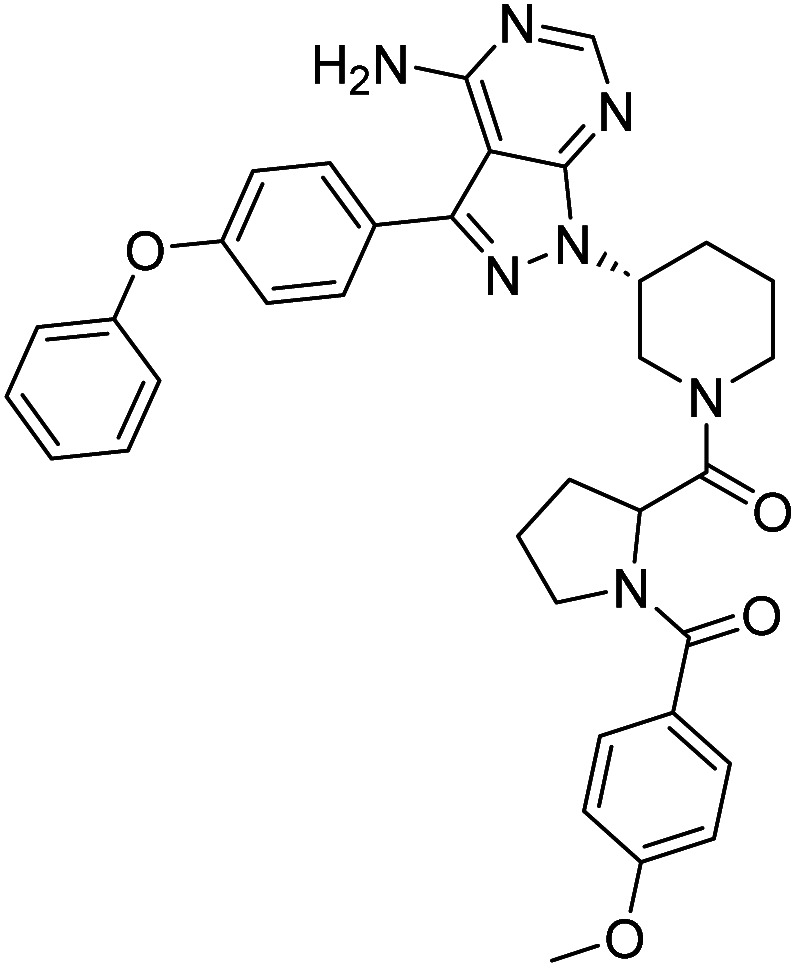
White powder; yield: 307.7 mg (83%); M.P.: 153 °C; ^1^H NMR (DMSO-D_6_, 400 MHz): *δ* ppm 8.23–8.34 (1H, m, Ar–H), 7.67–7.68 (2H, m, Ar–H), 7.42–7.55 (4H, m, Ar–H), 7.12–7.17 (6H, m, Ar–H), 6.98–7.00 (1H, t, *J* = 8 Hz, Ar–H), 4.92–5.02 (1H, m, CH), 4.72–4.77 (1H, t, *J* = 8 Hz, CH), 4.35–4.47 (2H, m, CH_2_), 3.81 (3H, s, OCH_3_), 3.54 (2H, brs, CH_2_), 3.29–3.32 (2H, m, CH_2_), 2.26 (2H, brs, CH_2_), 2.05–2.16 (2H, m, CH_2_), 1.89–1.91 (2H, m, CH_2_), 1.77 (2H, brs, CH_2_); ^13^C NMR (DMSO-D_6_, 100 MHz): *δ* ppm 160.9, 158.7, 157.6, 156.8, 156.1, 154.4, 130.6, 129.6, 129.2, 128.8, 128.4, 124.2, 119.5, 113.9, 57.1, 55.7, 50.3, 46.5, 30.1, 29.4, 29.0 & 25.6; IR (KBr disc, cm^−1^): 3466 s, 3397 s (N–H), 2925 s (sp^2^ stretch), 2855 s (sp^3^ stretch), 1623 s (C=O stretch), 1587 s (N–H bend), 1523 m, 1489 s, 1425 w (C–H bend), 1235 s (C–N stretch), 1167 w (C–O stretch), 871 w (N–H oop); HRMS: calcd for [C_35_H_35_N_7_O_4_]^+^ 617.7100, found 618.2807 [M]^+^, 619.2835 [M + 1]^+^ and 620.2804 [M + 2]^+^.

##### 7-Amino-3-((3*R*)-1-((3-chlorobenzoyl)prolyl)piperidin-3-yl)-1-(4-phenoxyphenyl)-1*H*-pyrazolo[4,3-*d*]pyrimidine (12r)


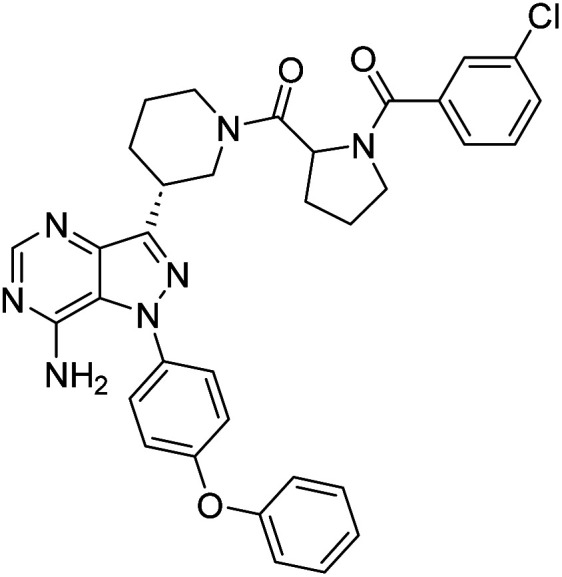
White powder; yield: 327.8 mg (85%); M.P.: 152 °C; ^1^H NMR (DMSO-D_6_, 400 MHz): *δ* ppm 8.24–8.31 (1H, m, Ar–H), 7.63–7.70 (2H, m, Ar–H), 7.54–7.58 (1H, m, Ar–H), 7.49–7.51 (1H, m, Ar–H), 7.40–7.46 (3H, m, Ar–H), 7.33 (1H, brs, Ar–H), 7.11–7.21 (5H, m, Ar–H), 4.91–5.03 (1H, m, CH), 4.71–4.83 (1H, m, CH), 4.01–4.35 (2H, m, CH_2_), 3.56–3.63 (2H, m, CH_2_), 3.28–3.30 (2H, m, CH_2_), 2.11–2.33 (2H, m, CH_2_), 1.91–2.02 (4H, m, CH_2_), 1.73–1.80 (2H, m, CH_2_); ^13^C NMR (DMSO-D_6_, 100 MHz): *δ* ppm 172.5, 170.5, 168.0, 166.6, 158.7, 157.5, 156.2, 143.7, 140.1, 133.5, 130.6, 129.8, 127.2, 126.1, 124.2, 119.4, 97.8, 59.6, 57.2, 52.2, 31.4, 30.5, 25.4, 22.5, 21.5 & 14.5; IR (KBr disc, cm^−1^): 3481 s, 3196 s (N–H), 2924 s (sp^2^ stretch), 2857 s (sp^3^ stretch), 1625 s (C=O stretch), 1567 s (N–H bend), 1480 s, 1439 w (C–H bend), 1221 s (C–N stretch), 1141 w (C–O stretch), 859 w (N–H oop), 762 w (C–Cl stretch); HRMS: calcd for [C_34_H_32_ClN_7_O_3_]^+^ 622.1260, found 622.2317 [M]^+^, 623.2348 [M + 1]^+^ and 624.2323 [M + 2]^+^, 644.2138 [M + Na]^+^, 644.2125 [M + Na + 1]^+^, 648.2165 [M + Na + 2]^+^.

##### 7-Amino-3-((3*R*)-1-((4-nitrobenzoyl)prolyl)piperidin-3-yl)-1-(4-phenoxyphenyl)-1*H*-pyrazolo[4,3-*d*]pyrimidine (12s)


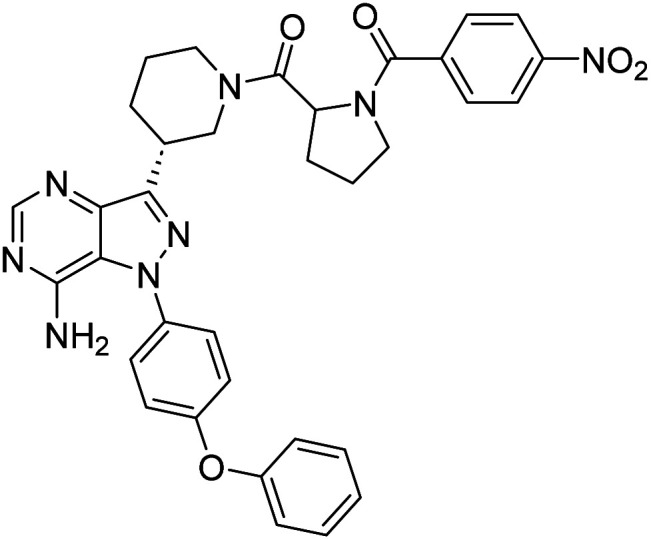
Yellowish-white powder; yield: 337.3 mg (86%); M.P.: 155 °C; ^1^H NMR (DMSO-D_6_, 400 MHz): *δ* ppm 8.42–8.44 (1H, d, *J* = 8 Hz, Ar–H), 8.29–8.37 (1H, m, Ar–H), 7.78–7.84 (1H, m, Ar–H), 7.67–7.73 (3H, m, Ar–H), 7.46–7.48 (2H, m, Ar–H), 7.16–7.23 (5H, m, Ar–H), 4.97–5.11 (1H, m, CH), 4.79–4.81 (1H, m, CH), 3.91–4.52 (2H, m, CH_2_), 3.66–3.68 (2H, m, CH_2_), 3.46–3.48 (2H, m, CH_2_), 2.21–2.38 (2H, m, CH_2_), 2.05–2.07 (2H, m, CH_2_), 1.80–1.86 (2H, m, CH_2_); ^13^C NMR (DMSO-D_6_, 100 MHz): *δ* ppm 170.0, 168.1, 166.4, 158.7, 157.5, 156.8, 156.1, 154.3, 148.3, 143.8, 130.6, 128.9, 128.3, 124.1, 119.5, 97.7, 59.3, 52.2, 36.2, 31.3, 29.5, 25.3, 23.0, 21.6 & 14.4; IR (KBr disc, cm^−1^): 3468 s, 3317 s (N–H), 2925 s (sp^2^ stretch), 2856 s (sp^3^ stretch), 1631 s (C=O stretch), 1596 s (N–H bend), 1522 m, 1488 s, 1436 w (C–H bend), 1346 s (N–O stretch), 1235 s (C–N stretch), 1103 w (C–O stretch), 867 w (N–H oop); HRMS: calcd for [C_34_H_32_N_8_O_5_]^+^ 632.6810, found 633.1482 [M]^+^, 634.1482 [M + 1]^+^ and 635.1465 [M + 2]^+^.

##### ((*R*)-3-(7-Amino-1-(4-phenoxyphenyl)-1*H*-pyrazolo[4,3-*d*]pyrimidin-3-yl)piperidin-1-yl)(1-(5-((6-chloro-2-methylpyrimidin-4-yl)amino)thiazole-2-carbonyl)pyrrolidin-2-yl)methanone (12t)


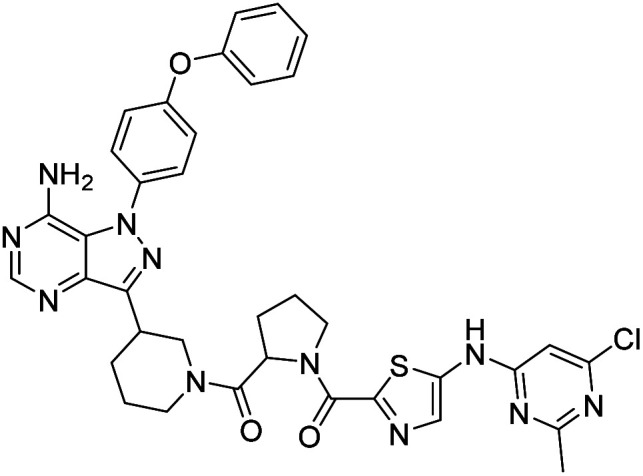
White powder; yield: 419.96 mg (92%); M.P.: 278 °C; ^1^H NMR (DMSO-D_6_): *δ* ppm ^1^H NMR (DMSO-D_6_, 400 MHz): *δ* ppm 8.24–8.29 (1H, m, Ar–H), 7.98–8.01 (1H, d, *J* = 12 Hz, Ar–H), 7.60–7.68 (2H, m, Ar–H), 7.42–7.46 (2H, t, *J* = 4 Hz, Ar–H), 7.15–7.21 (5H, m, Ar–H), 6.94 (1H, brs, Ar–H), 5.06 (1H, m, CH), 4.71–4.72 (1H, m, CH), 4.33–4.39 (2H, m, CH_2_), 3.84–3.86 (2H, m, CH_2_), 3.57–3.63 (2H, m, CH_2_), 2.60 (3H, s, CH_3_), 2.18–2.30 (4H, m, CH_2_), 1.81–1.98 (4H, m, CH_2_); ^13^C NMR (DMSO-D_6_, 100 MHz): *δ* ppm 170.3, 167.9, 161.3, 160.1, 158.7, 157.5, 156.8, 156.1, 154.3, 143.7, 141.0, 130.6, 128.9, 124.2, 119.5, 103.9, 97.7, 58.3, 52.3, 49.1, 46.5, 45.7, 30.1, 28.5, 26.8 & 25.7; IR (KBr disc, cm^−1^): 3470 s (N–H), 2924 s (sp^2^ stretch), 2851 s (sp^3^ stretch), 1574 s (C=O stretch, N–H bend), 1523 s, 1489 s, 1408 w (C–H bend), 1238 s (C–N stretch), 1129 w (C–O stretch), 850 w (N–H oop); ESI-MS: calcd for [C_36_H_34_ClN_11_O_3_S]^+^ 736.2520, found 736.23 [M]^+^, and 737.22 [M + 1]^+^.

### Biological studies

Based on their structural novelty, all synthesized compounds were tested for a preliminary single-dose screening under the Developmental Therapeutics Program by the National Cancer Institute, USA. A one-dose screening was performed at a single high dose of 10 μM for all synthesized compounds across all cell lines in the NCI60 panel consisting of 60 human tumour cell lines from nine tissue origins: leukaemia, breast, lung, prostate, renal, ovarian, melanoma, central nervous system, and colon. Following this, only the compounds that exhibited exceptional activity in a maximum number of tumour cell lines were taken up for a dose-dependent experiment to understand the anti-cancer efficacy of the compounds.

Sulforhodamine B (SRB) colorimetric assay was performed as per the literature,^33^ in both single and five-dose assays, to evaluate the *in vitro* effectiveness of the compounds. Shortly, the tumour cell lines were cultures in RPMI 1640 medium containing 5% fetal bovine serum and 2 mM l-glutamine. The plating densities for the cells in 96 well plates ranged from 5000 to 40 000 cells per well depending on the doubling time of individual cell lines. The well plates were incubated at 37 °C, 5% CO_2_, 95% air and 100% relative humidity for 24 h before adding experimental drugs. The compounds were solubilized in DMSO. For the five-dose experiment, the compounds were diluted by ½ log serial dilutions to provide five different concentrations (*i.e.*, −8 to −4 log concentrations or 100 to 0.01 μM) along with control. The plates were incubated for 48 hours before the addition of SRB solution at 0.4% (w/v) in 1% acetic acid (100 μL per well), incubated for another 10 minutes before the bound stain was stained with 10 mM Trizma base, and the absorbance is read on an automated plate reader at 515 nm. The half maximal effective concentration (IC_50_) was calculated from [(*T*_i_ − *T*_z_)/(*C* − *T*_z_)] × 100 = 50, which is the drug concentration resulting in a 50% reduction in the net protein increase (as measured by SRB staining) in control cells during the drug incubation, where *T*_i_ = test growth in presence of the drug agent, *T*_z_ = absorbance at time zero, and *C* = control growth. All experiments were conducted in triplicates and the man values were represented in this paper. A detailed description of the screening process and methodology is available at the DTP website (https://dtp.cancer.gov/discovery_development/nci-60/methodology.htm).

## Conclusions

In this study, we successfully synthesized and characterized a novel library of 20 compounds featuring a 4-aminopyrazolo[3,4-*d*]pyrimidine core using advanced spectroscopic techniques. These compounds were evaluated for their growth inhibitory activities against 60 human tumor cell lines across nine cancer panels, demonstrating significant anticancer activity, particularly in leukemia, melanoma, and renal cancer cell lines. Compounds 11, 12c, 12d, 12f, and 12j especially stood out with superior activity compared to established small molecule drugs. Compound 12c demonstrated 2.88 times greater activity than sorafenib and 1.45 times greater than sunitinib when tested against the UO-31 renal cancer cell line. Structure–activity relationship analysis provided insights into the critical structural features influencing biological activity, and several compounds exhibited *in vitro* activity comparable to or exceeding that of known small molecule therapies. These findings underscore the potential behavior of 4-aminopyrazolo[3,4-*d*]pyrimidine derivatives as promising candidates as anti-cancer molecules for further development, warranting additional preclinical studies to fully elucidate their therapeutic potential and mechanisms of action.

## Data availability

All the analyzed data during this work was included in the published article.

## Author contributions

AD and GV conceived and designed the analysis. MBH and MSA drafted the manuscript, and PKG and MN analyzed the data and edited the manuscript. All authors read and approved the final manuscript.

## Conflicts of interest

There are no conflicts to declare.

## Supplementary Material

RA-014-D4RA05136J-s001
