# Supplementary material for: Design, synthesis, and high-throughput in vitro anti-cancer evaluation of novel 4-aminopyrazolo[3,4-d]pyrimidine derivatives: potential anti-cancer candidates against UO-31 renal cancer cells
Source: RSC Adv. 2024 Sep 27;14(42):30938–53. doi: 10.1039/d4ra05136j (PMC11428193; doi:10.1039/d4ra05136j)
Supplement: RA-014-D4RA05136J-s001 [file RA-014-D4RA05136J-s001.pdf]

Supplementary Information (SI) for RSC Advances.
This journal is © The Royal Society of Chemistry 2024

Novel 4-Aminopyrazolo[3,4-d]Pyrimidine Derivatives: Design, Synthesis And High-Throughput In-Vitro Anti-Cancer Evaluation

Amitananda Dash,[a] Guruswamy Vaddamanu, [b] Mohammed Bakr Hawsawi,[c] Mustafa S. Alluhaibi, [c] Pavana Kumari G., [a] * Naveen Mulakayala [b] *

Compound code	IUPAC name	Data type	Figure Notation
**12a**	2,2,2-trifluoro-N-(3-(4-phenoxyphenyl)-1-((3R)-1-(1-(2,2,2-trifluoroacetyl)pyrrolidine-2-carbonyl)piperidin-3-yl)-1H-pyrazolo[3,4-d]pyrimidin-4-yl)acetamide	HRMS	Figure 1
		^{1}H NMR full spectrum	Figure 2
		^{1}H NMR aromatic region	Figure 3
		^{1}H NMR aliphatic region	Figure 4
		^{13}C NMR	Figure 5
		FT-IR	Figure 6
**12b**	4-amino-1-((3R)-1-((4-chloro-2-fluorobenzoyl)prolyl)piperidin-3-yl)-3-(4-phenoxyphenyl)-1H-pyrazolo[3,4-d]pyrimidine	HRMS	Figure 7
		^{1}H NMR full spectrum	Figure 8
		^{1}H NMR aromatic region	Figure 9
		^{1}H NMR aliphatic region	Figure 10
		^{13}C NMR	Figure 11
		FT-IR	Figure 12
**12c**	4-amino-1-((3R)-1-((4-fluorobenzoyl)prolyl)piperidin-3-yl)-3-(4-phenoxyphenyl)-1H-pyrazolo[3,4-d]pyrimidine	HRMS	Figure 13
		^{1}H NMR full spectrum	Figure 14
		^{1}H NMR aromatic region	Figure 15
		^{1}H NMR aliphatic region	Figure 16
		^{13}C NMR	Figure 17
		FT-IR	Figure 18
**12d**	4-amino-3-(4-phenoxyphenyl)-1-((3R)-1-((2,4,5-trifluorobenzoyl)prolyl)piperidin-3-yl)-1H-pyrazolo[3,4-d]pyrimidine	HRMS	Figure 19
		^{1}H NMR full spectrum	Figure 20
		^{1}H NMR aromatic region	Figure 21
		^{1}H NMR aliphatic region	Figure 22
		^{13}C NMR	Figure 23
		FT-IR	Figure 24
**12e**	4-amino-1-((3R)-1-((4-chloropicolinoyl)prolyl)piperidin-3-yl)-3-(4-phenoxyphenyl)-1H-pyrazolo[3,4-d]pyrimidine	HRMS	Figure 25
		^{1}H NMR full spectrum	Figure 26
		^{1}H NMR aromatic region	Figure 27
		^{1}H NMR aliphatic region	Figure 28
		^{13}C NMR	Figure 29
		FT-IR	Figure 30
**12f**	4-amino-1-((3R)-1-((2,4-difluorobenzoyl)prolyl)piperidin-3-yl)-3-(4-phenoxyphenyl)-1H-pyrazolo[3,4-d]pyrimidine	HRMS	Figure 31
		^{1}H NMR full spectrum	Figure 32
		^{1}H NMR aromatic region	Figure 33
		^{1}H NMR aliphatic region	Figure 34
		^{13}C NMR	Figure 35
		FT-IR	Figure 36
**12g**	4-amino-1-((3R)-1-(benzoylprolyl)piperidin-3-yl)-3-(4-phenoxyphenyl)-1H-pyrazolo[3,4-d]pyrimidine	HRMS	Figure 37
		^{1}H NMR full spectrum	Figure 38
		^{1}H NMR aromatic region	Figure 39
		^{1}H NMR aliphatic region	Figure 40
		^{13}C NMR	Figure 41
		FT-IR	Figure 42
**12h**	4-amino-3-(4-phenoxyphenyl)-1-((3R)-1-(picolinoylprolyl)piperidin-3-yl)-1H-pyrazolo[3,4-d]pyrimidine	HRMS	Figure 43
		^{1}H NMR full spectrum	Figure 44
		^{1}H NMR aromatic region	Figure 45
		^{1}H NMR aliphatic region	Figure 46
		^{13}C NMR	Figure 47
		FT-IR	Figure 48

**12i**	4-amino-1-((3R)-1-((3-methyl-4-nitrobenzoyl)prolyl)piperidin-3-yl)-3-(4-phenoxyphenyl)-1H-pyrazolo[3,4-d]pyrimidine	HRMS	Figure 49
		^{1}H NMR full spectrum	Figure 50
		^{1}H NMR aromatic region	Figure 51
		^{1}H NMR aliphatic region	Figure 52
		^{13}C NMR	Figure 53
		FT-IR	Figure 54
**12j**	4-amino-1-((3R)-1-((4-chlorobenzoyl)prolyl)piperidin-3-yl)-3-(4-phenoxyphenyl)-1H-pyrazolo[3,4-d]pyrimidine	HRMS	Figure 55
		^{1}H NMR full spectrum	Figure 56
		^{1}H NMR aromatic region	Figure 57
		^{1}H NMR aliphatic region	Figure 58
		^{13}C NMR	Figure 59
		FT-IR	Figure 60
**12k**	4-amino-1-((3R)-1-((3-nitrobenzoyl)prolyl)piperidin-3-yl)-3-(4-phenoxyphenyl)-1H-pyrazolo[3,4-d]pyrimidine	HRMS	Figure 61
		^{1}H NMR full spectrum	Figure 62
		^{1}H NMR aromatic region	Figure 63
		^{1}H NMR aliphatic region	Figure 64
		^{13}C NMR	Figure 65
		FT-IR	Figure 66
**12l**	4-(2-((R)-3-(4-amino-3-(4-phenoxyphenyl)-1H-pyrazolo[3,4-d]pyrimidin-1-yl)piperidine-1-carbonyl)pyrrolidine-1-carbonyl)phenyl acetate	HRMS	Figure 67
		^{1}H NMR full spectrum	Figure 68
		^{1}H NMR aromatic region	Figure 69
		^{1}H NMR aliphatic region	Figure 70
		^{13}C NMR	Figure 71
		FT-IR	Figure 72
**12m**	4-amino-1-((3R)-1-((4-bromo-3-(trifluoromethyl)benzoyl)prolyl)piperidin-3-yl)-3-(4-phenoxyphenyl)-1H-pyrazolo[3,4-d]pyrimidine	HRMS	Figure 73
		^{1}H NMR full spectrum	Figure 74
		^{1}H NMR aromatic region	Figure 75
		^{1}H NMR aliphatic region	Figure 76
		^{13}C NMR	Figure 77
		FT-IR	Figure 78
**12n**	1-(2-((R)-3-(4-amino-3-(4-phenoxyphenyl)-1H-pyrazolo[3,4-d]pyrimidin-1-yl)piperidine-1-carbonyl)pyrrolidin-1-yl)-2-(4-fluorophenyl)ethanone	ESI-MS	Figure 79
		^{1}H NMR full spectrum	Figure 80
		^{1}H NMR aromatic region	Figure 81
		^{1}H NMR aliphatic region	Figure 82
		^{13}C NMR	Figure 83
		FT-IR	Figure 84
**12o**	4-amino-1-((3R)-1-((3-methylbenzoyl)prolyl)piperidin-3-yl)-3-(4-phenoxyphenyl)-1H-pyrazolo[3,4-d]pyrimidine	HRMS	Figure 85
		^{1}H NMR full spectrum	Figure 86
		^{1}H NMR aromatic region	Figure 87
		^{1}H NMR aliphatic region	Figure 88
		^{13}C NMR	Figure 89
		FT-IR	Figure 90
**12p**	4-amino-1-((3R)-1-((5-chlorothiophene-2-carbonyl)prolyl)piperidin-3-yl)-3-(4-phenoxyphenyl)-1H-pyrazolo[3,4-d]pyrimidine	HRMS	Figure 91
		^{1}H NMR full spectrum	Figure 92
		^{1}H NMR aromatic region	Figure 93
		^{1}H NMR aliphatic region	Figure 94
		^{13}C NMR	Figure 95
		FT-IR	Figure 96
**12q**	4-amino-1-((3R)-1-((4-methoxybenzoyl)prolyl)piperidin-3-yl)-3-(4-phenoxyphenyl)-1H-pyrazolo[3,4-d]pyrimidine	HRMS	Figure 97
		^{1}H NMR full spectrum	Figure 98
		^{1}H NMR aromatic region	Figure 99
		^{1}H NMR aliphatic region	Figure 100
		^{13}C NMR	Figure 101
		FT-IR	Figure 102
**12r**	7-amino-3-((3R)-1-((3-	HRMS	Figure 103

	chlorobenzoyl)prolyl)piperidin-3-yl)-1-(4-phenoxyphenyl)-1H-pyrazolo[4,3-d]pyrimidine	^{1}H NMR full spectrum	Figure 104
		^{1}H NMR aromatic region	Figure 105
		^{1}H NMR aliphatic region	Figure 106
		^{13}C NMR	Figure 107
		FT-IR	Figure 108
**12s**	7-amino-3-((3R)-1-((4-nitrobenzoyl)prolyl)piperidin-3-yl)-1-(4-phenoxyphenyl)-1H-pyrazolo[4,3-d]pyrimidine	HRMS	Figure 109
		^{1}H NMR full spectrum	Figure 110
		^{1}H NMR aromatic region	Figure 111
		^{1}H NMR aliphatic region	Figure 112
		^{13}C NMR	Figure 113
		FT-IR	Figure 114
**12t**	((R)-3-(7-amino-1-(4-phenoxyphenyl)-1H-pyrazolo[4,3-d]pyrimidin-3-yl)piperidin-1-yl)(1-(5-((6-chloro-2-methylpyrimidin-4-yl)amino)thiazole-2-carbonyl)pyrrolidin-2-yl)methanone	ESI-MS	Figure 115
		^{1}H NMR full spectrum	Figure 116
		^{1}H NMR aromatic region	Figure 117
		^{1}H NMR aliphatic region	Figure 118
		^{13}C NMR	Figure 119
		FT-IR	Figure 120
**11**	((S)-3-(4-amino-3-(3-phenoxyphenyl)-1H-pyrazolo[3,4-d]pyrimidin-1-yl)piperidin-1-yl)(pyrrolidin-3-yl)methanone	ESI-MS	Figure 121
		^{1}H NMR full spectrum	Figure 122
		^{1}H NMR aromatic region	Figure 123
		^{1}H NMR aliphatic region	Figure 124
		^{13}C NMR	Figure 125
		FT-IR	Figure 126
**10**	tert-butyl 3-((S)-3-(4-amino-3-(3-phenoxyphenyl)-1H-pyrazolo[3,4-d]pyrimidin-1-yl)piperidine-1-carbonyl) pyrrolidine-1-carboxylate	ESI-MS	Figure 127
		^{1}H NMR full spectrum	Figure 128
		^{1}H NMR aromatic region	Figure 129
		^{1}H NMR aliphatic region	Figure 130
		^{13}C NMR	Figure 131
		FT-IR	Figure 132
**8**	(S)-3-(3-phenoxyphenyl)-1-(piperidin-3-yl)-1H-pyrazolo[3,4-d]pyrimidin-4-amine	ESI-MS	Figure 133
		^{1}H NMR full spectrum	Figure 134
		^{1}H NMR aromatic region	Figure 135
		^{1}H NMR aliphatic region	Figure 136
		^{13}C NMR	Figure 137
		FT-IR	Figure 138
**12c**		*In-vitro* study data	Figure 139
	Dose-response curves in individual cell lines		Figure 140
	Dose-response curves in all cell lines		Figure 141
	In-vitro five dose testing results		Figure 142
	IC_{50} values depicted in the waterfall graph		Figure 143
**12d**	Single dose mean graph	*In-vitro* study data	Figure 144
	Dose-response curves in individual cell lines		Figure 145
	Dose-response curves in all cell lines		Figure 146
	In-vitro five dose testing results		Figure 147
	IC_{50} values depicted in the waterfall graph		Figure 148
**12f**	Single dose mean graph	*In-vitro* study data	Figure 149
	Dose-response curves in individual cell lines		Figure 150
	Dose-response curves in all cell lines		Figure 151
	In-vitro five dose testing results		Figure 152
	IC_{50} values depicted in the waterfall graph		Figure 153
**12j**	Single dose mean graph	*In-vitro* study data	Figure 154
	Dose-response curves in individual cell		Figure 155

	lines		
	Dose-response curves in all cell lines		Figure 156
	In-vitro five dose testing results		Figure 157
	IC_{50} values depicted in the waterfall graph		Figure 158
**11**	Single dose mean graph	*In-vitro* study data	Figure 159
	Dose-response curves in individual cell lines		Figure 160
	Dose-response curves in all cell lines		Figure 161
	In-vitro five dose testing results		Figure 162
	IC_{50} values depicted in the waterfall graph		Figure 163

Spectral data of ***2,2,2-trifluoro-N-(3-(4-phenoxyphenyl)-1-((3R)-1-(1-(2,2,2-trifluoroacetyl)pyrrolidine-2-carbonyl)piperidin-3-yl)-1H-pyrazolo[3,4-d]pyrimidin-4-yl)acetamide (12a)***

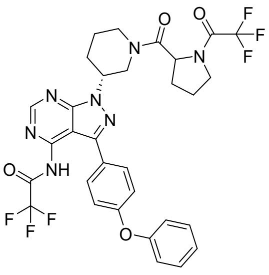

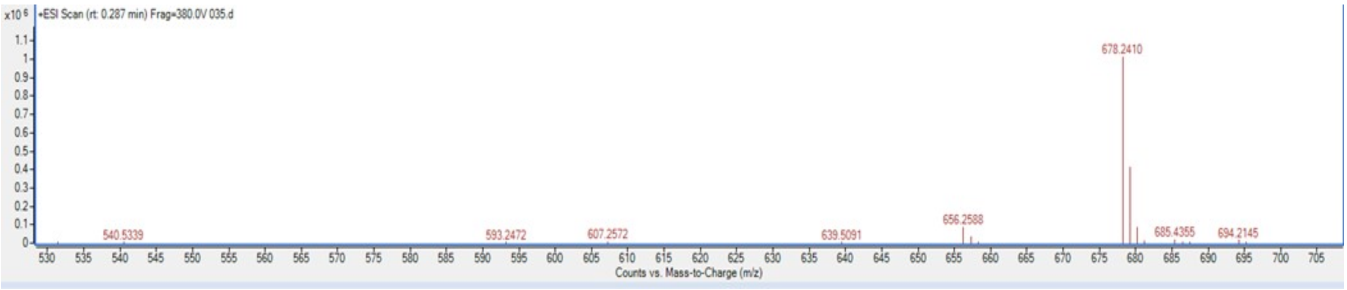

Figure 1. HRMS spectrum of **12a**

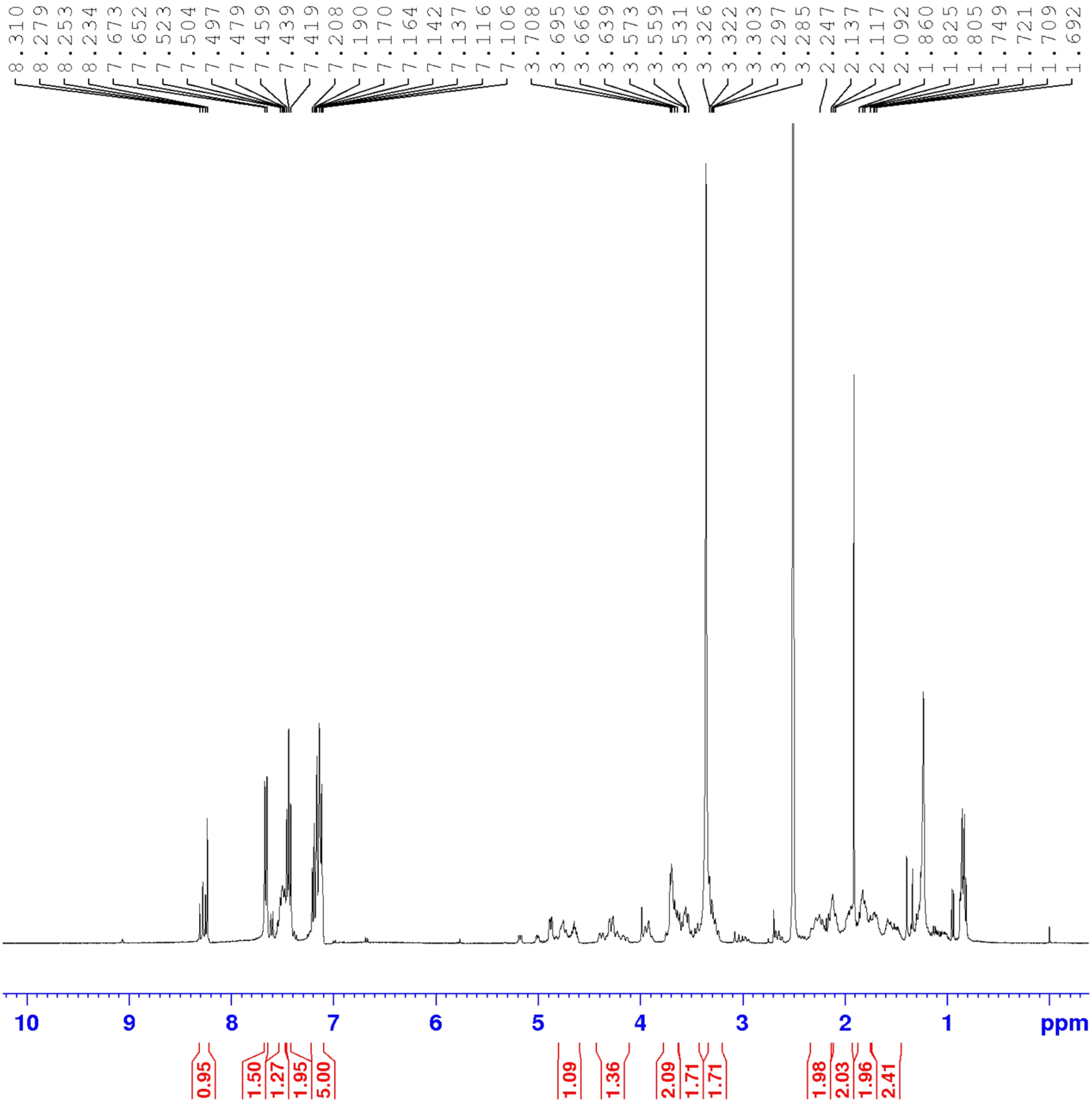

Figure 2. ^{1}H NMR full spectrum of **12a**

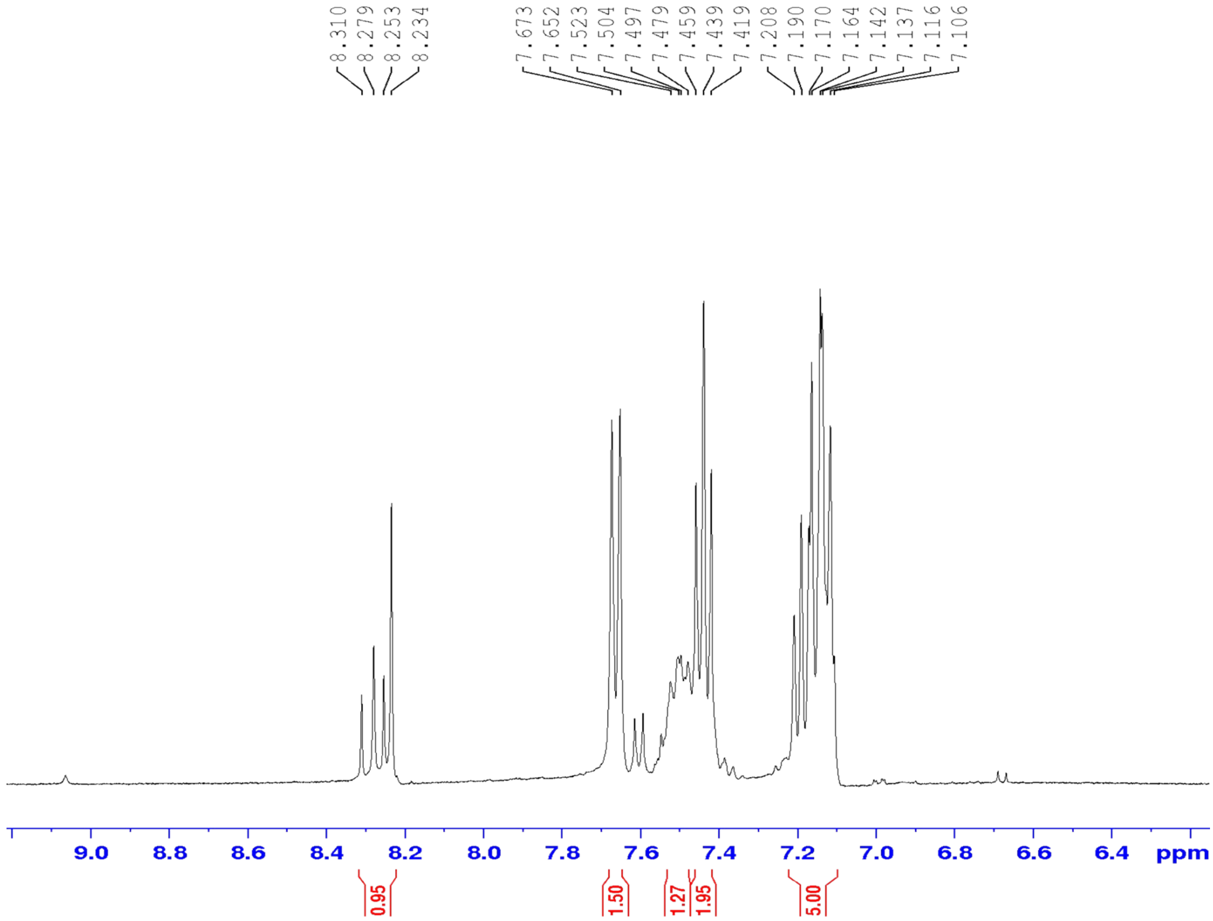

Figure 3. Aromatic region of the ^{1}H NMR spectrum for **12a**

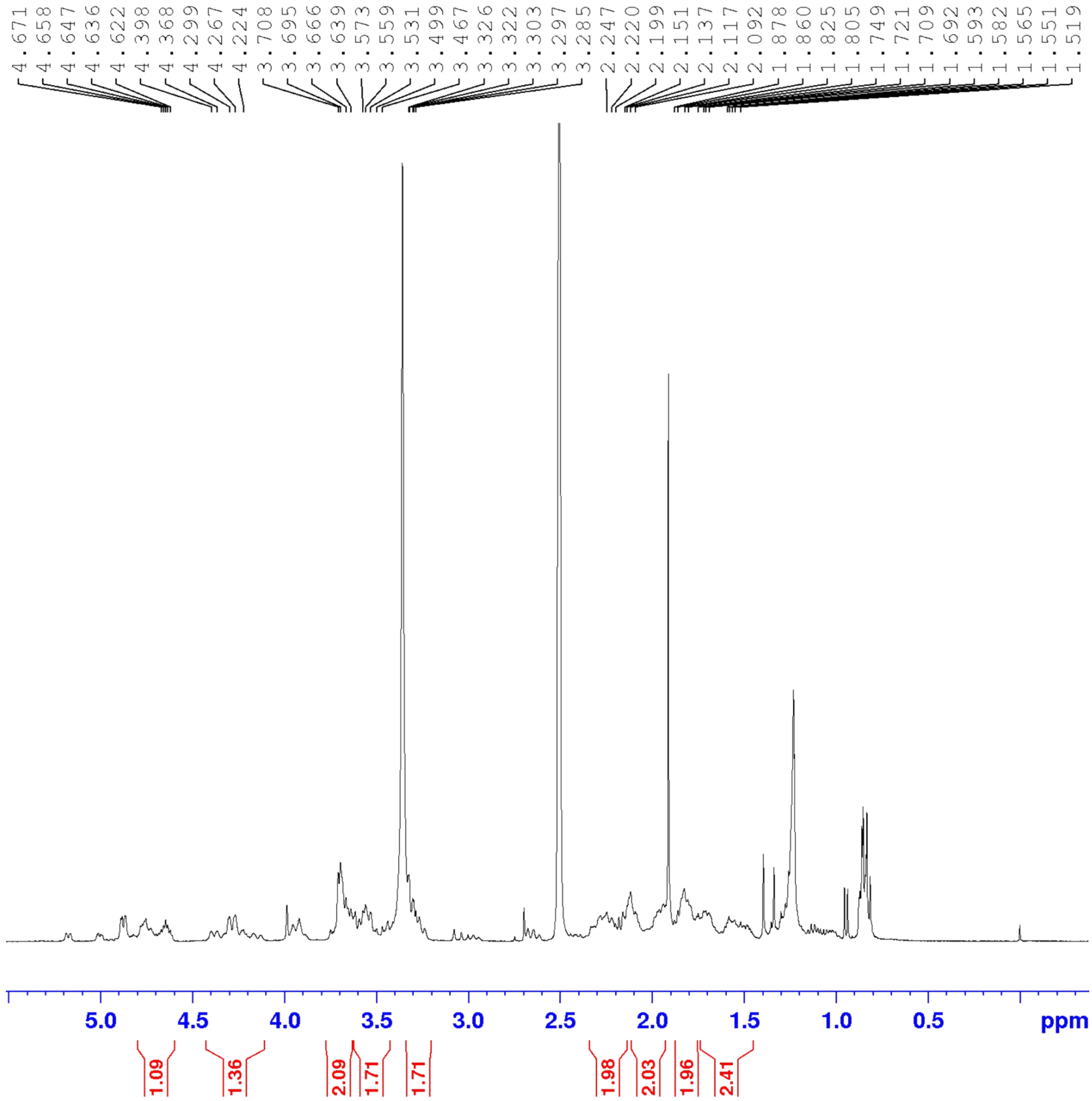

Figure 4. Aliphatic region of the ^{1}H NMR spectrum for **12a**

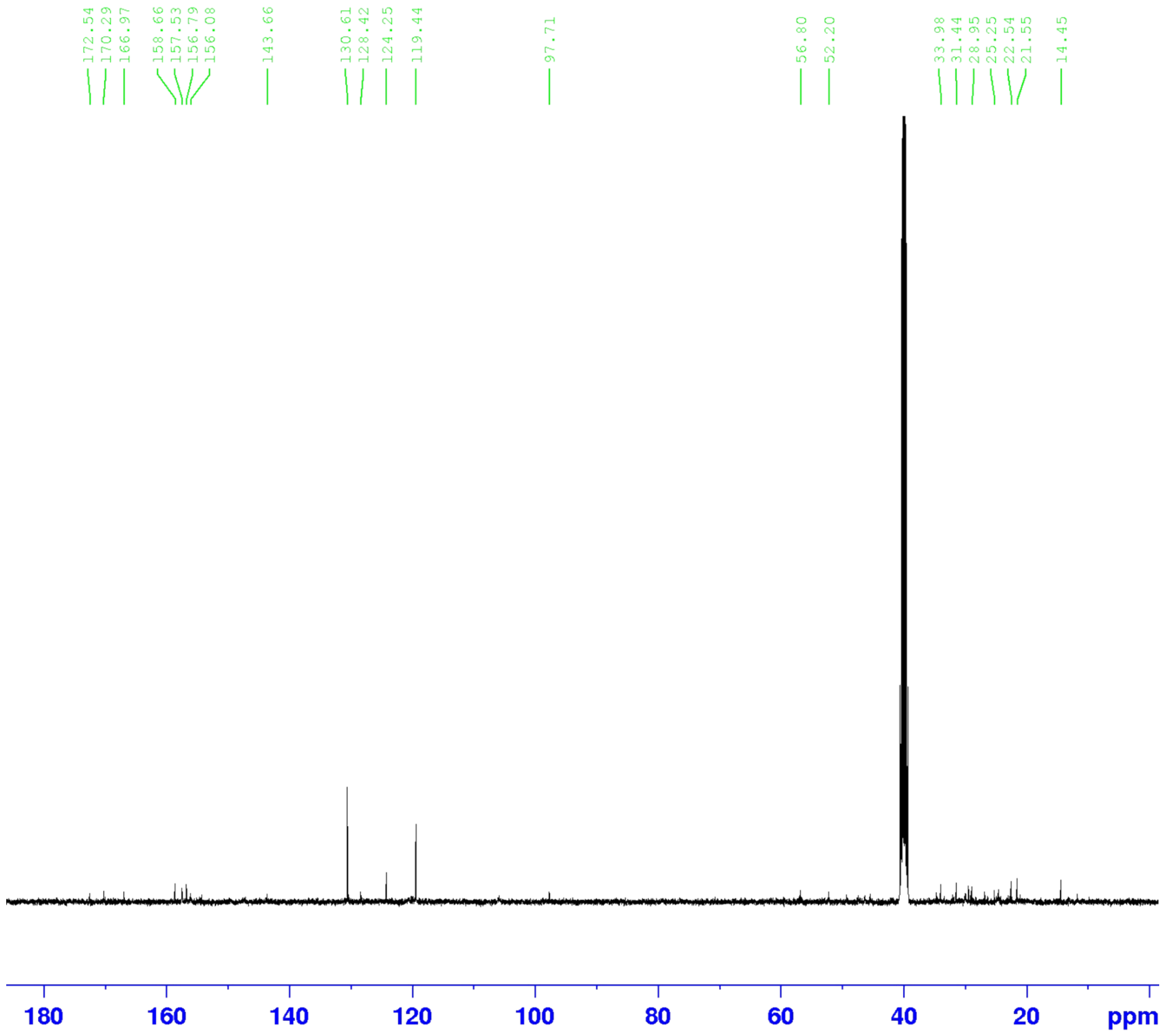

Figure 5. ^{13}C-NMR spectrum of **12a**

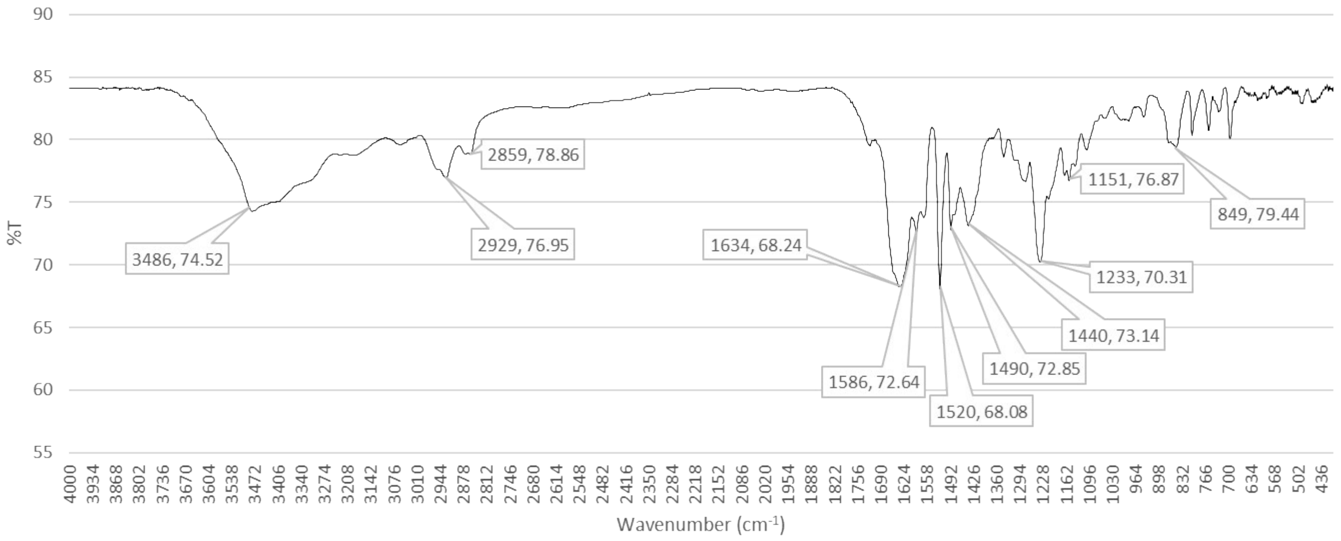

Figure 6. FT-IR spectrum of **12a**

Spectral data of ***4-amino-1-((3R)-1-((4-chloro-2-fluorobenzoyl)prolyl)piperidin-3-yl)-3-(4-phenoxyphenyl)-1H-pyrazolo[3,4-d]pyrimidine (12b)***

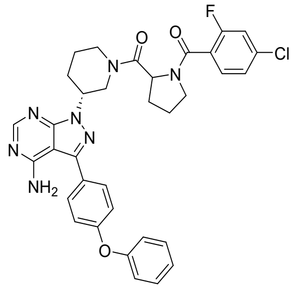

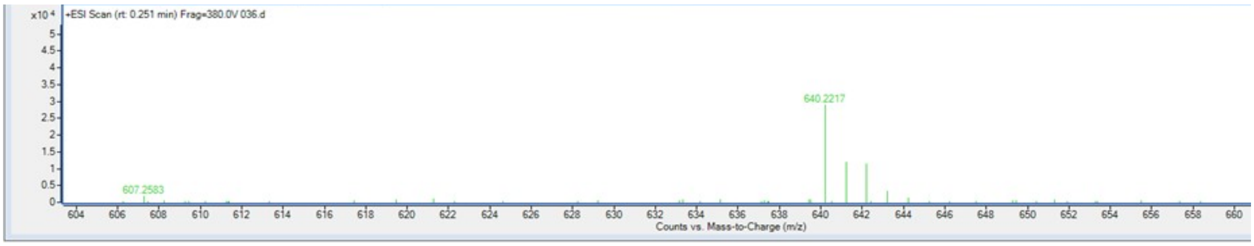

Figure 7. HRMS spectrum of **12b**

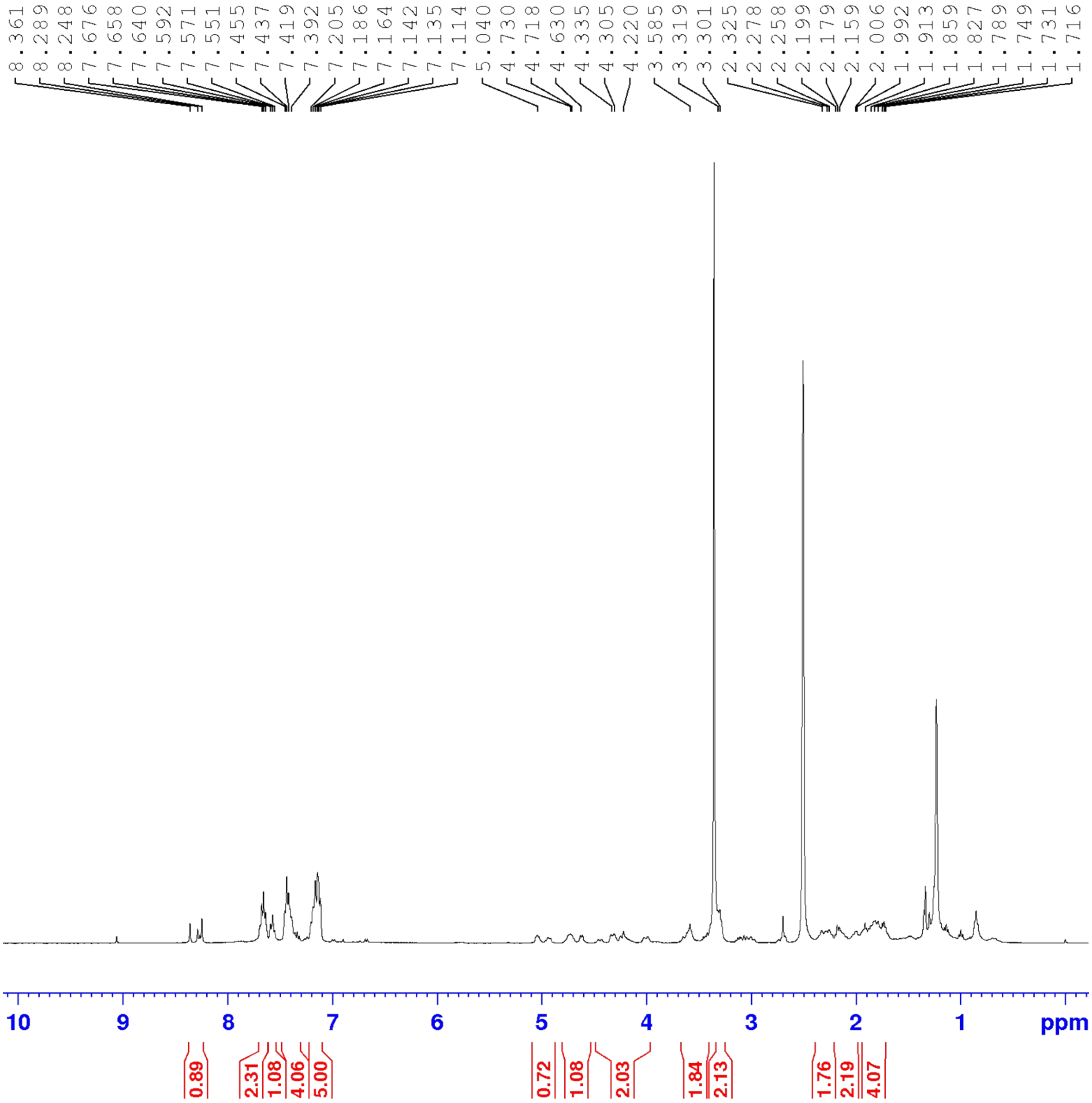

Figure 8. ^{1}H NMR full spectrum of **12b**

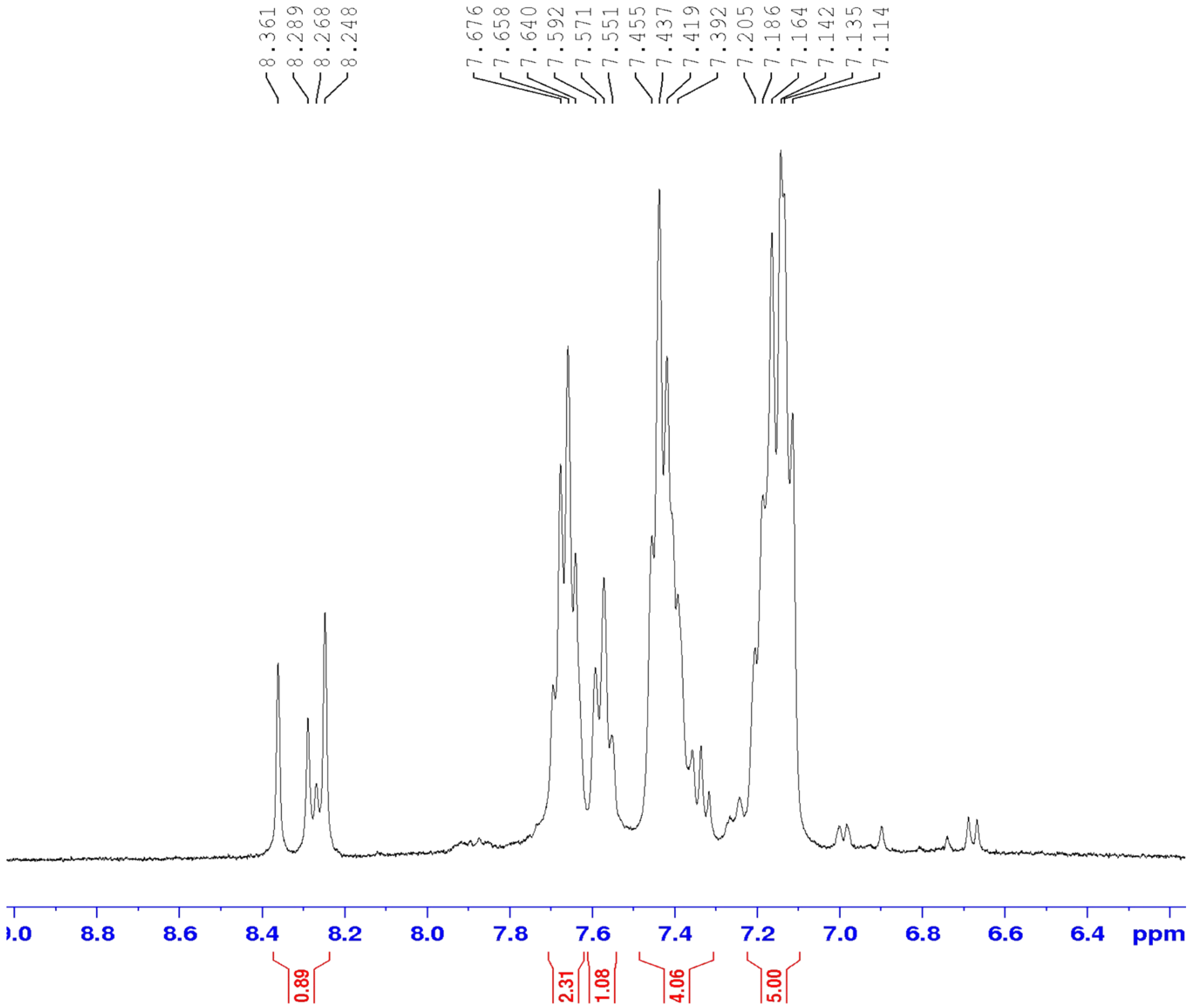

Figure 9. Aromatic region of the ^{1}H NMR spectrum for **12b**

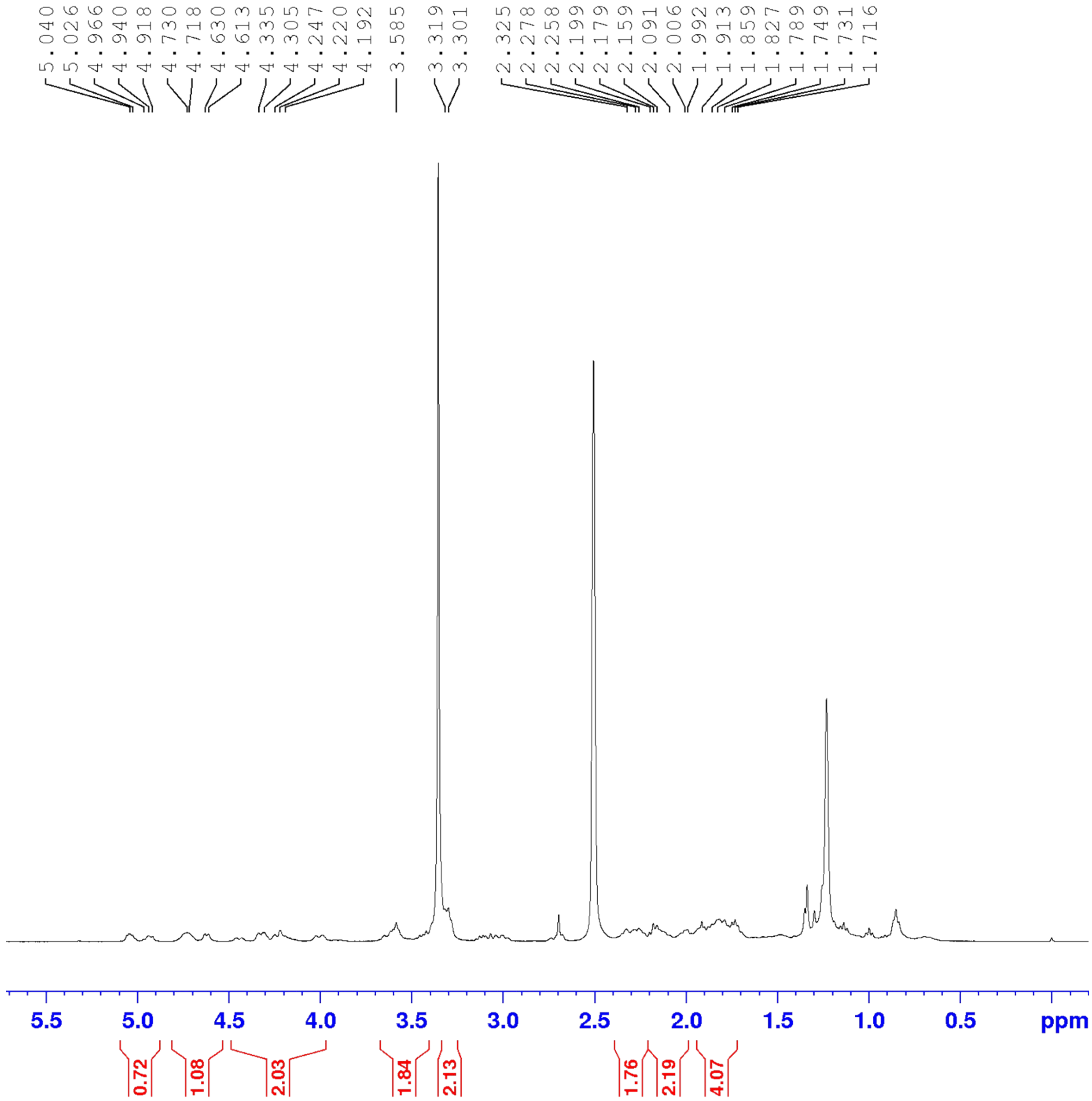

Figure 10. Aliphatic region of the ^{1}H NMR spectrum for **12b**

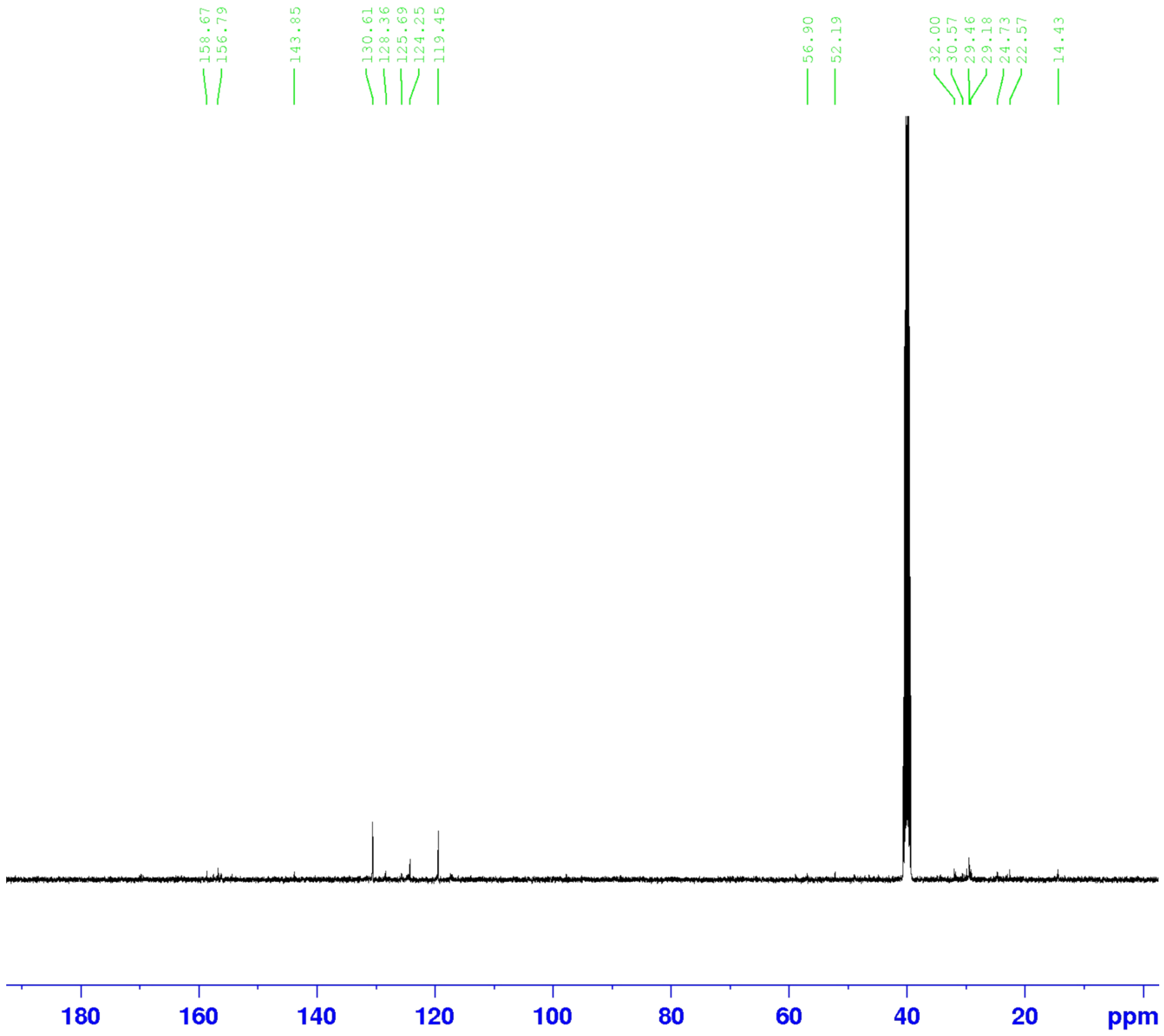

Figure 11. ^{13}C-NMR spectrum of **12b**

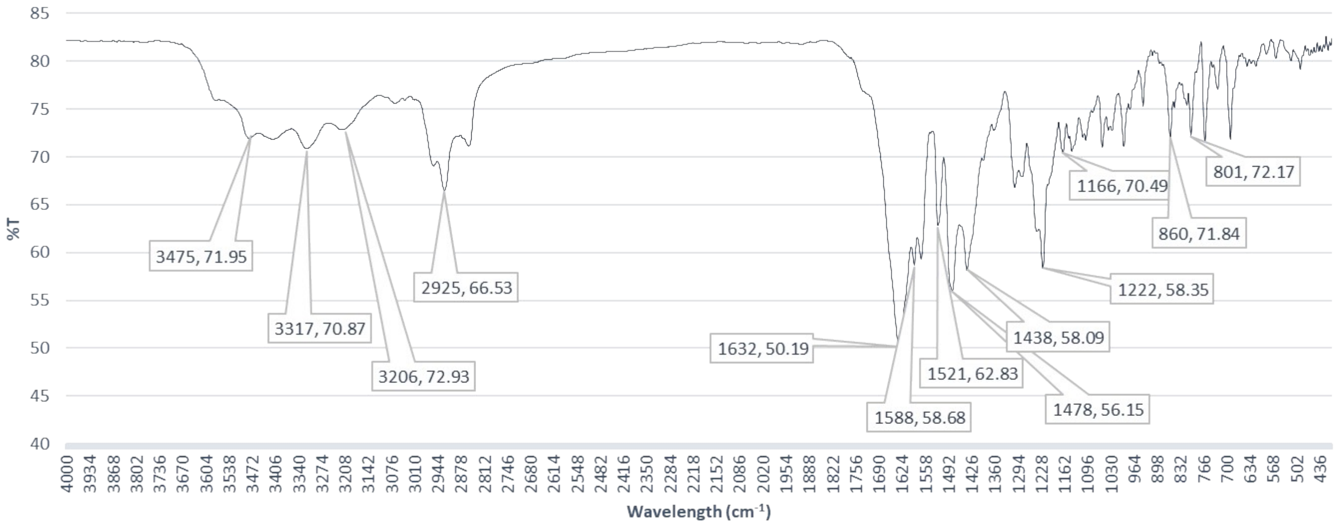

Figure 12. FT-IR spectrum of **12b**

Spectral data of ***4-amino-1-((3R)-1-((4-fluorobenzoyl)prolyl)piperidin-3-yl)-3-(4-phenoxyphenyl)-1H-pyrazolo[3,4-d]pyrimidine (12c)***

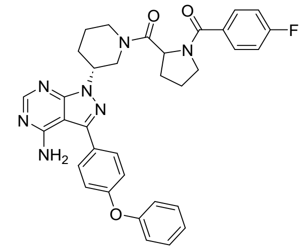

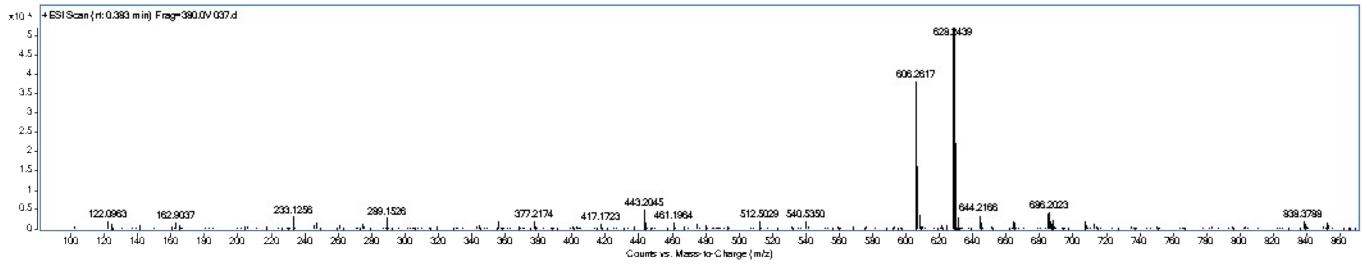

Figure 13. HRMS spectrum of **12c**

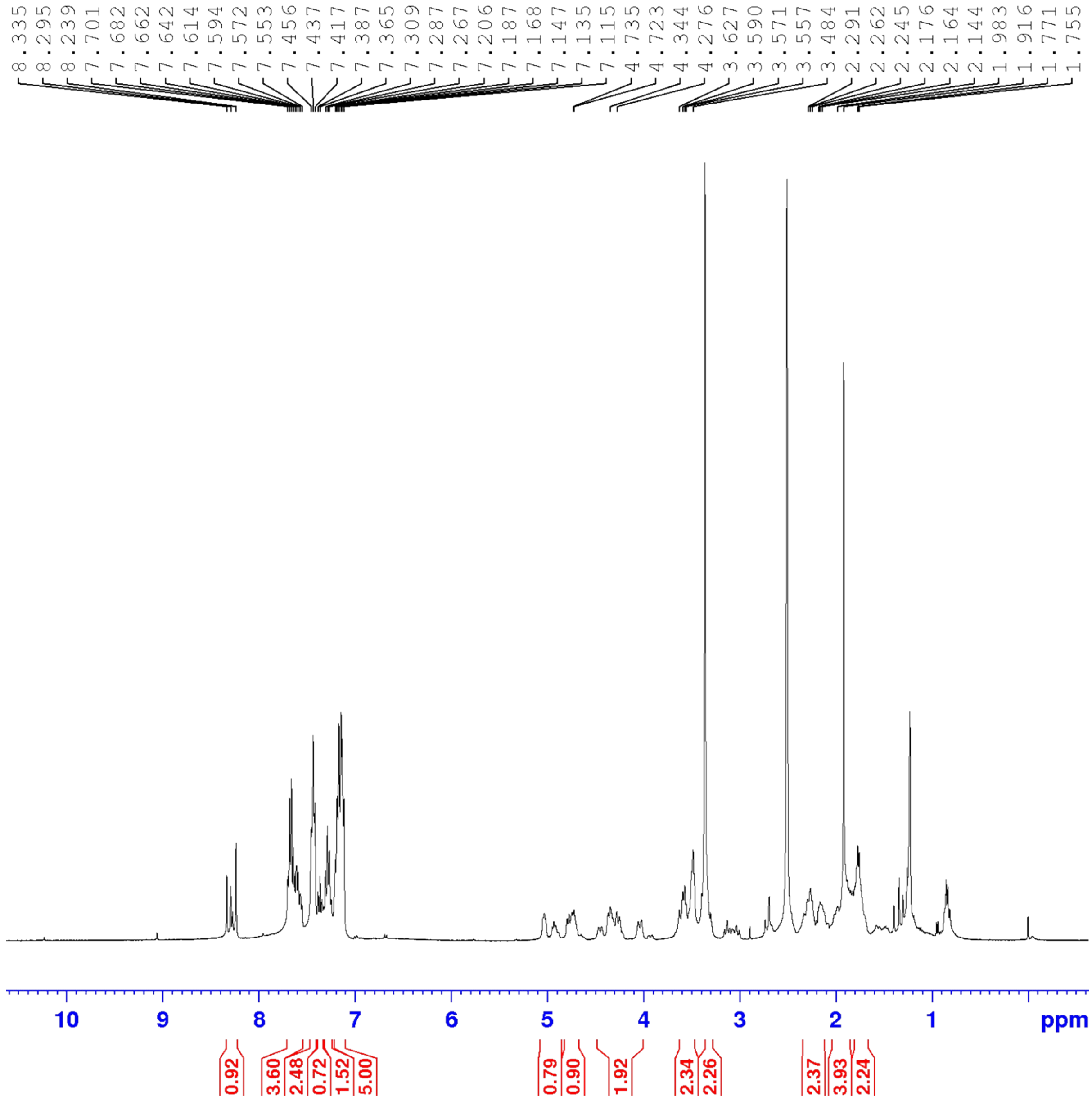

Figure 14. ^{1}H NMR full spectrum of **12c**

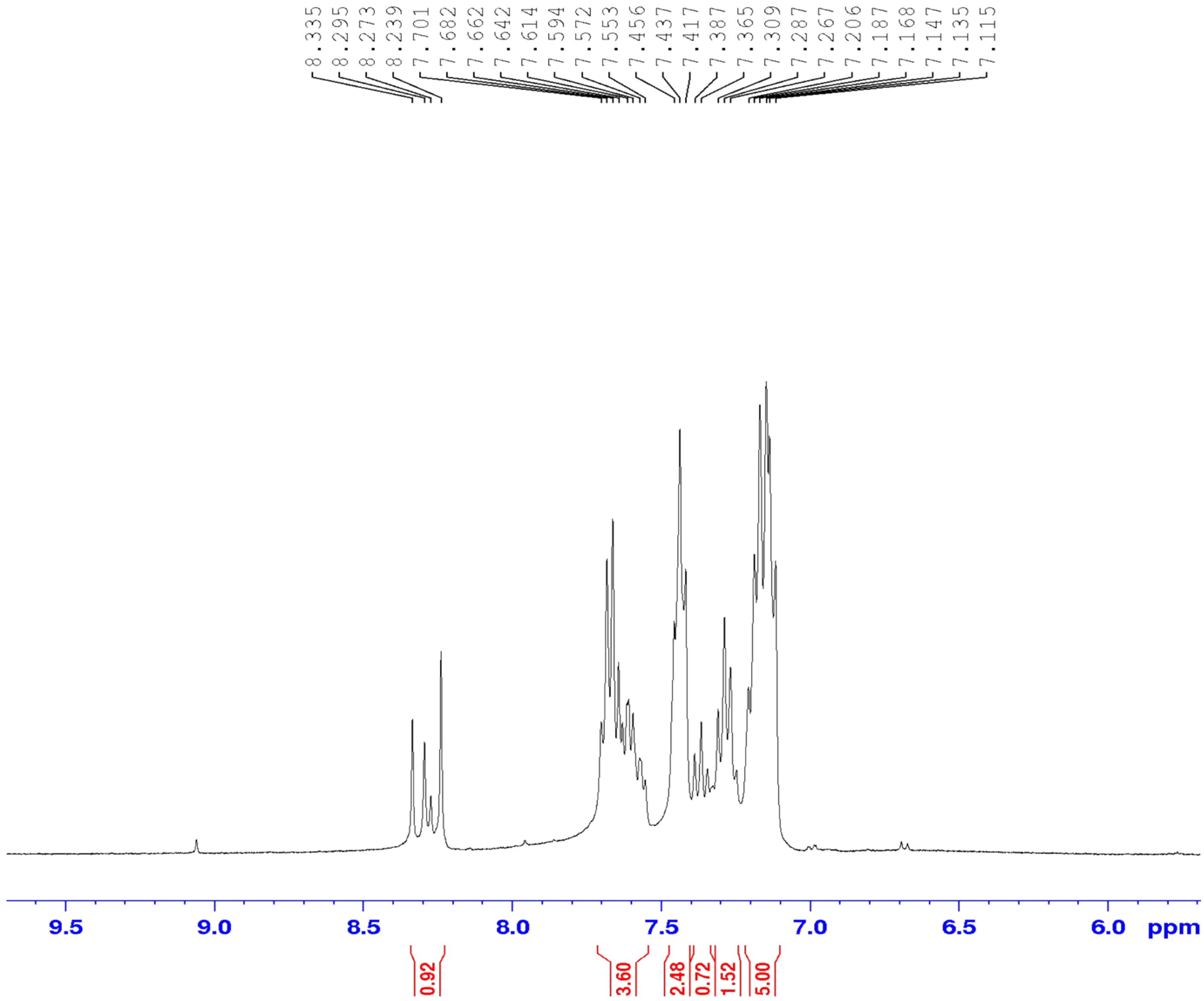

Figure 15. Aromatic region of the ^{1}H NMR spectrum for **12c**

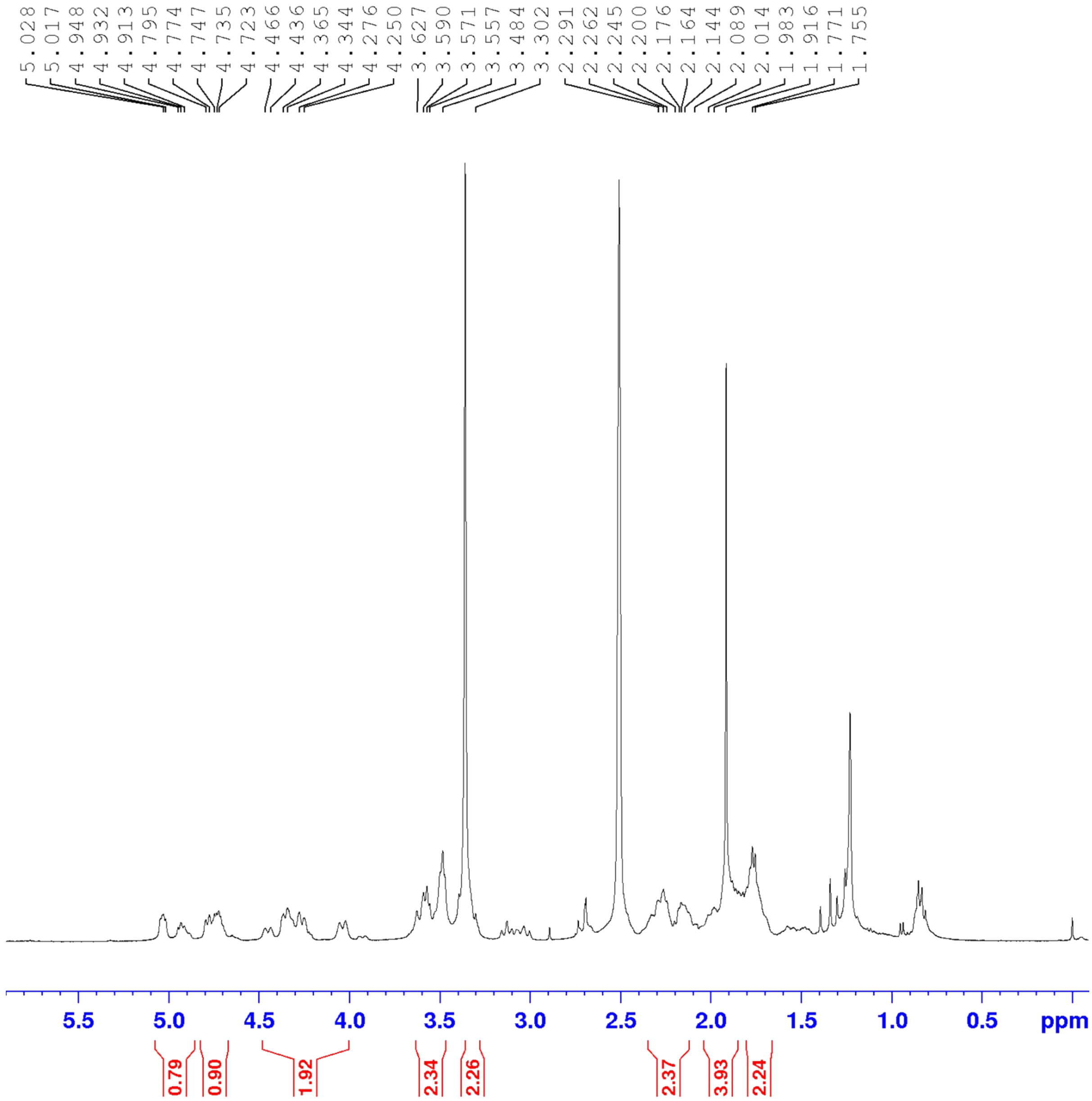

Figure 16. Aliphatic region of the ¹H NMR spectrum for **12c**

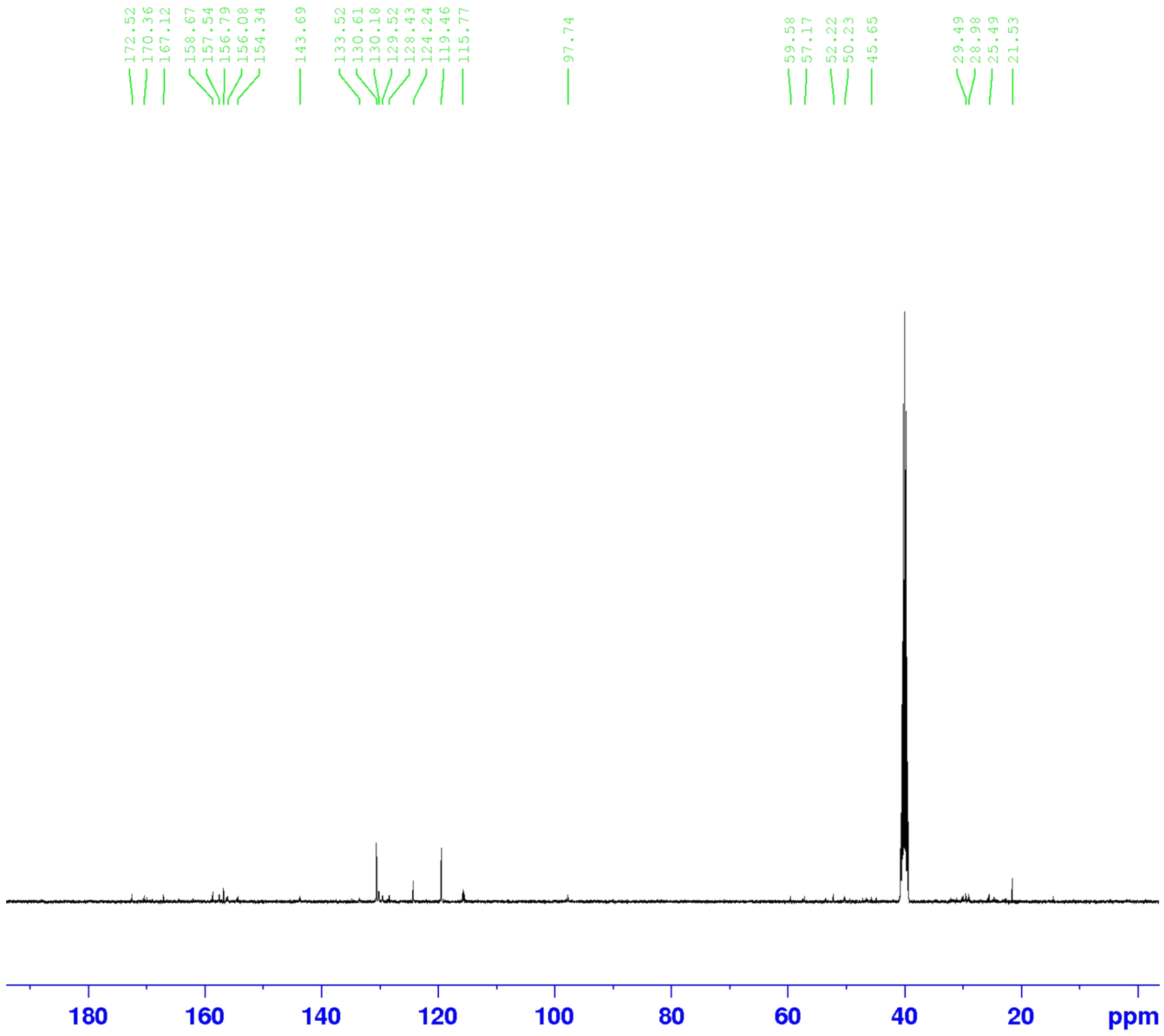

Figure 17. ^{13}C-NMR spectrum of **12c**

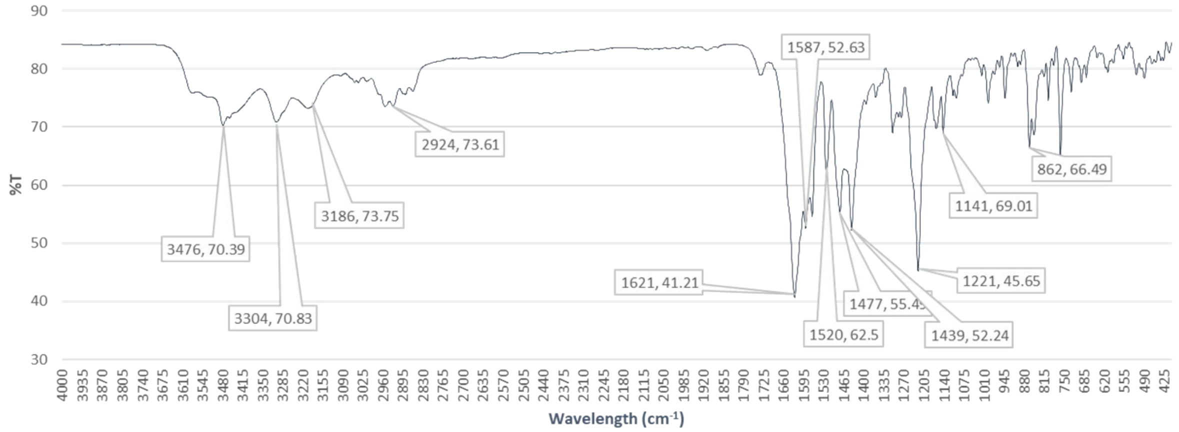

Figure 18. FT-IR spectrum of **12c**

Spectral data of ***4-amino-3-(4-phenoxyphenyl)-1-((3R)-1-((2,4,5-trifluorobenzoyl)prolyl)piperidin-3-yl)-1H-pyrazolo[3,4-d]pyrimidine (12d)***

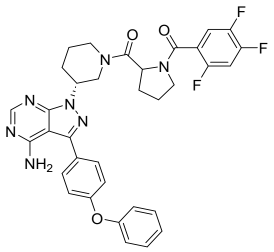

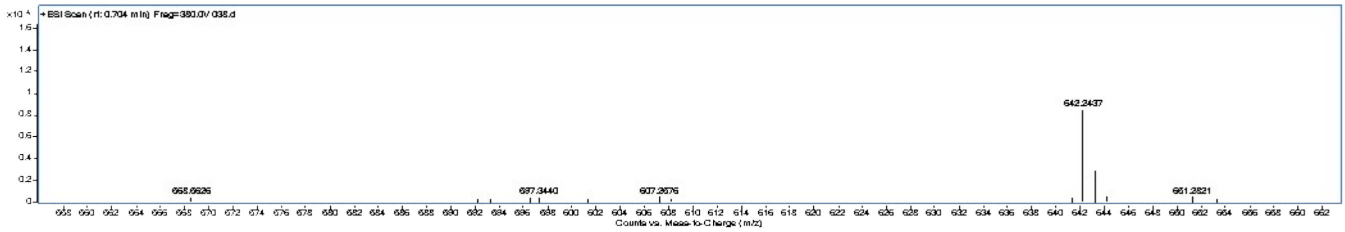

Figure 19. HRMS spectrum of **12d**

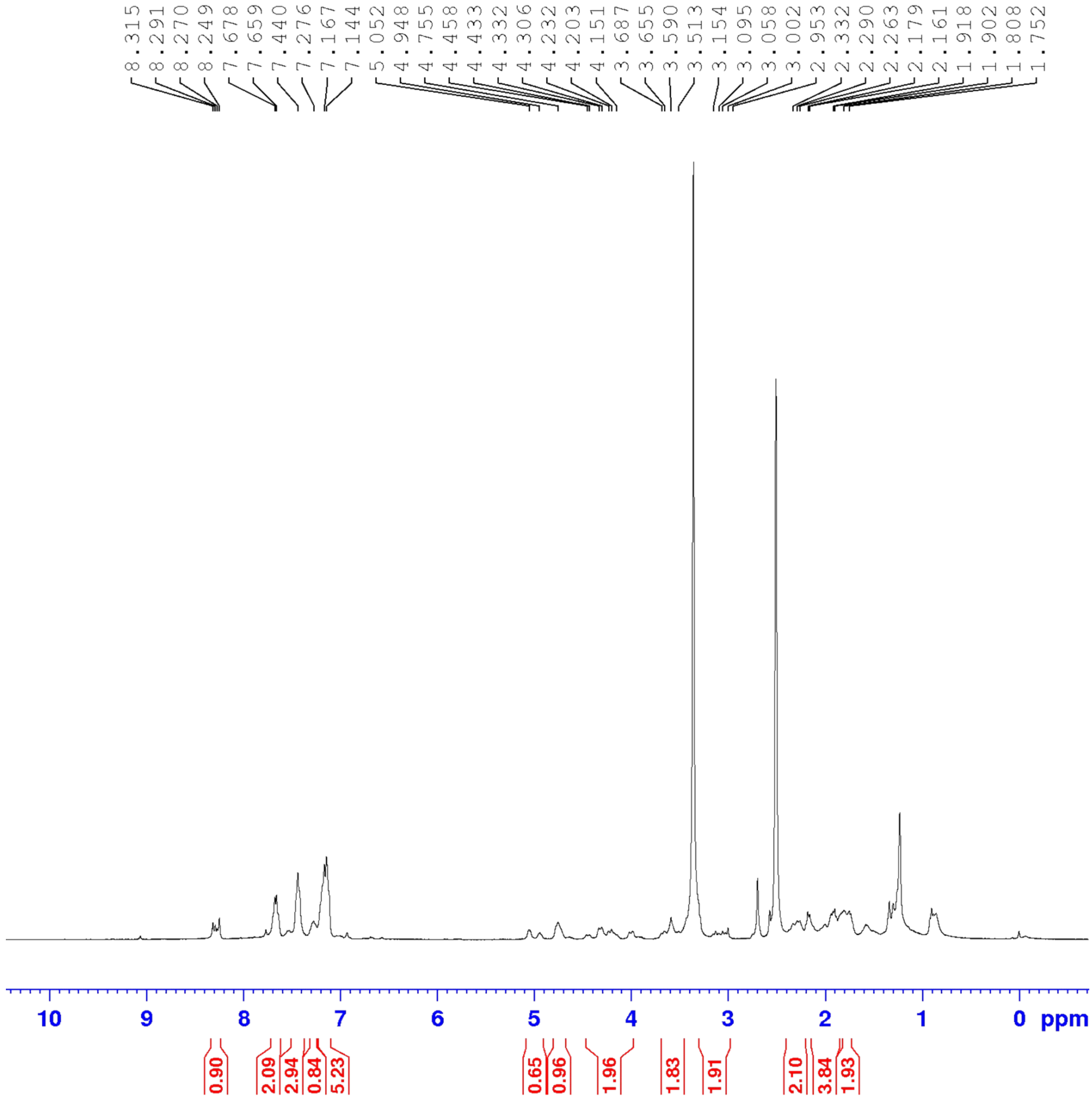

Figure 20. ^{1}H NMR full spectrum of **12d**

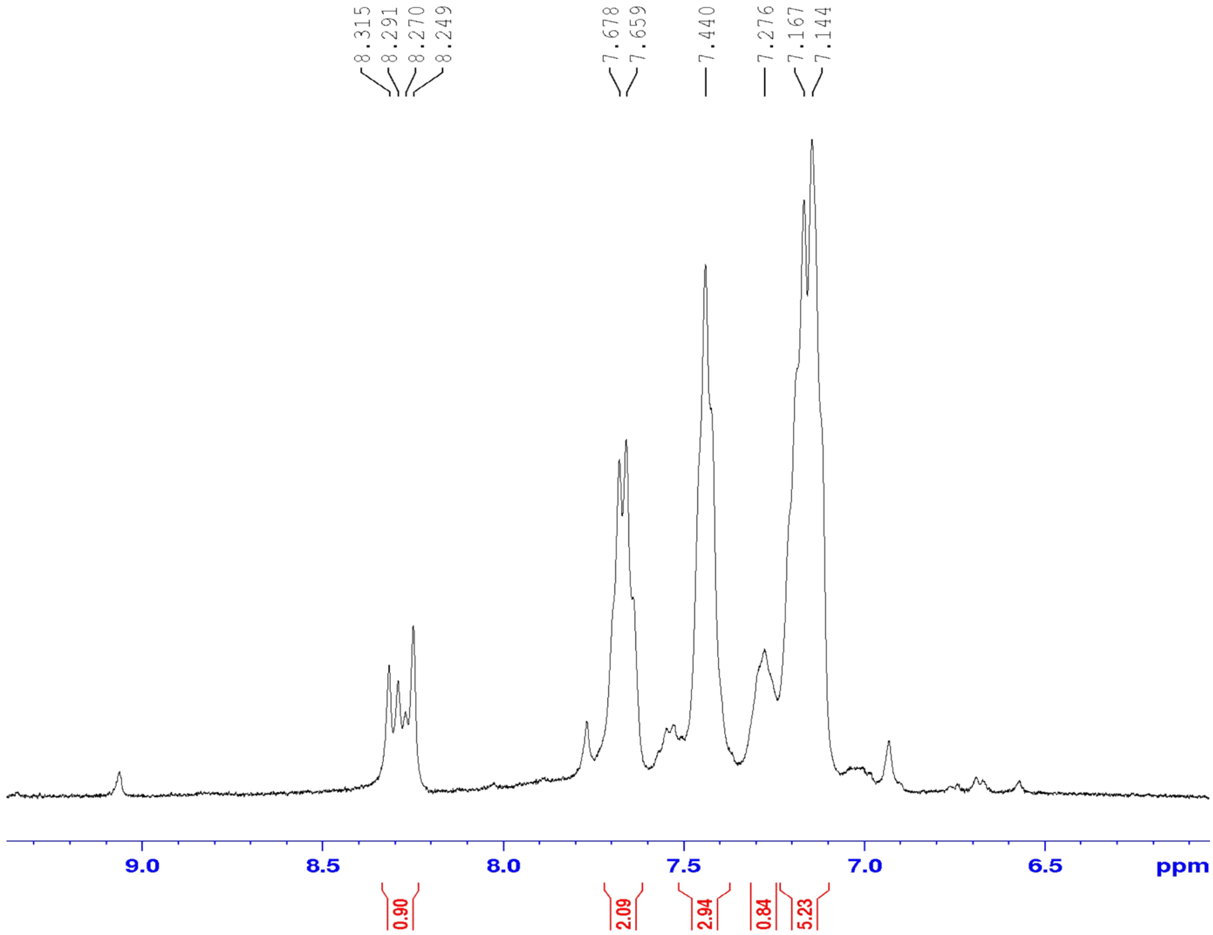

Figure 21. Aromatic region of the ^{1}H NMR spectrum for **12d**

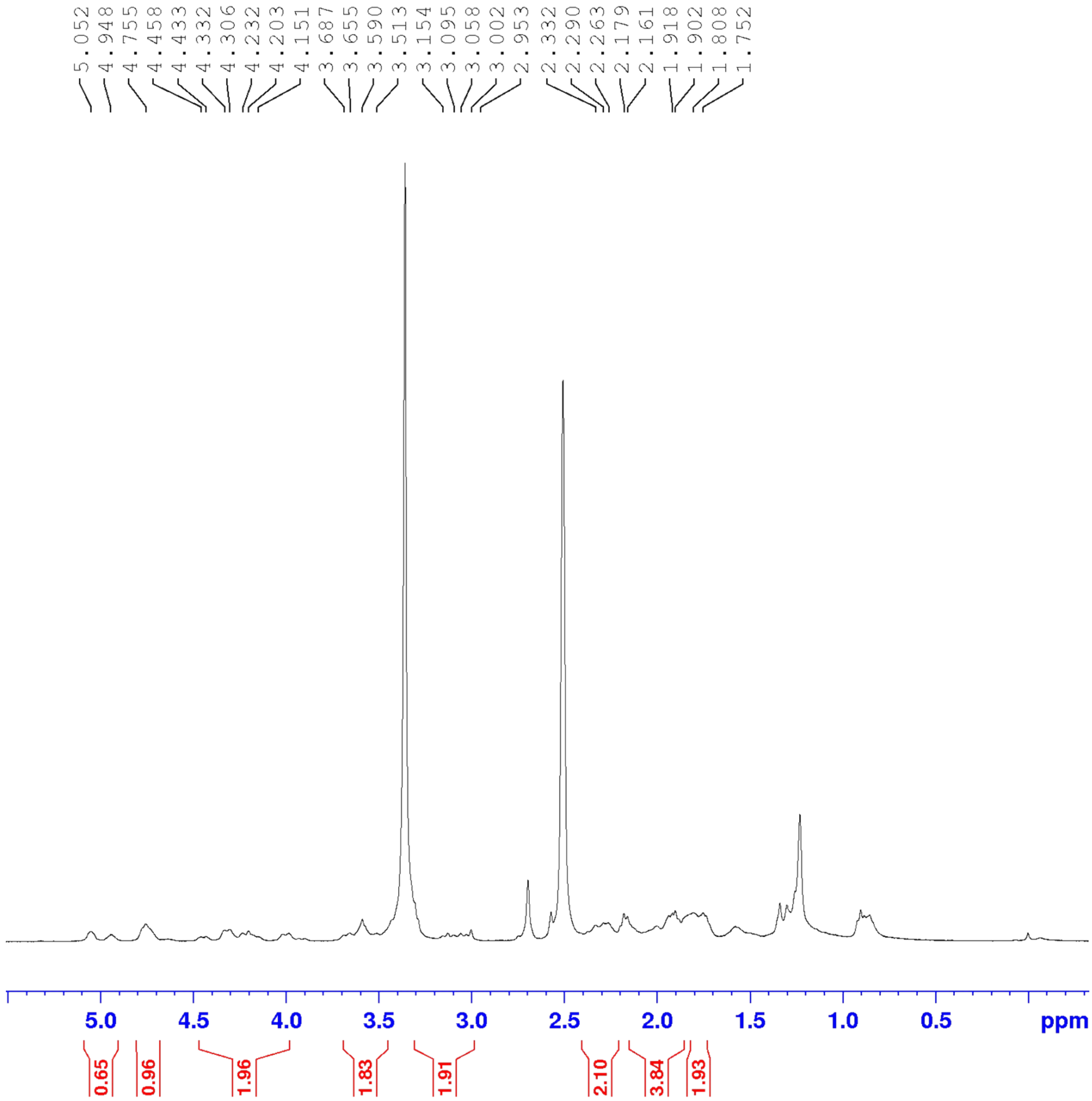

Figure 22. Aliphatic region of the ^{1}H NMR spectrum for **12d**

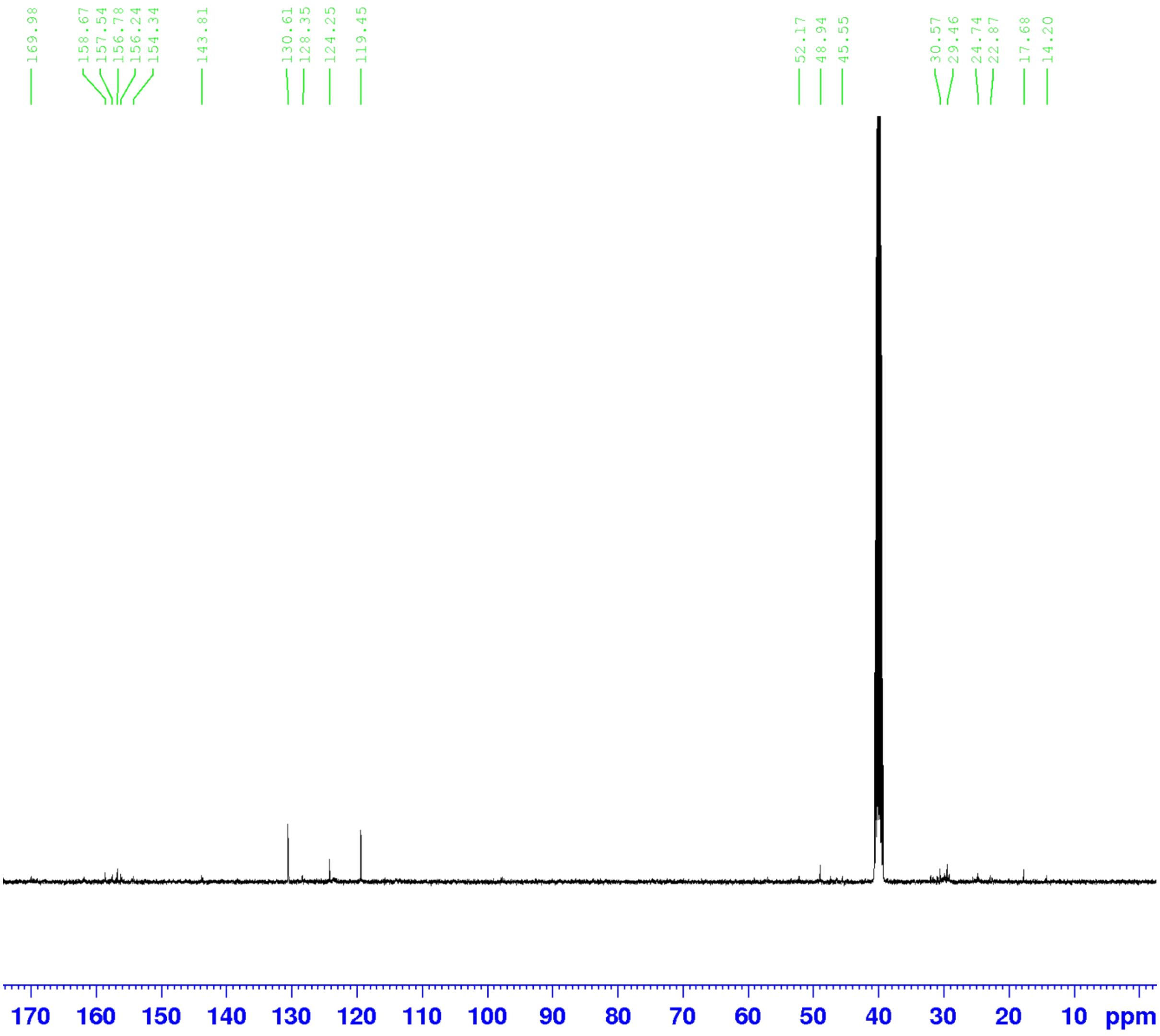

Figure 23. ^{13}C-NMR spectrum of **12d**

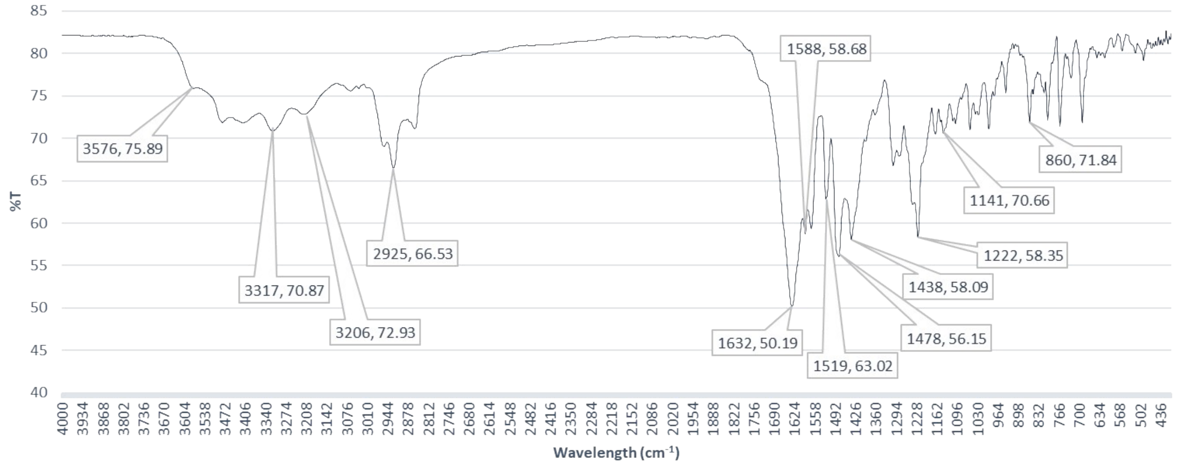

Figure 24. FT-IR spectrum of **12d**

Spectral data of ***4-amino-1-((3R)-1-((4-chloropicolinoyl)prolyl)piperidin-3-yl)-3-(4-phenoxyphenyl)-1H-pyrazolo[3,4-d]pyrimidine (12e)***

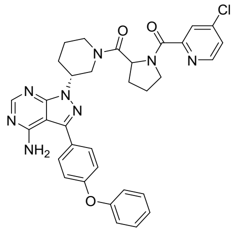

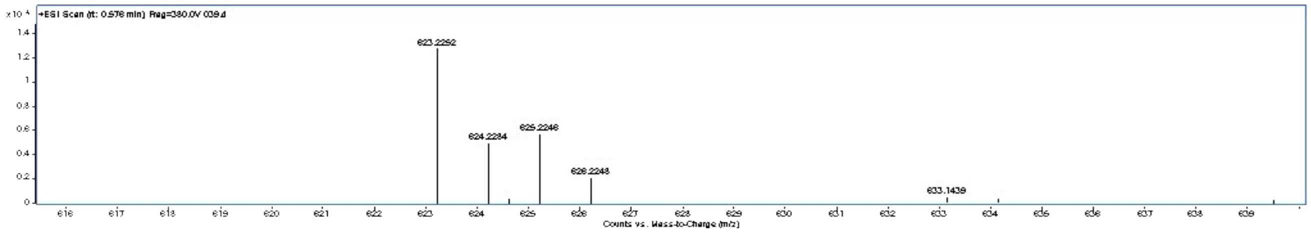

Figure 25. HRMS spectrum of **12e**

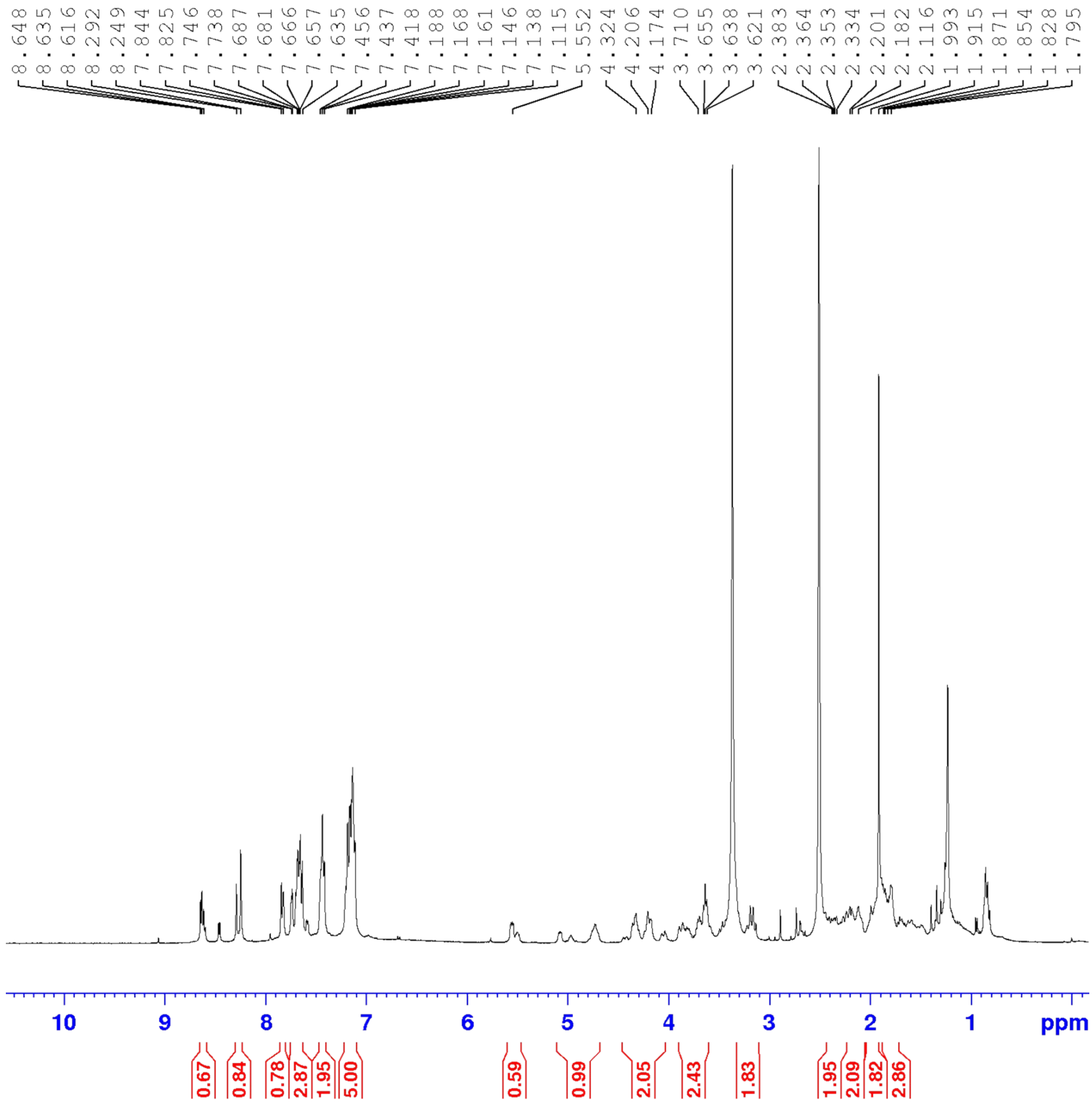

Figure 26. ^{1}H NMR full spectrum of **12e**

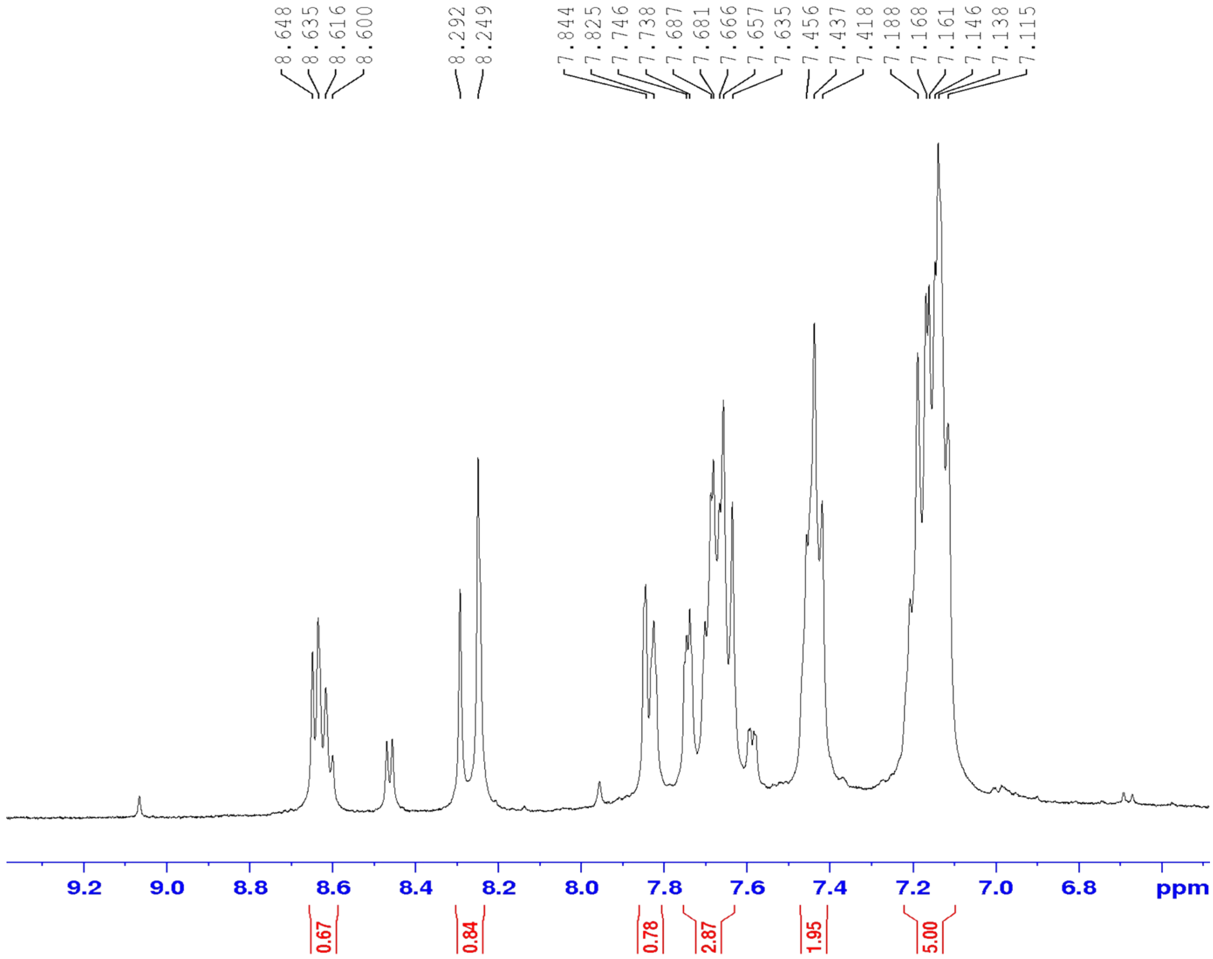

Figure 27. Aromatic region of the ^{1}H NMR spectrum for **12e**

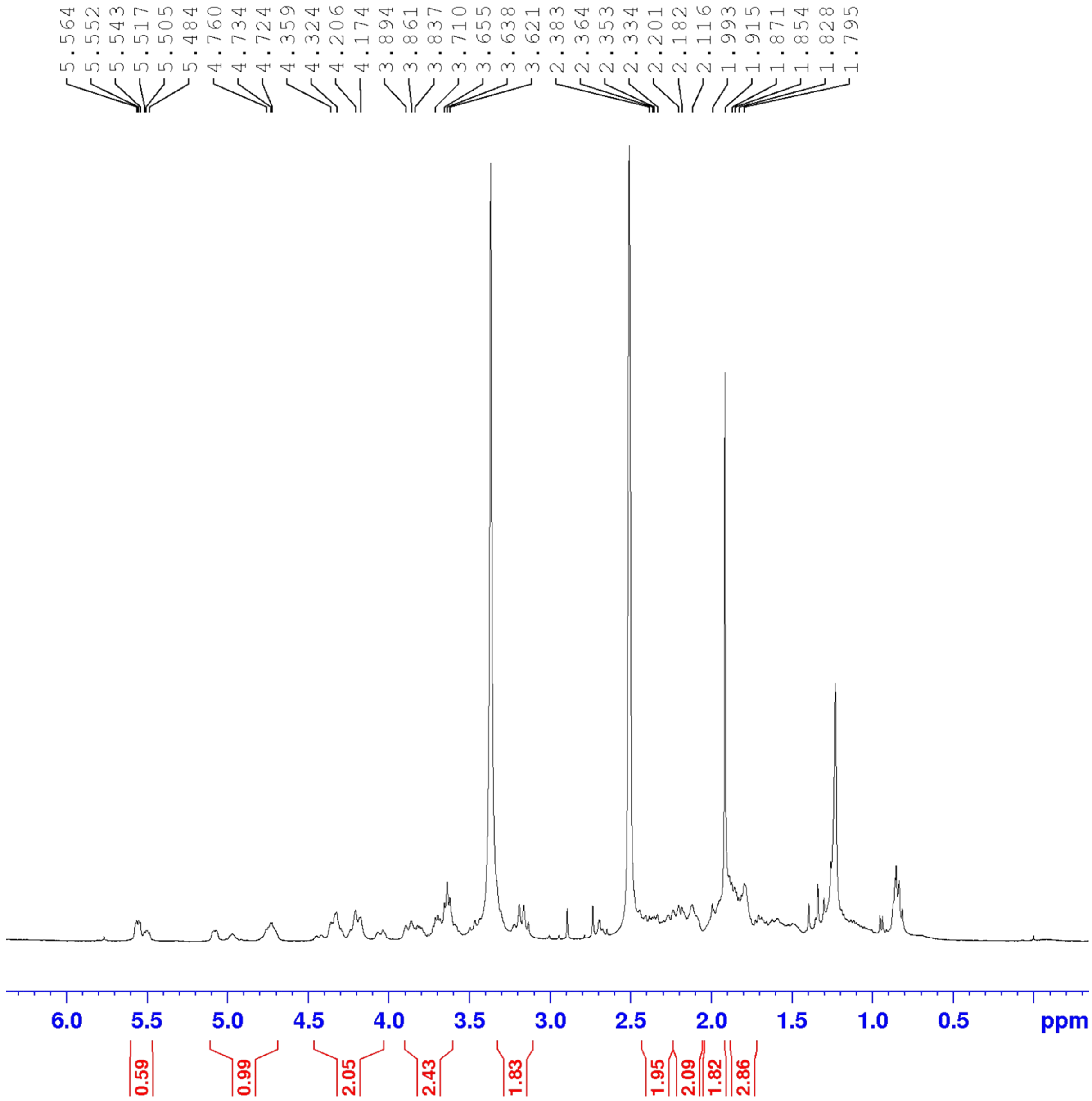

Figure 28. Aliphatic region of the ^{1}H NMR spectrum for **12e**

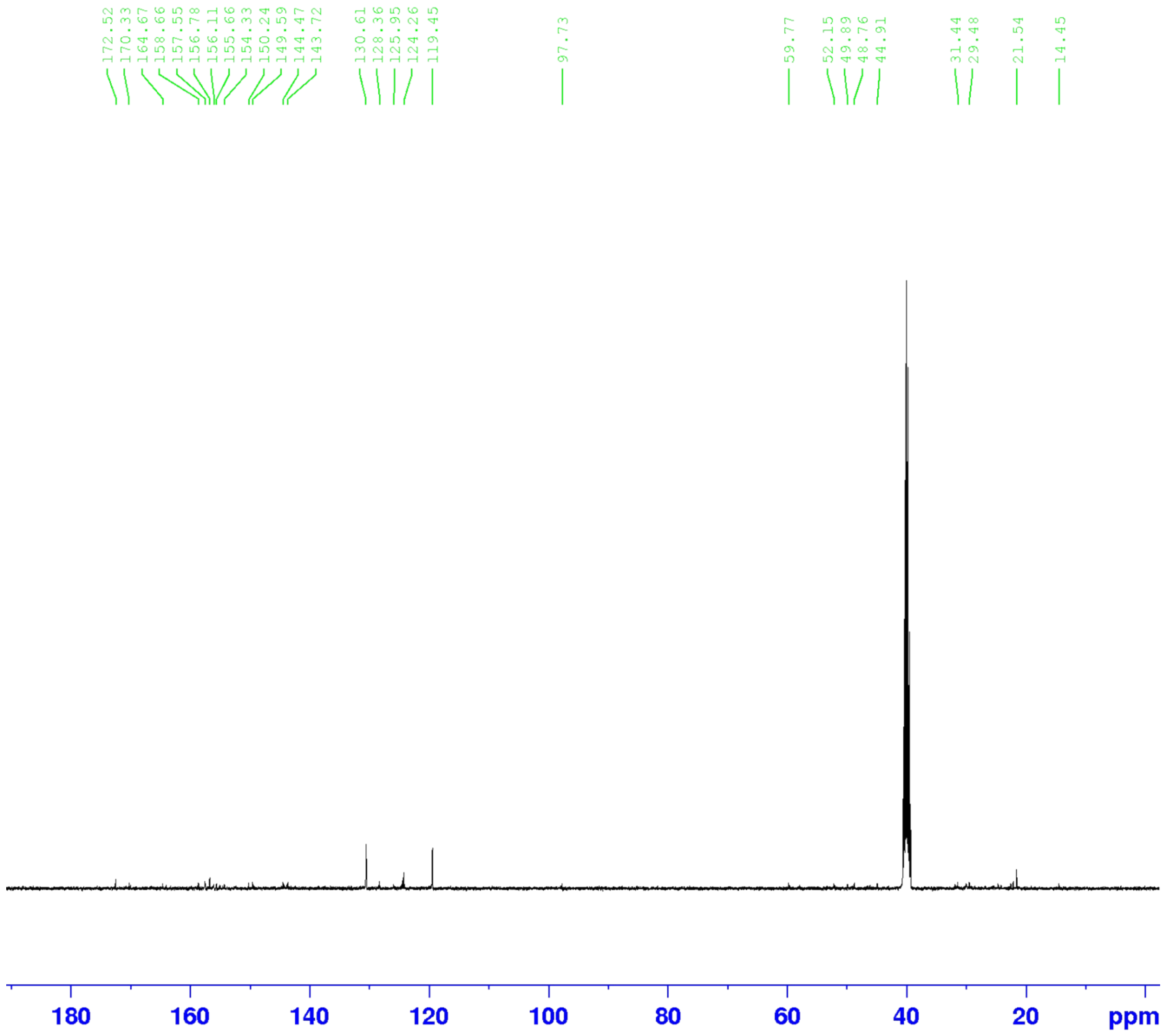

Figure 29. ^{13}C-NMR spectrum of **12e**

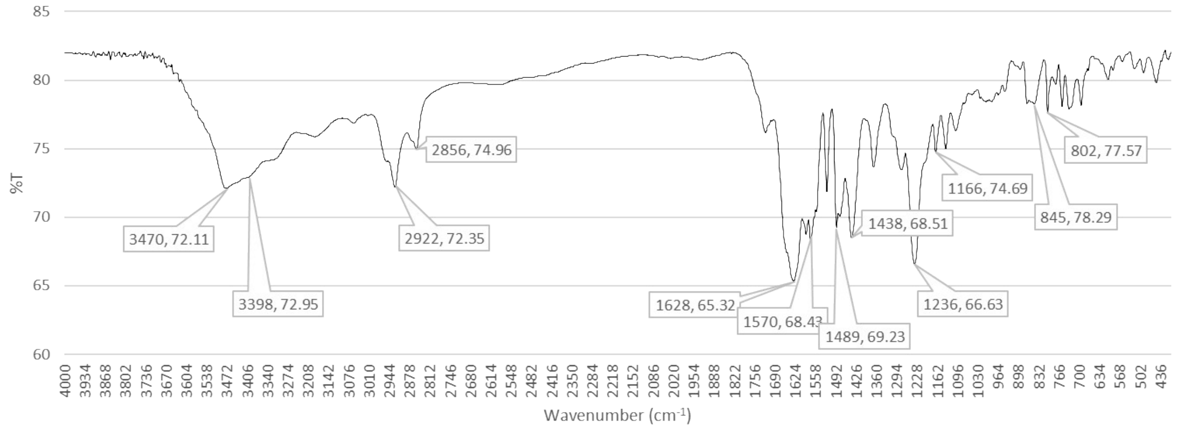

Figure 30. FT-IR spectrum of **12e**

Spectral data of ***4-amino-1-((3R)-1-((2,4-difluorobenzoyl)prolyl)piperidin-3-yl)-3-(4-phenoxyphenyl)-1H-pyrazolo[3,4-d]pyrimidine (12f)***

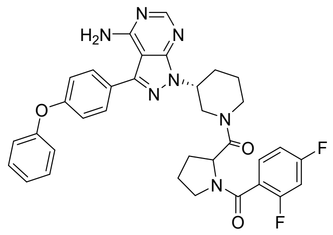

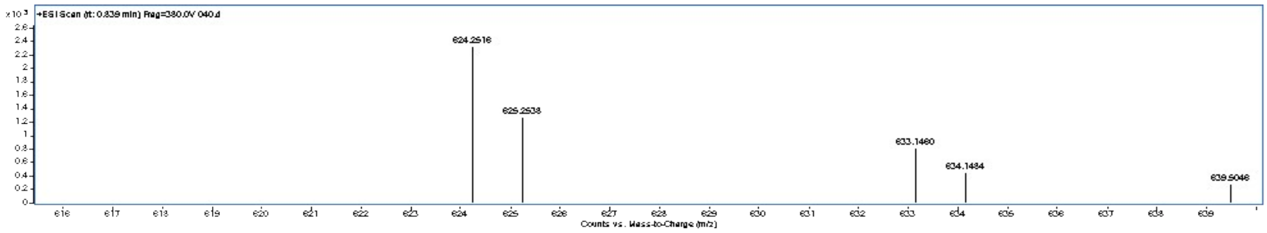

Figure 31. HRMS spectrum of **12f**

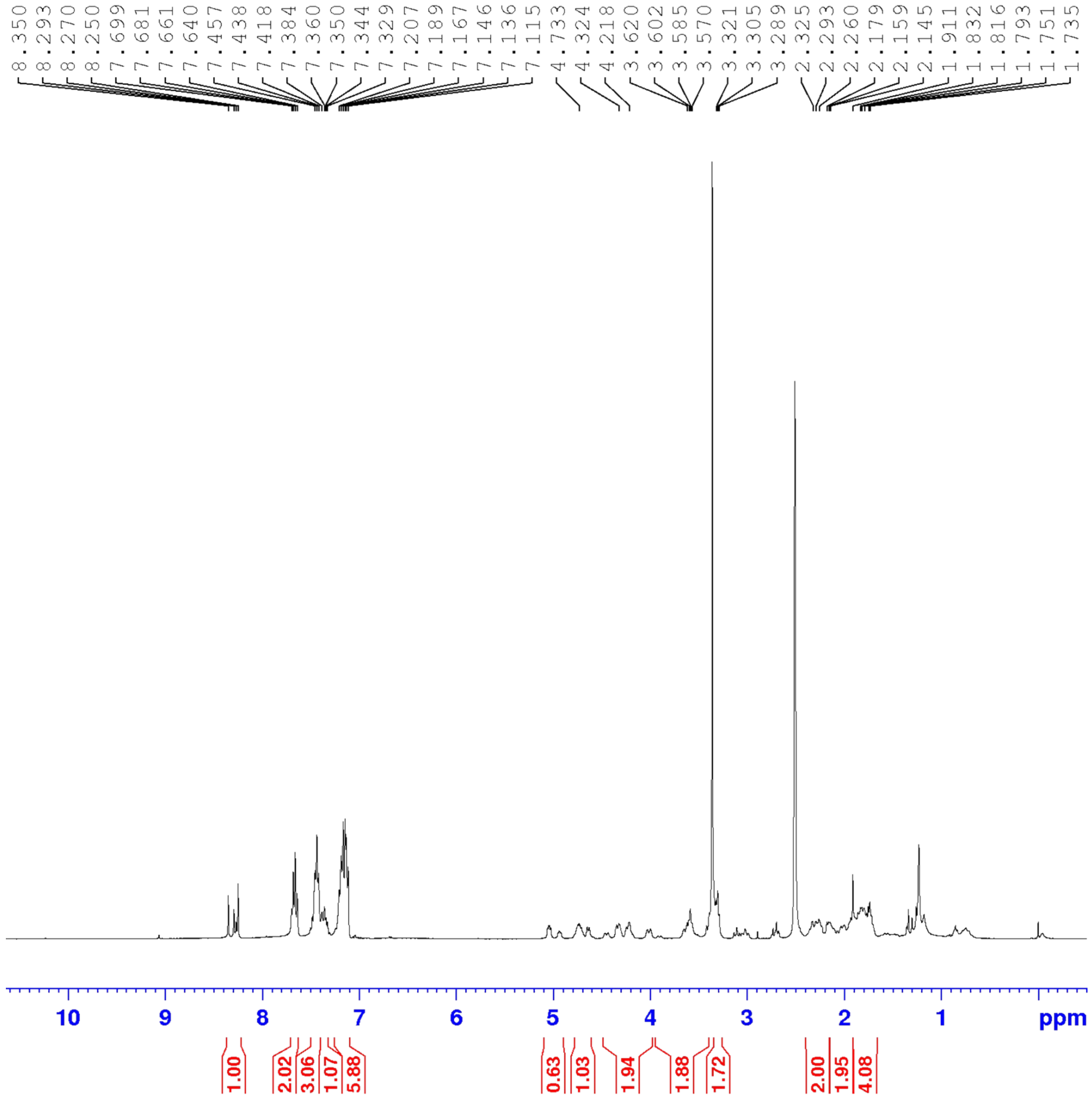

Figure 32. ^{1}H NMR full spectrum of **12f**

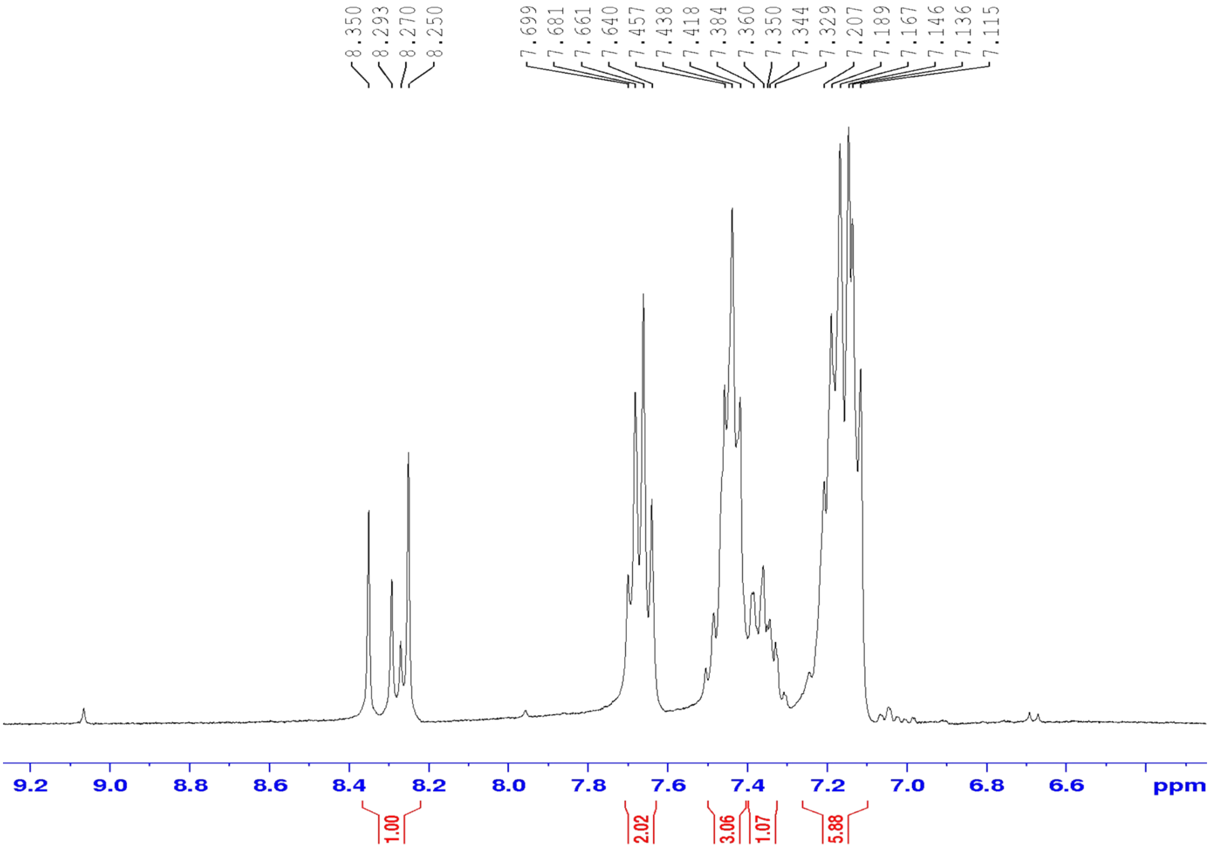

Figure 33. Aromatic region of the ^{1}H NMR spectrum for **12f**

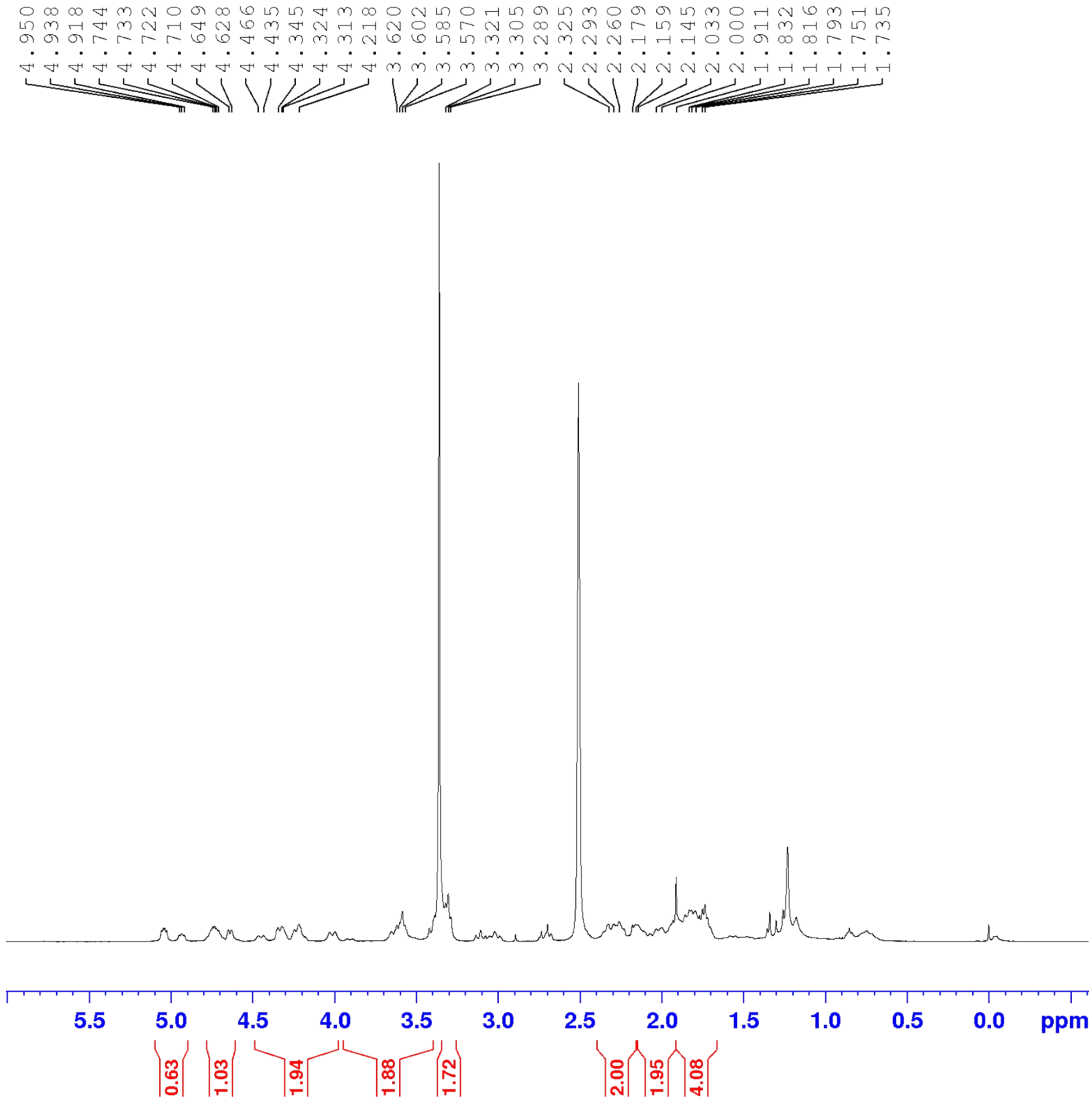

Figure 34. Aliphatic region of the ^{1}H NMR spectrum for **12f**

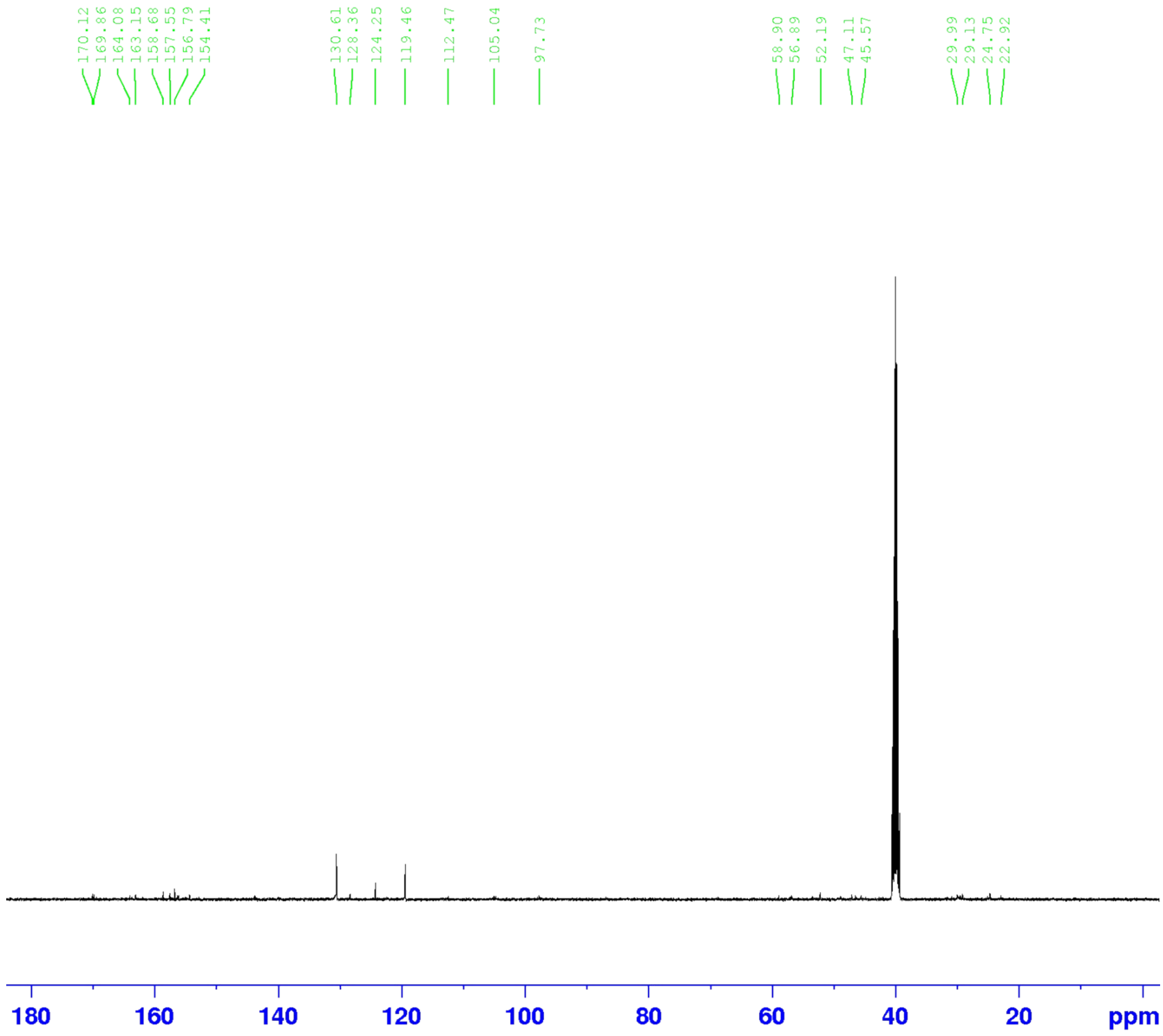

Figure 35. ^{13}C-NMR spectrum of **12f**

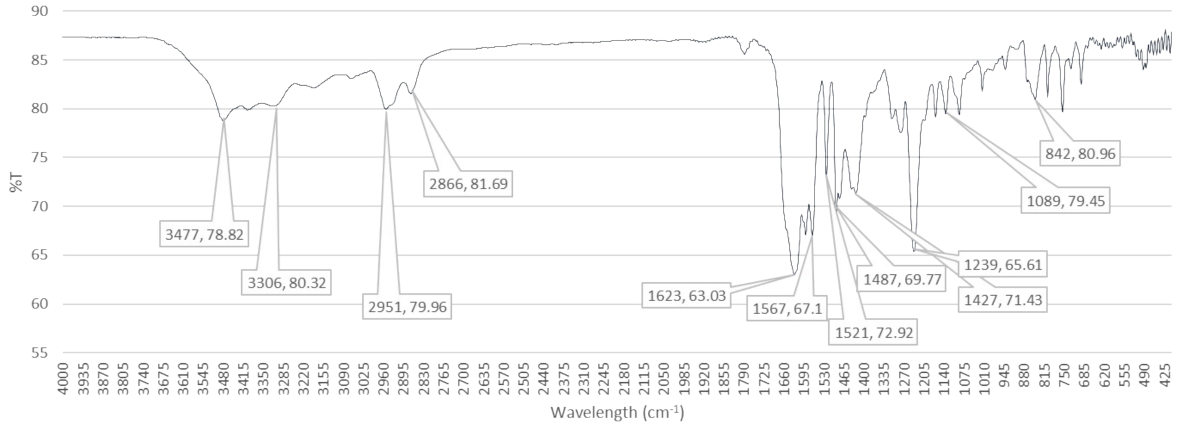

Figure 36. FT-IR spectrum of **12f**

Spectral data of ***4-amino-1-((3R)-1-(benzoylprolyl)piperidin-3-yl)-3-(4-phenoxyphenyl)-1H-pyrazolo[3,4-d]pyrimidine (12g)***

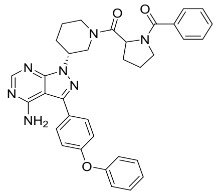

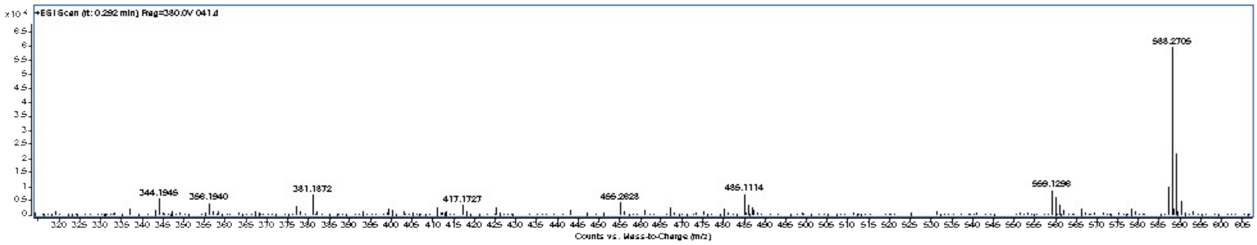

Figure 37. HRMS spectrum of **12g**

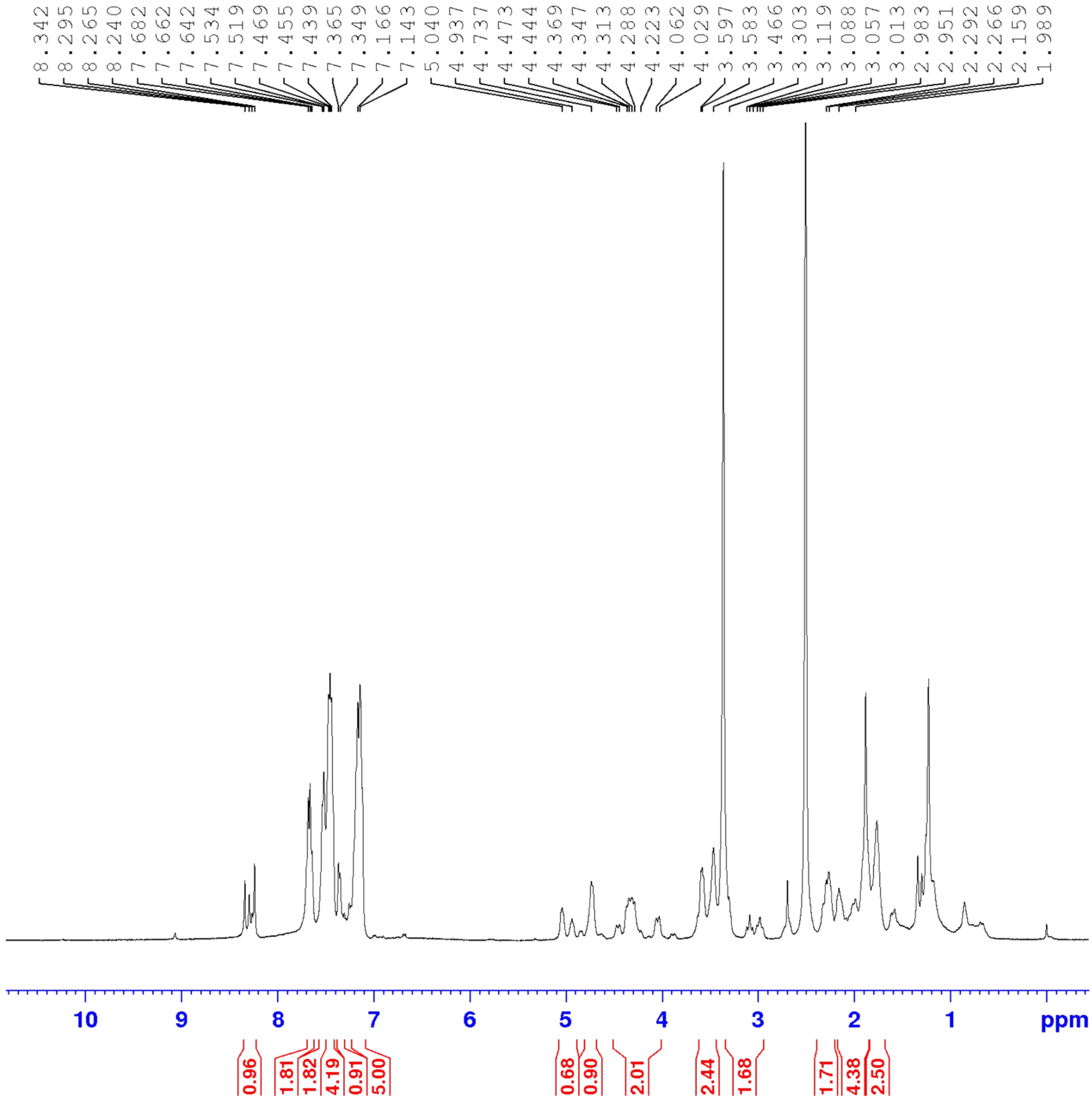

Figure 38. ^{1}H NMR full spectrum of **12g**

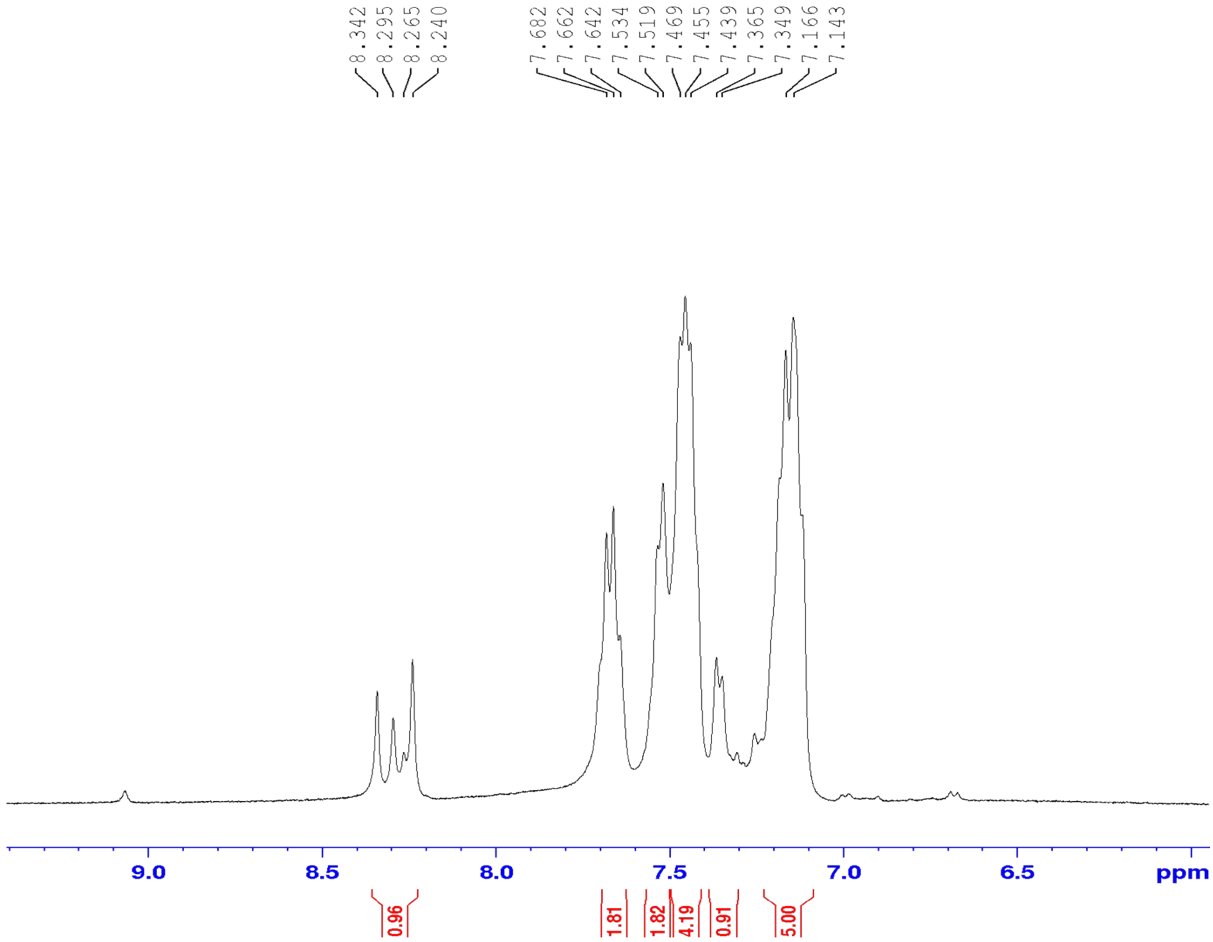

Figure 39. Aromatic region of the ^{1}H NMR spectrum for **12g**

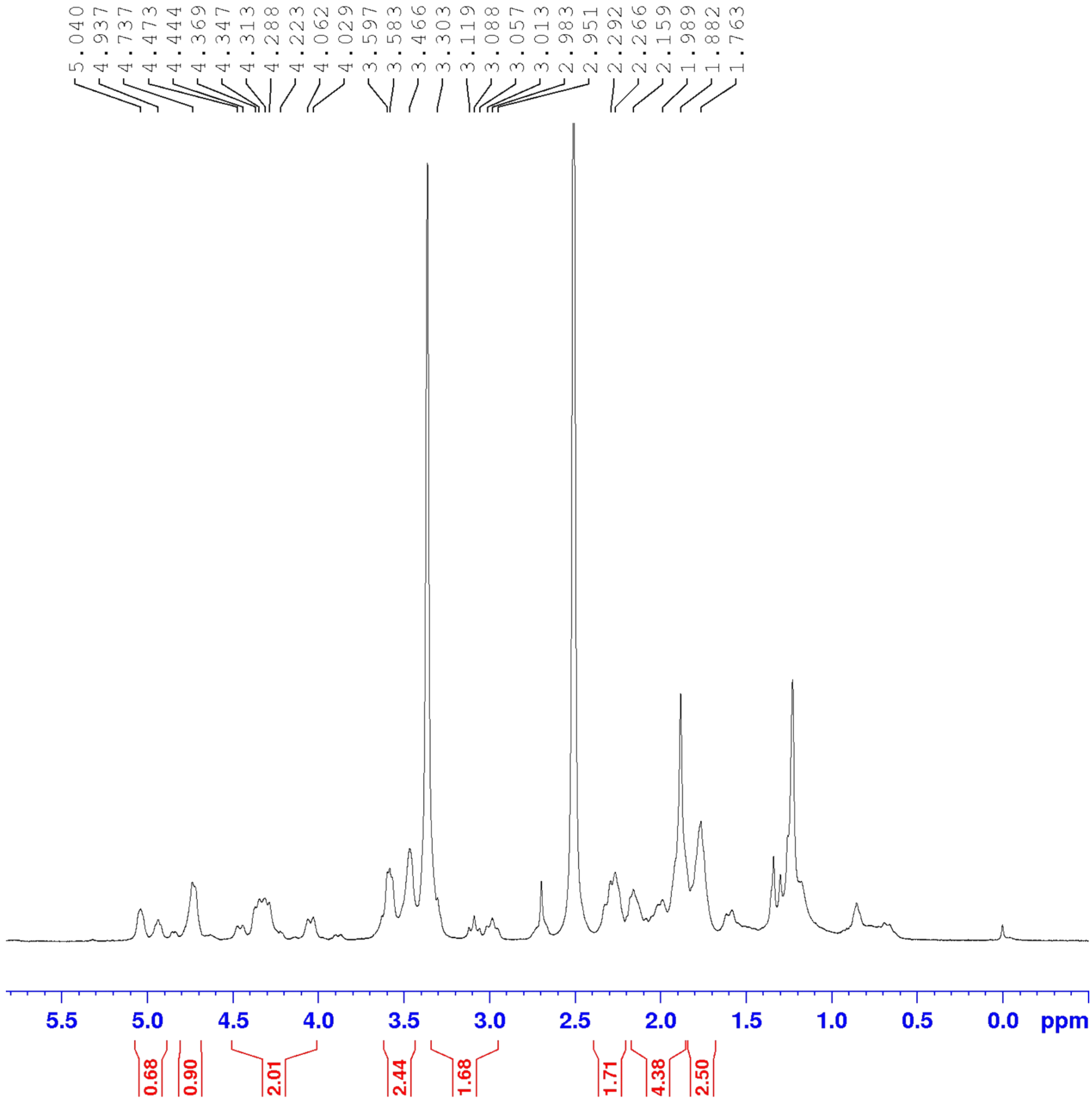

Figure 40. Aliphatic region of the ^{1}H NMR spectrum for **12g**

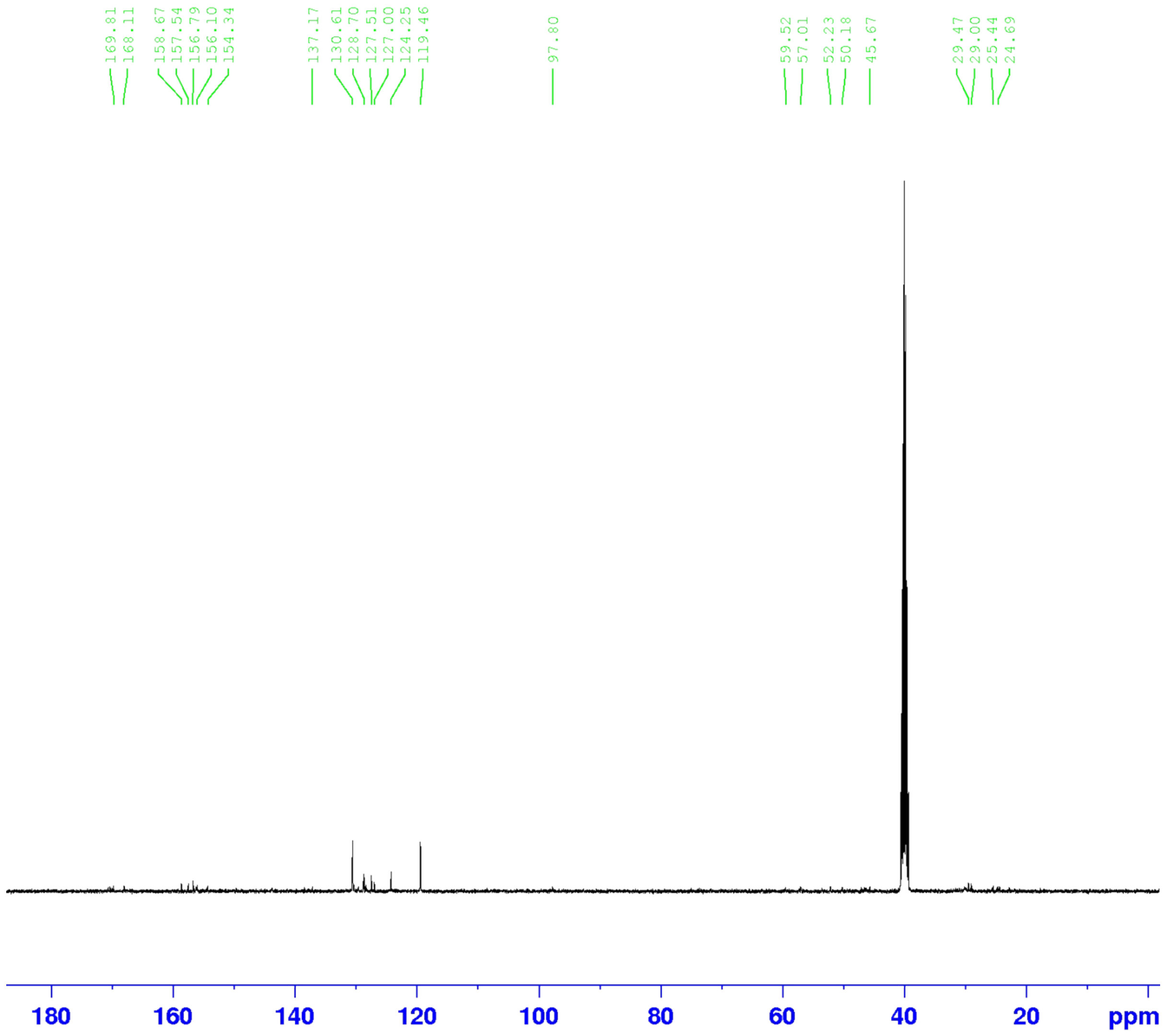

Figure 41. ^{13}C-NMR spectrum of **12g**

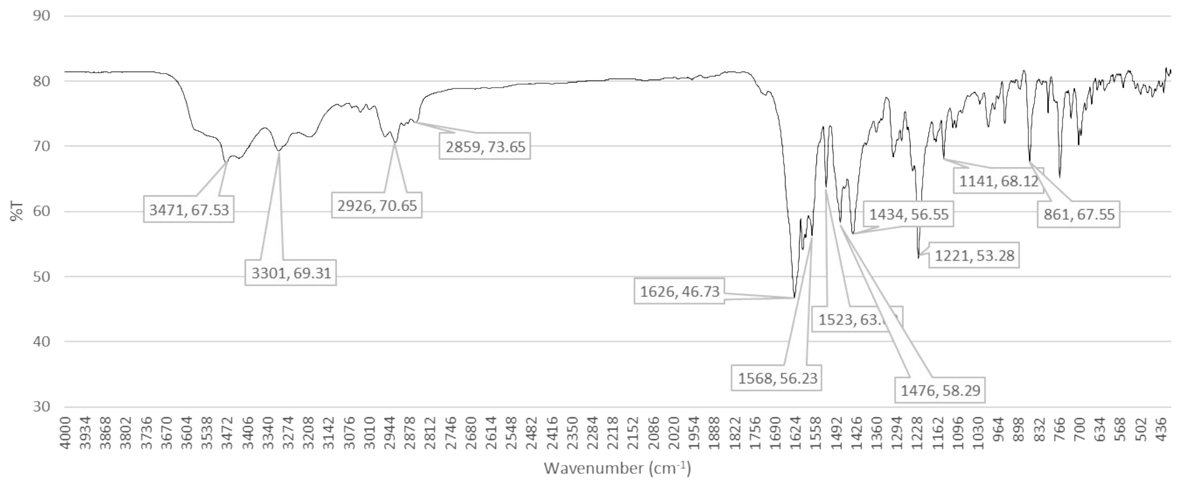

Figure 42. FT-IR spectrum of **12g**

Spectral data of ***4-amino-3-(4-phenoxyphenyl)-1-((3R)-1-(picolinoylprolyl)piperidin-3-yl)-1H-pyrazolo[3,4-d]pyrimidine (12h)***

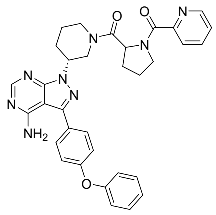

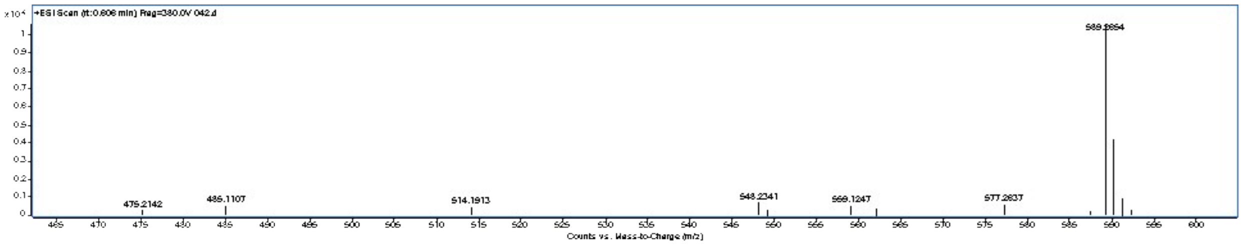

Figure 43. HRMS spectrum of **12h**

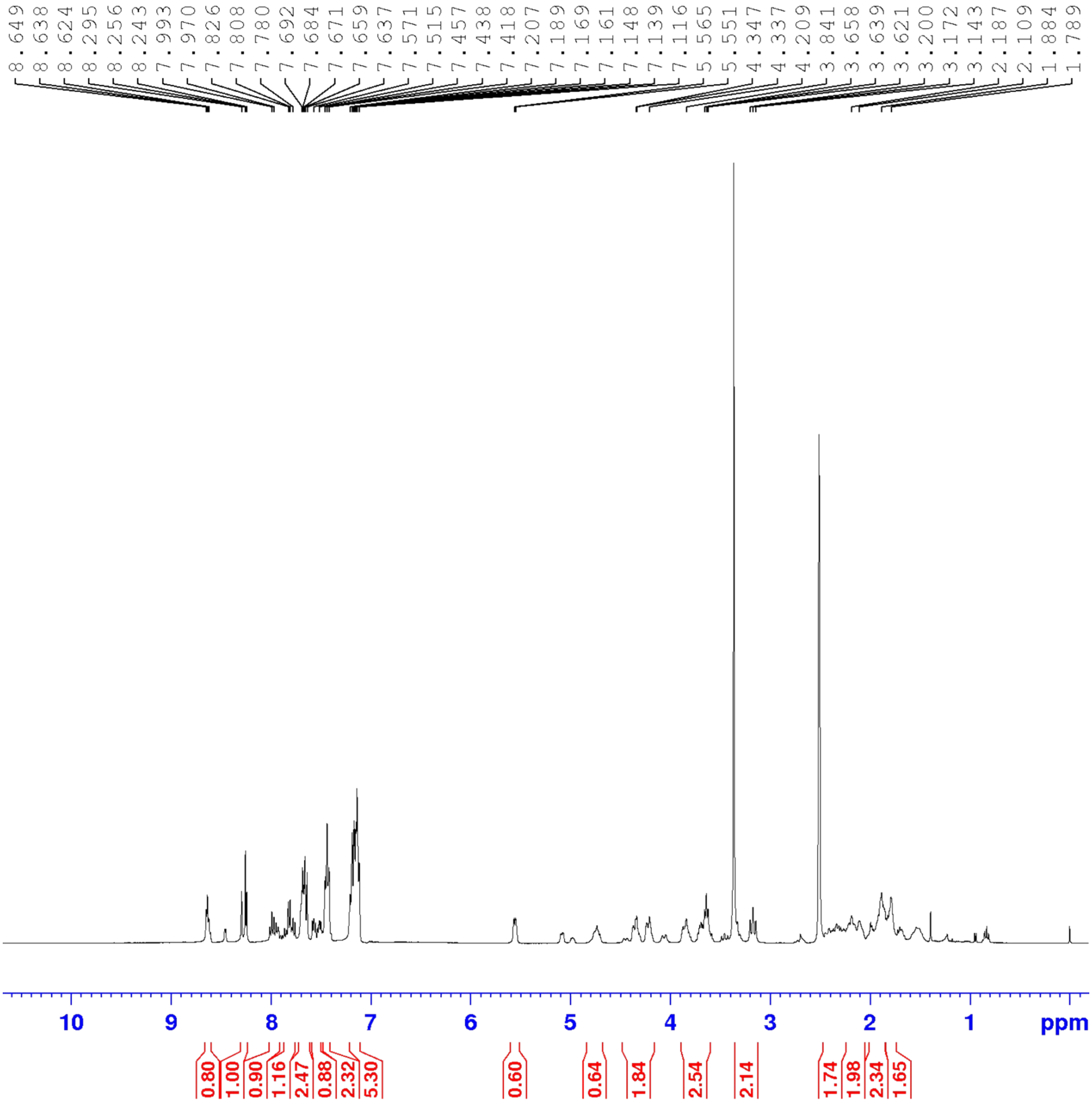

Figure 44. ^{1}H NMR full spectrum of **12h**

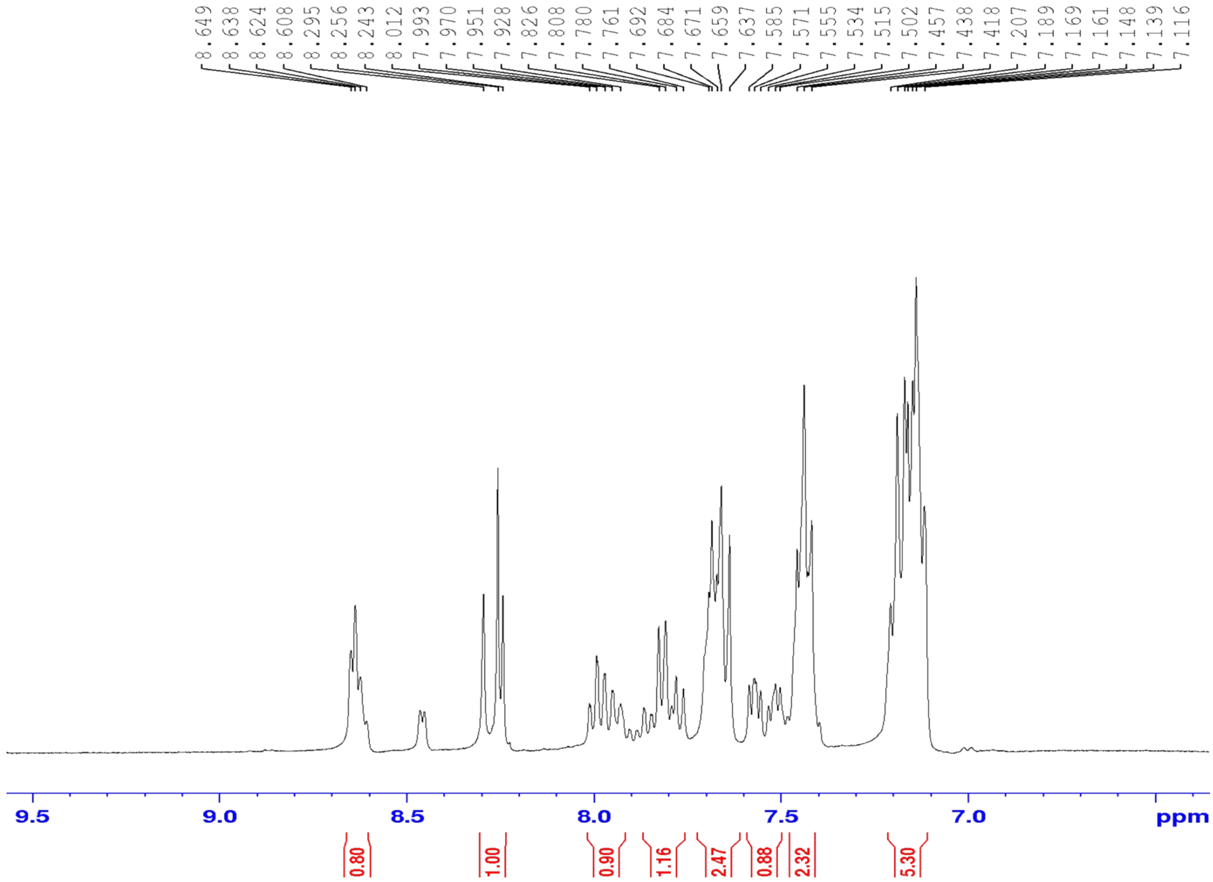

Figure 45. Aromatic region of the ^{1}H NMR spectrum for **12h**

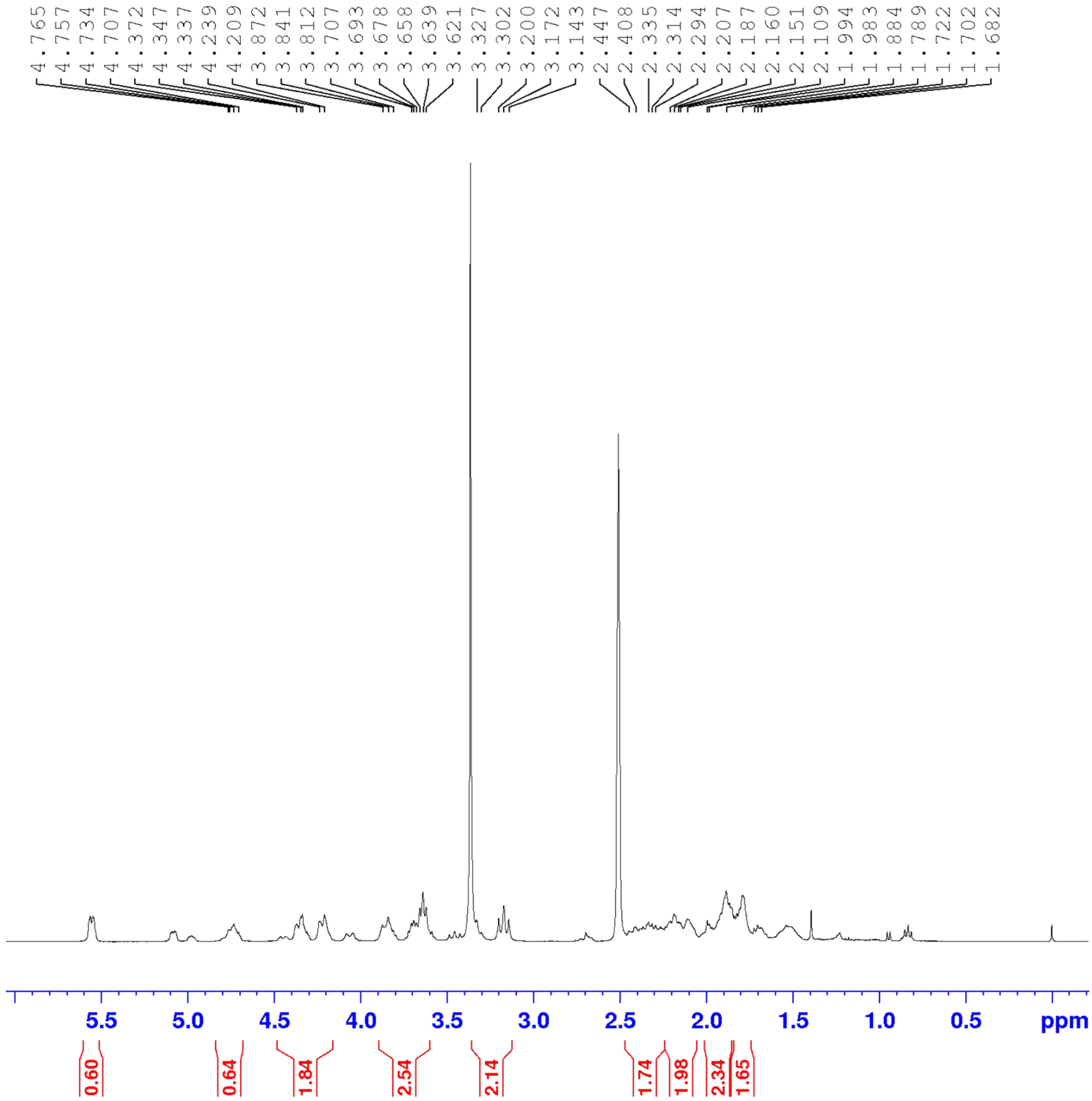

Figure 46. Aliphatic region of the ^{1}H NMR spectrum for **12h**

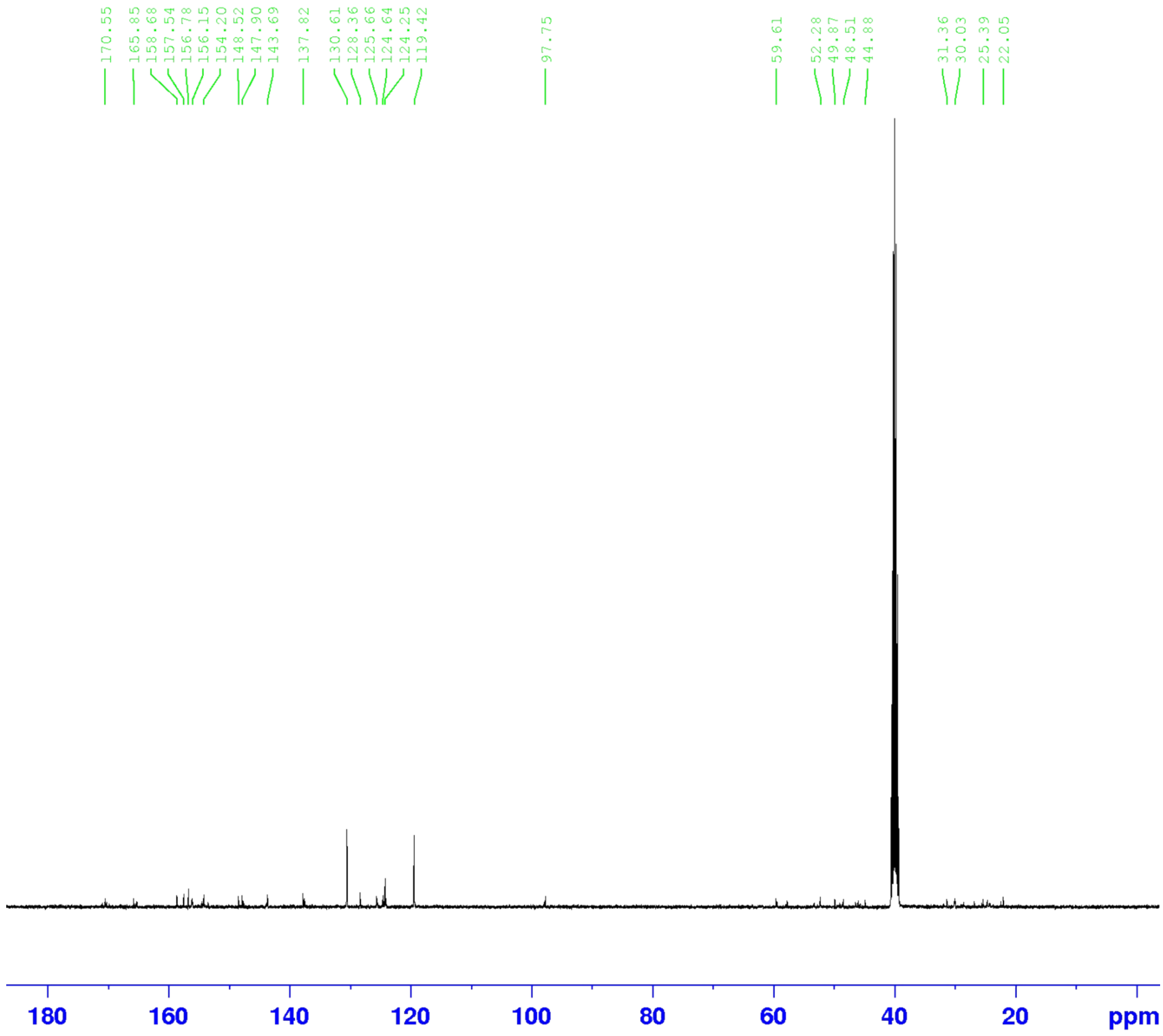

Figure 47. ^{13}C-NMR spectrum of **12h**

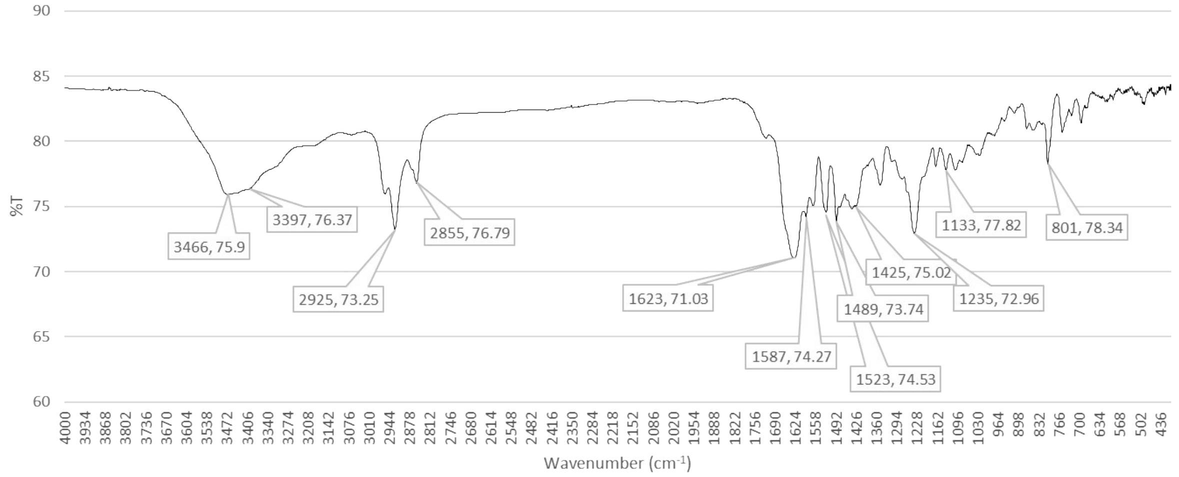

Figure 48. FT-IR spectrum of **12h**

Spectral data of ***4-amino-1-((3R)-1-((3-methyl-4-nitrobenzoyl)prolyl)piperidin-3-yl)-3-(4-phenoxyphenyl)-1H-pyrazolo[3,4-d]pyrimidine (12i)***

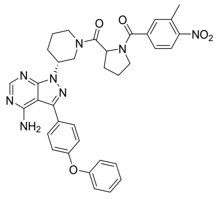

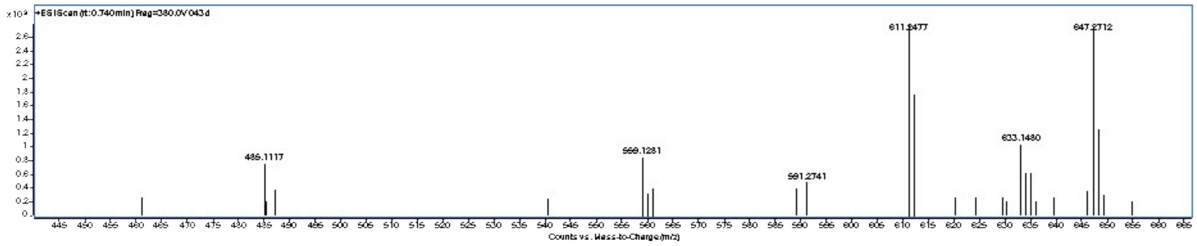

Figure 49. HRMS spectrum of **12i**

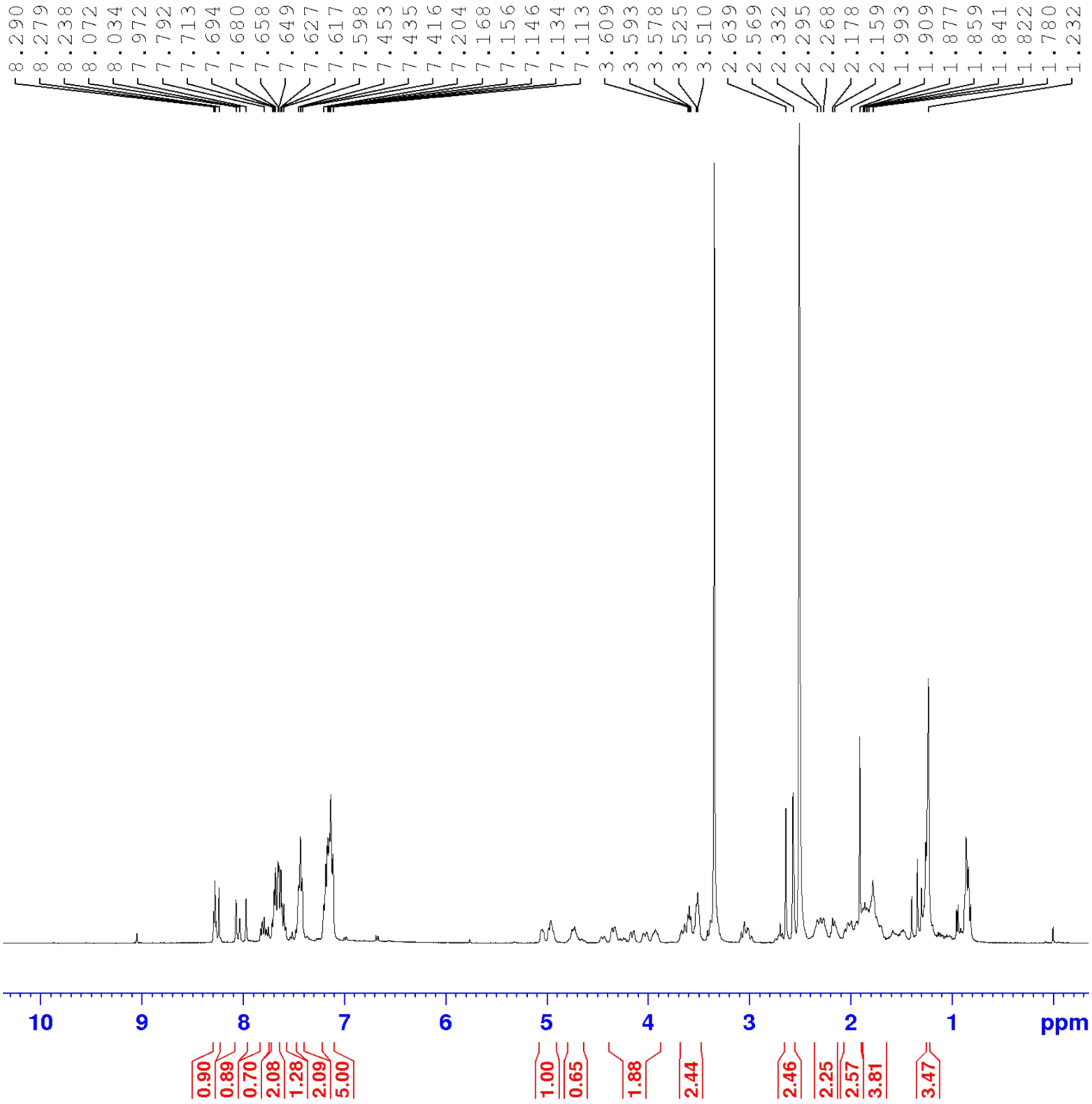

Figure 50. ^{1}H NMR full spectrum of **12i**

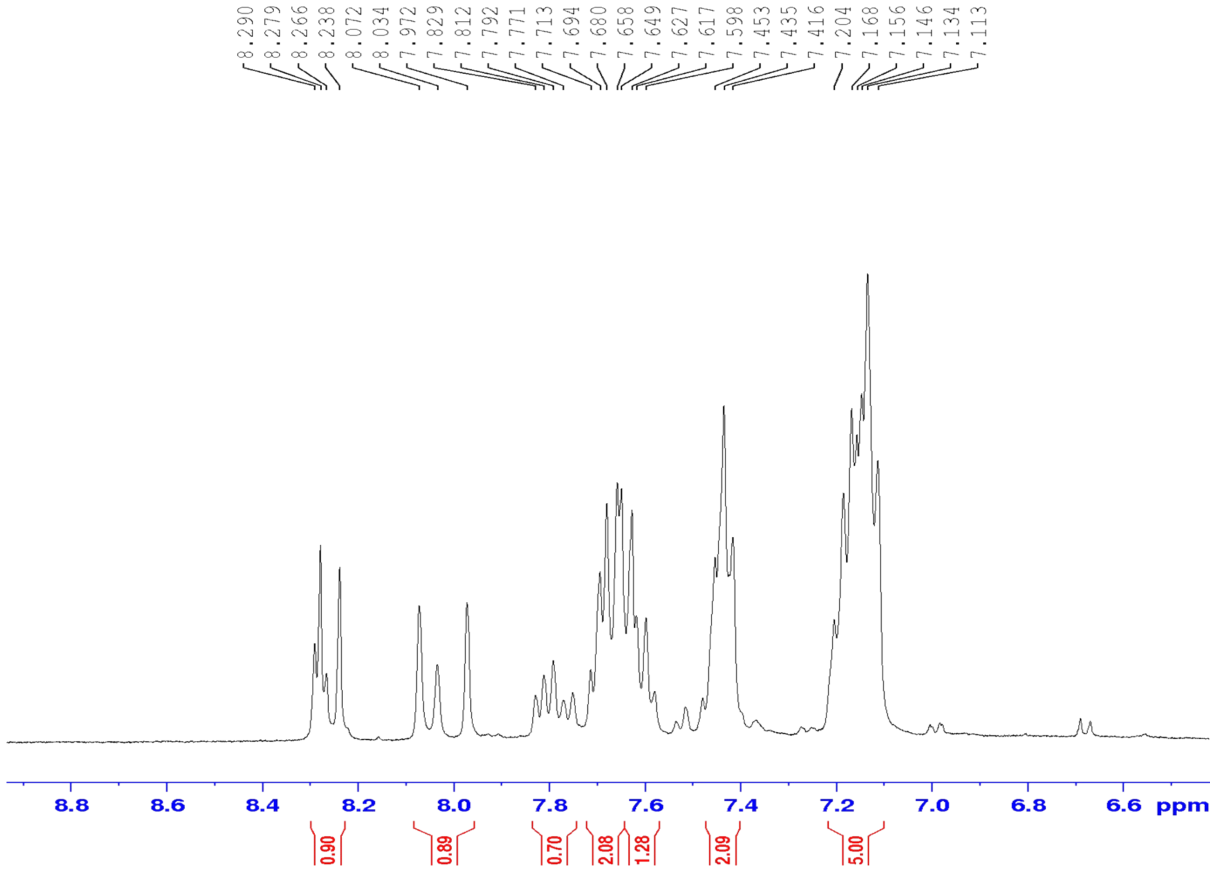

Figure 51. Aromatic region of the ^{1}H NMR spectrum for **12i**

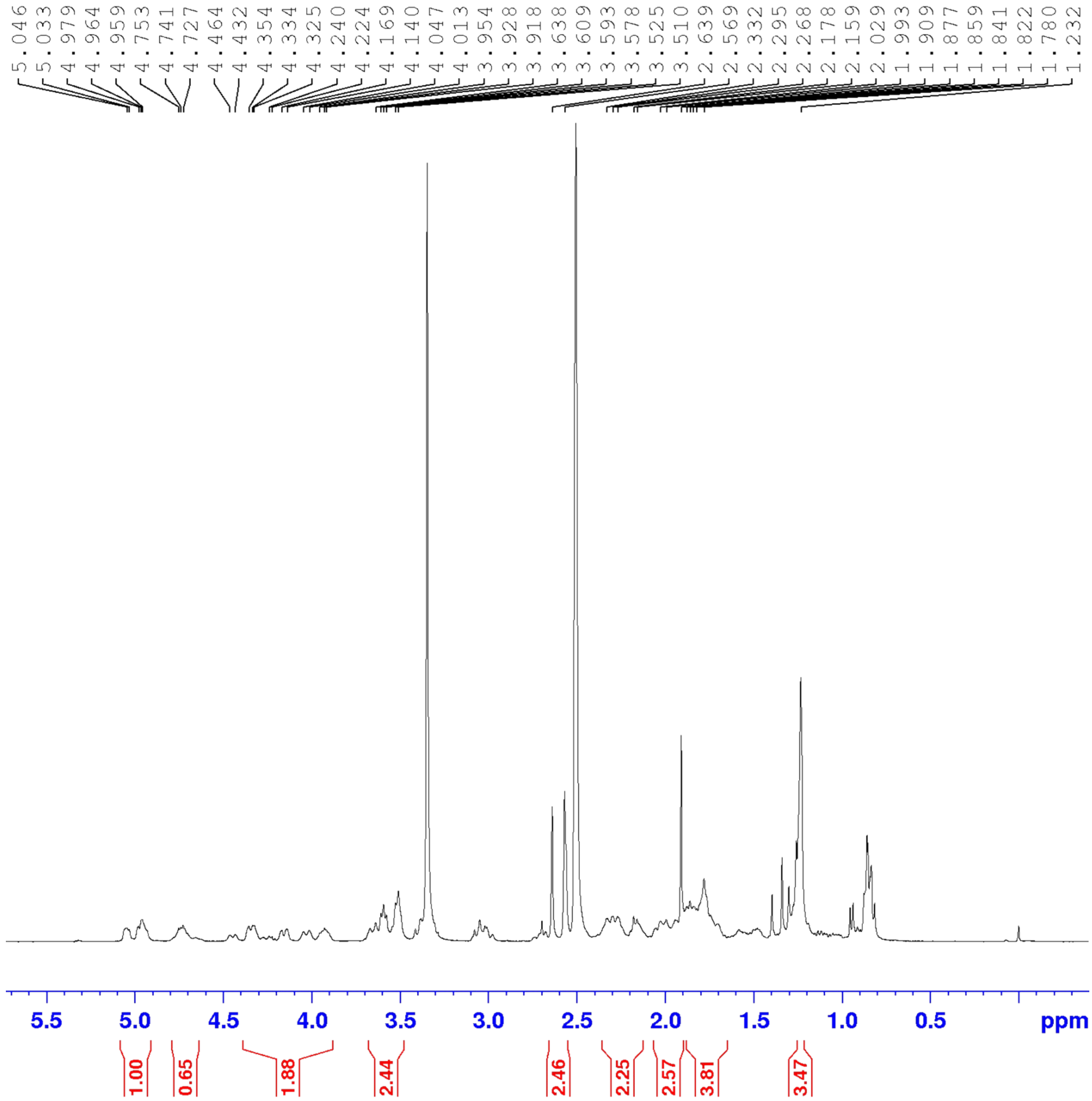

Figure 52. Aliphatic region of the ^{1}H NMR spectrum for **12i**

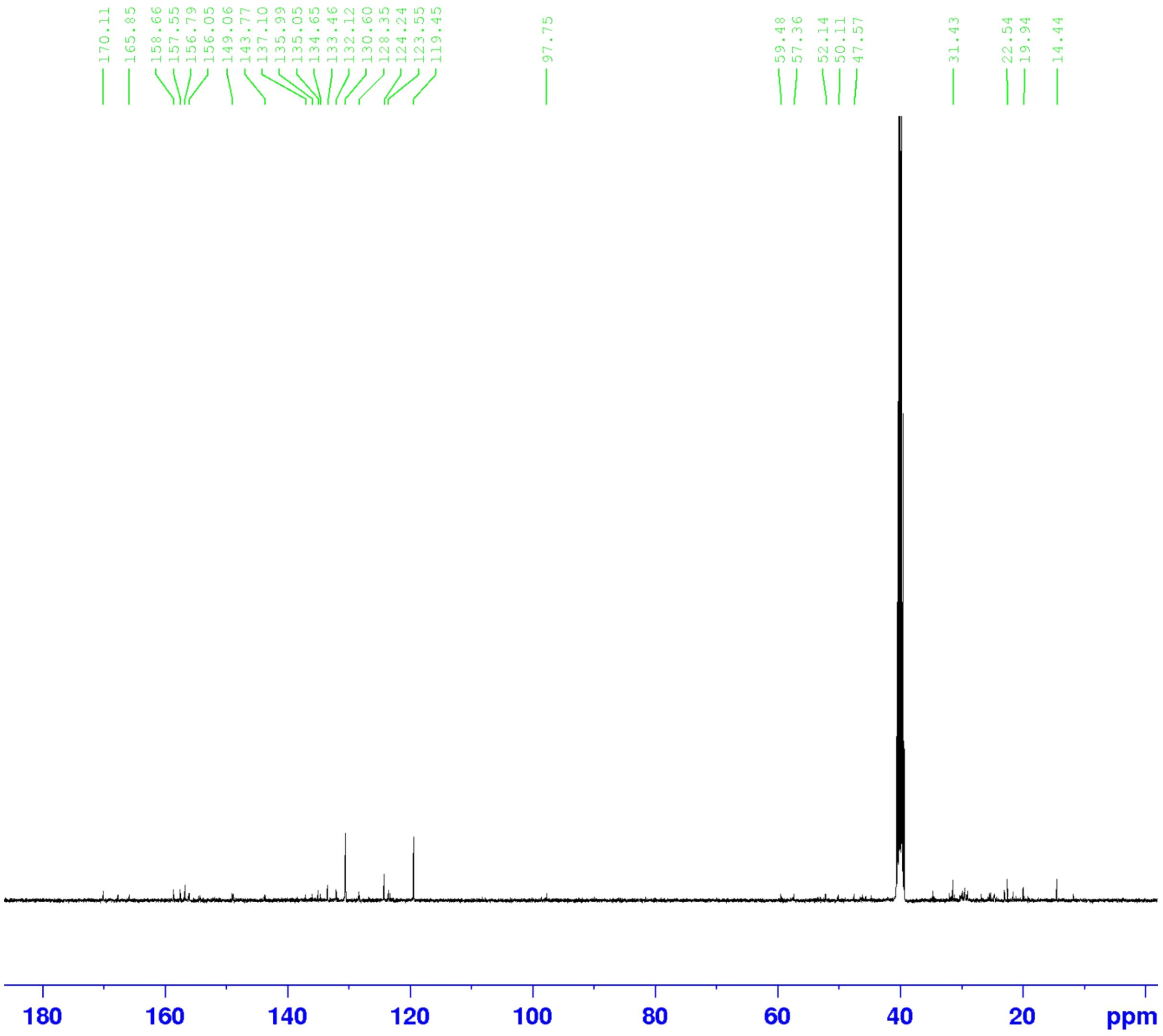

Figure 53. ^{13}C-NMR spectrum of **12i**

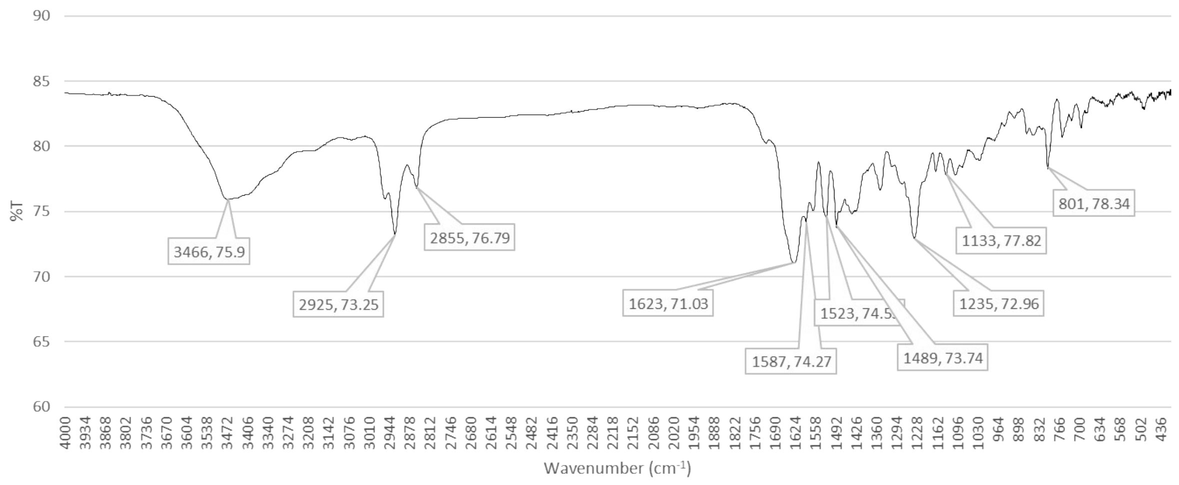

Figure 54. FT-IR spectrum of **12i**

Spectral data of ***4-amino-1-((3R)-1-((4-chlorobenzoyl)prolyl)piperidin-3-yl)-3-(4-phenoxyphenyl)-1H-pyrazolo[3,4-d]pyrimidine (12j)***

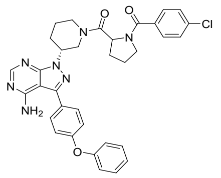

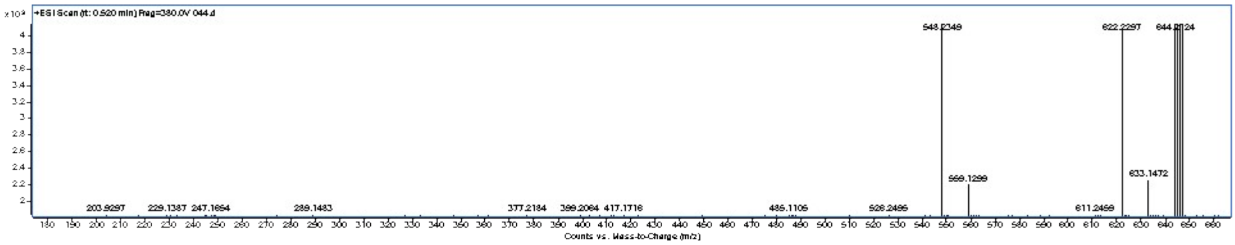

Figure 55. HRMS spectrum of **12j**

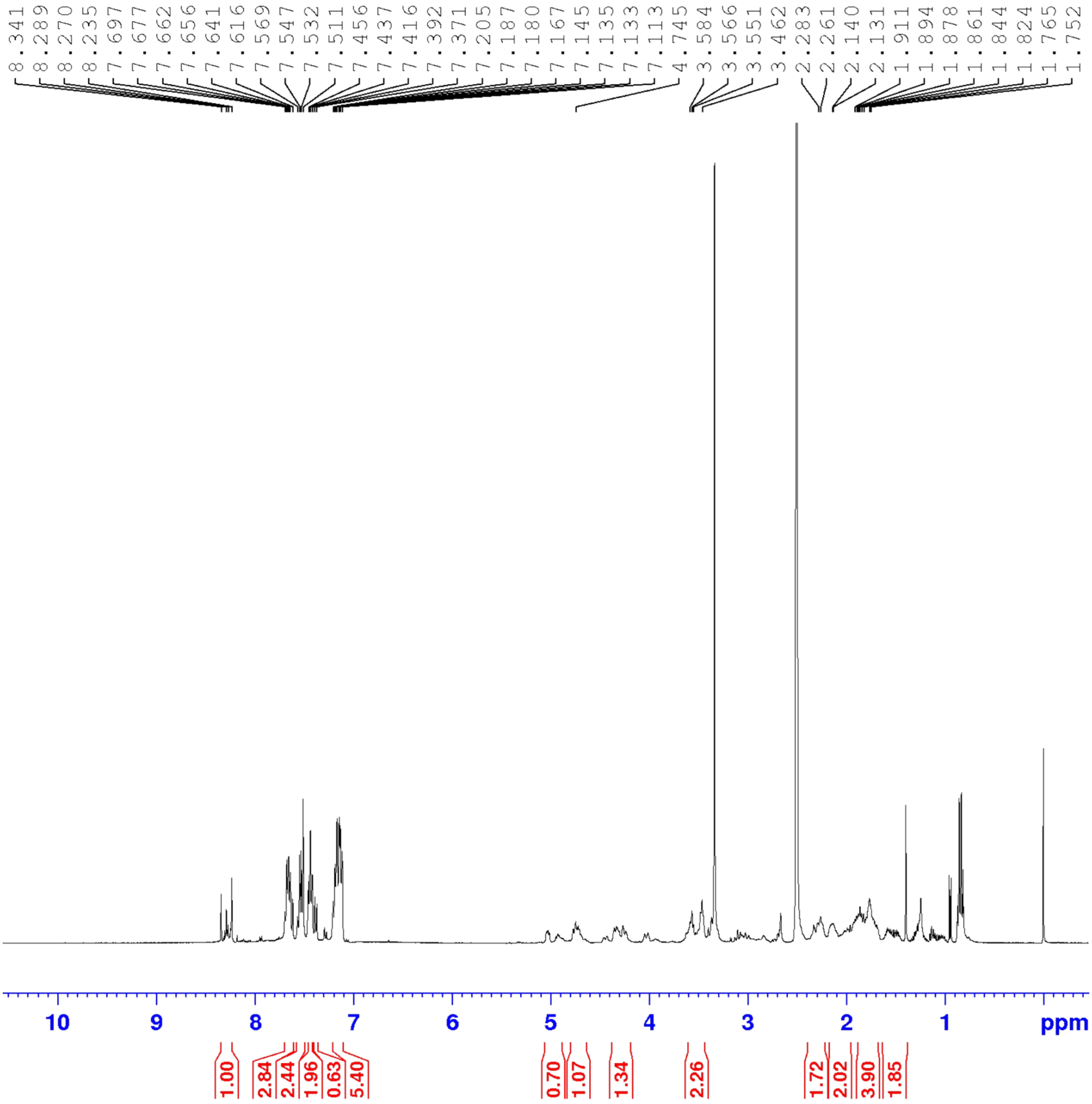

Figure 56. ^{1}H NMR full spectrum of **12j**

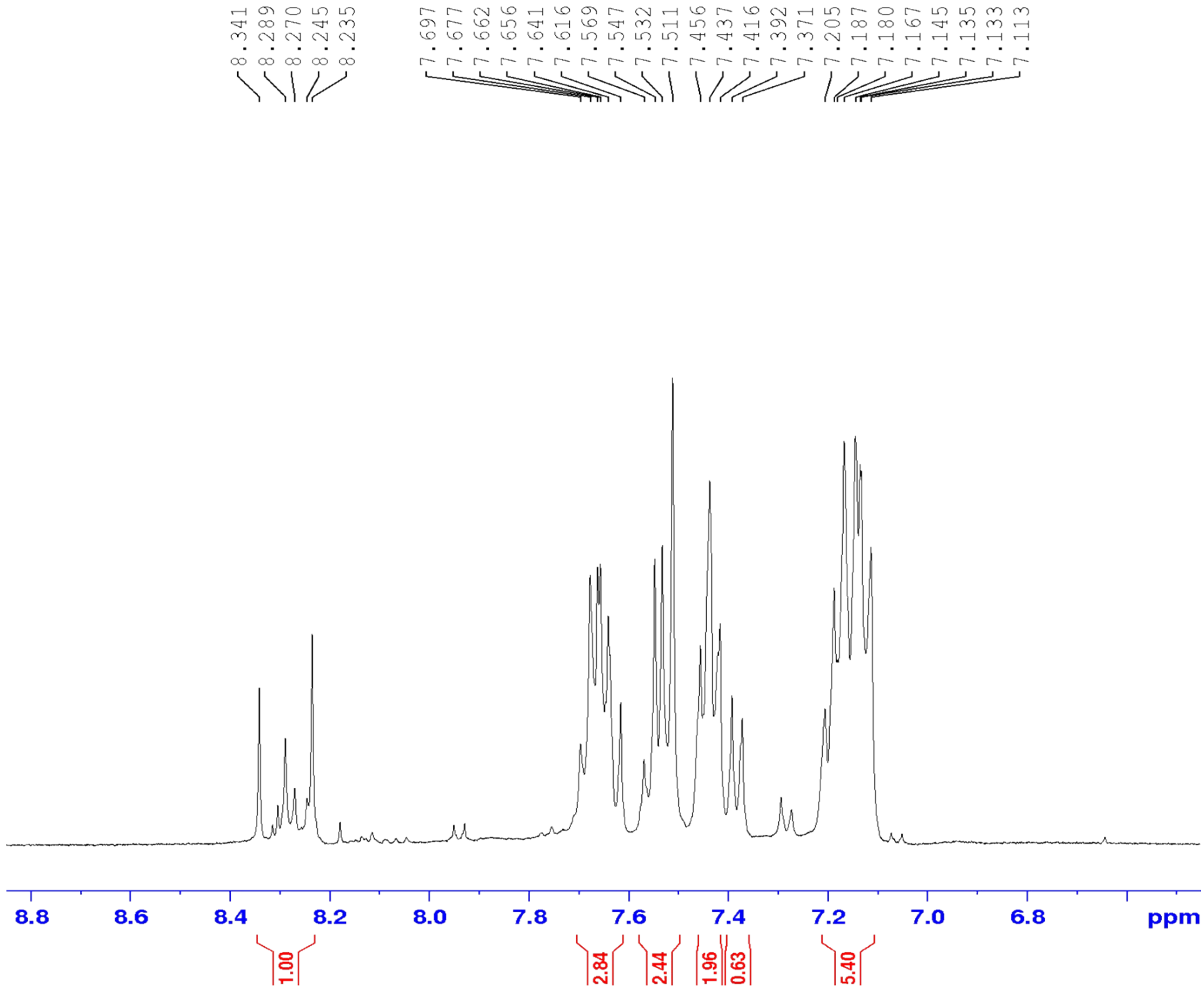

Figure 57. Aromatic region of the ^{1}H NMR spectrum for **12j**

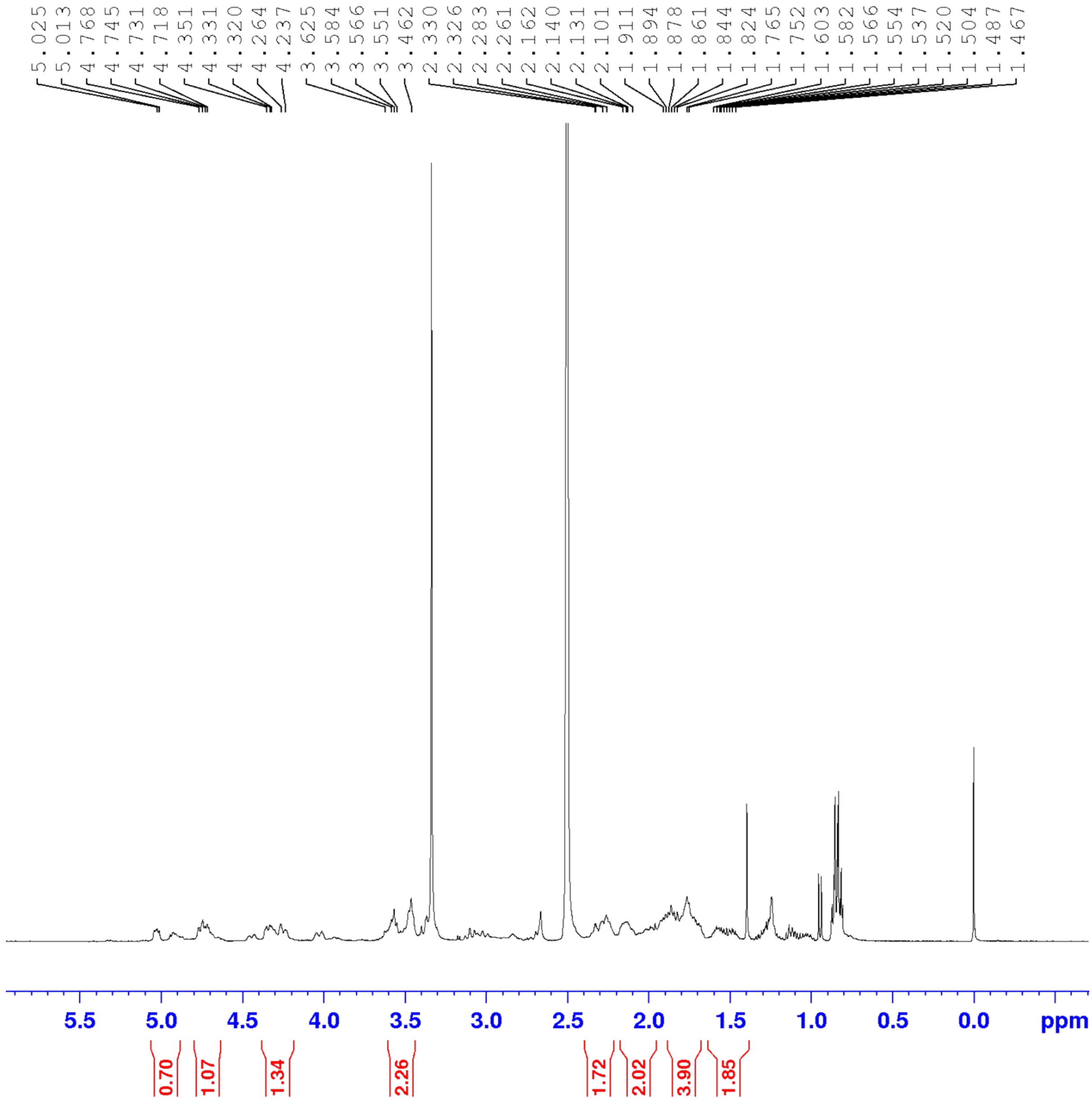

Figure 58. Aliphatic region of the ^{1}H NMR spectrum for **12j**

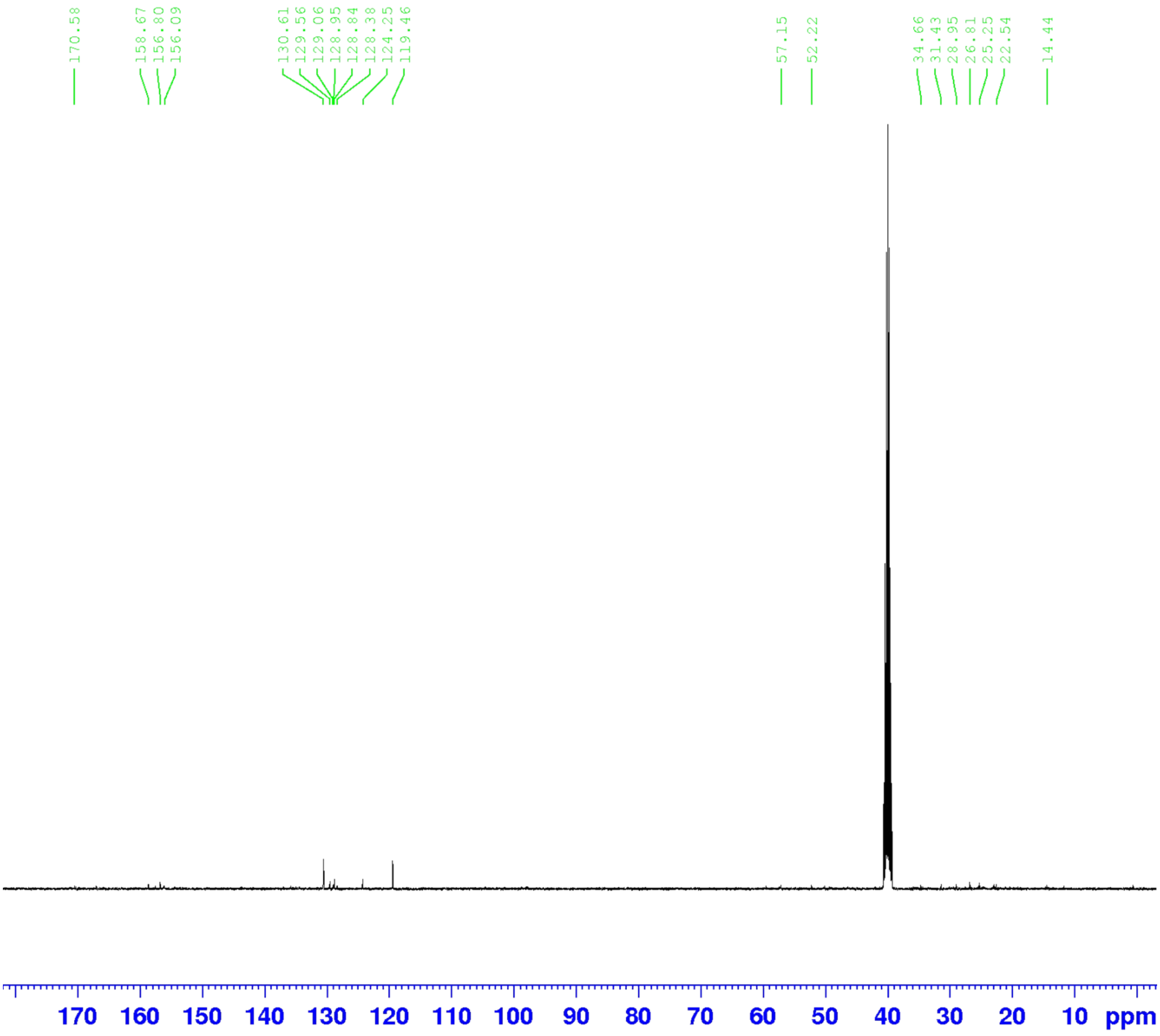

Figure 59. ^{13}C-NMR spectrum of **12j**

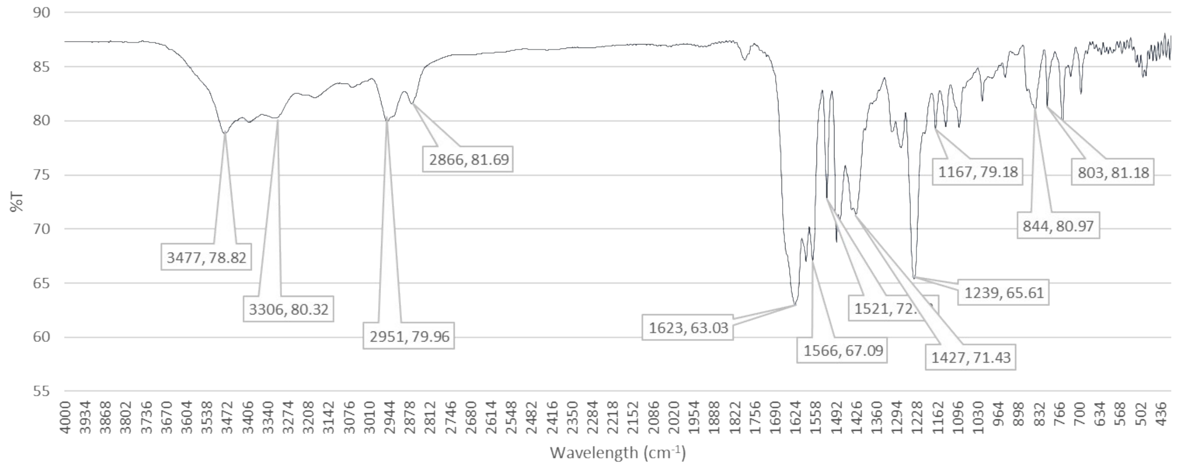

Figure 60. FT-IR spectrum of **12j**

Spectral data of ***4-amino-1-((3R)-1-((3-nitrobenzoyl)prolyl)piperidin-3-yl)-3-(4-phenoxyphenyl)-1H-pyrazolo[3,4-d]pyrimidine (12k)***

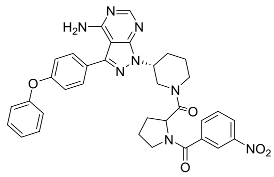

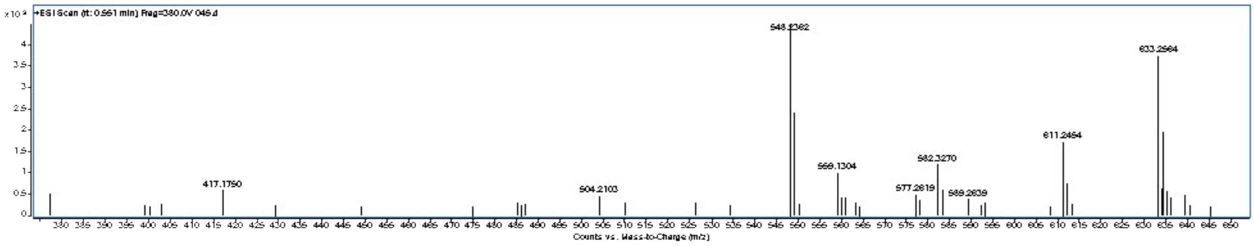

Figure 61. HRMS spectrum of **12k**

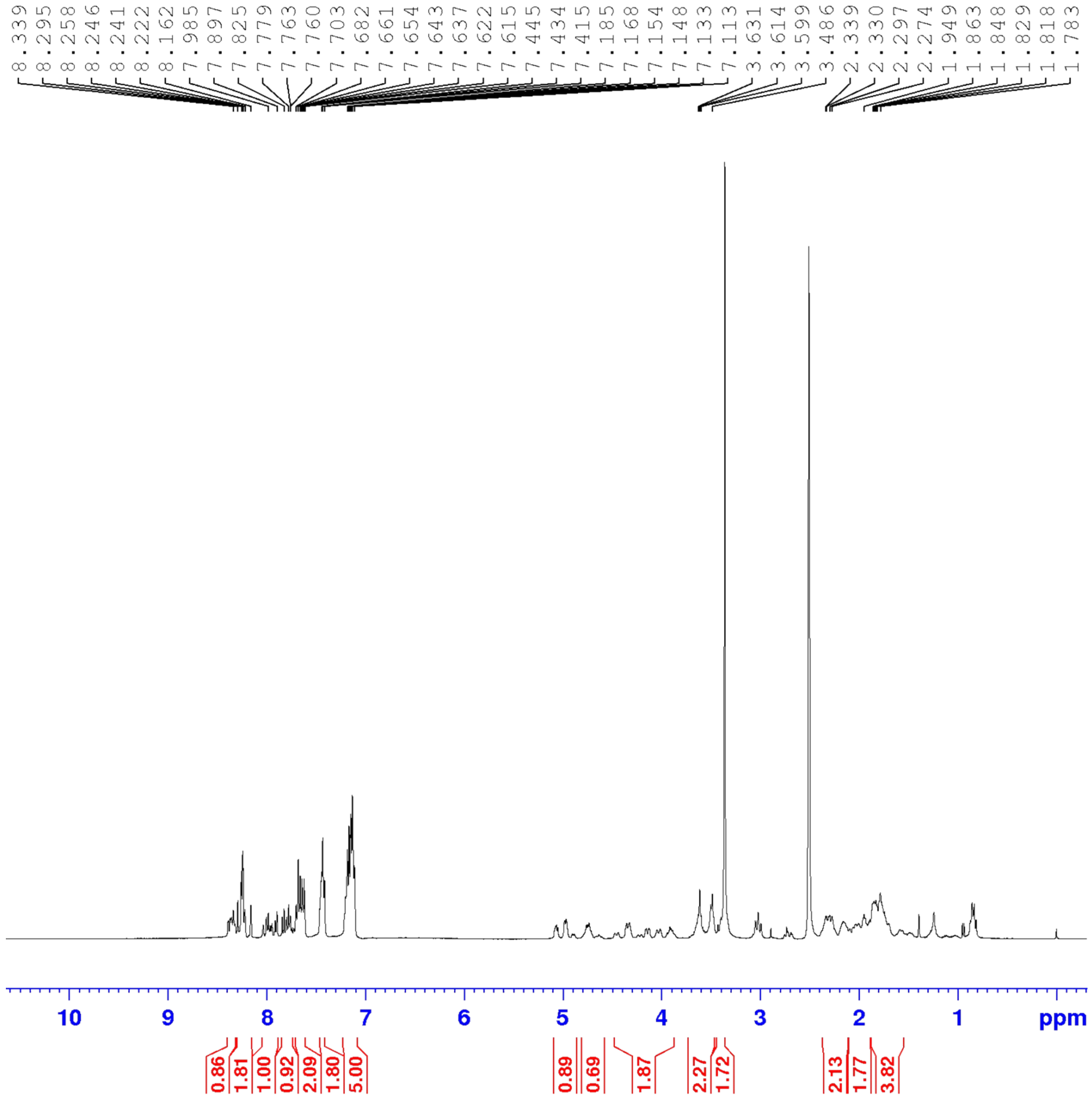

Figure 62. ^{1}H NMR full spectrum of **12k**

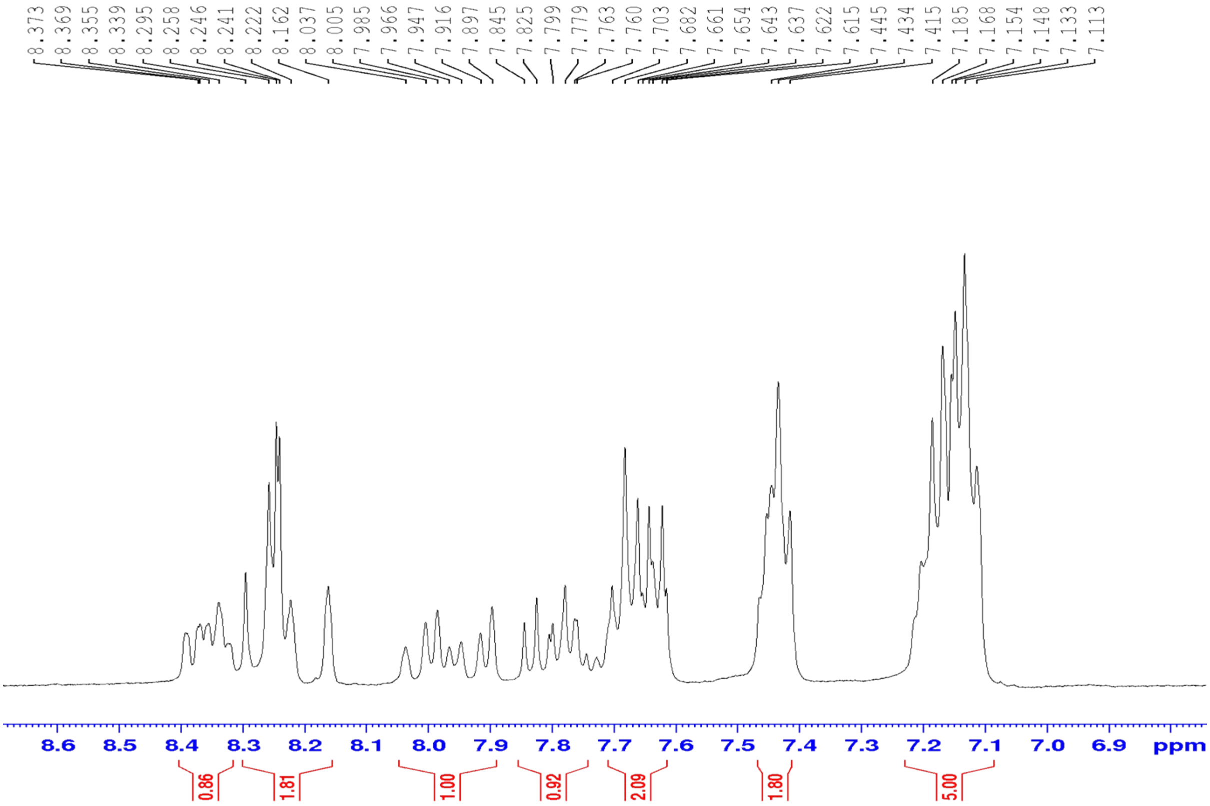

Figure 63. Aromatic region of the ^{1}H NMR spectrum for **12k**

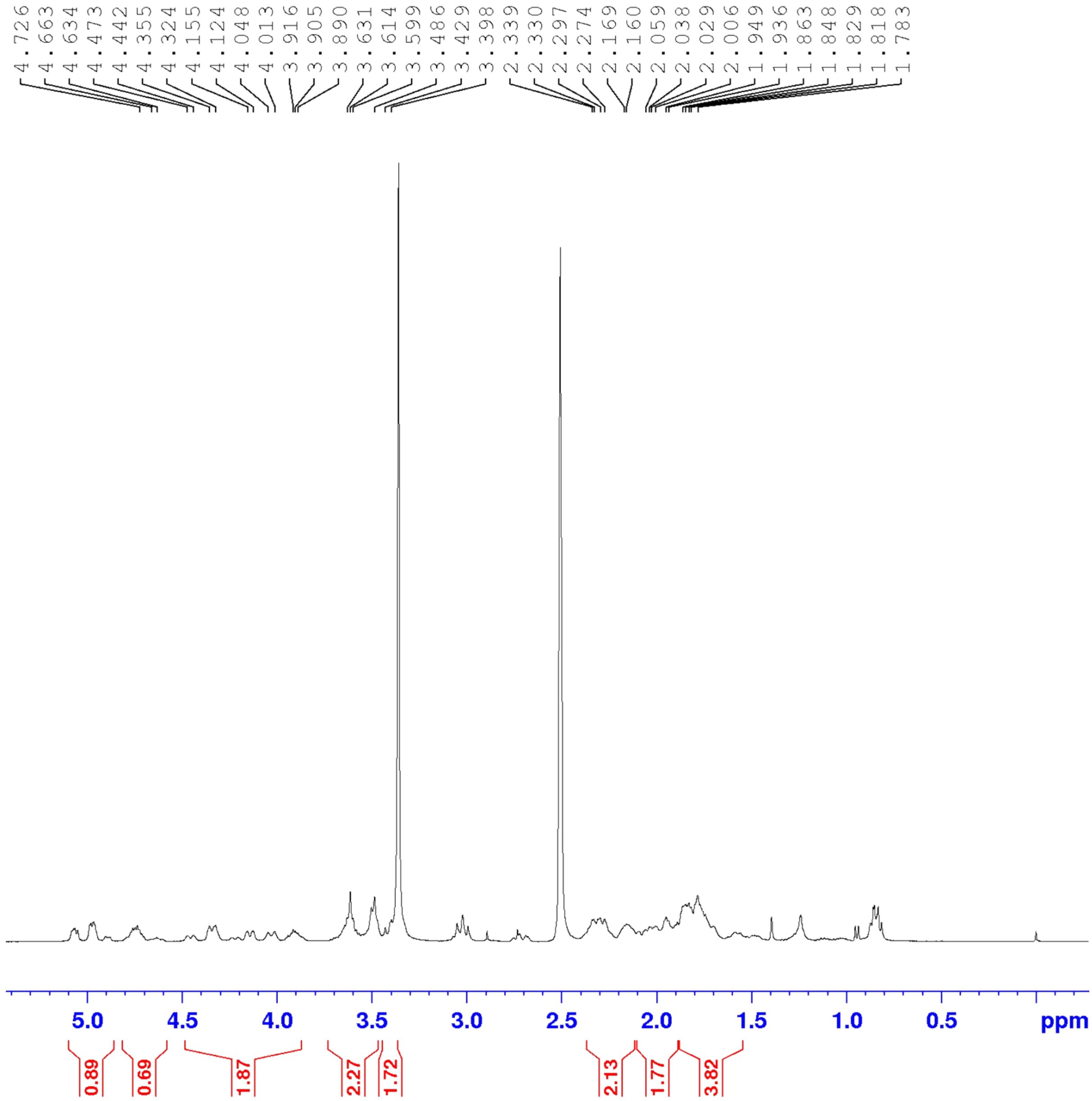

Figure 64. Aliphatic region of the ^{1}H NMR spectrum for **12k**

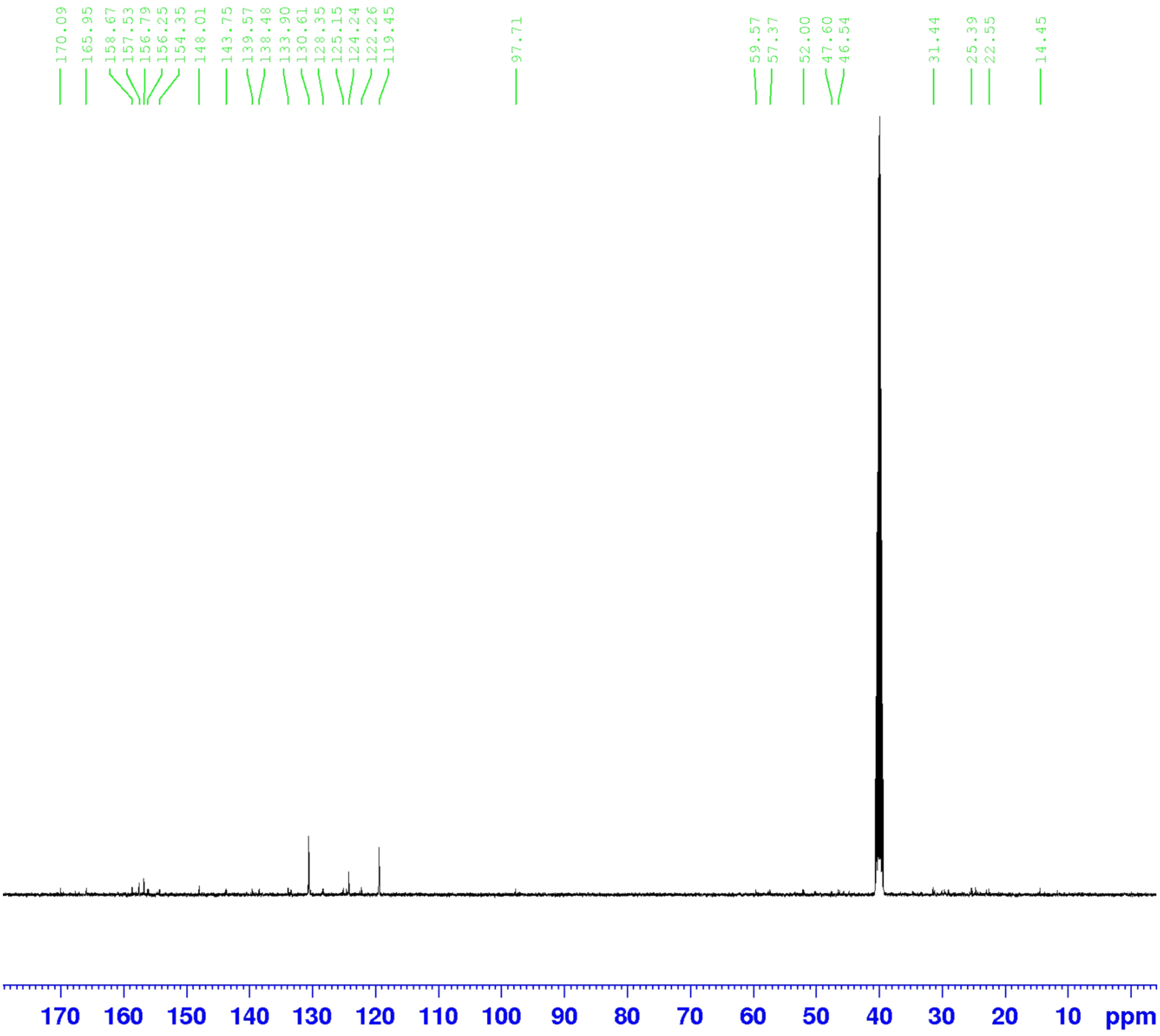

Figure 65. ^{13}C-NMR spectrum of **12k**

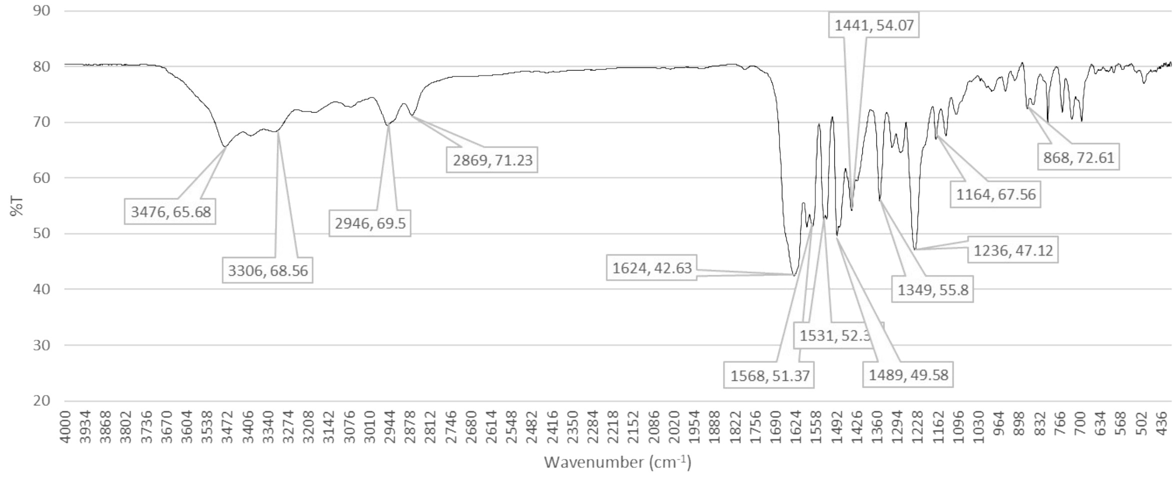

Figure 66. FT-IR spectrum of **12k**

Spectral data of ***4-(2-((R)-3-(4-amino-3-(4-phenoxyphenyl)-1H-pyrazolo[3,4-d]pyrimidin-1-yl)piperidine-1-carbonyl)pyrrolidine-1-carbonyl)phenyl acetate (12l)***

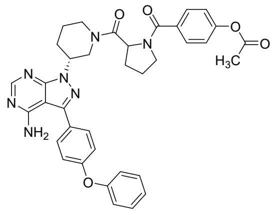

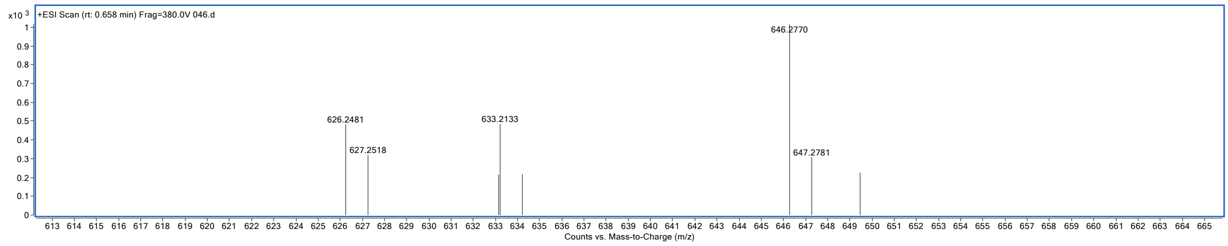

Figure 67. HRMS spectrum of **12l**

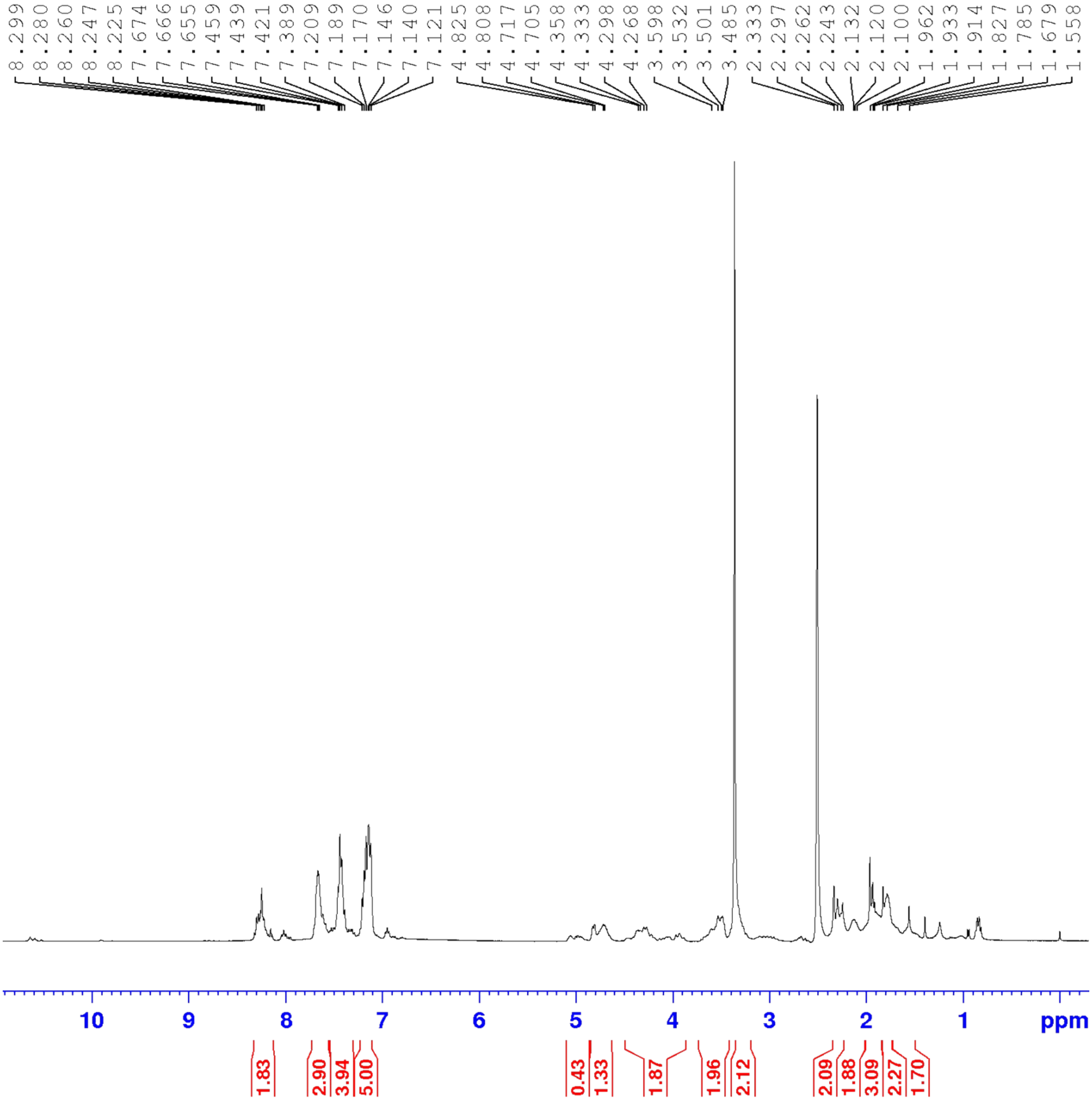

Figure 68. ^{1}H NMR full spectrum of **12l**

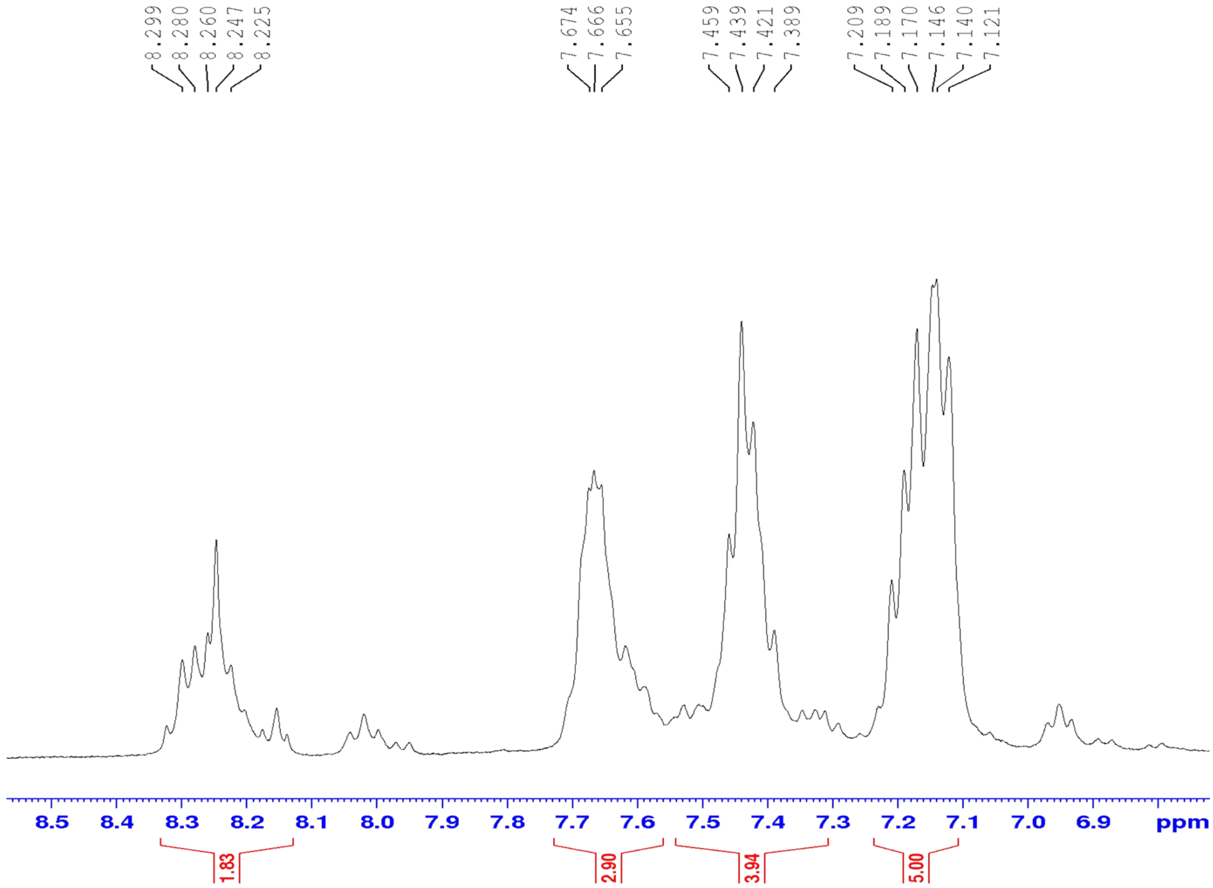

Figure 69. Aromatic region of the ^{1}H NMR spectrum for **12l**

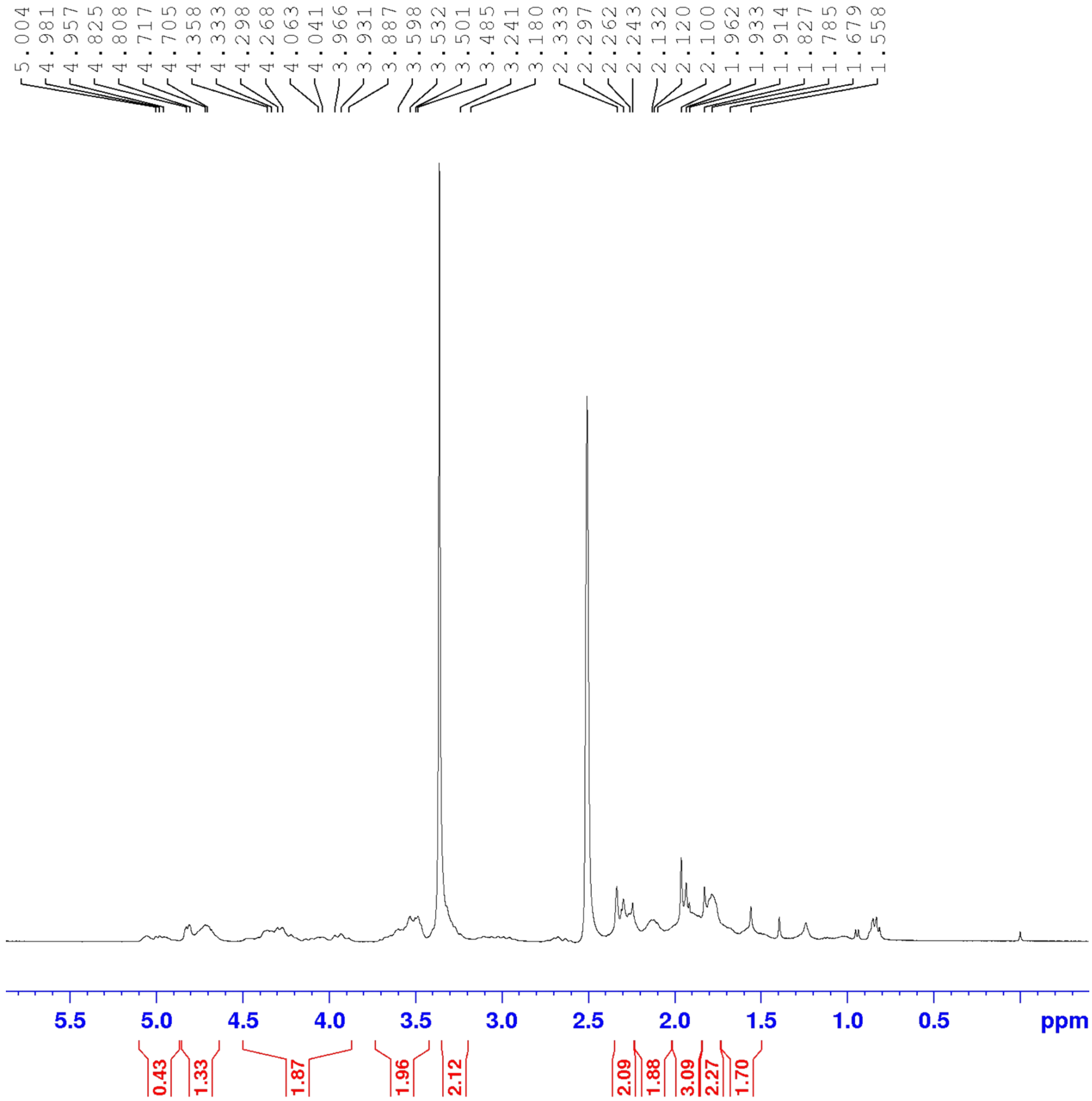

Figure 70. Aliphatic region of the ^{1}H NMR spectrum for **12l**

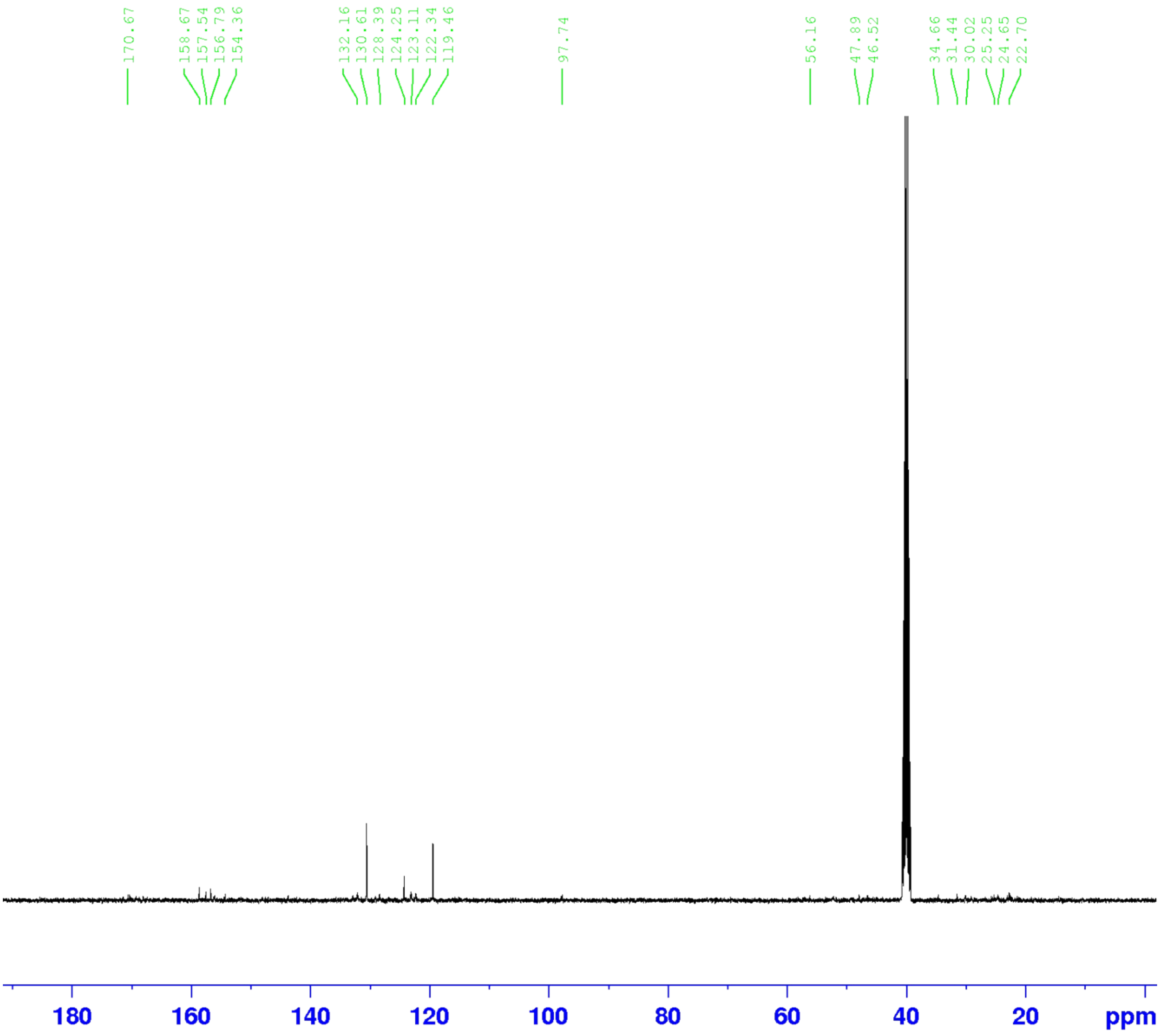

Figure 71. ^{13}C-NMR spectrum of **12l**

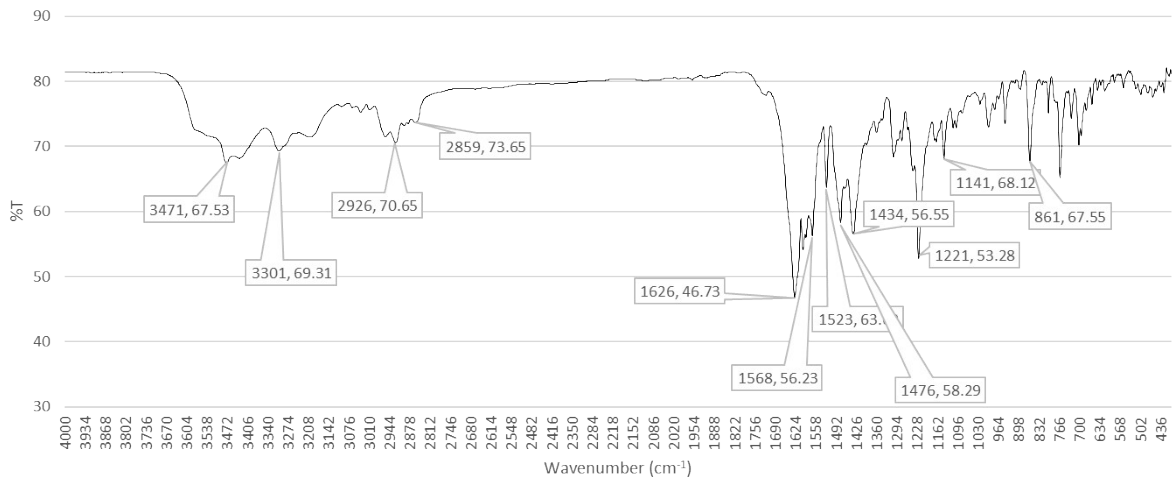

Figure 72. FT-IR spectrum of **12l**

Spectral data of ***4-amino-1-((3R)-1-((4-bromo-3-(trifluoromethyl)benzoyl)prolyl)piperidin-3-yl)-3-(4-phenoxyphenyl)-1H-pyrazolo[3,4-d]pyrimidine (12m)***

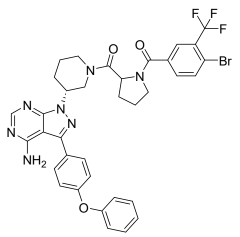

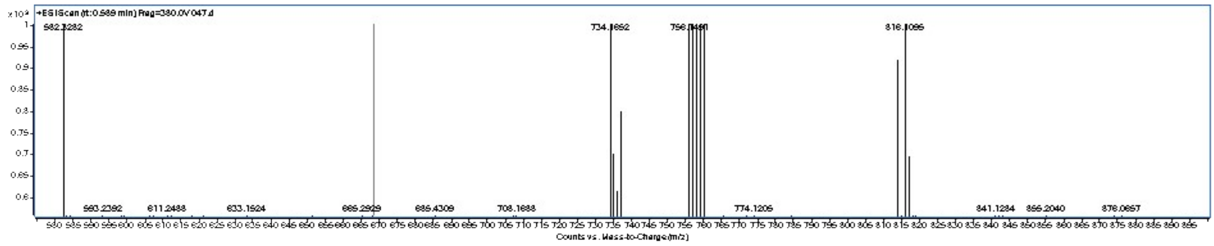

Figure 73. HRMS spectrum of **12m**

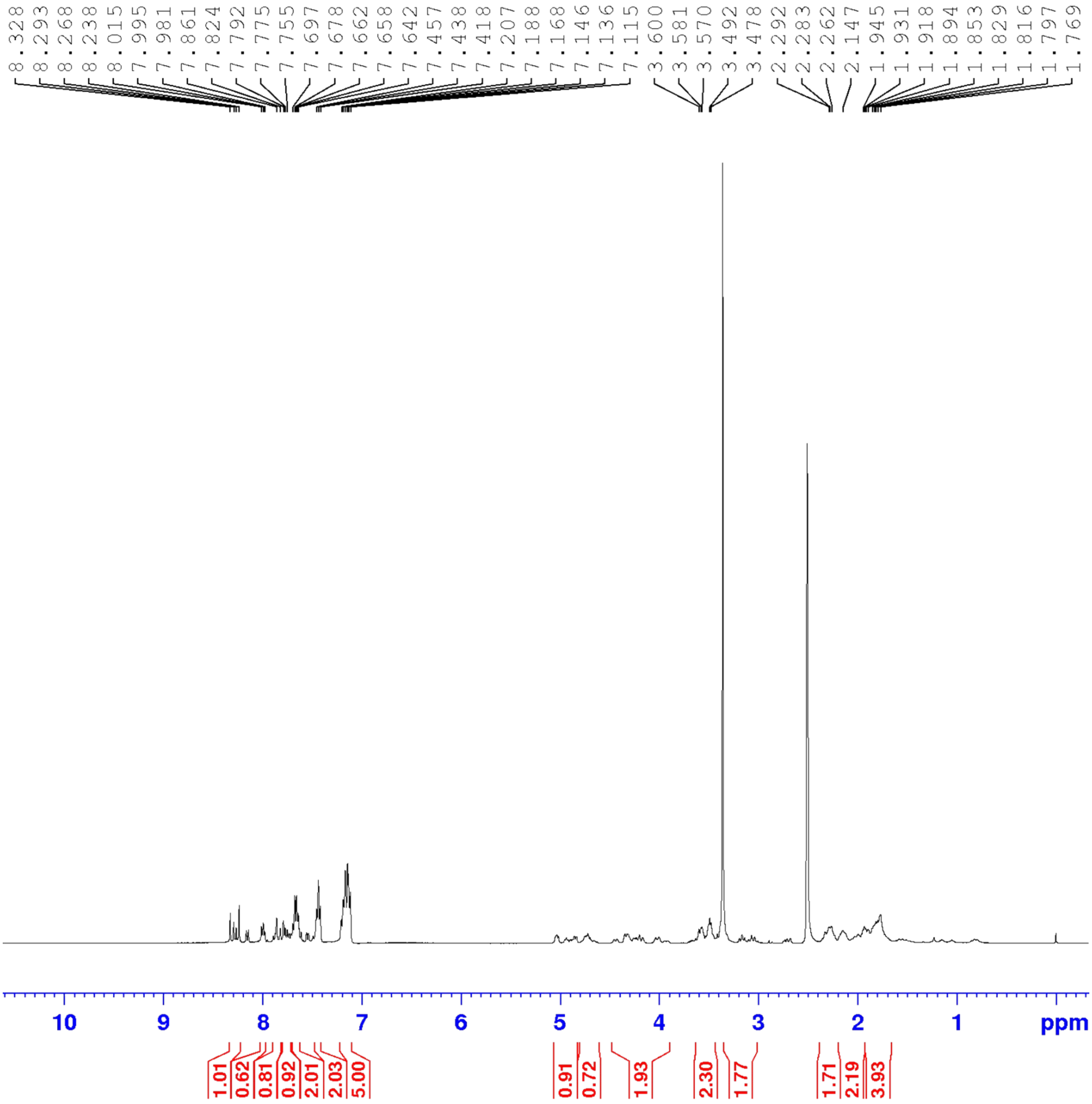

Figure 74. ^{1}H NMR full spectrum of **12m**

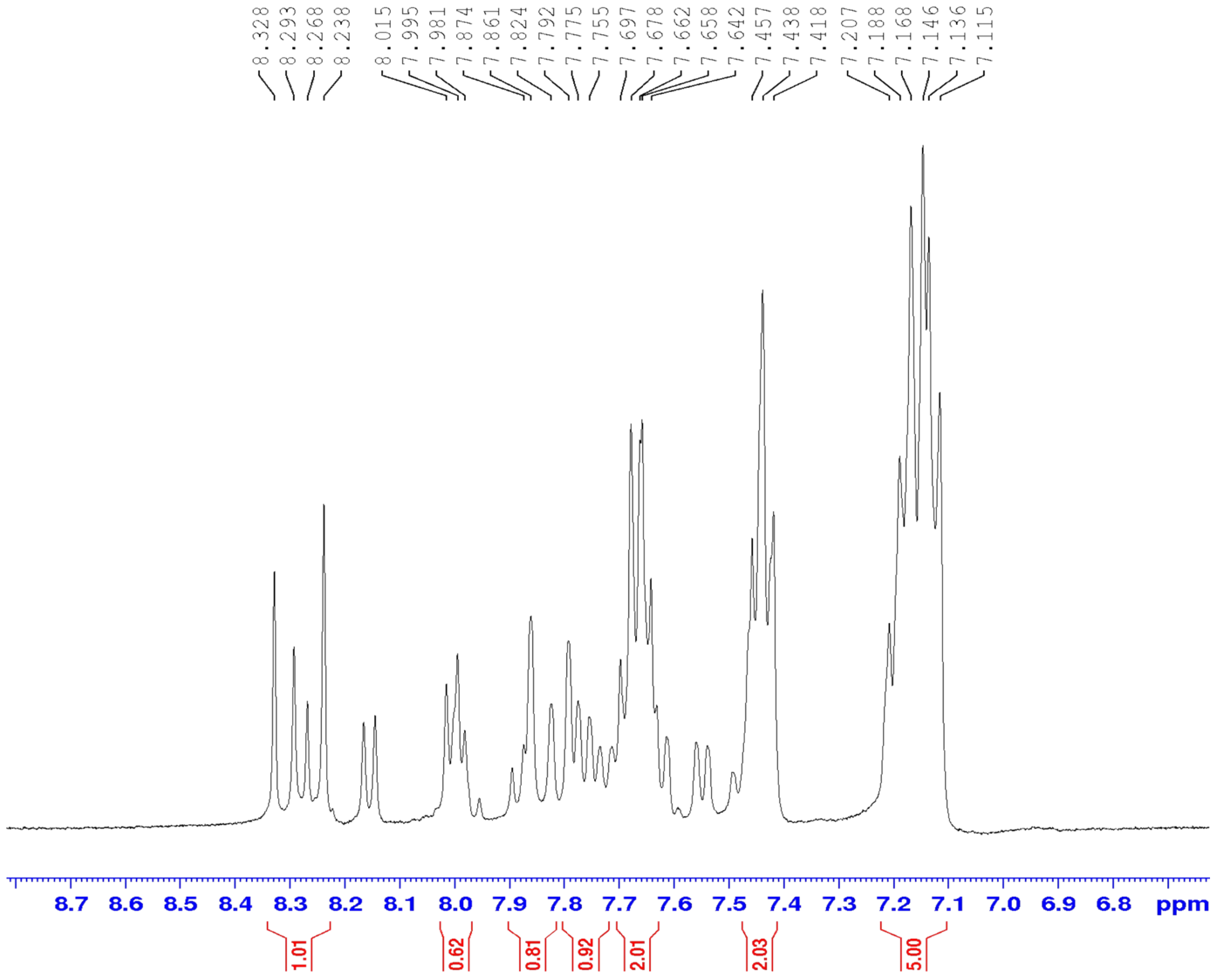

Figure 75. Aromatic region of the ^{1}H NMR spectrum for **12m**

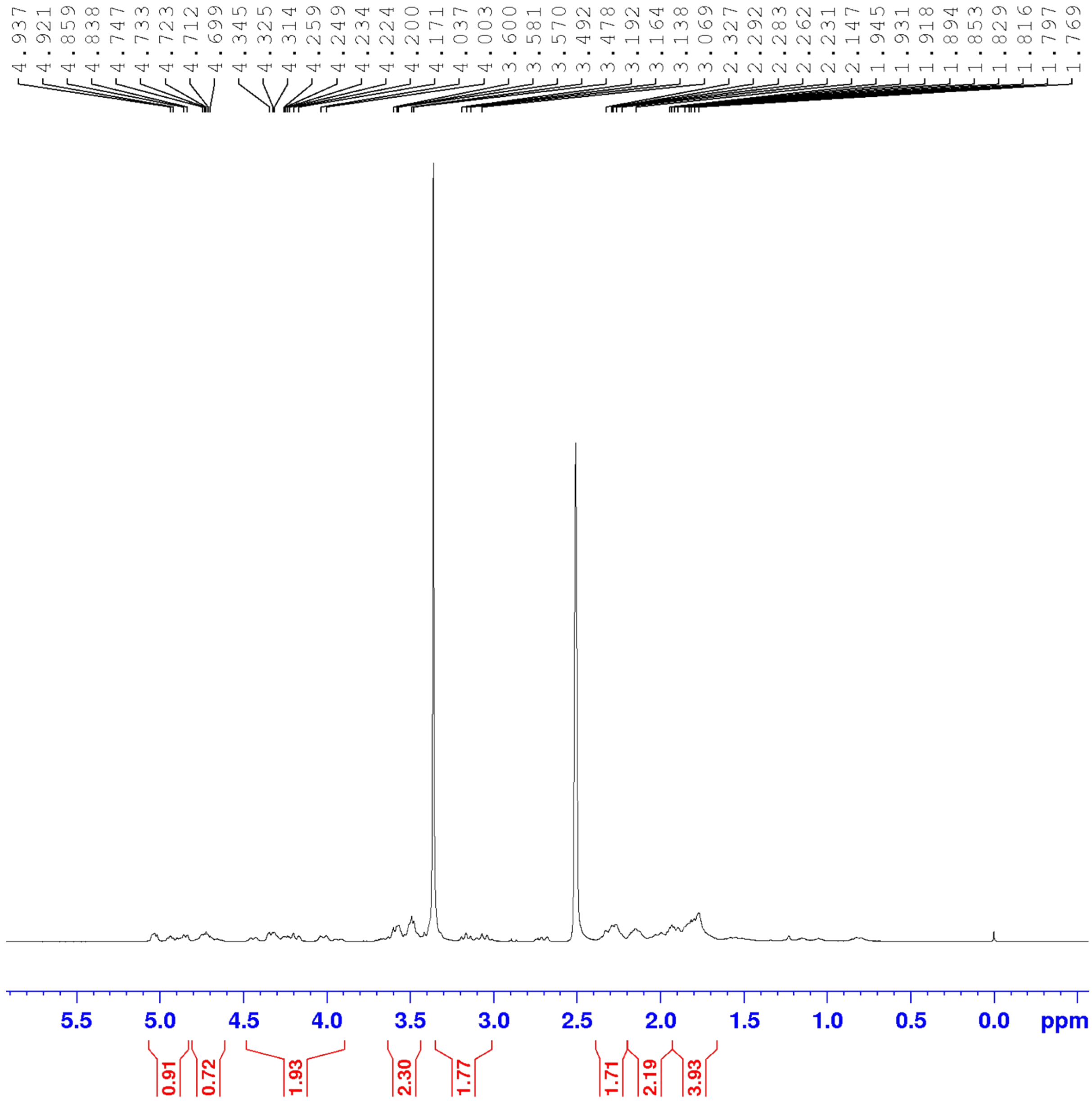

Figure 76. Aliphatic region of the ^{1}H NMR spectrum for **12m**

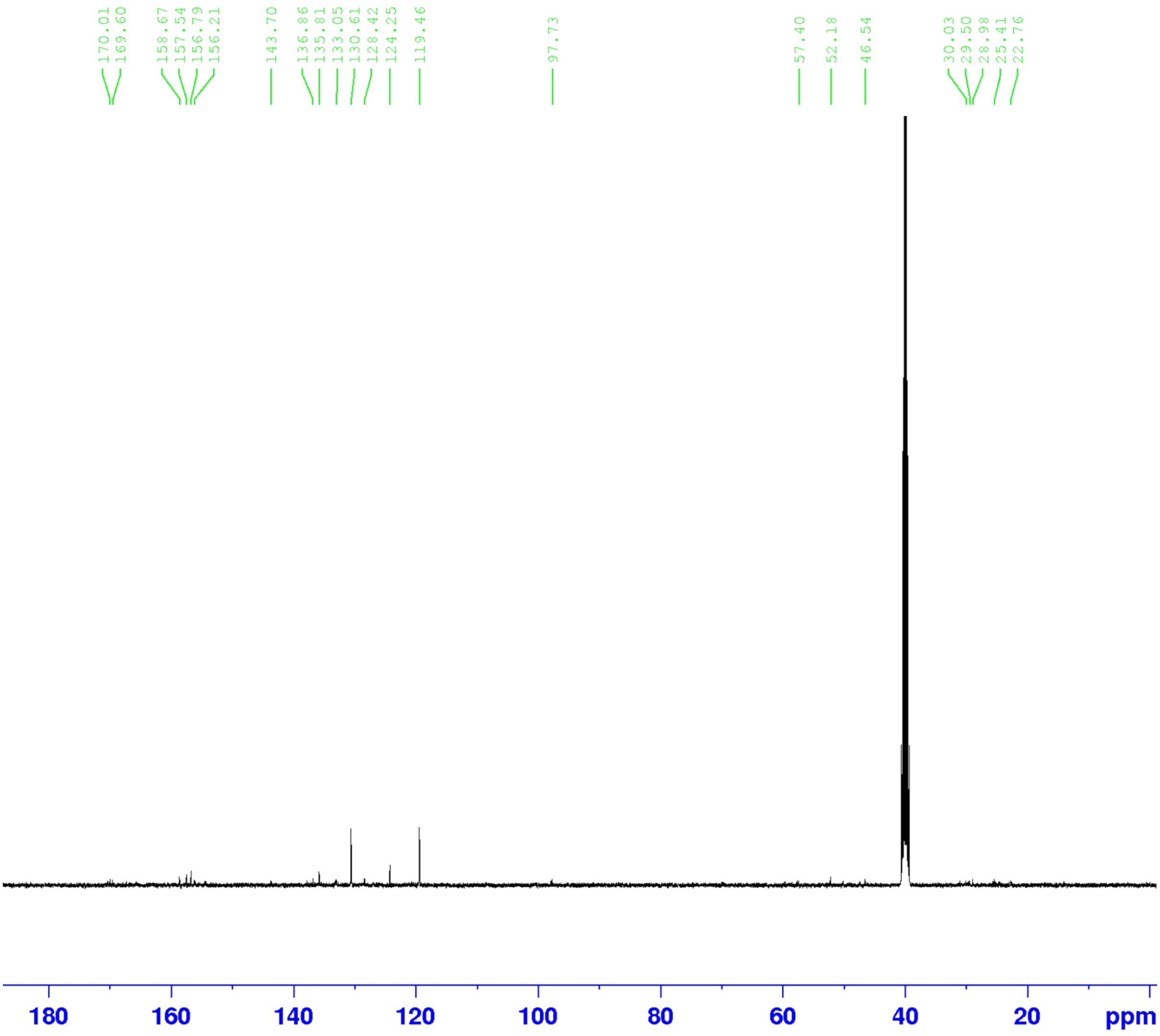

Figure 77. ^{13}C-NMR spectrum of **12m**

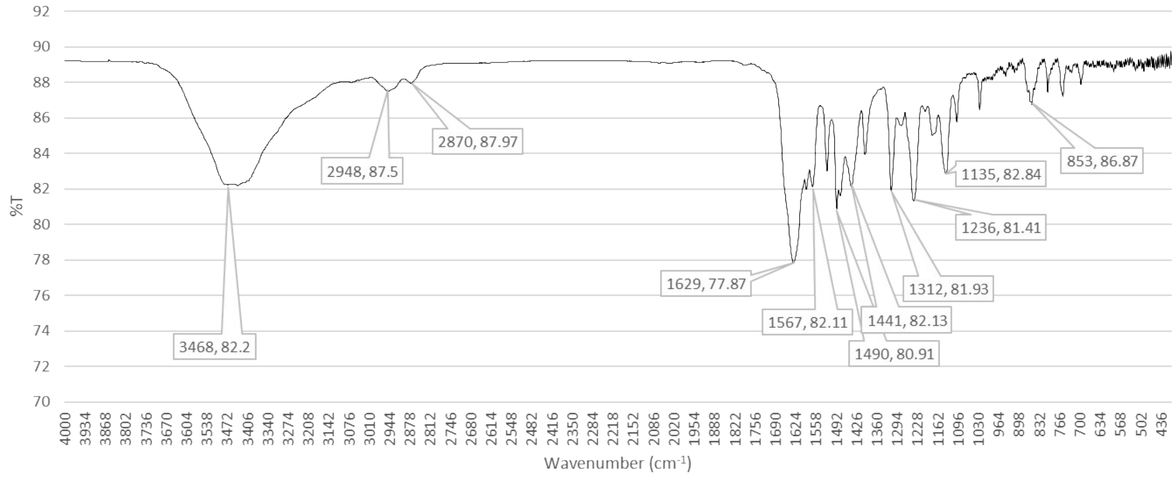

Figure 78. FT-IR spectrum of **12m**

Spectral data of ***1-(2-((R)-3-(4-amino-3-(4-phenoxyphenyl)-1H-pyrazolo[3,4-d]pyrimidin-1-yl)piperidine-1-carbonyl)pyrrolidin-1-yl)-2-(4-fluorophenyl)ethan-1-one (12n)***

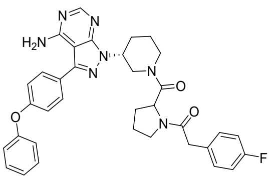

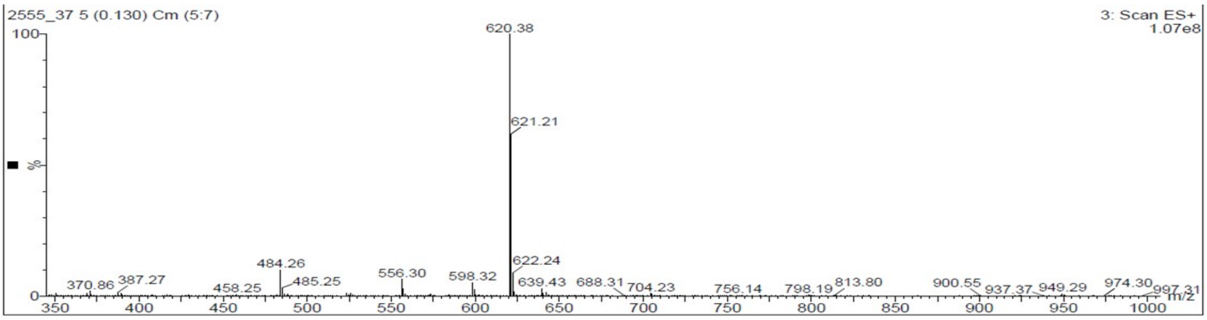

Figure 79. ESI-MS spectrum of **12n**

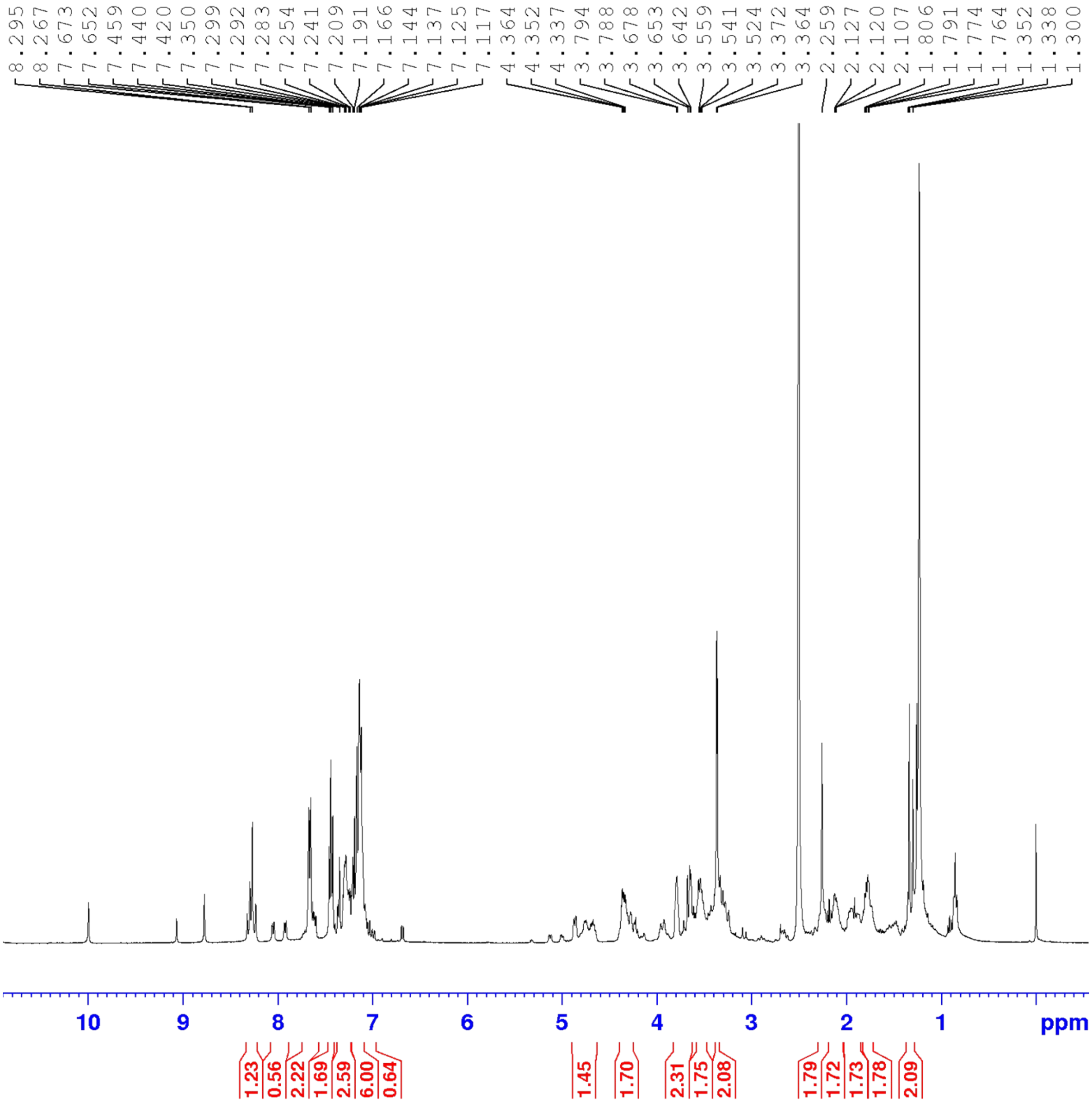

Figure 80. ^{1}H NMR full spectrum of **12n**

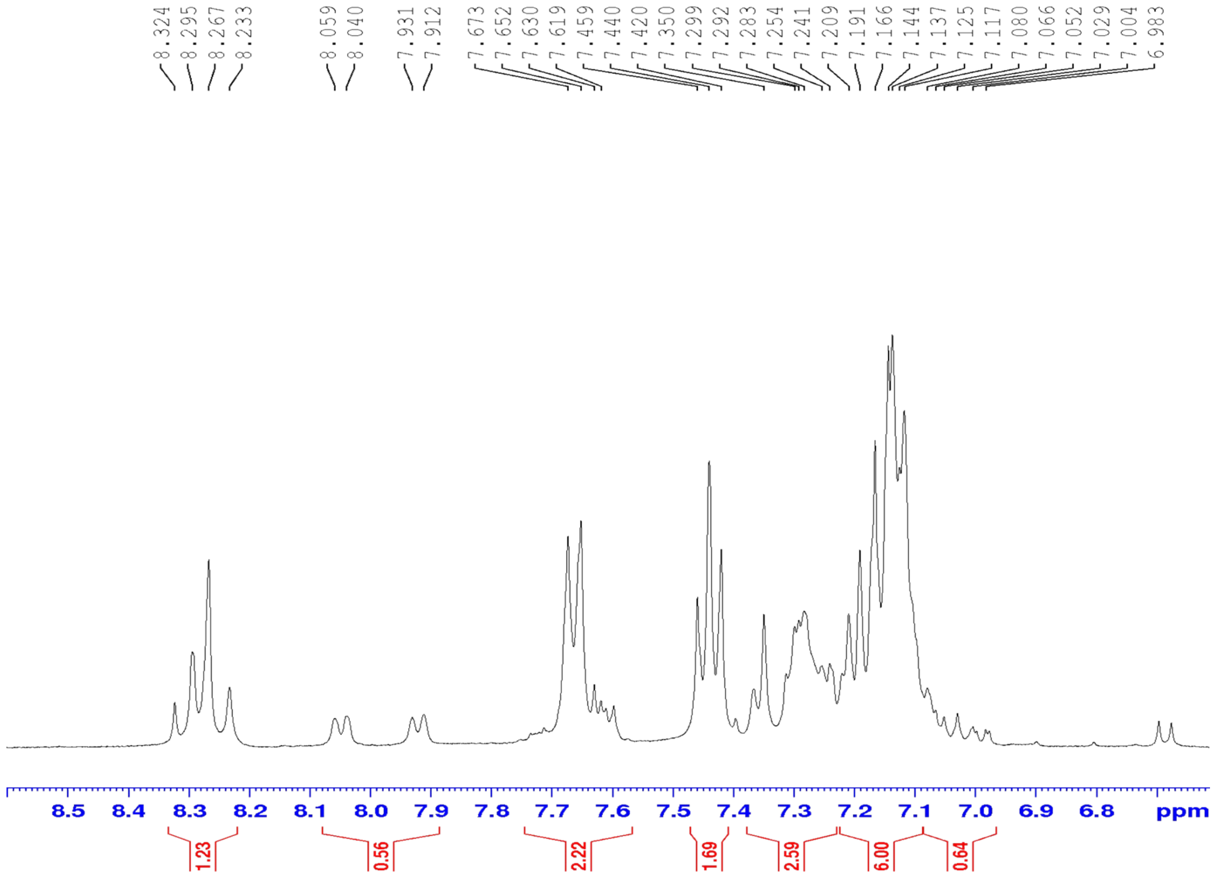

Figure 81. Aromatic region of the ^{1}H NMR spectrum for **12n**

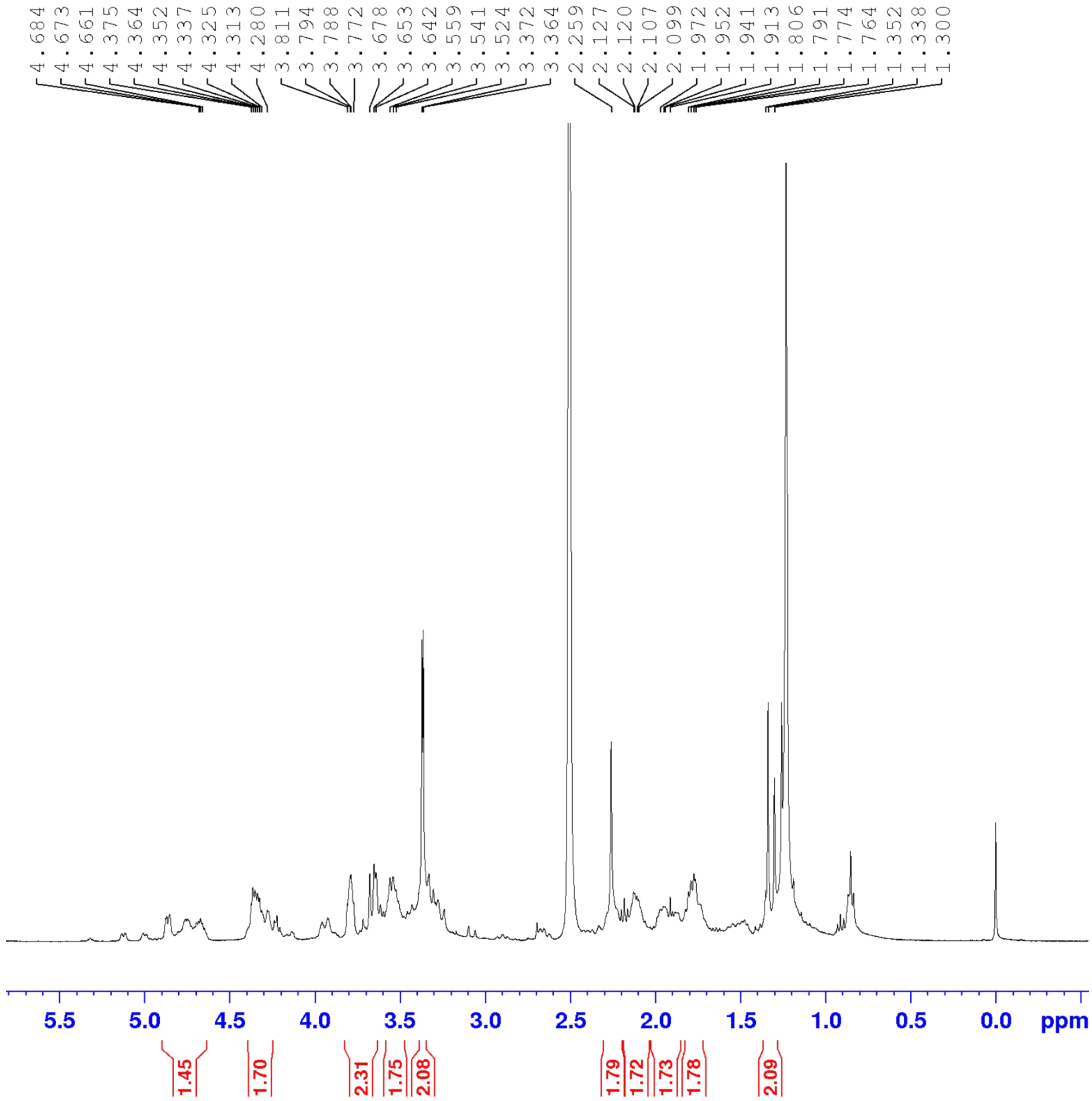

Figure 82. Aliphatic region of the ^{1}H NMR spectrum for **12n**

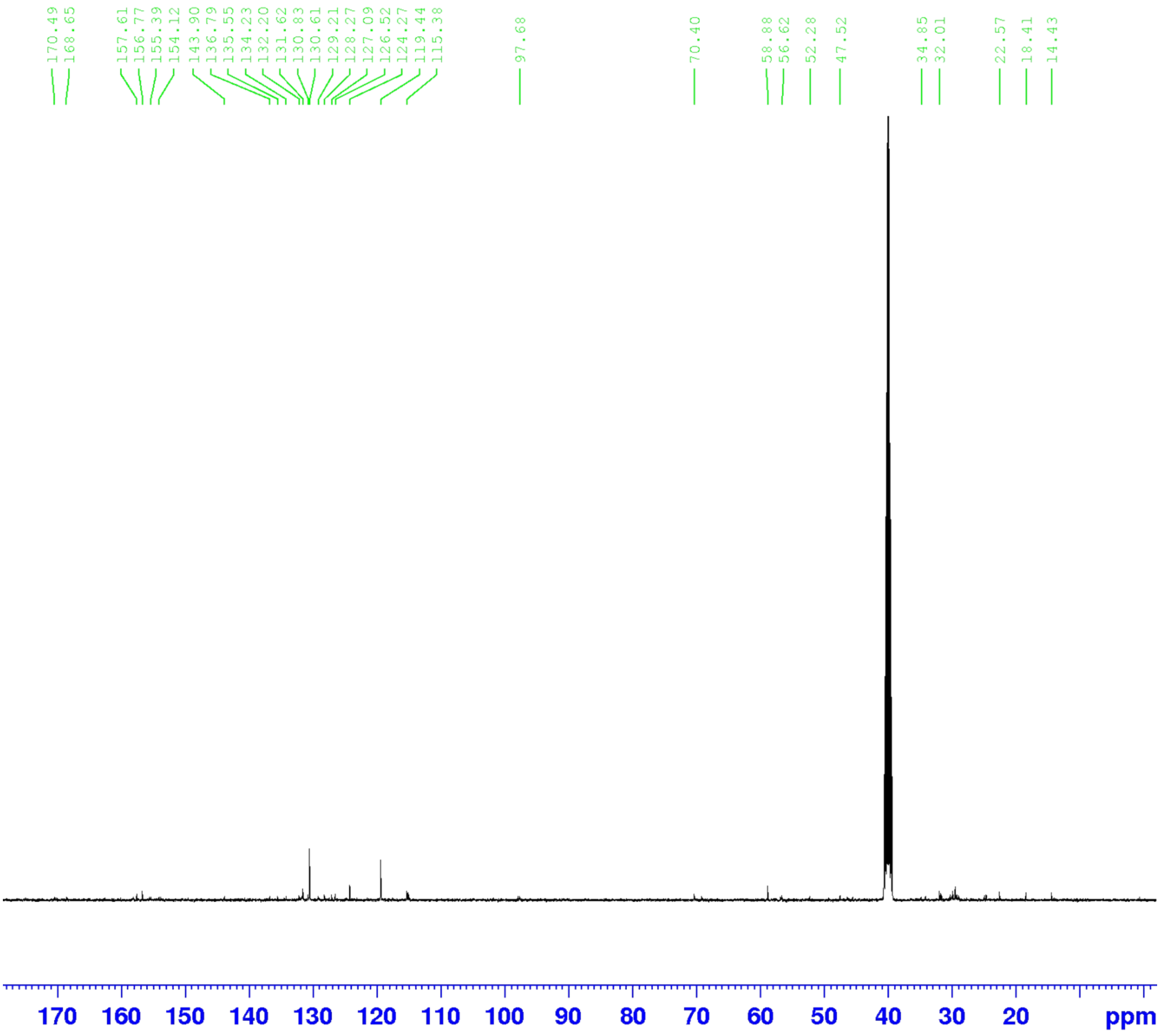

Figure 83. ^{13}C-NMR spectrum of **12n**

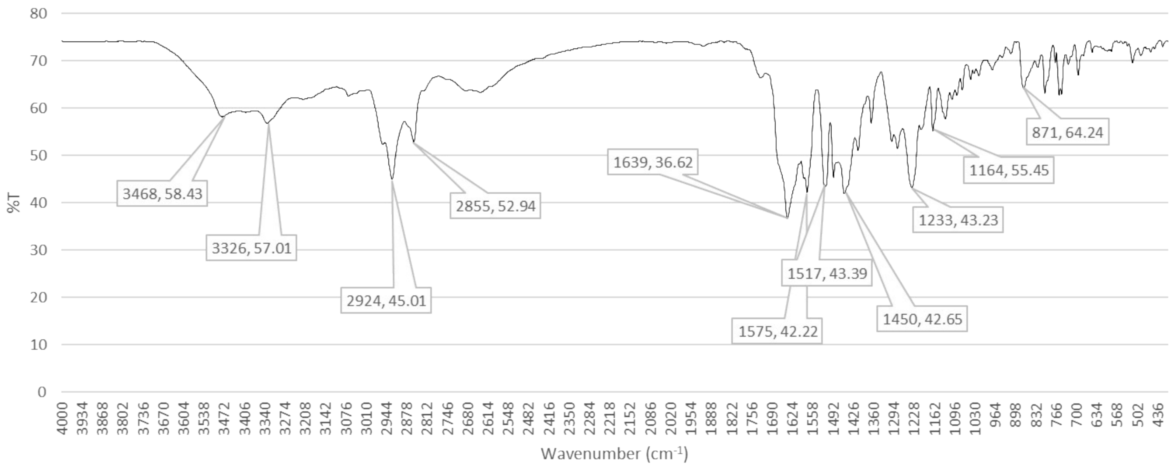

Figure 84. FT-IR spectrum of **12n**

Spectral data of ***4-amino-1-((3R)-1-((3-methylbenzoyl)prolyl)piperidin-3-yl)-3-(4-phenoxyphenyl)-1H-pyrazolo[3,4-d]pyrimidine (12o)***

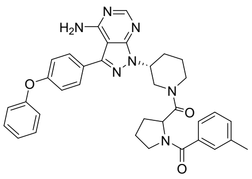

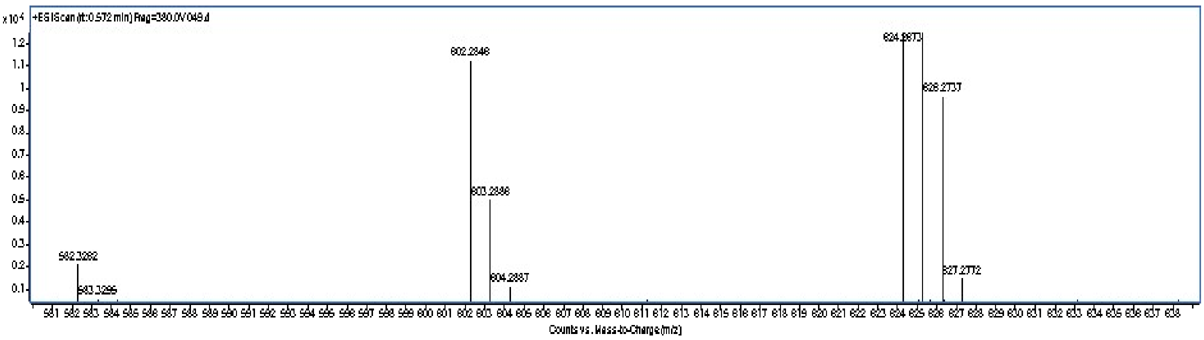

Figure 85. HRMS spectrum of **12o**

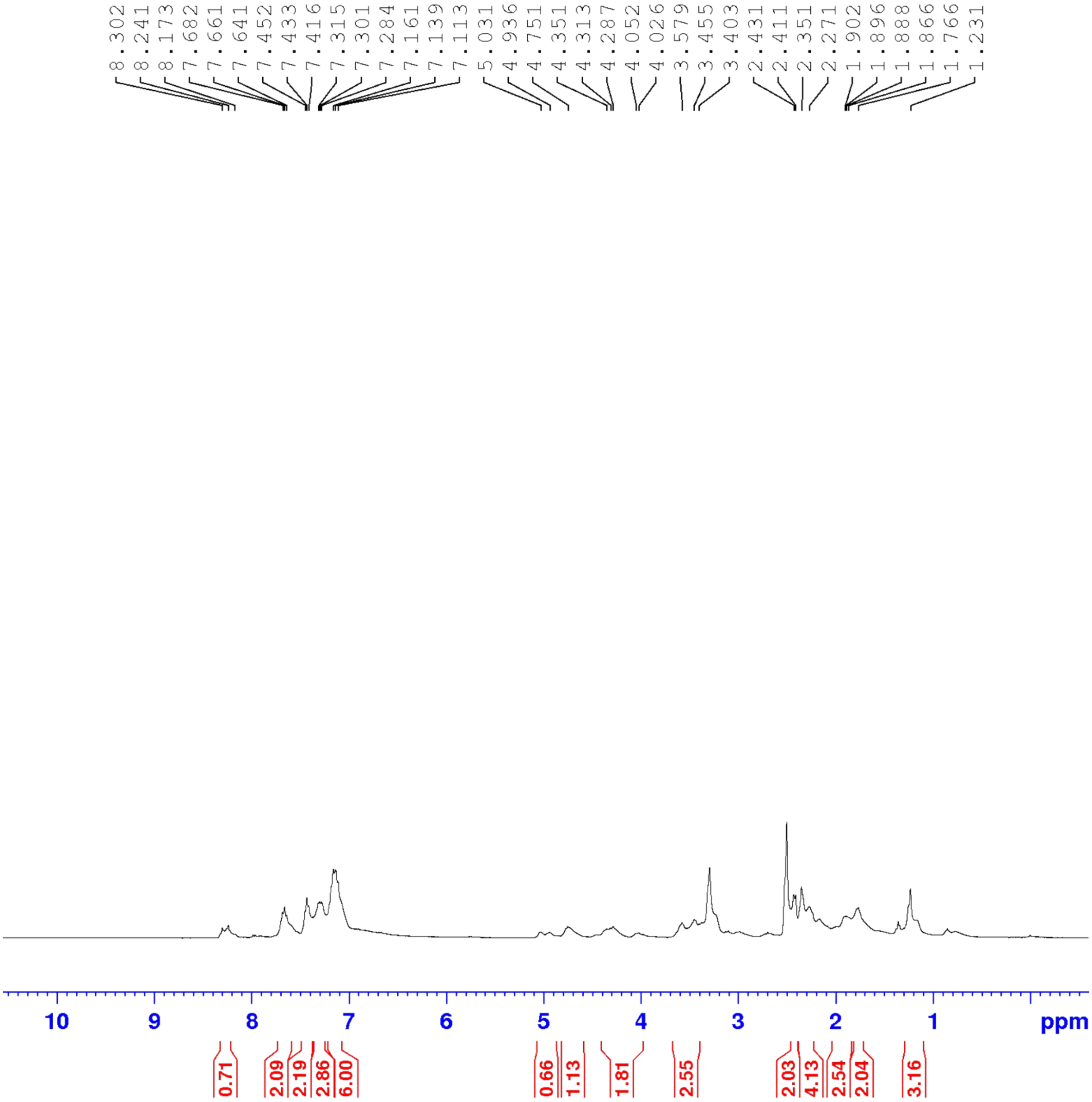

Figure 86. ^{1}H NMR full spectrum of **12o**

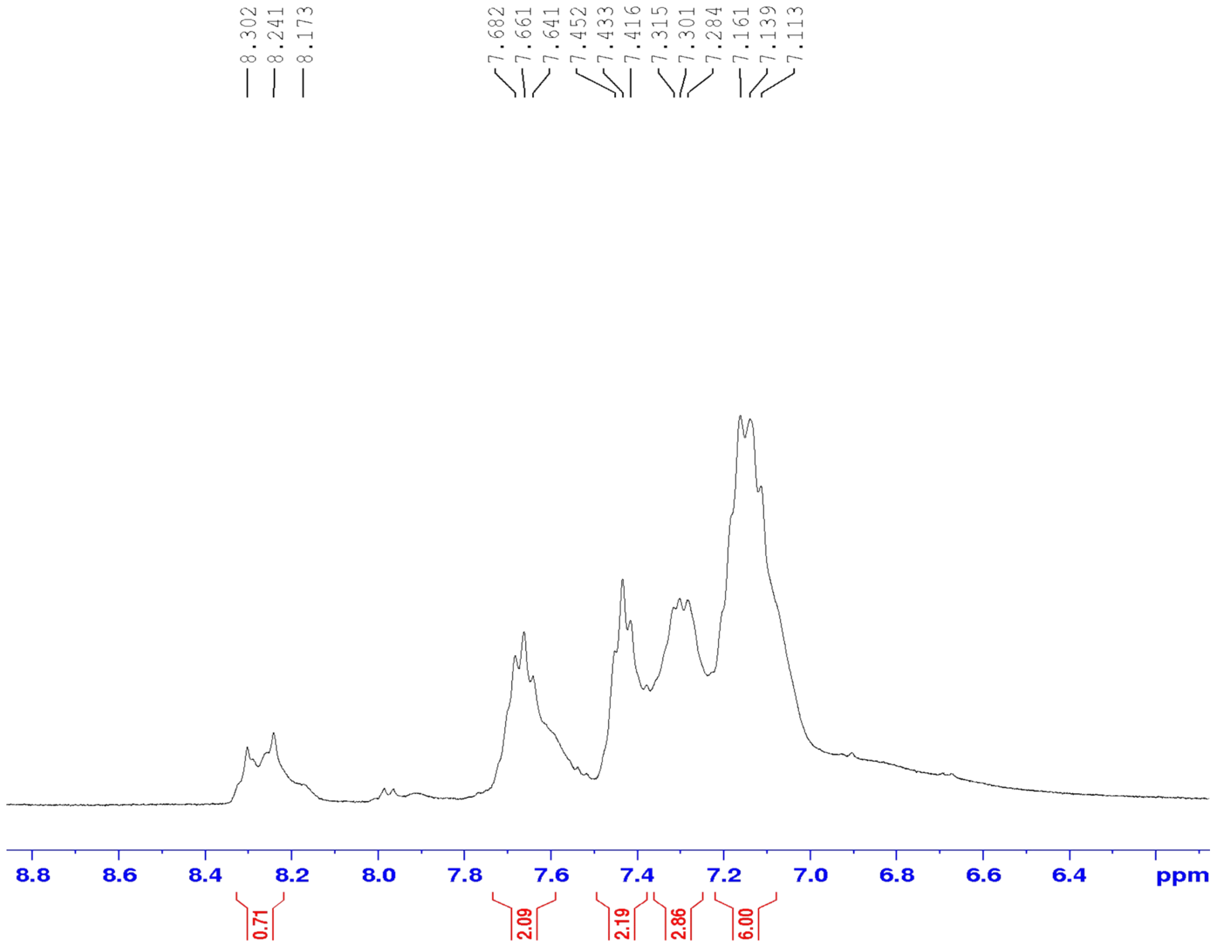

Figure 87. Aromatic region of the ^{1}H NMR spectrum for **12o**

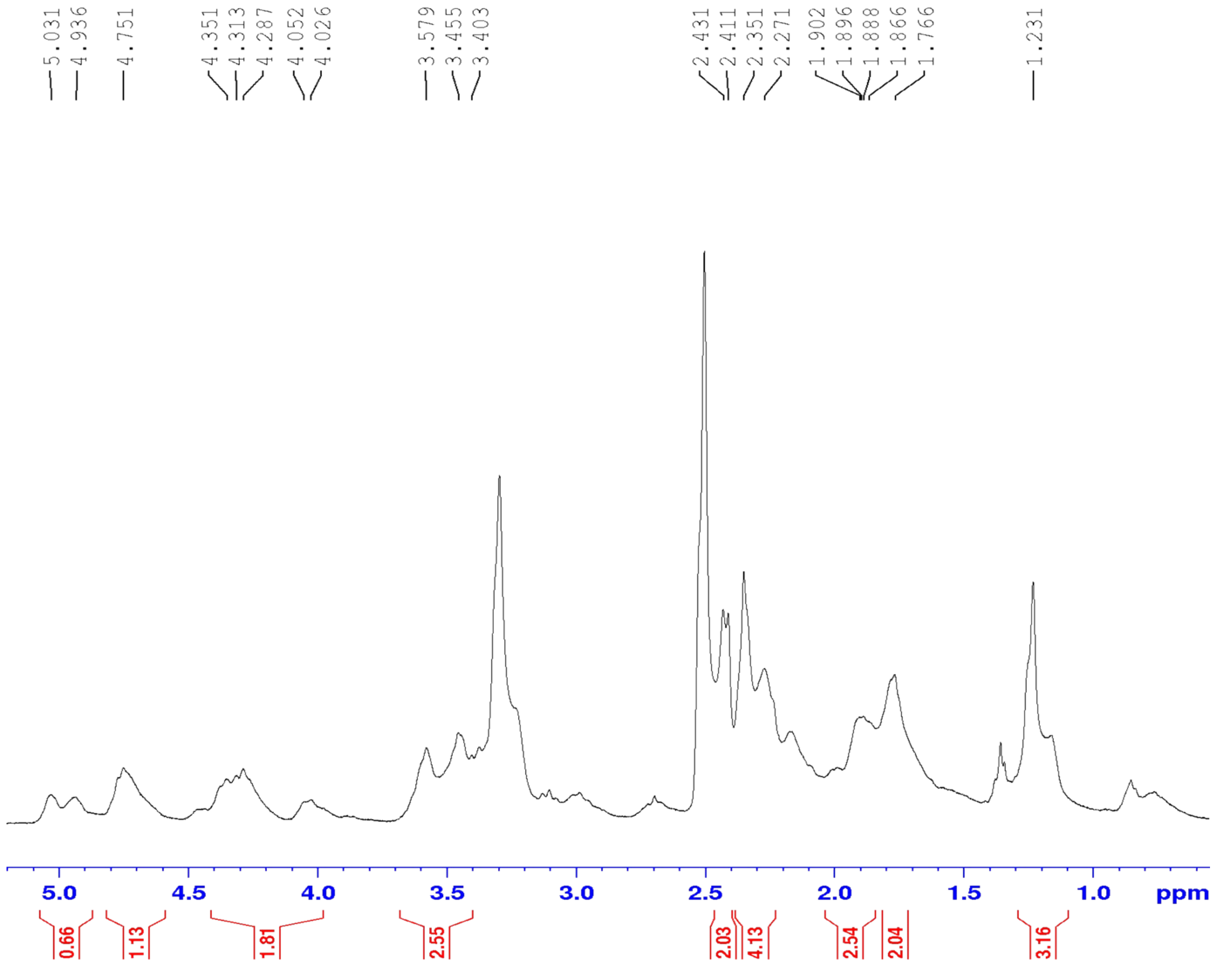

Figure 88. Aliphatic region of the ^{1}H NMR spectrum for **12o**

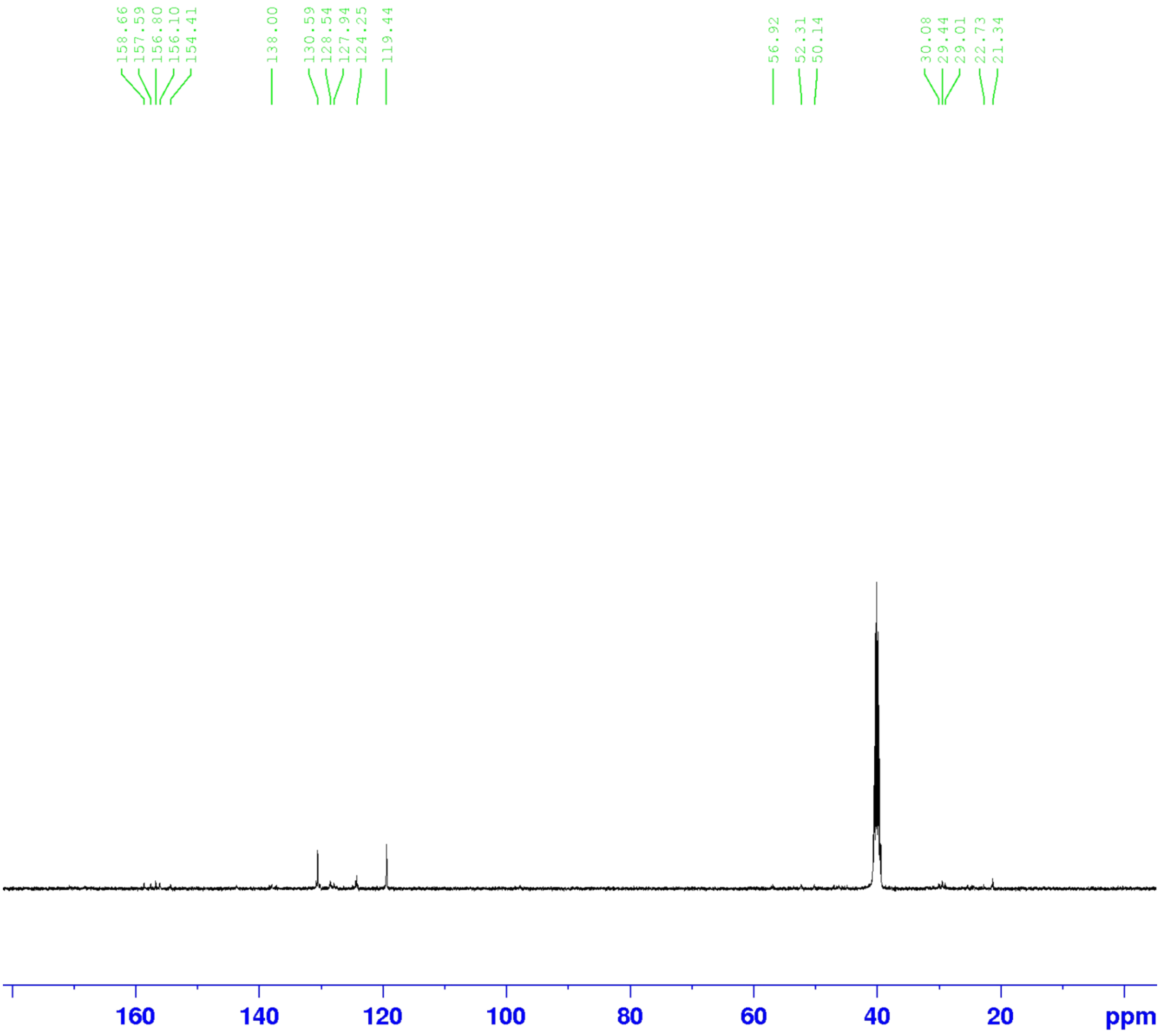

Figure 89. ^{13}C-NMR spectrum of **12o**

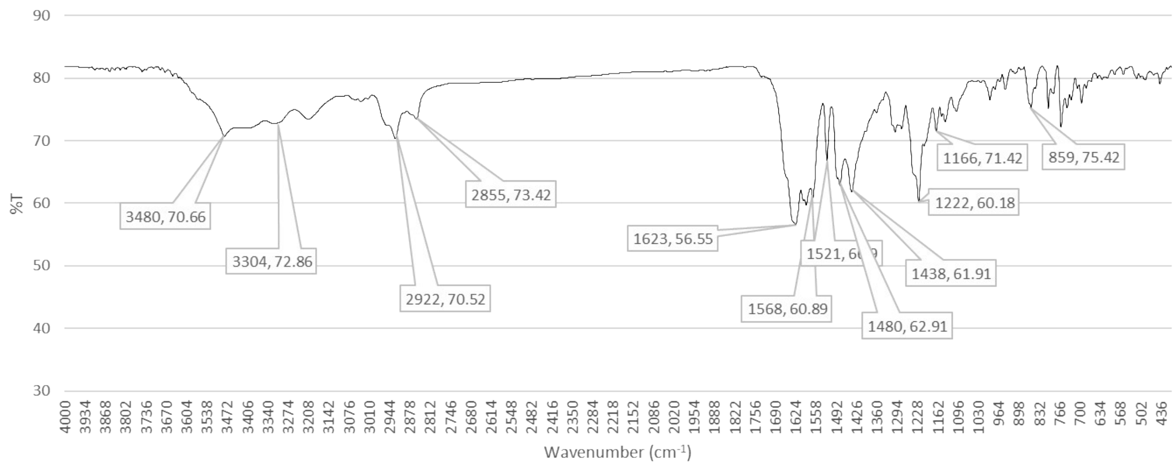

Figure 90. FT-IR spectrum of **12o**

Spectral data of ***4-amino-1-((3R)-1-((5-chlorothiophene-2-carbonyl)prolyl)piperidin-3-yl)-3-(4-phenoxyphenyl)-1H-pyrazolo[3,4-d]pyrimidine (12p)***

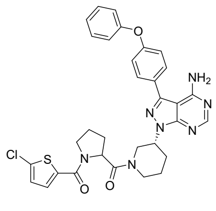

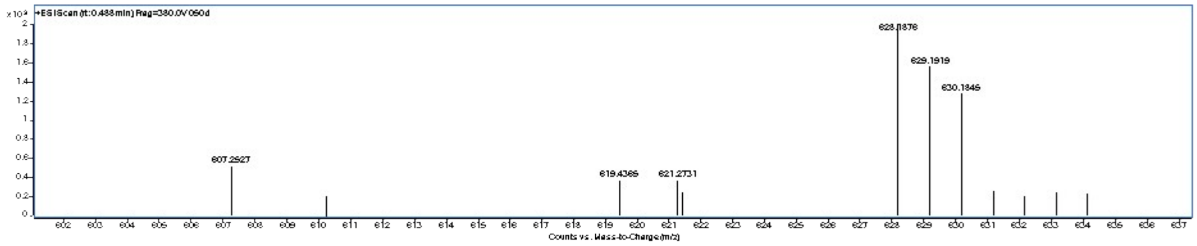

Figure 91. HRMS spectrum of **12p**

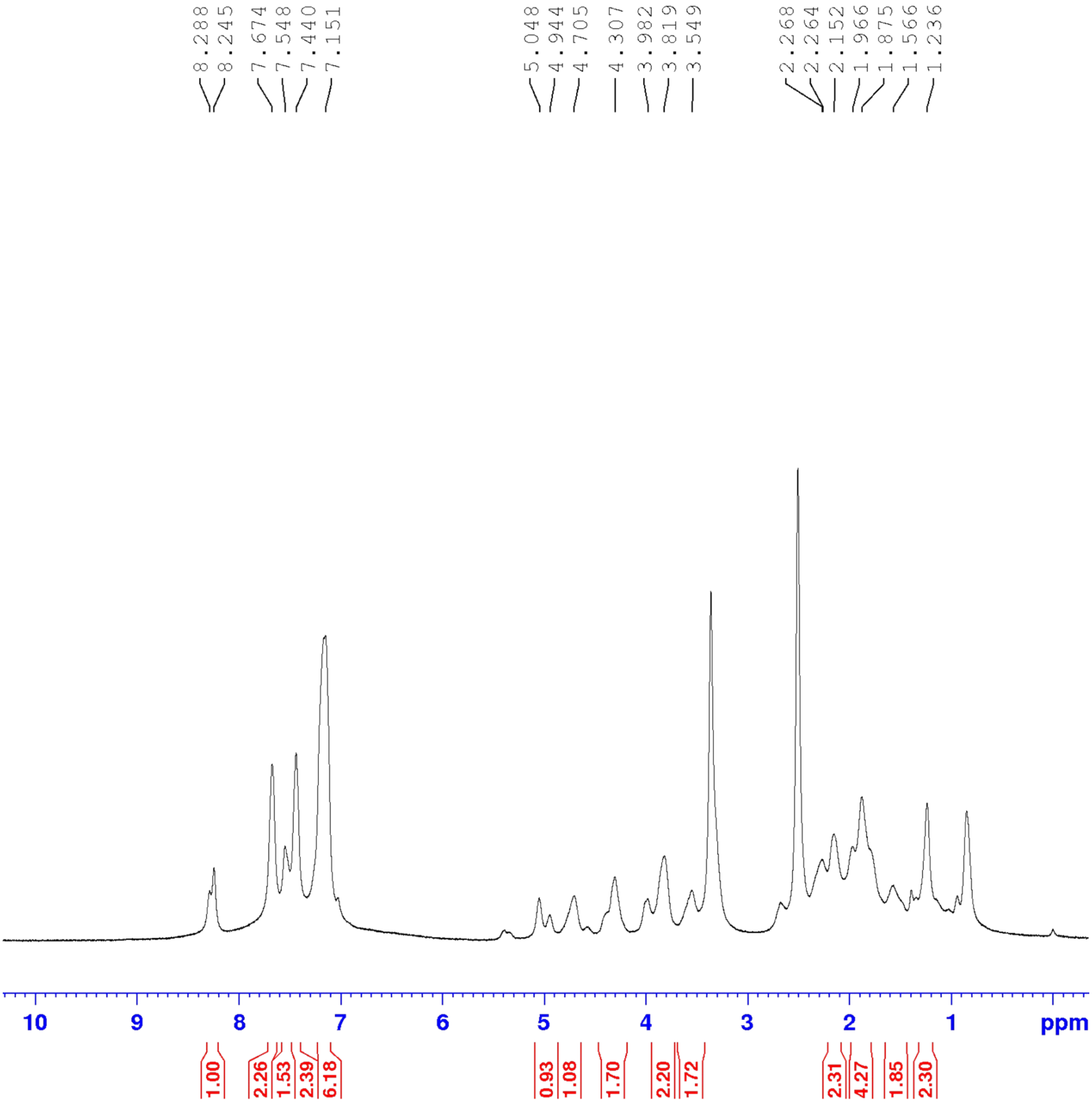

Figure 92. ^{1}H NMR full spectrum of **12p**

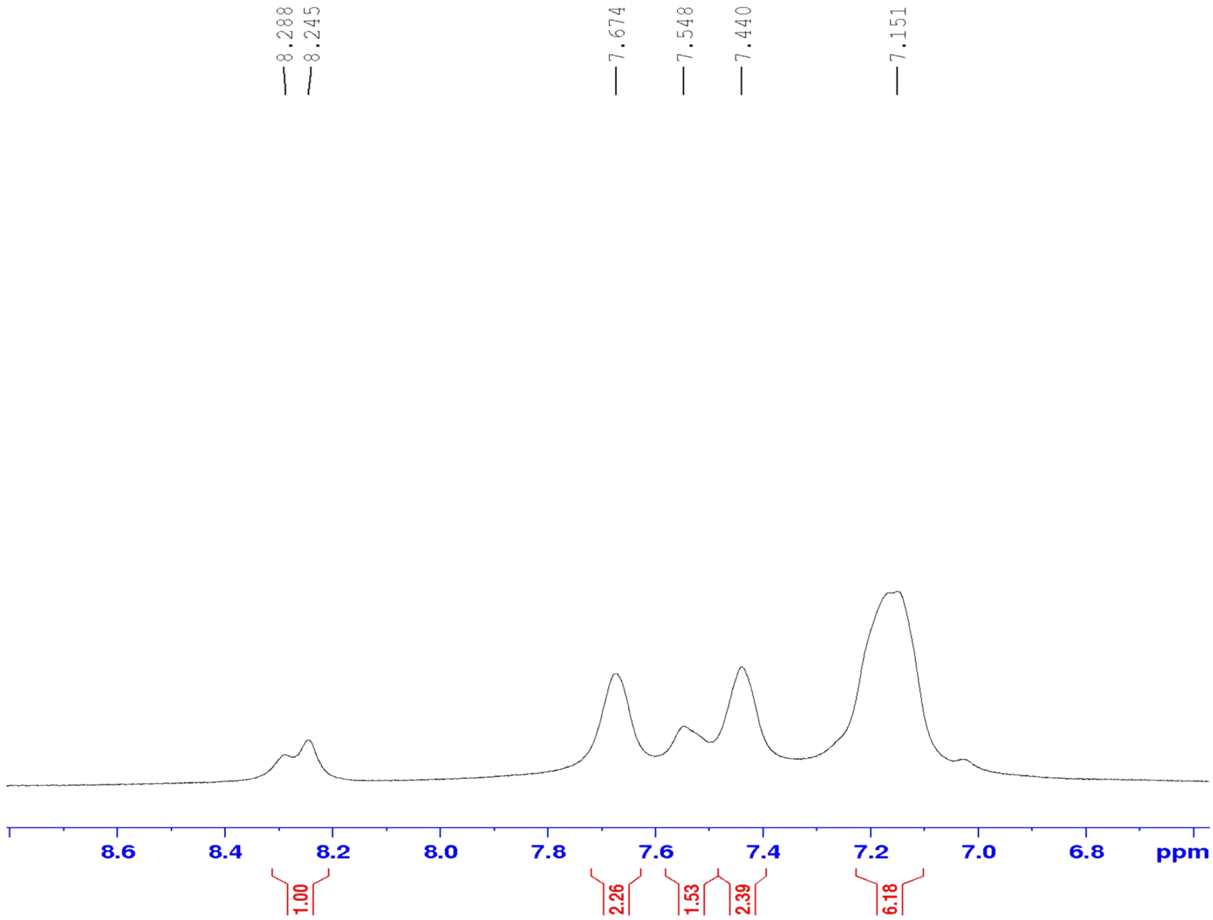

Figure 93. Aromatic region of the ^{1}H NMR spectrum for **12p**

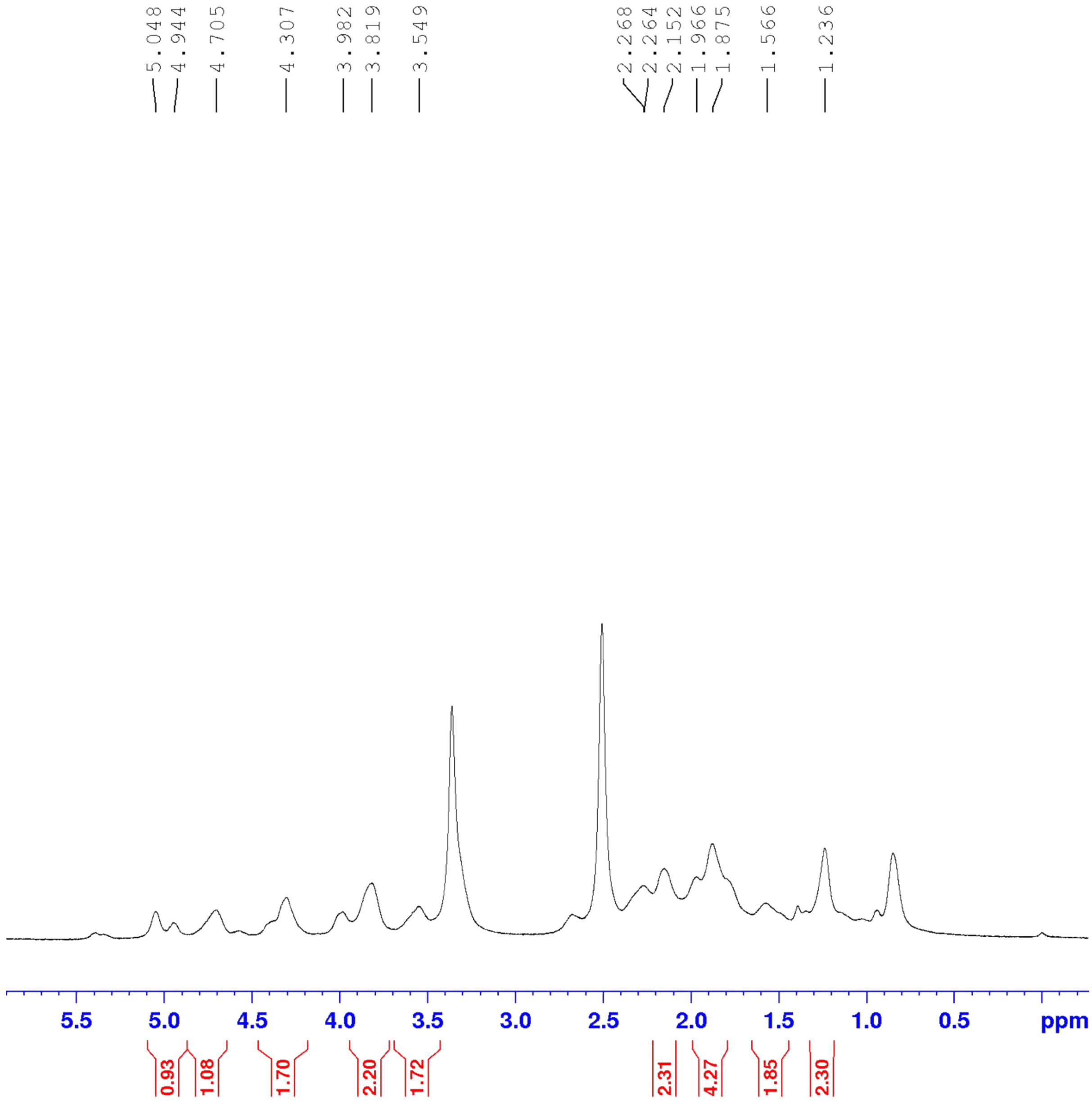

Figure 94. Aliphatic region of the ^{1}H NMR spectrum for **12p**

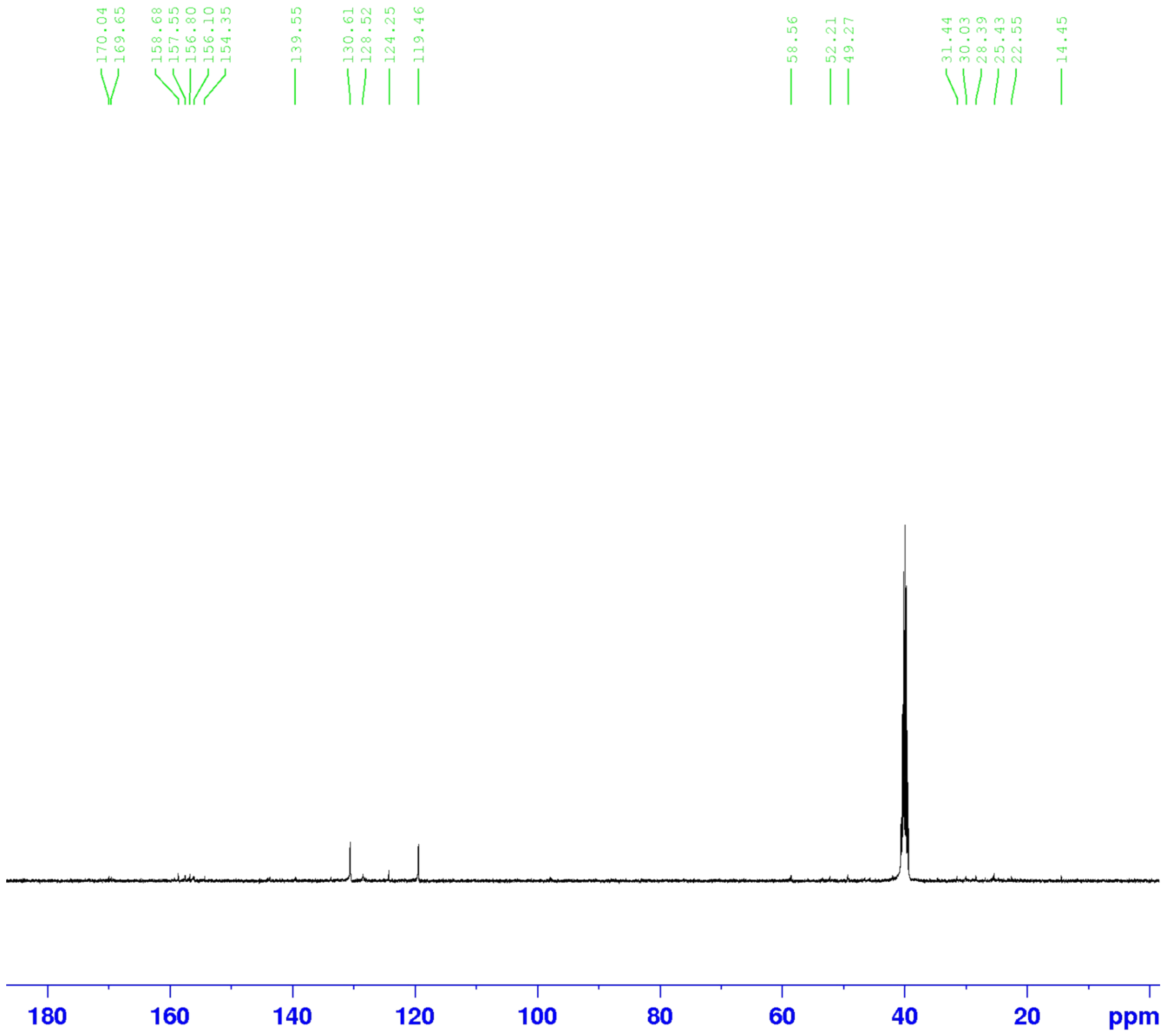

Figure 95. ^{13}C-NMR spectrum of **12p**

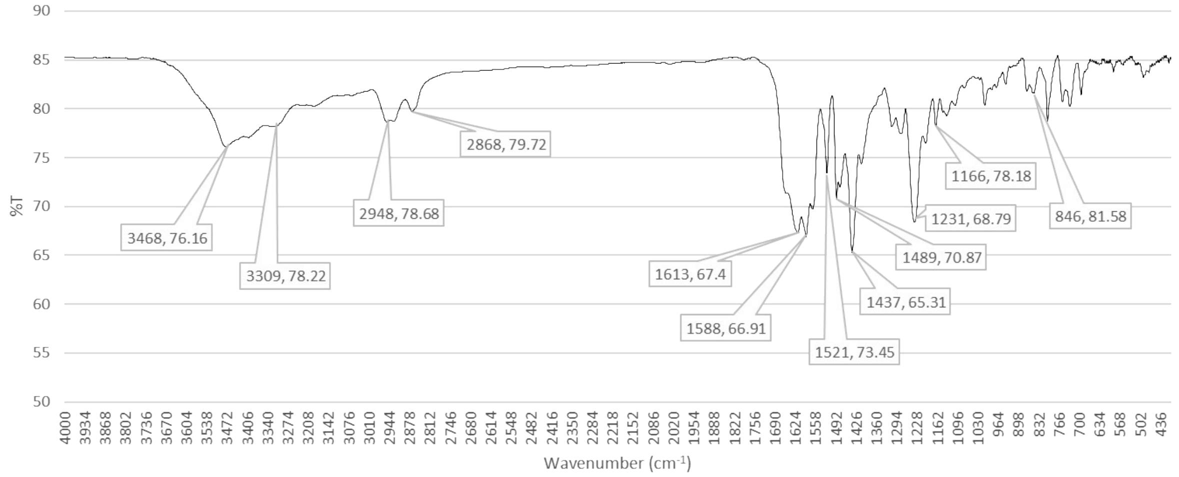

Figure 96. FT-IR spectrum of **12p**

Spectral data of ***4-amino-1-((3R)-1-((4-methoxybenzoyl)prolyl)piperidin-3-yl)-3-(4-phenoxyphenyl)-1H-pyrazolo[3,4-d]pyrimidine (12q)***

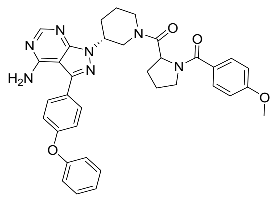

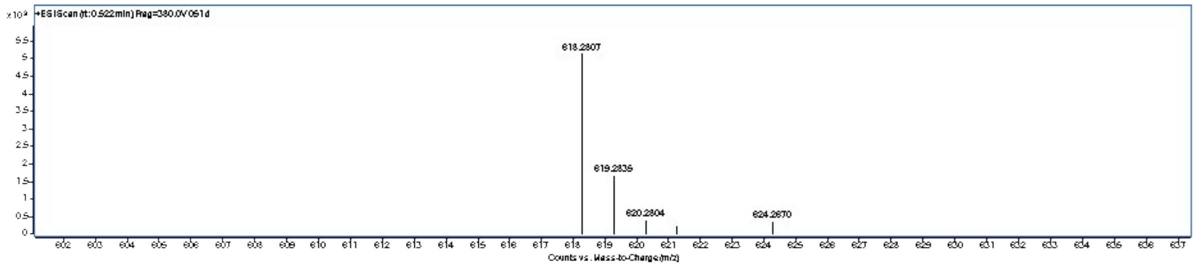

Figure 97. HRMS spectrum of **12q**

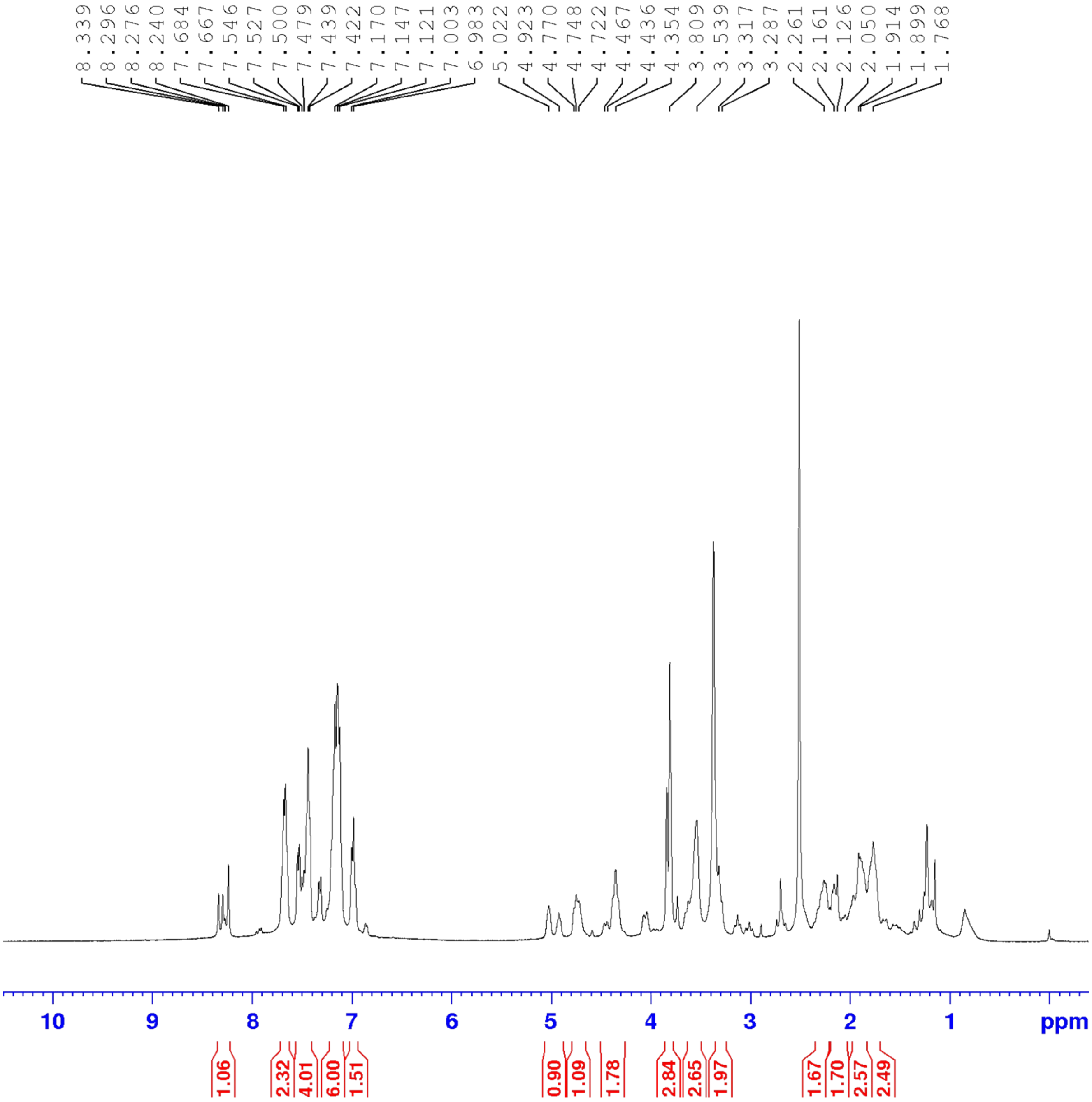

Figure 98. ^{1}H NMR full spectrum of **12q**

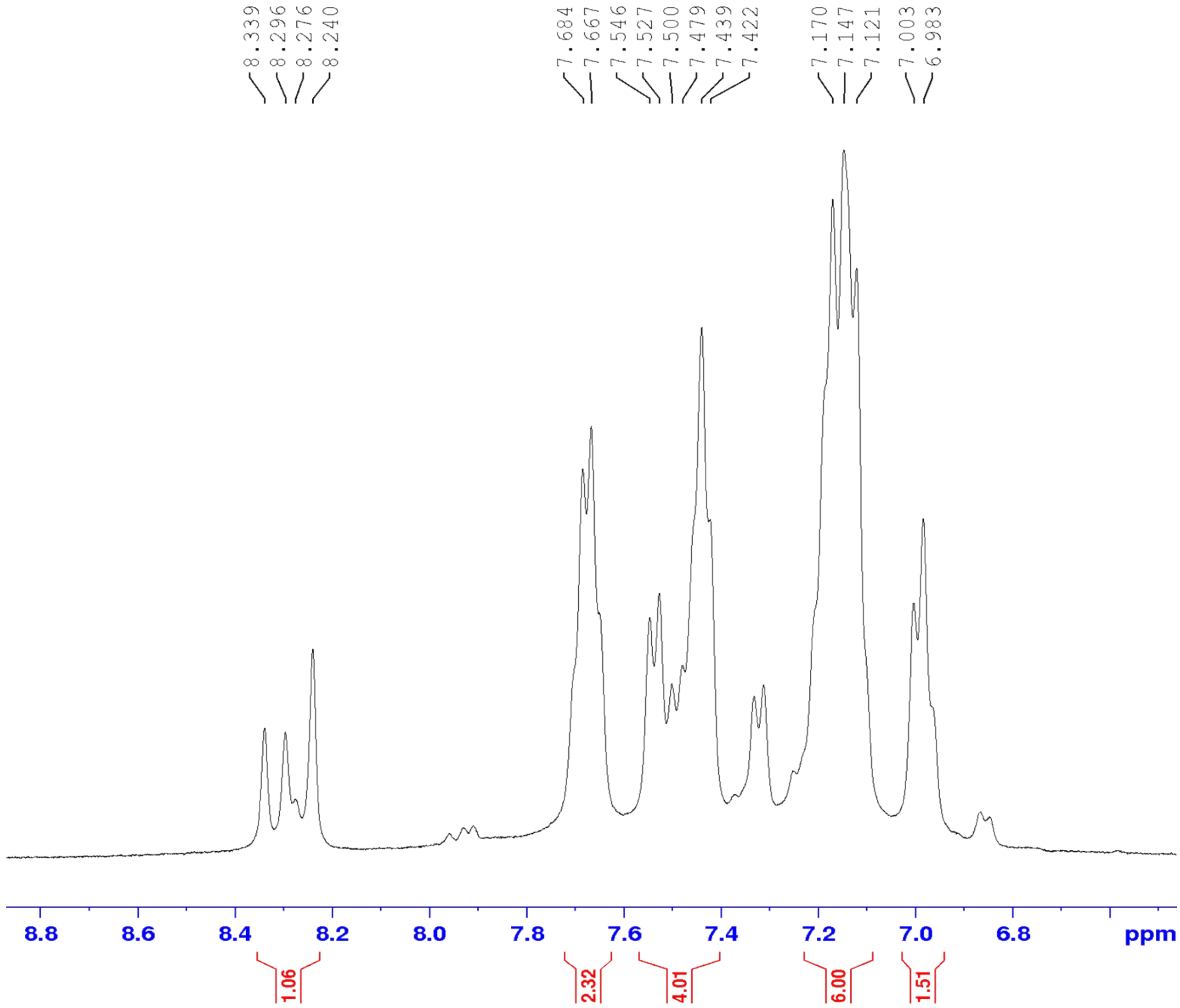

Figure 99. Aromatic region of the ^{1}H NMR spectrum for **12q**

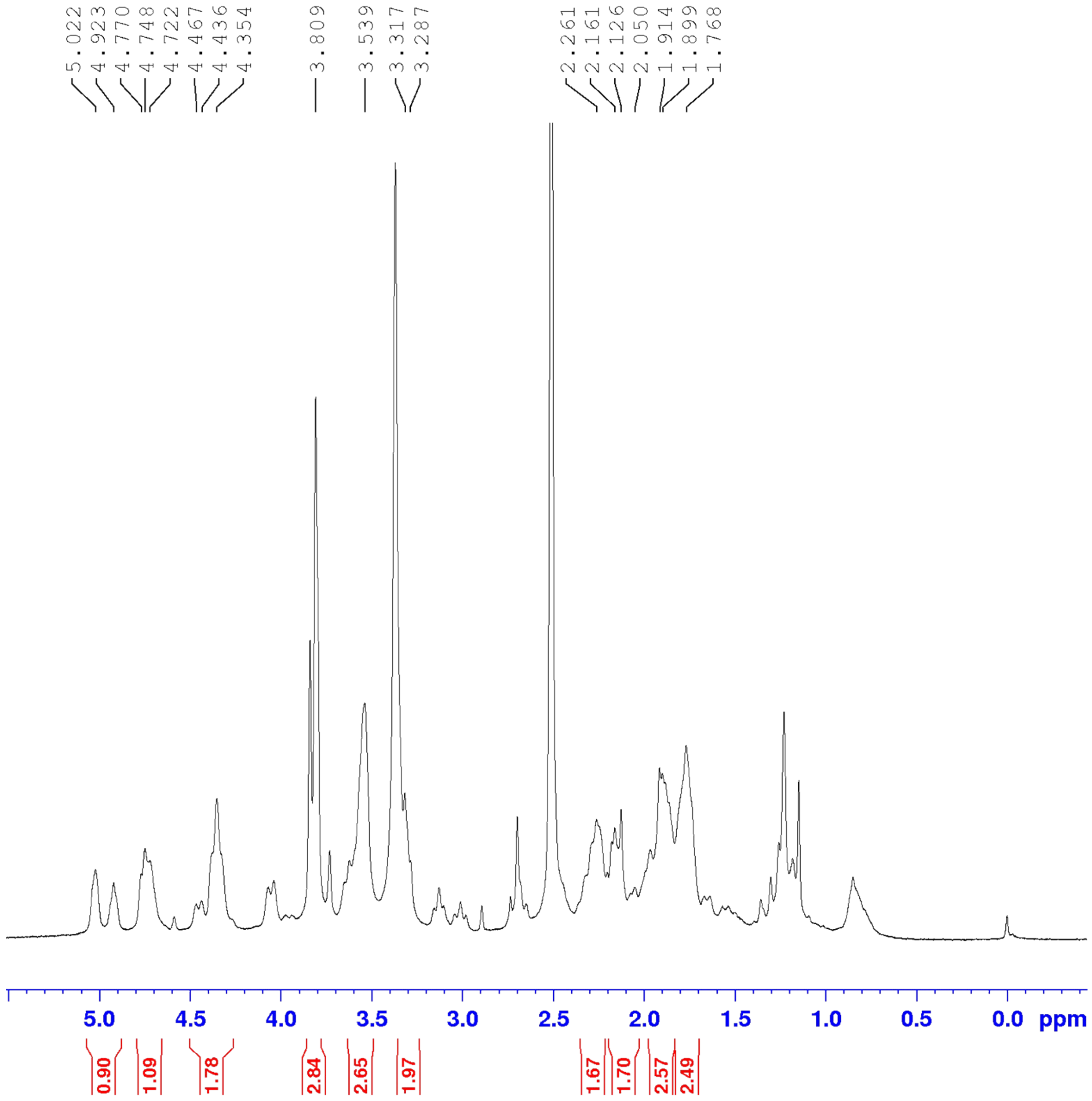

Figure 100. Aliphatic region of the ^{1}H NMR spectrum for **12q**

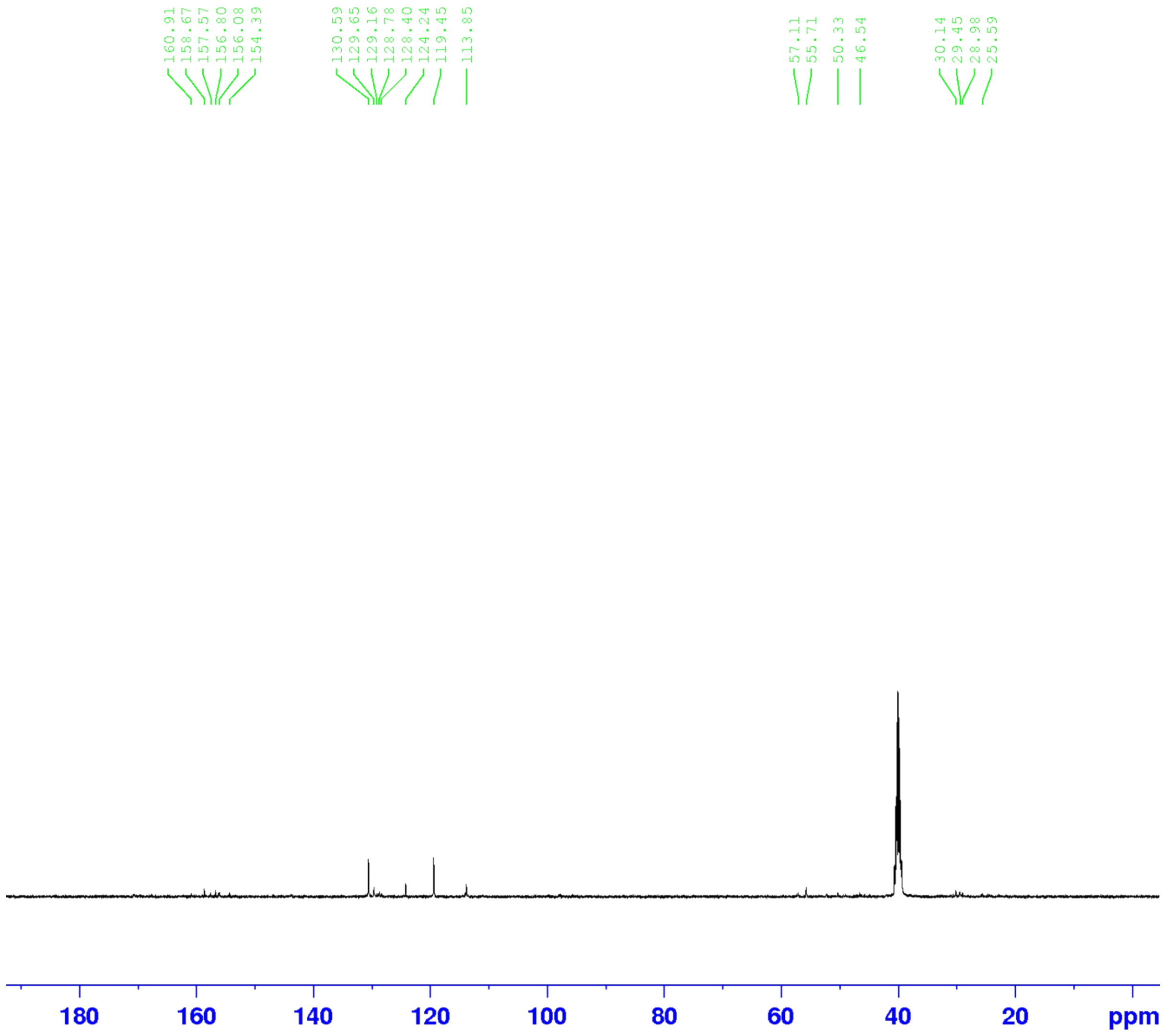

Figure 101. ^{13}C-NMR spectrum of **12q**

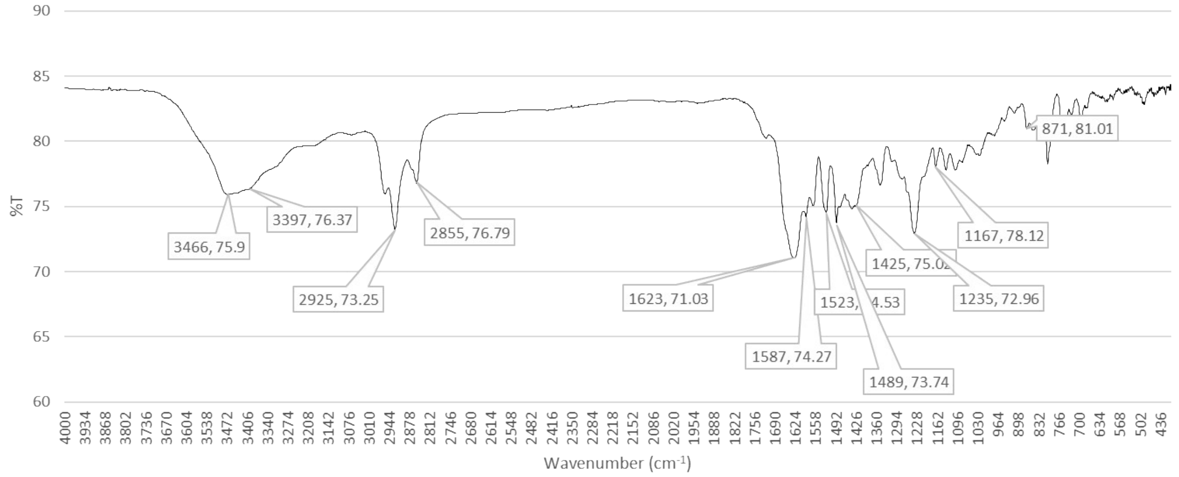

Figure 102. FT-IR spectrum of **12q**

Spectral data of ***7-amino-3-((3R)-1-((3-chlorobenzoyl)prolyl)piperidin-3-yl)-1-(4-phenoxyphenyl)-1H-pyrazolo[4,3-d]pyrimidine (12r)***

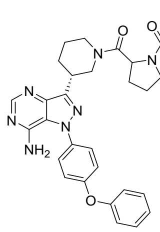

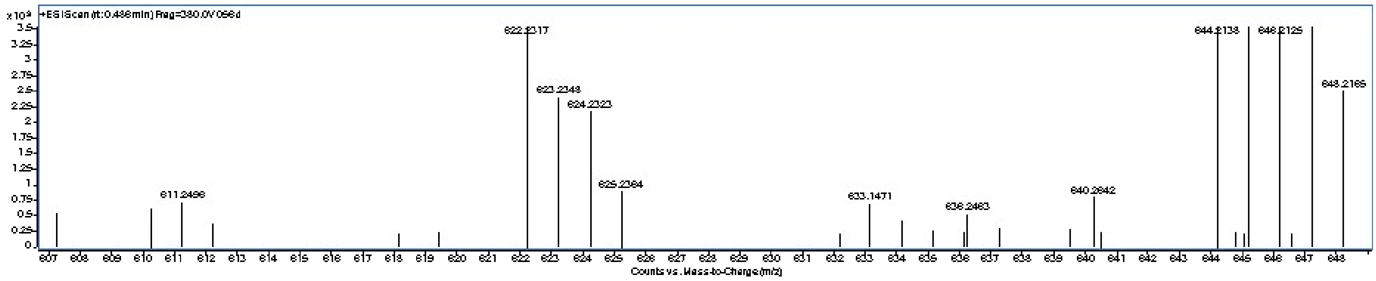

Figure 103. HRMS spectrum of **12r**

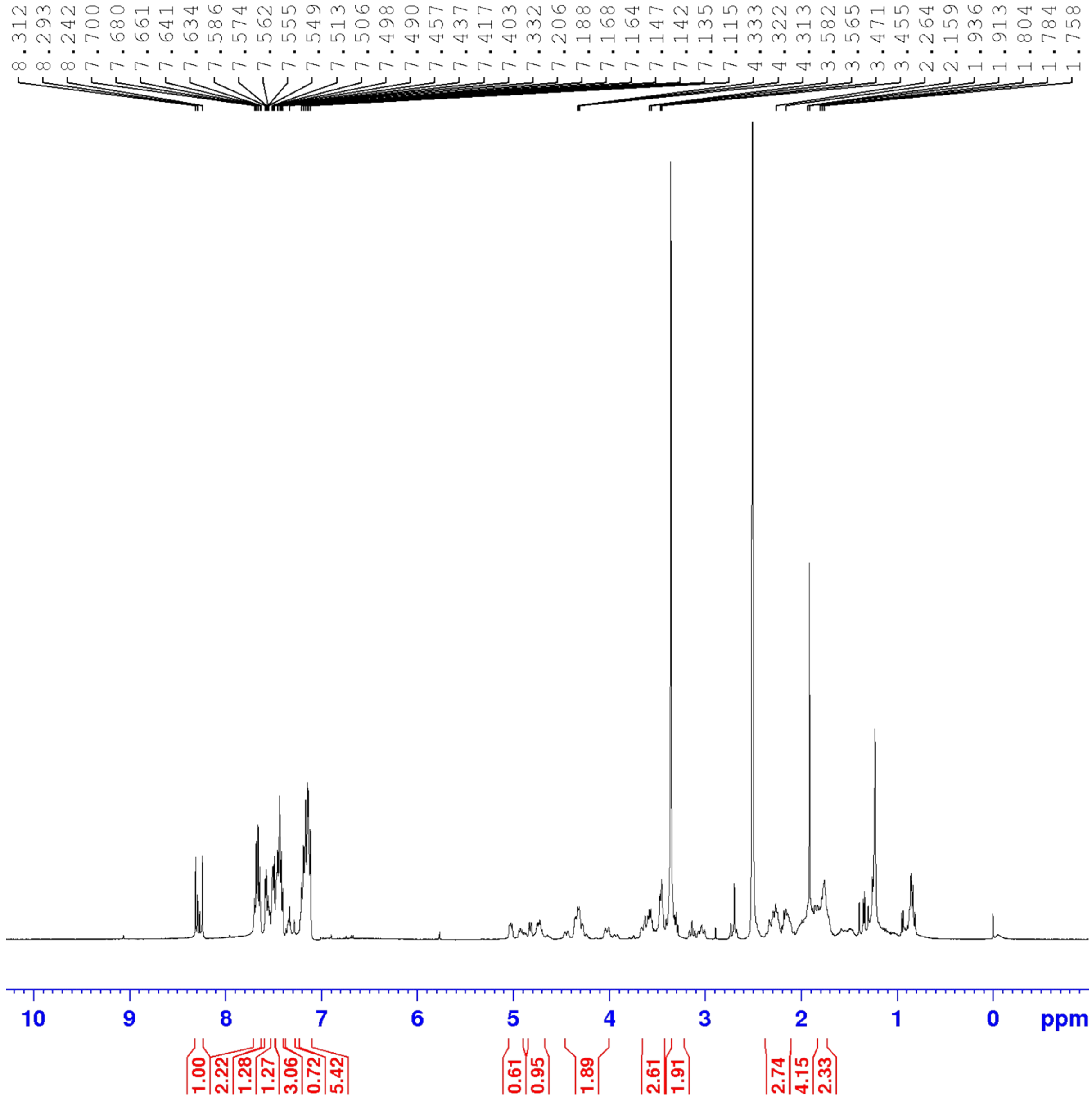

Figure 104. ^{1}H NMR full spectrum of **12r**

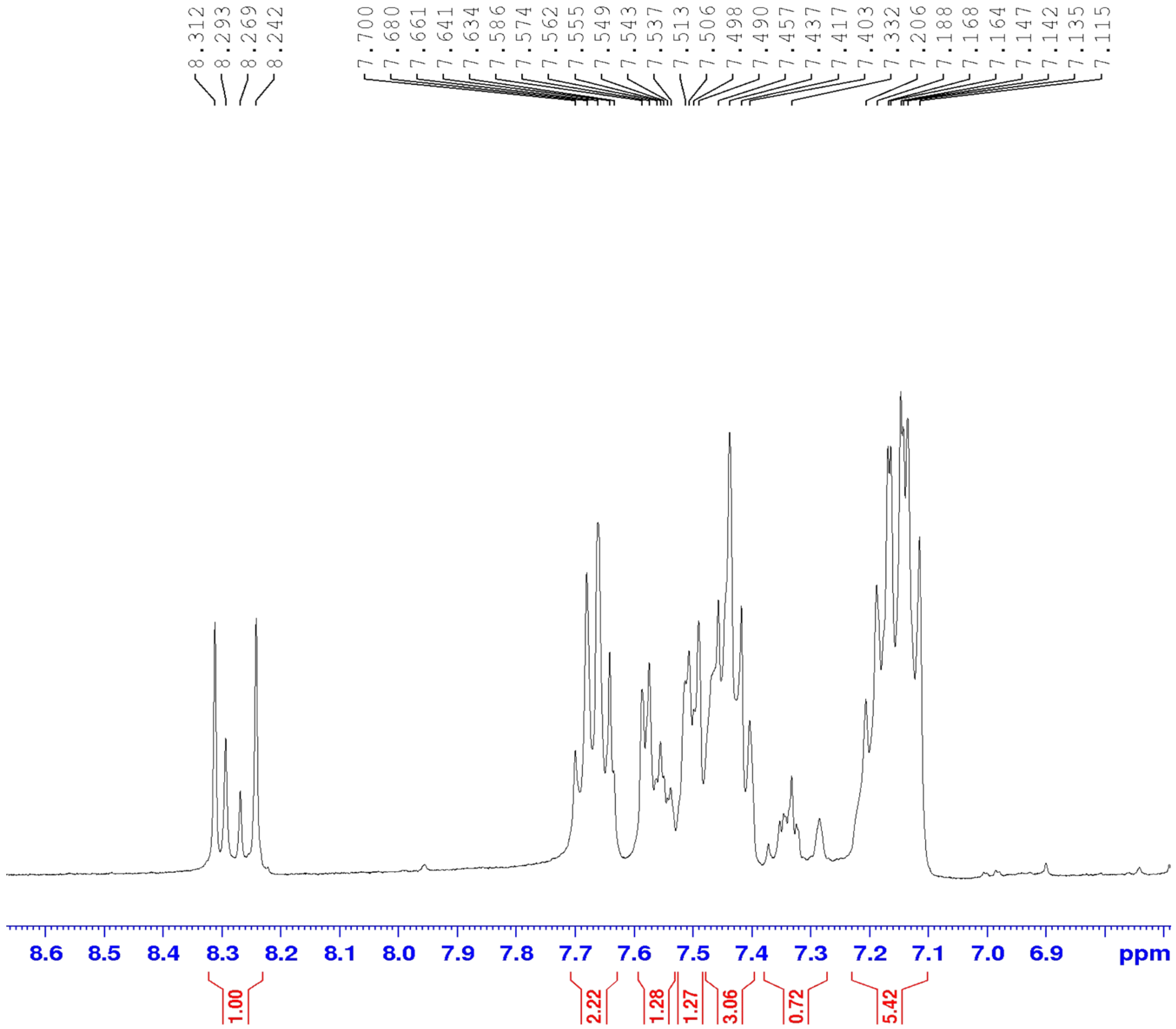

Figure 105. Aromatic region of the ^1H NMR spectrum for **12r**

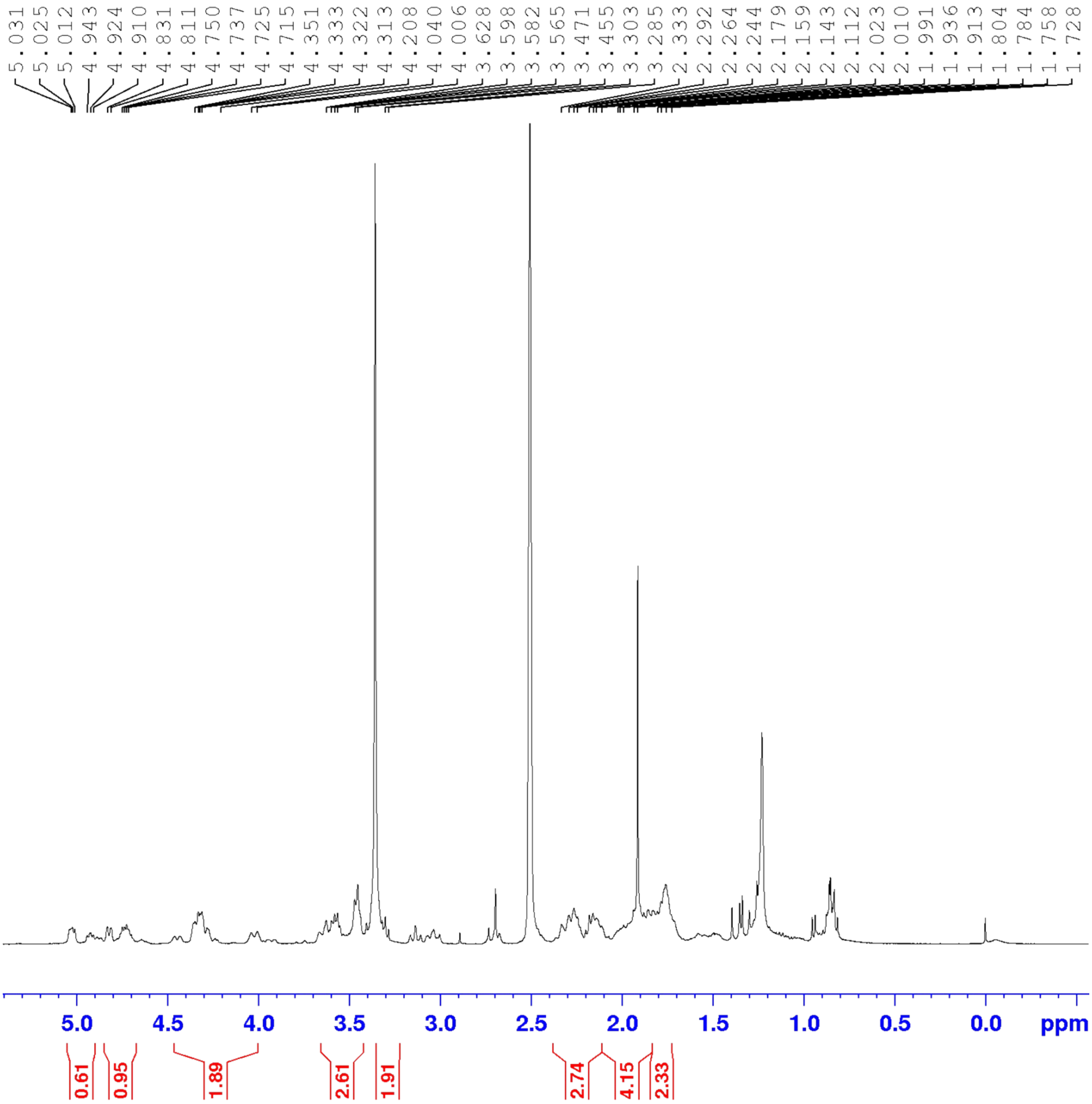

Figure 106. Aliphatic region of the ^{1}H NMR spectrum for **12r**

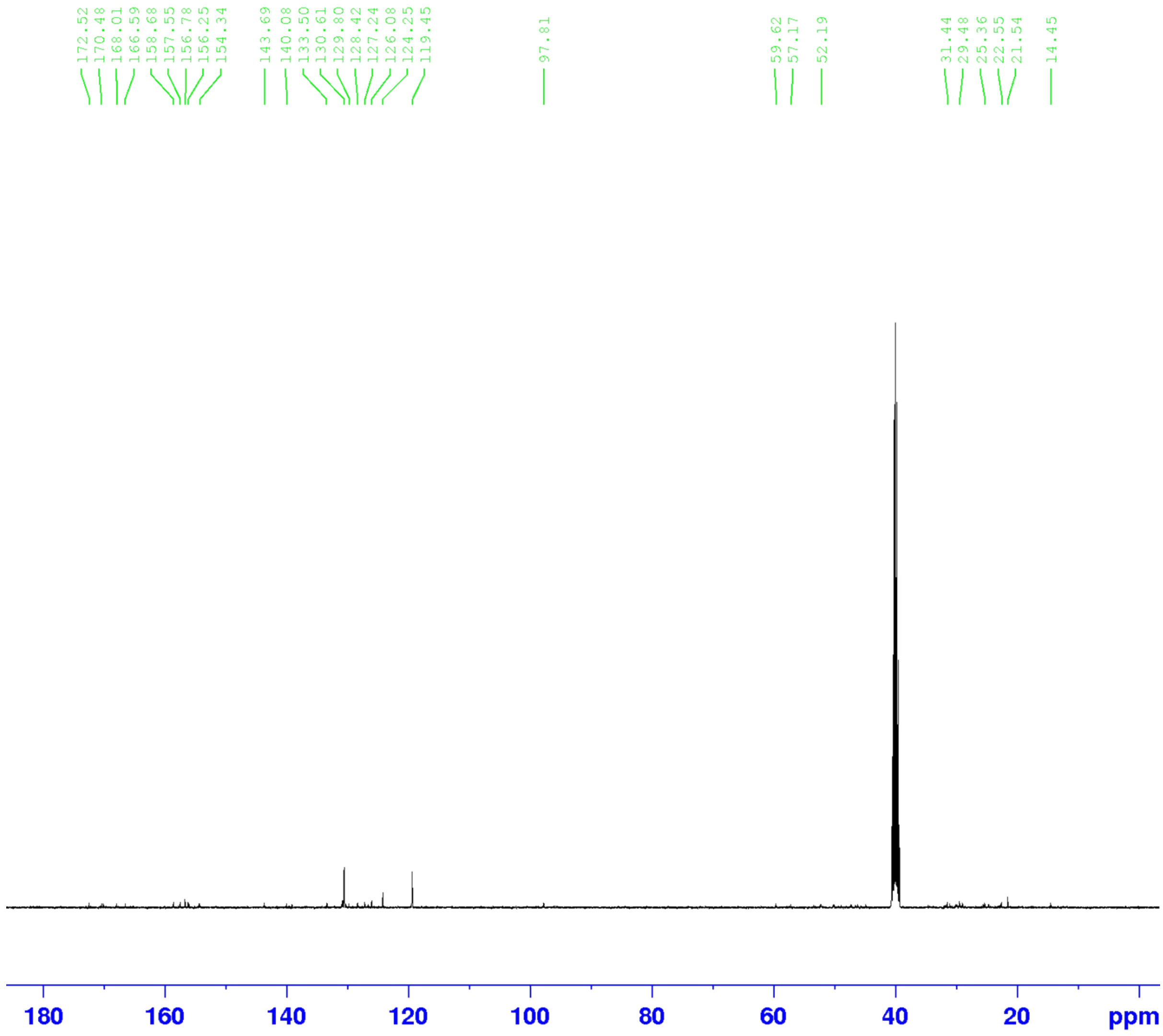

Figure 107. ^{13}C-NMR spectrum of **12r**

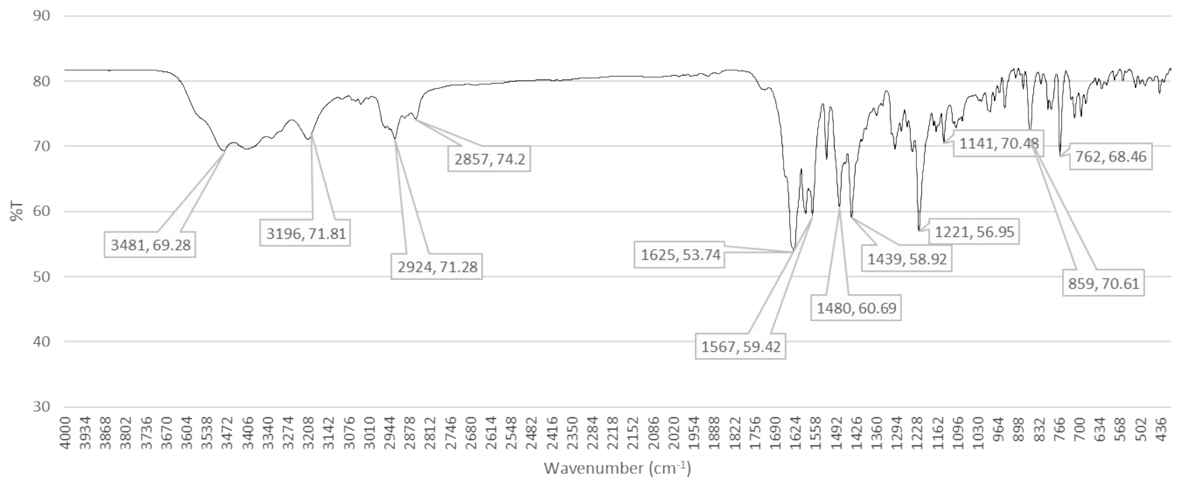

Figure 108. FT-IR spectrum of **12r**

Spectral data of ***7-amino-3-((3R)-1-((4-nitrobenzoyl)prolyl)piperidin-3-yl)-1-(4-phenoxyphenyl)-1H-pyrazolo[4,3-d]pyrimidine (12s)***

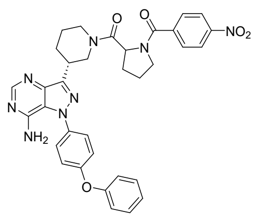

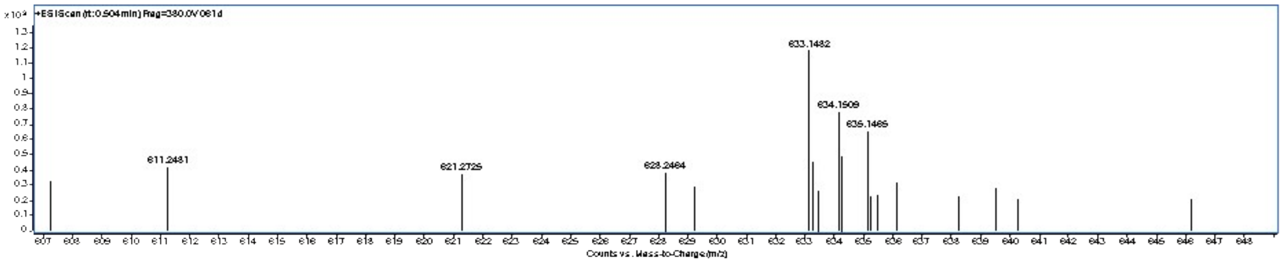

Figure 109. HRMS spectrum of **12s**

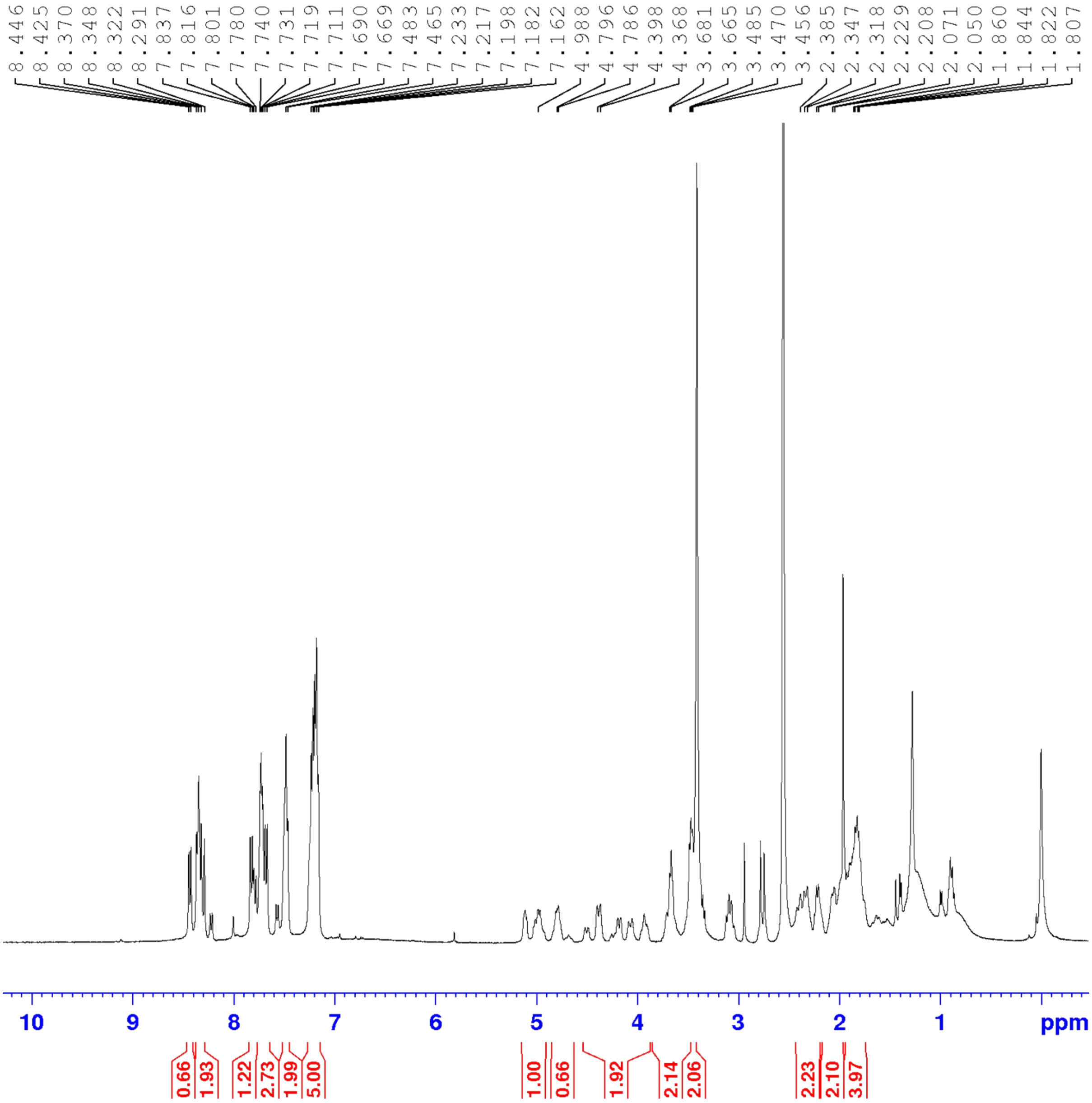

Figure 110. ^{1}H NMR full spectrum of **12s**

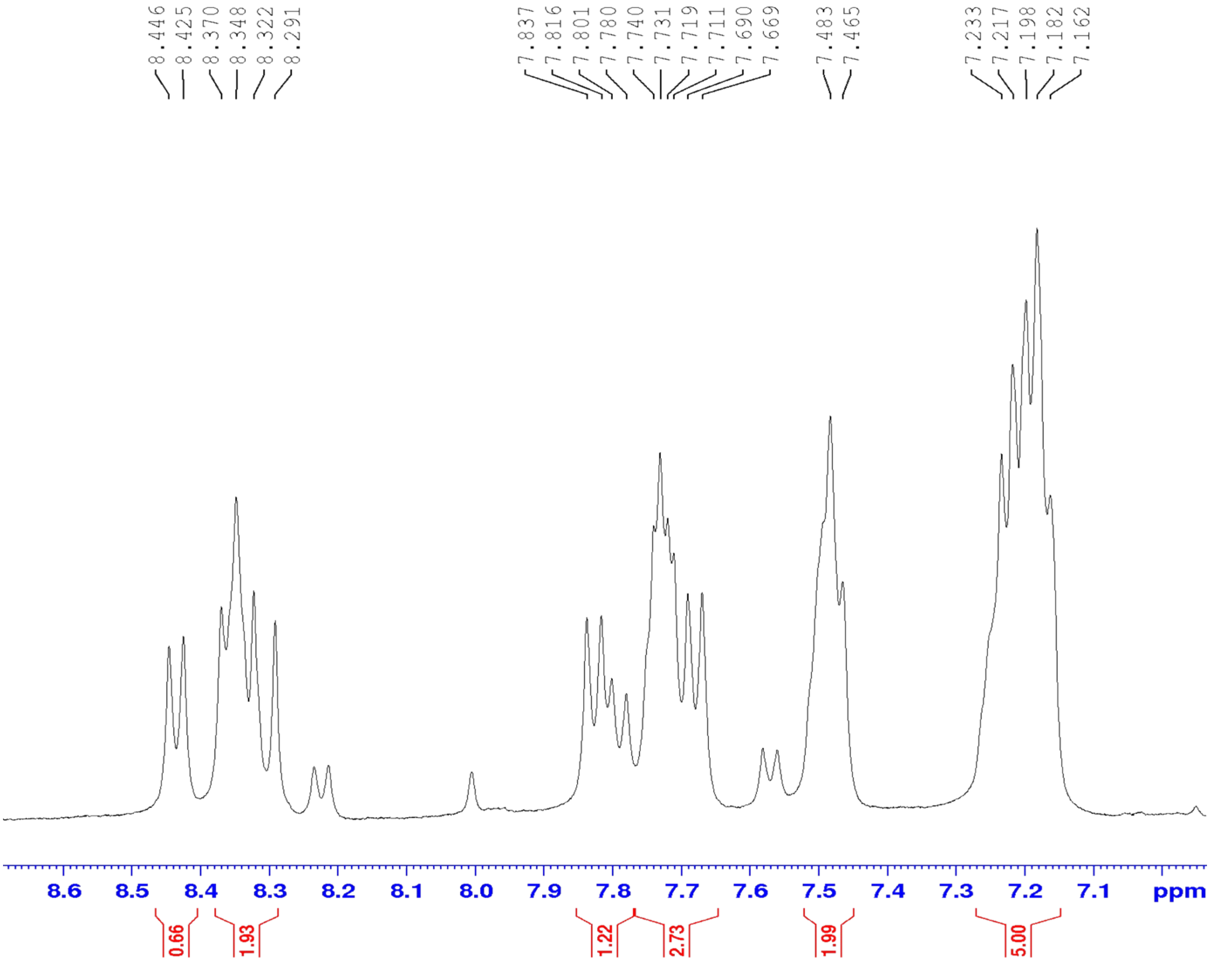

Figure 111. Aromatic region of the ^{1}H NMR spectrum for **12s**

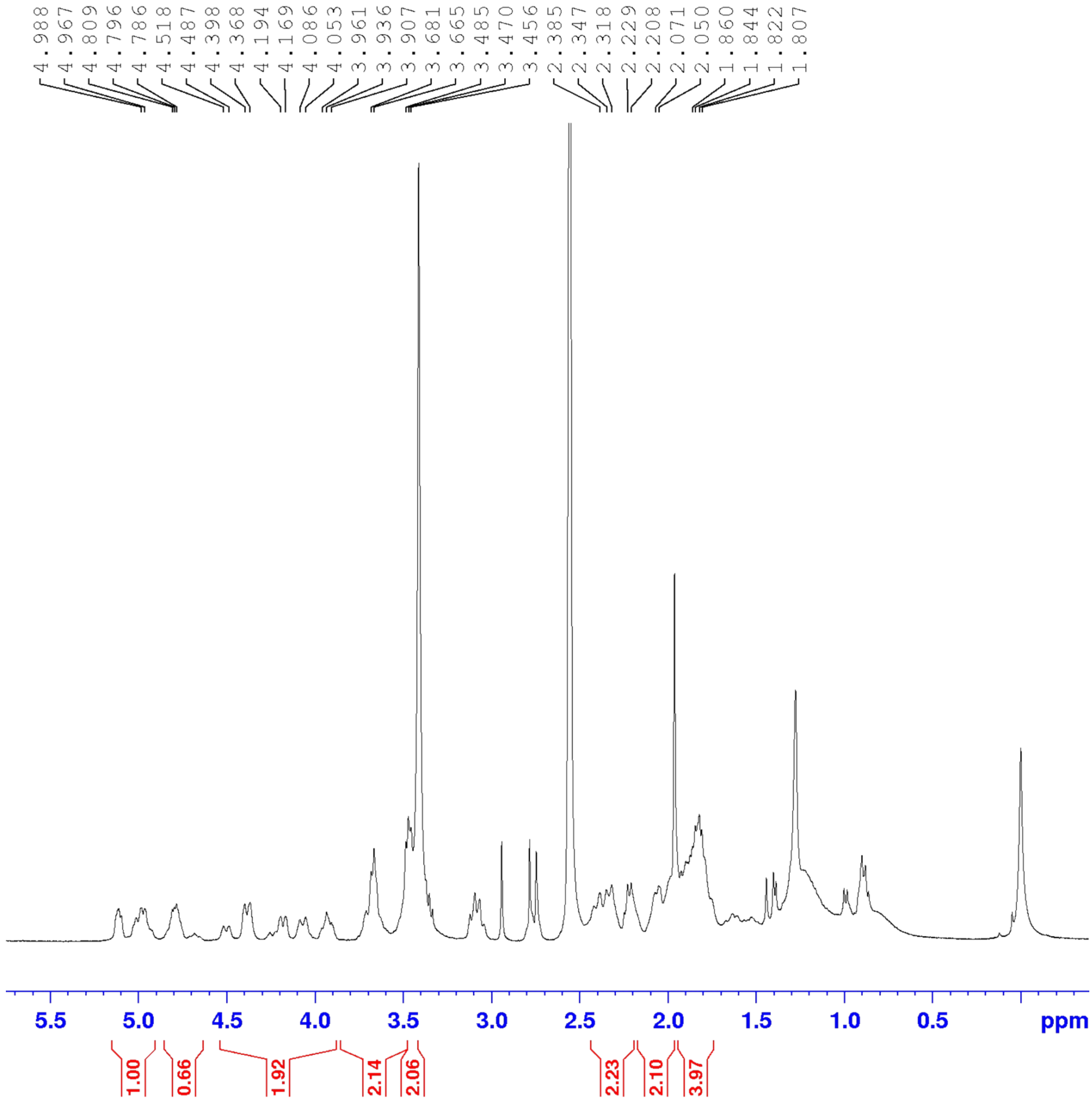

Figure 112. Aliphatic region of the ^{1}H NMR spectrum for **12s**

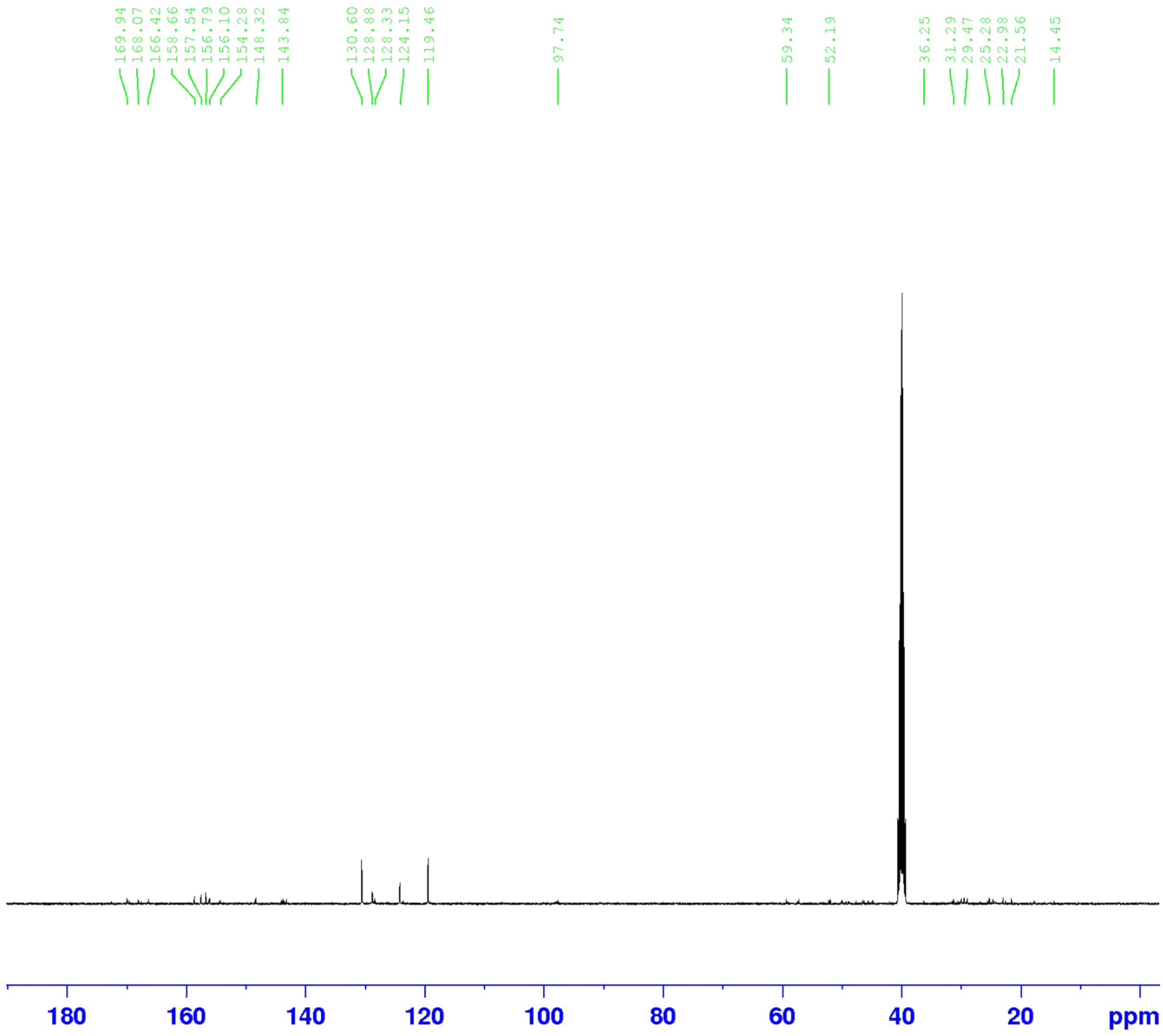

Figure 113. ^{13}C-NMR spectrum of **12s**

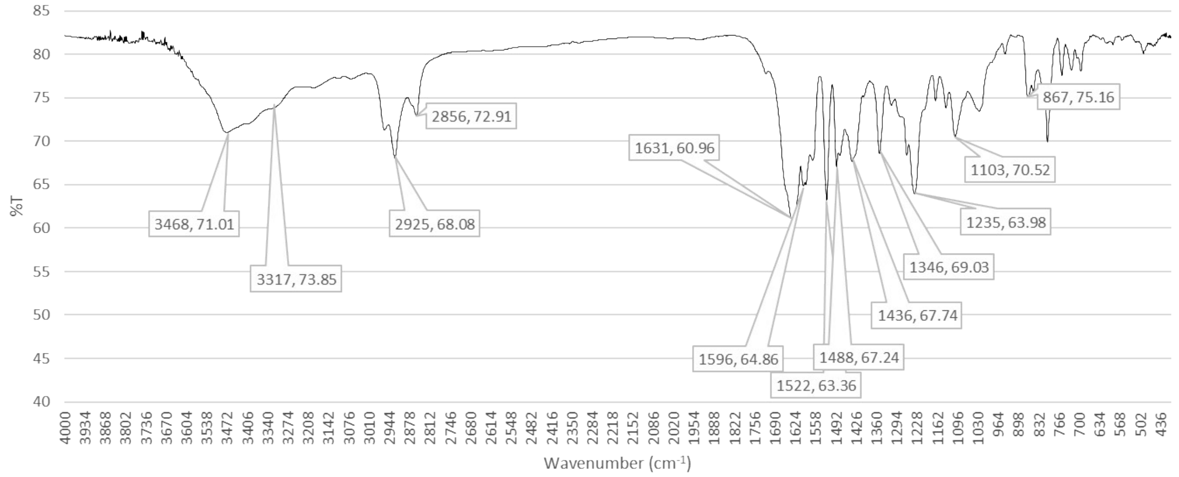

Figure 114. FT-IR spectrum of **12s**

Spectral data of ***((R)-3-(7-amino-1-(4-phenoxyphenyl)-1H-pyrazolo[4,3-d]pyrimidin-3-yl)piperidin-1-yl)(1-(5-((6-chloro-2-methylpyrimidin-4-yl)amino)thiazole-2-carbonyl)pyrrolidin-2-yl)methanone (12t)***

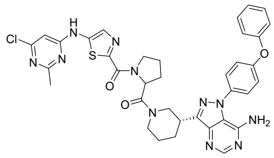

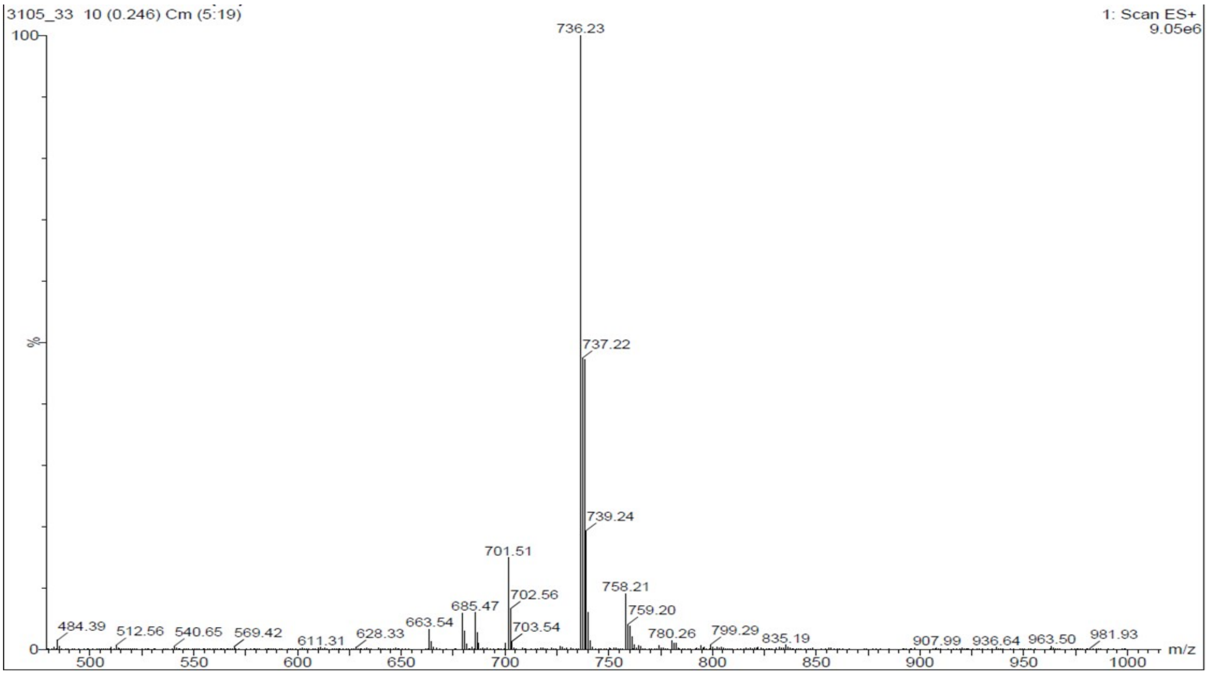

Figure 115. ESI-MS spectrum of **12t**

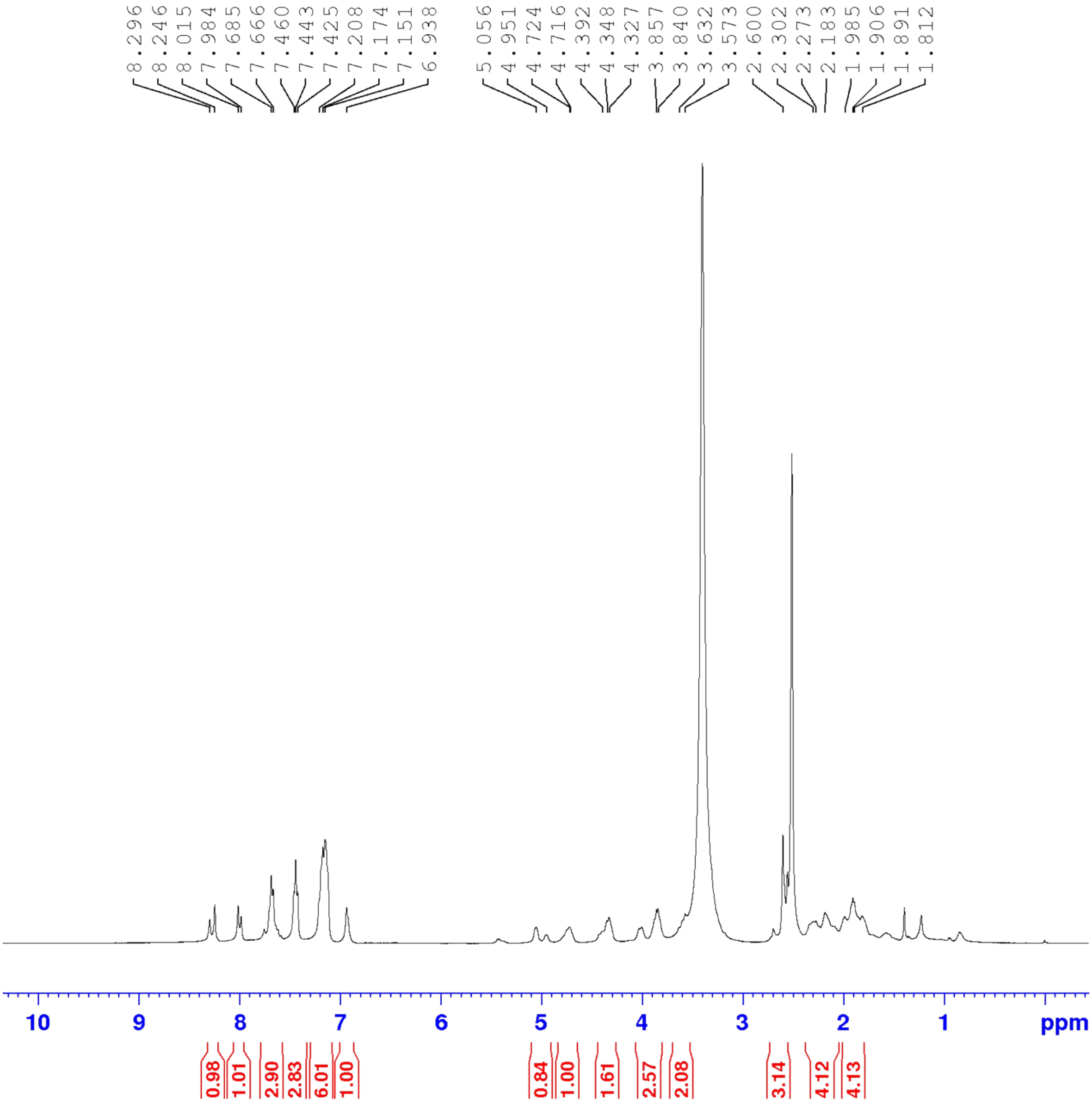

Figure 116. ^{1}H NMR full spectrum of **12t**

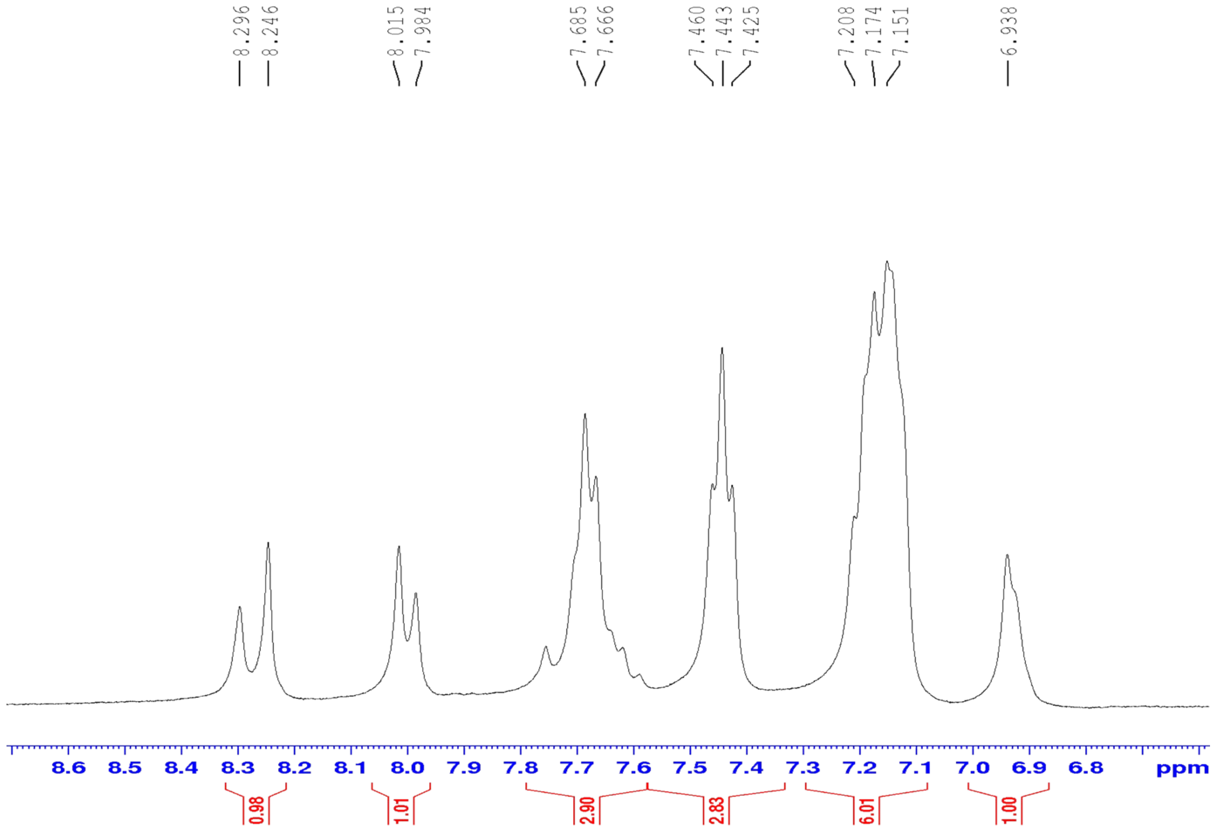

Figure 117. Aromatic region of the ^{1}H NMR spectrum for **12t**

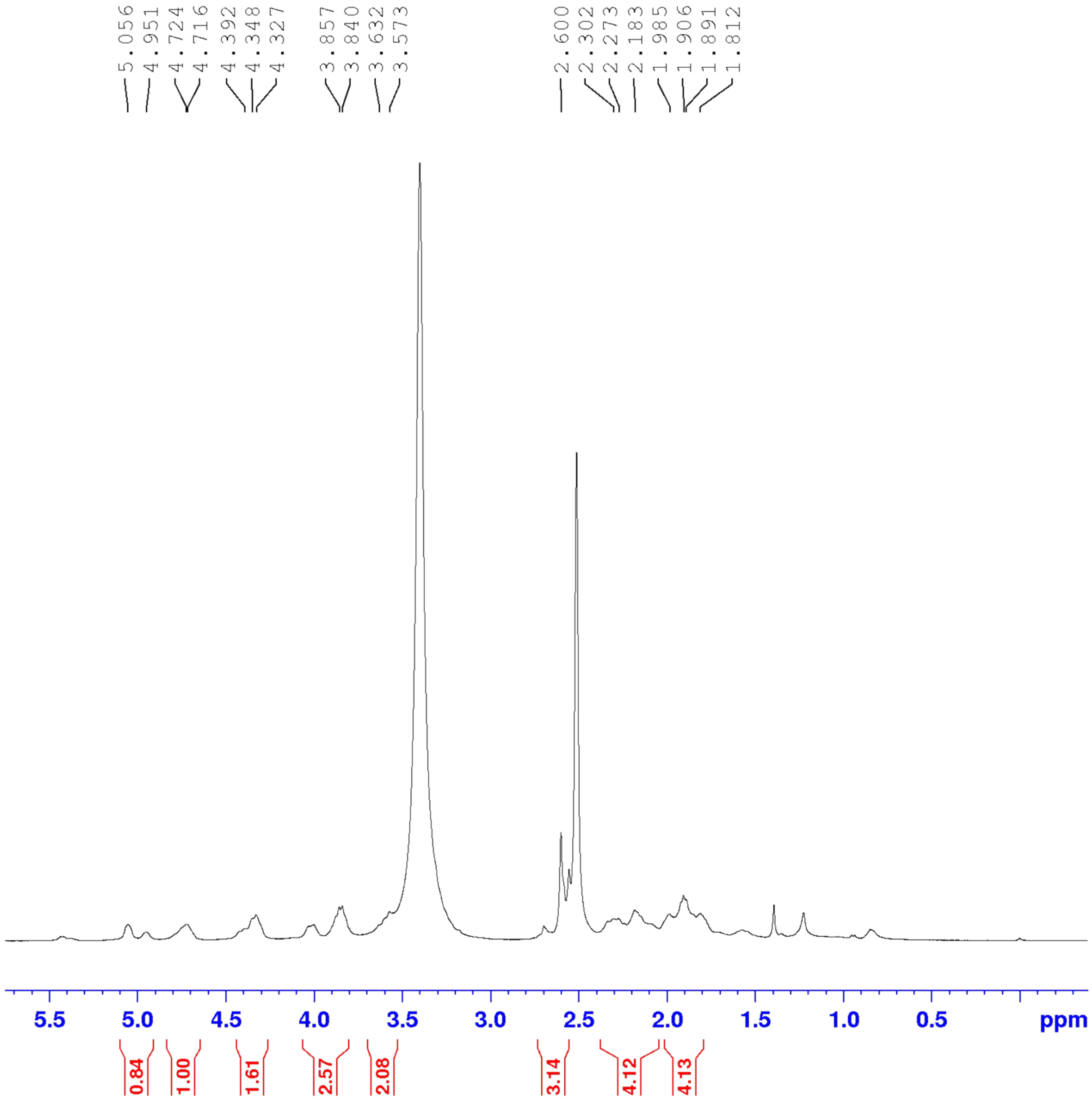

Figure 118. Aliphatic region of the ^{1}H NMR spectrum for **12t**

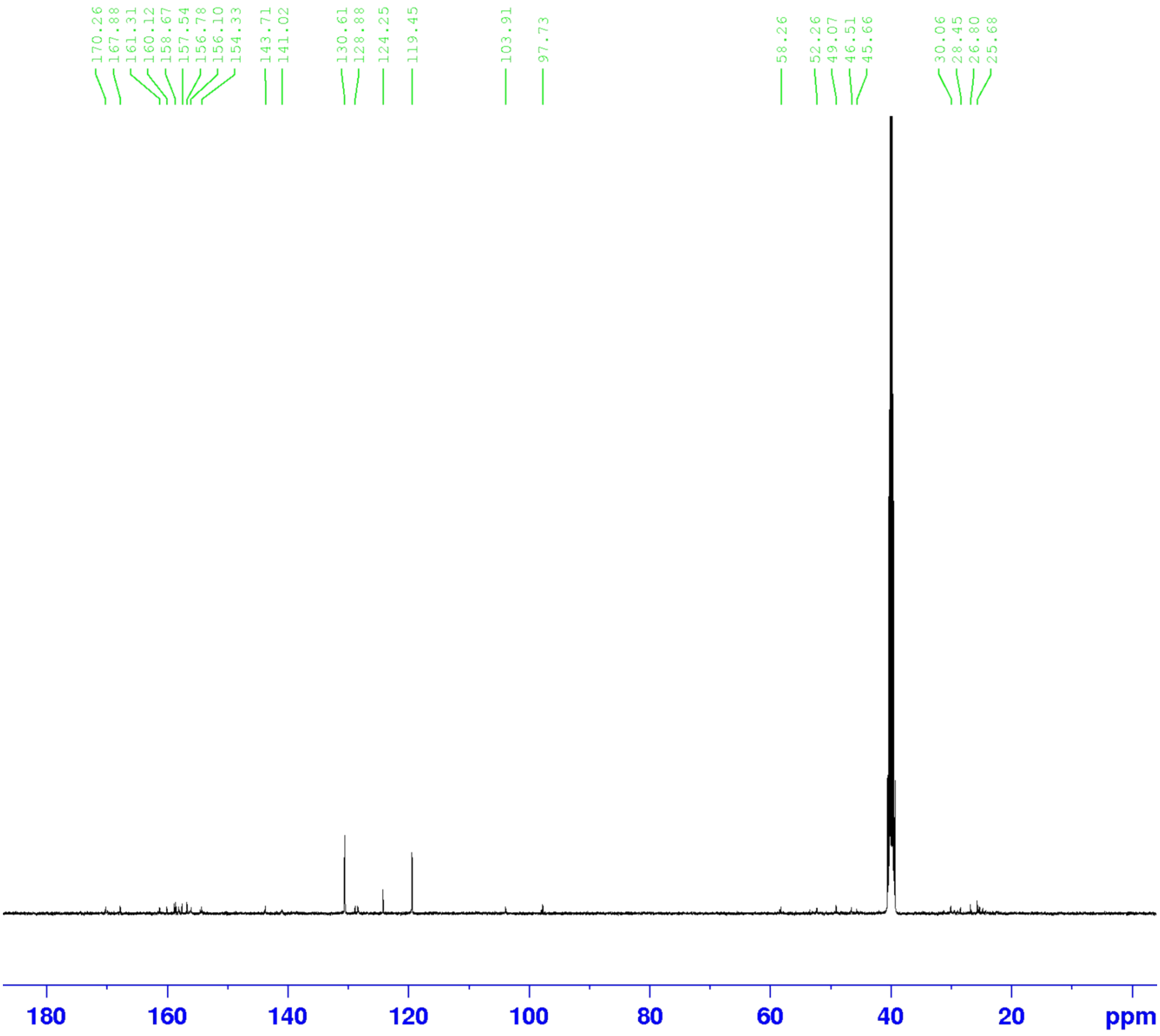

Figure 119. ^{13}C-NMR spectrum of **12t**

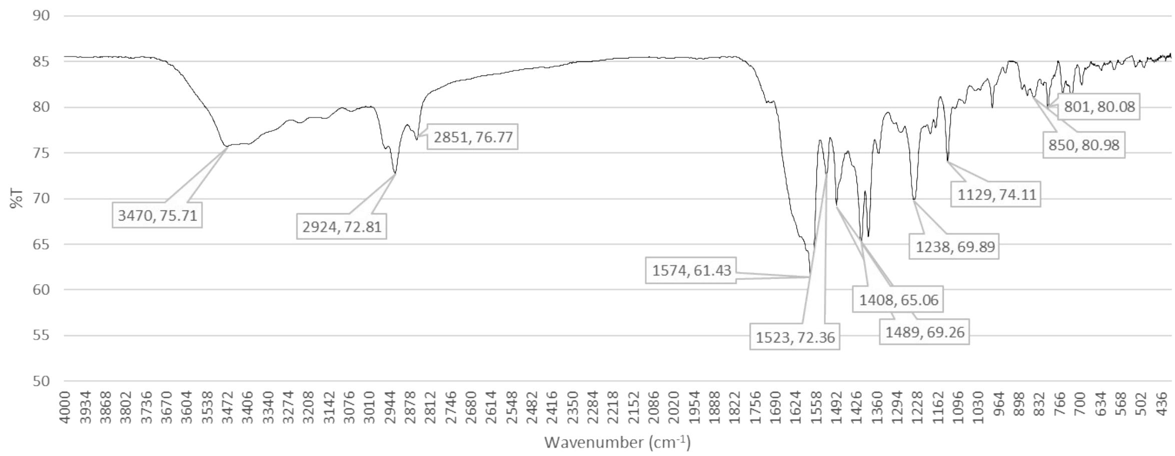

Figure 120. FT-IR spectrum of **12t**

Spectral data of **((S)-3-(4-amino-3-(3-phenoxyphenyl)-1H-pyrazolo[3,4-d]pyrimidin-1-yl)piperidin-1-yl)(pyrrolidin-3-yl)methanone (11)**

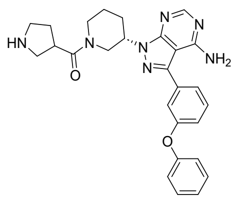

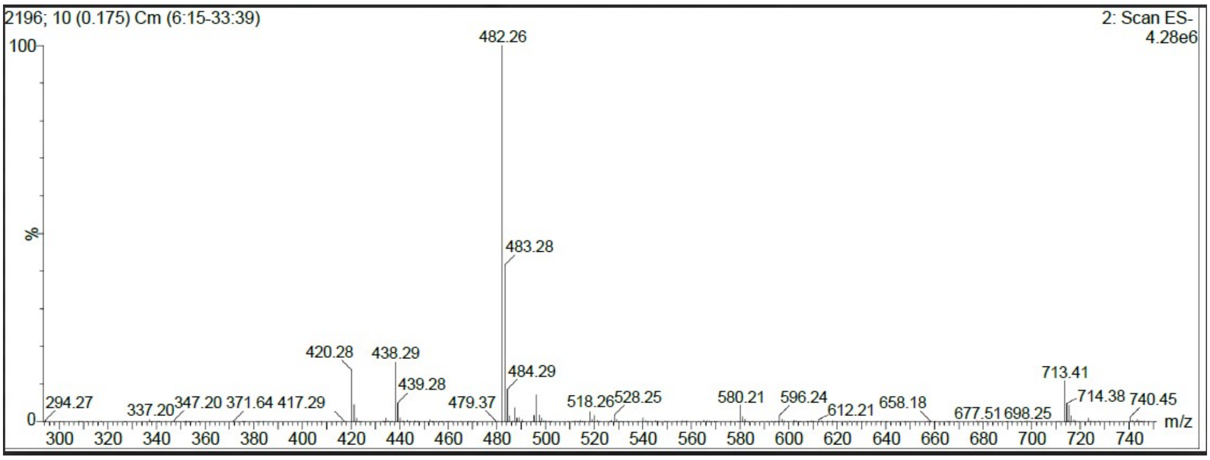

Figure 121. ESI-MS spectrum of **11**

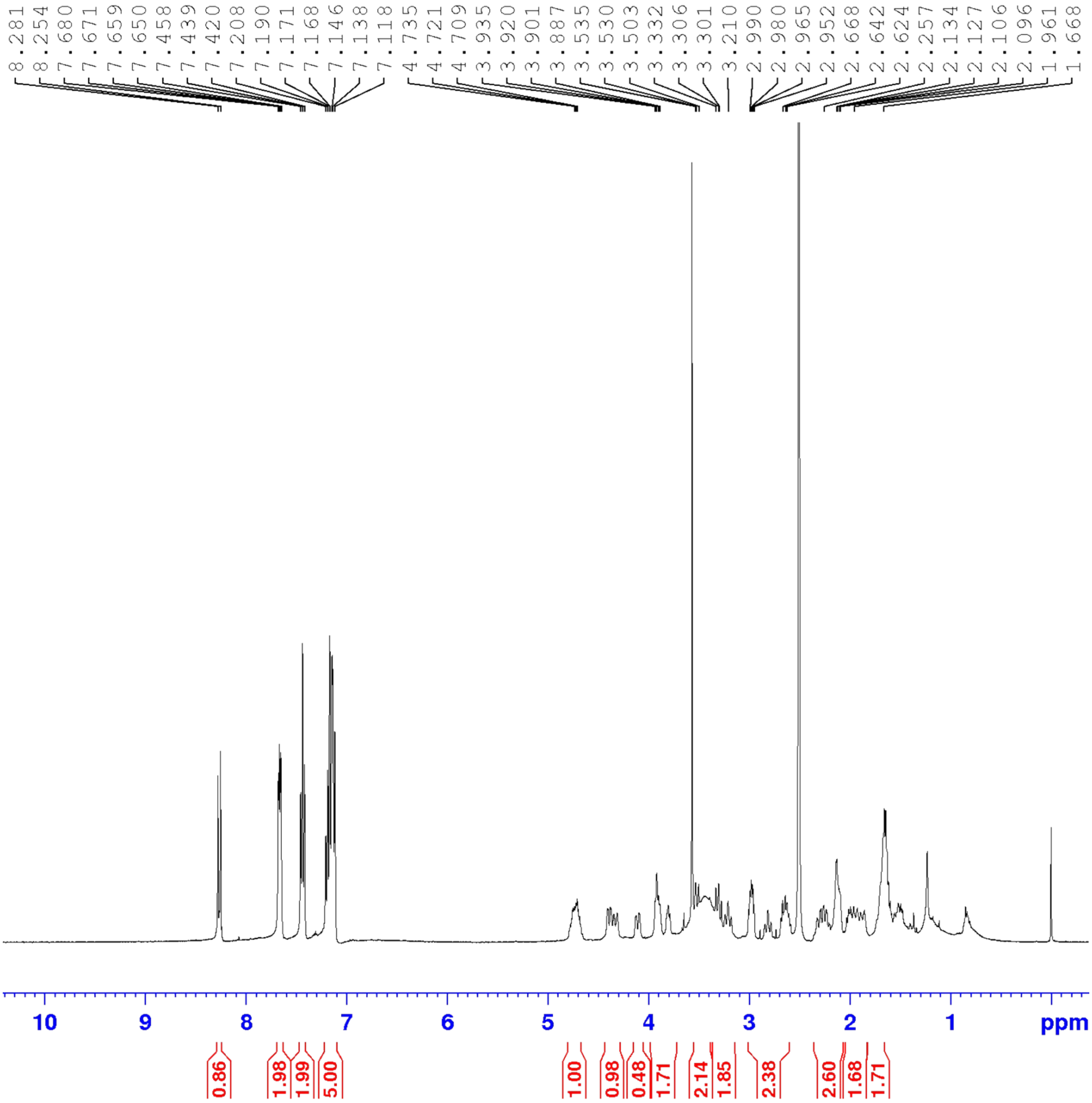

Figure 122. ^{1}H NMR full spectrum of **11**

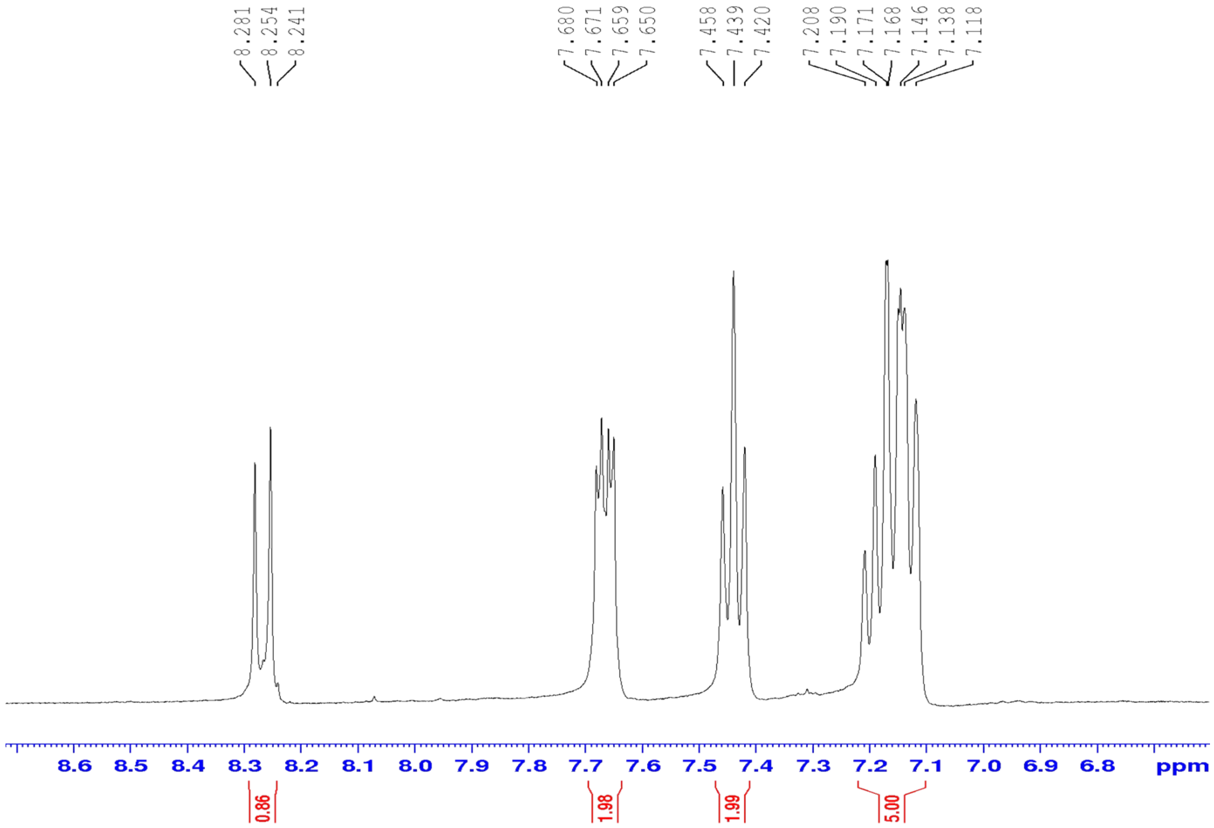

Figure 123. Aromatic region of the ^{1}H NMR spectrum for **11**

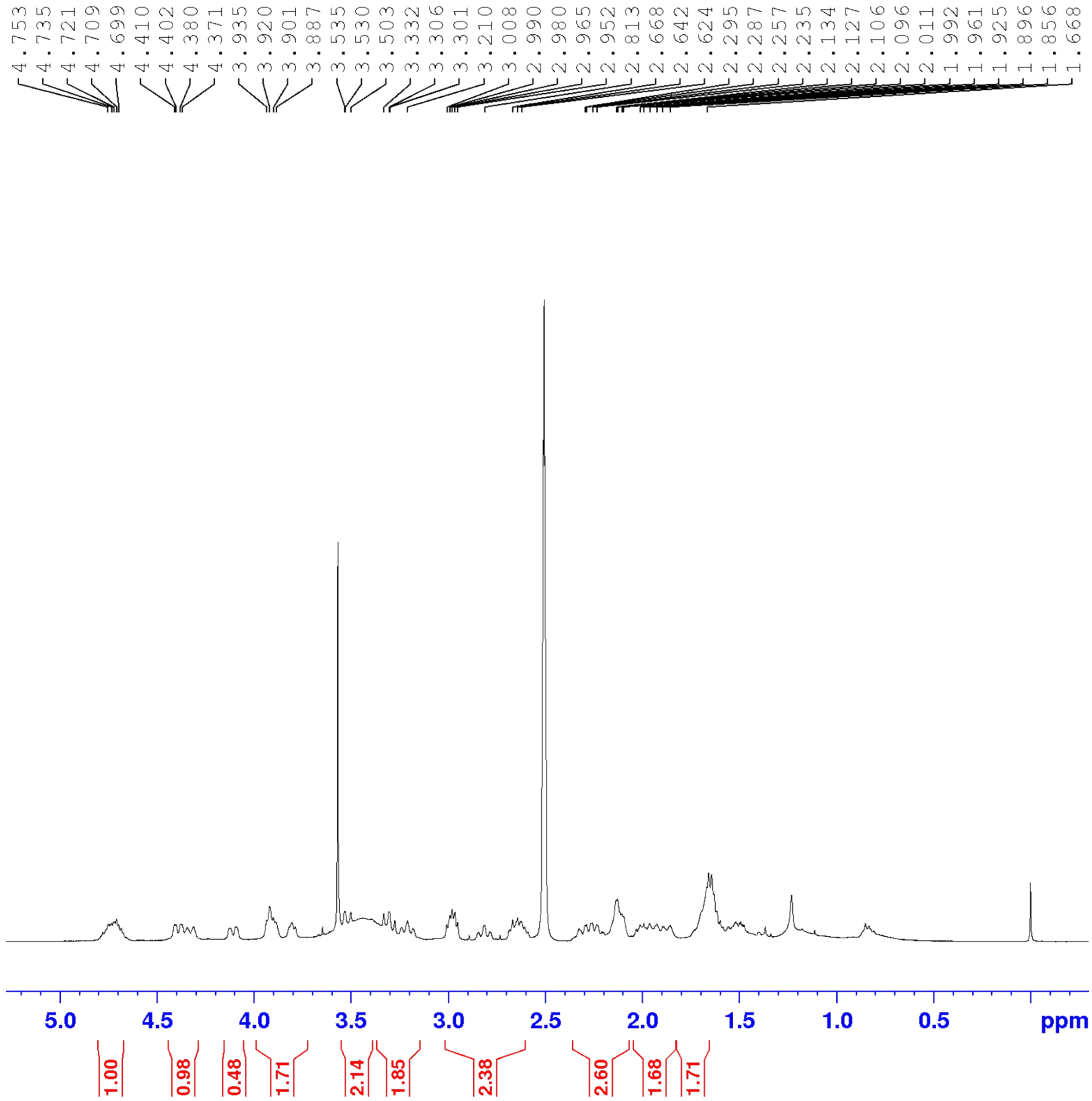

Figure 124. Aliphatic region of the ^{1}H NMR spectrum for **11**

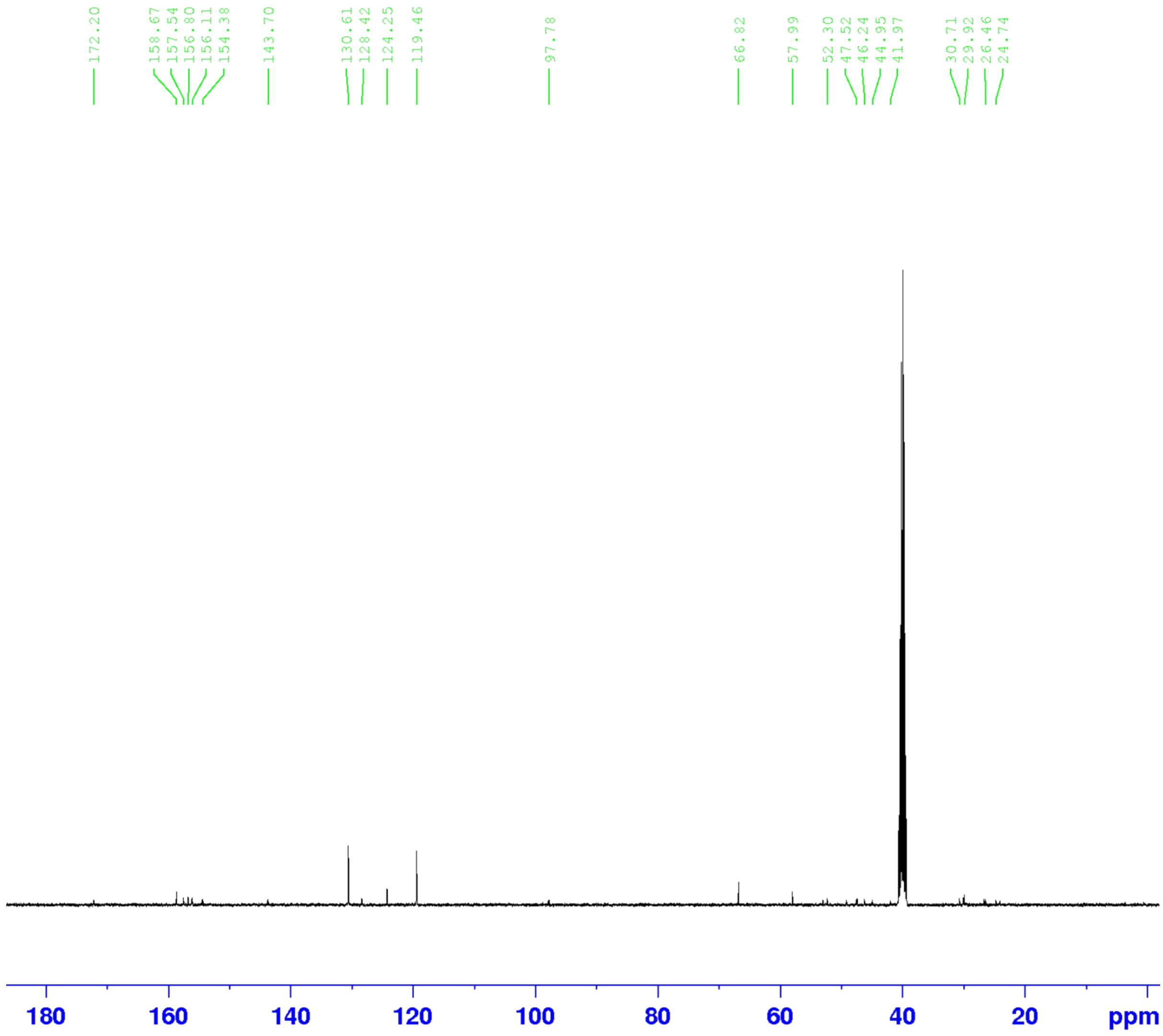

Figure 125. ^{13}C-NMR spectrum of **11**

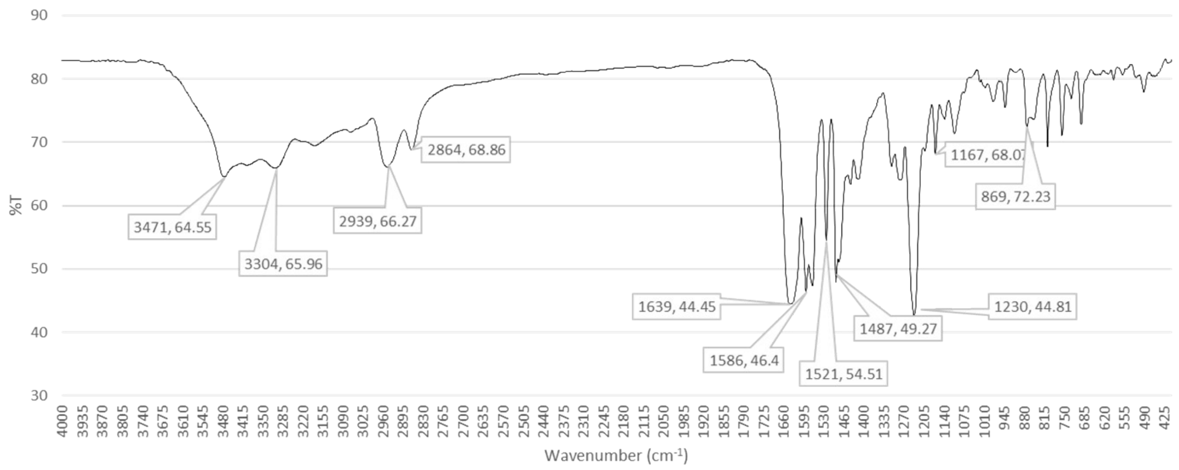

Figure 126. FT-IR spectrum of **11**

Spectral data of **tert-butyl 3-((S)-3-(4-amino-3-(3-phenoxyphenyl)-1H-pyrazolo[3,4-d]pyrimidin-1-yl)piperidine-1-carbonyl)pyrrolidine-1-carboxylate (10)**

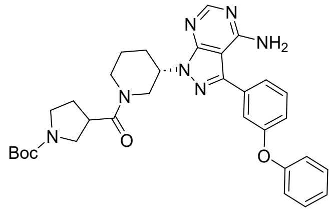

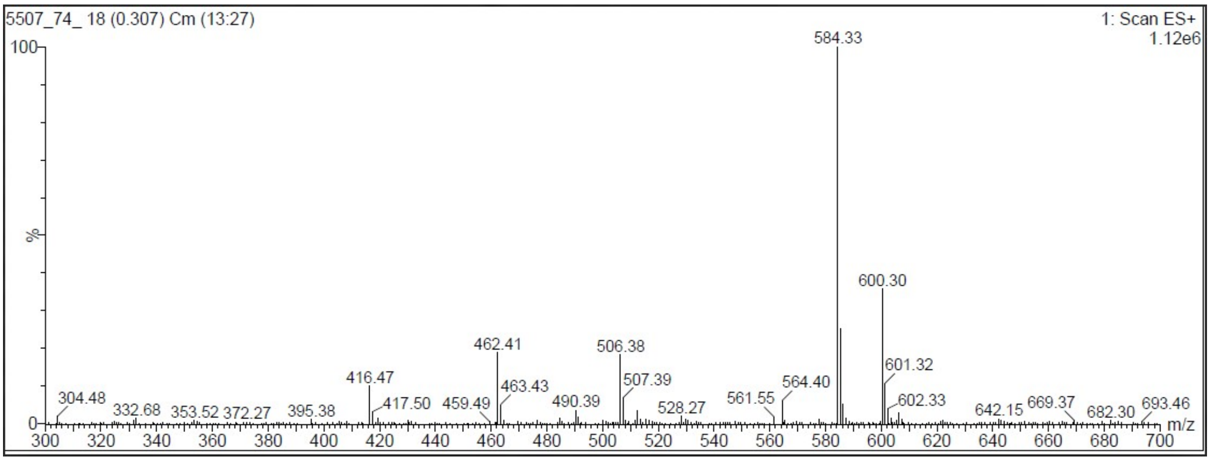

Figure 127. ESI-MS spectrum of **10**

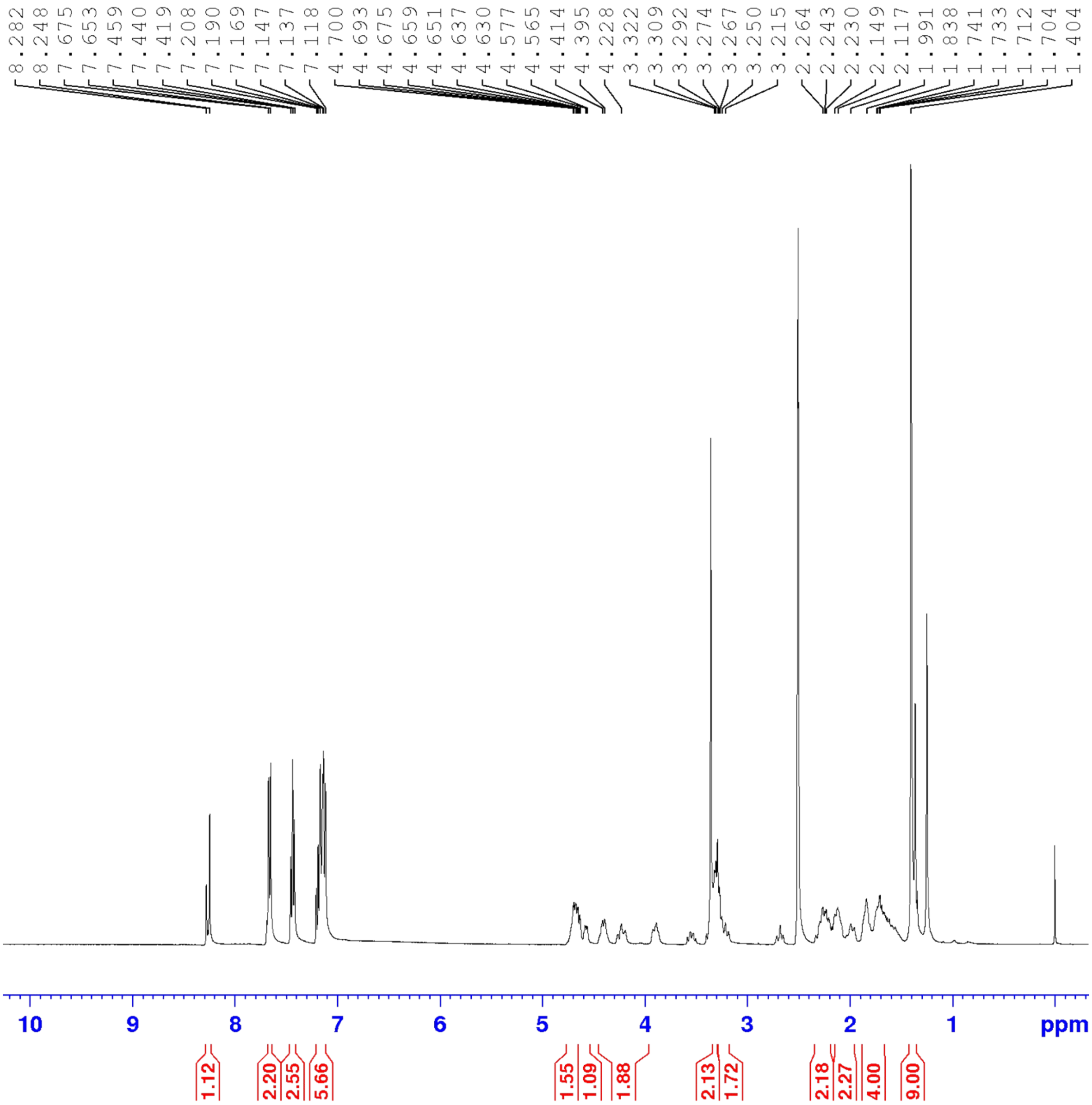

Figure 128. ^{1}H NMR spectrum of **10**

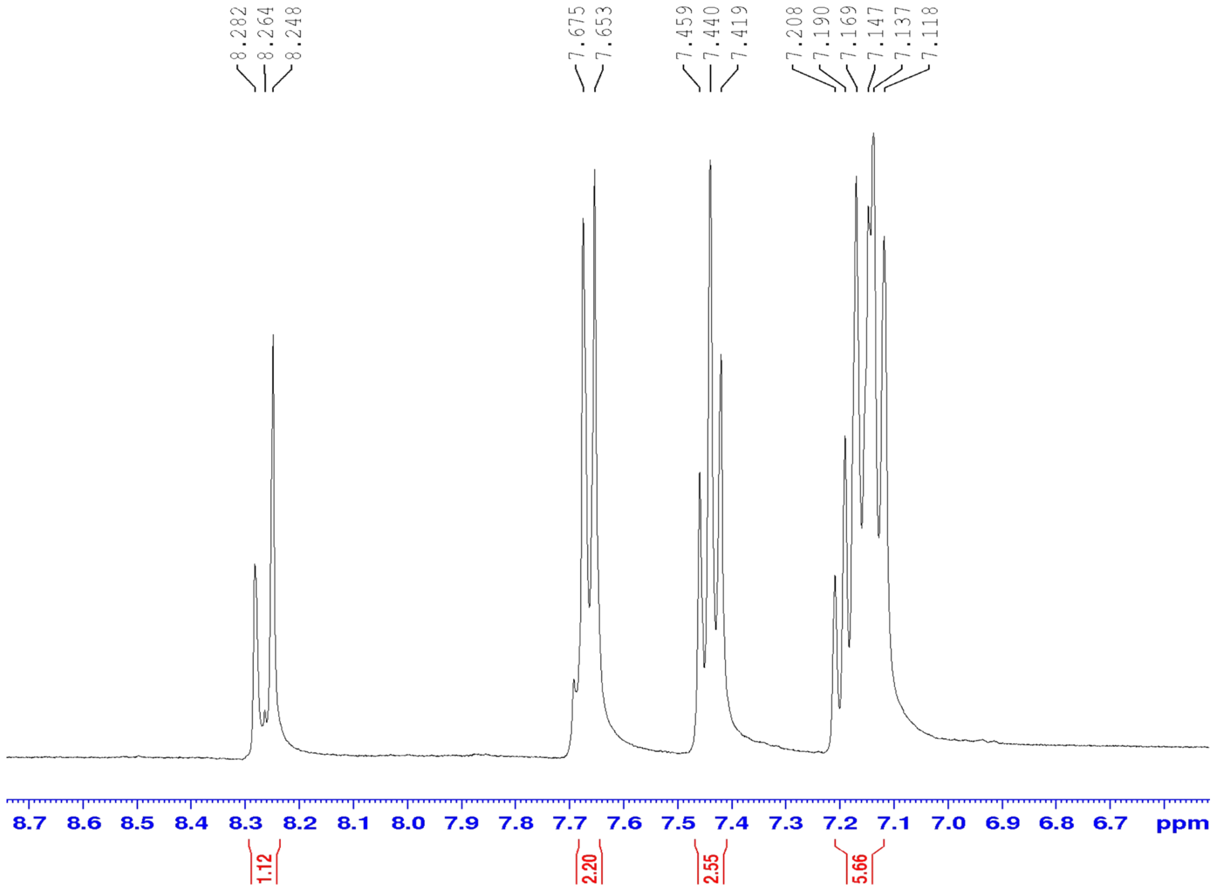

Figure 129. Aromatic region of the ^{1}H NMR spectrum for **10**

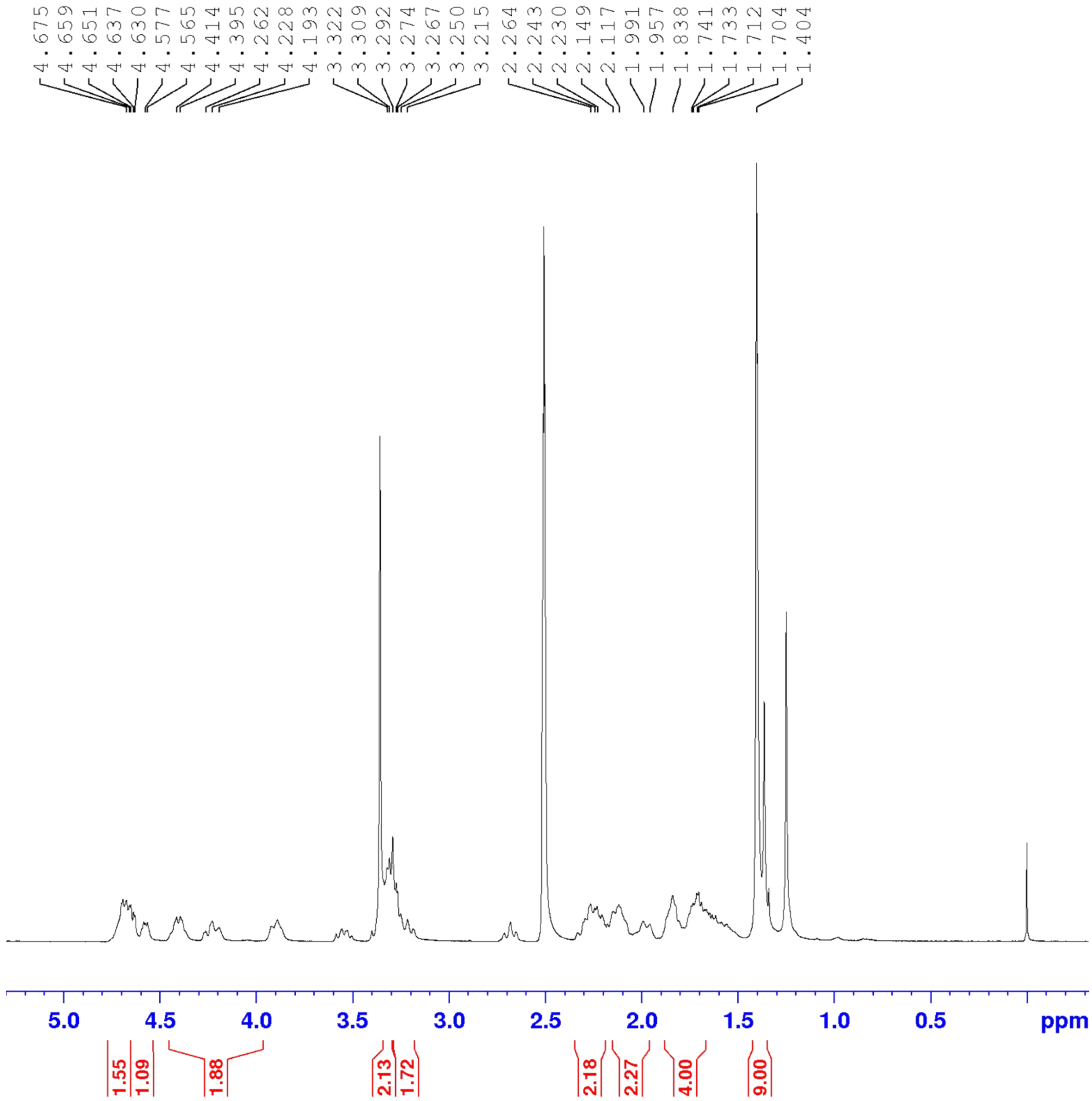

Figure 130. Aliphatic region of the ^{1}H NMR spectrum for **10**

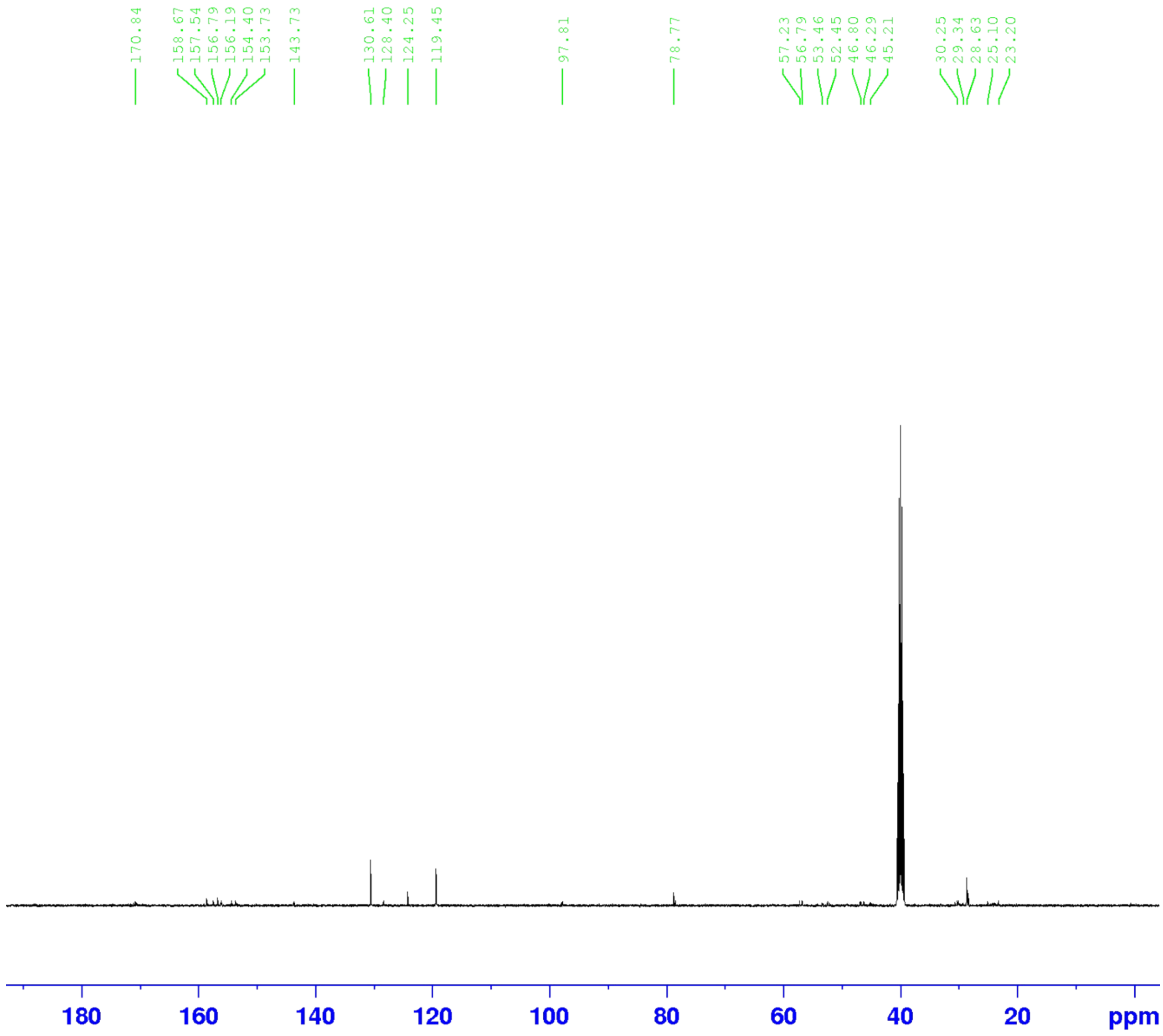

Figure 131. ^{13}C-NMR spectrum of **10**

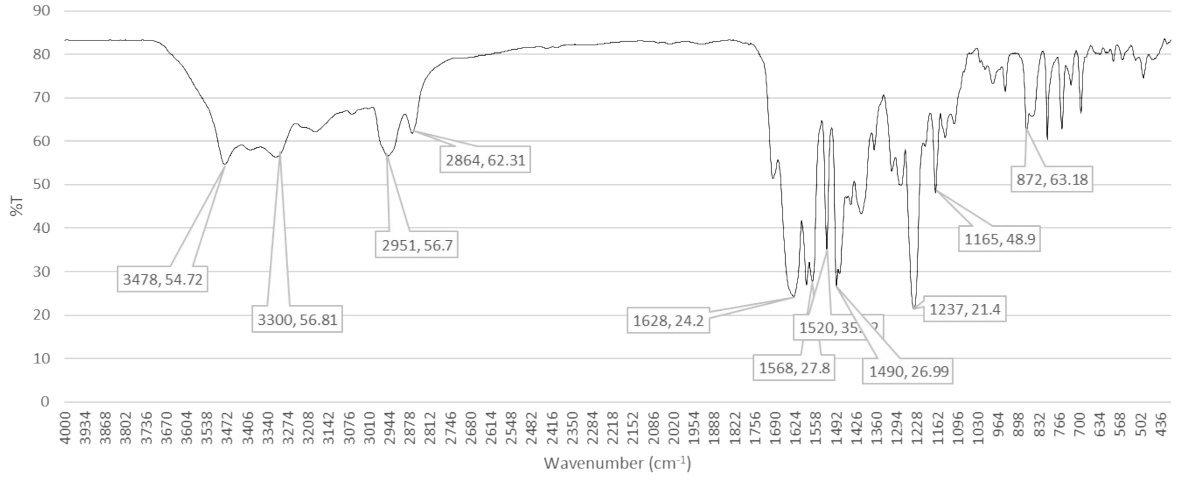

Figure 132. FT-IR spectrum of **10**

Spectral data of **(S)-3-(3-phenoxyphenyl)-1-(piperidin-3-yl)-1H-pyrazolo[3,4-d]pyrimidin-4-amine (8)**

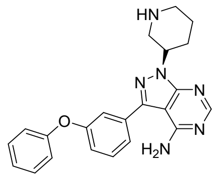

Figure 133. ESI-MS spectrum of **8**

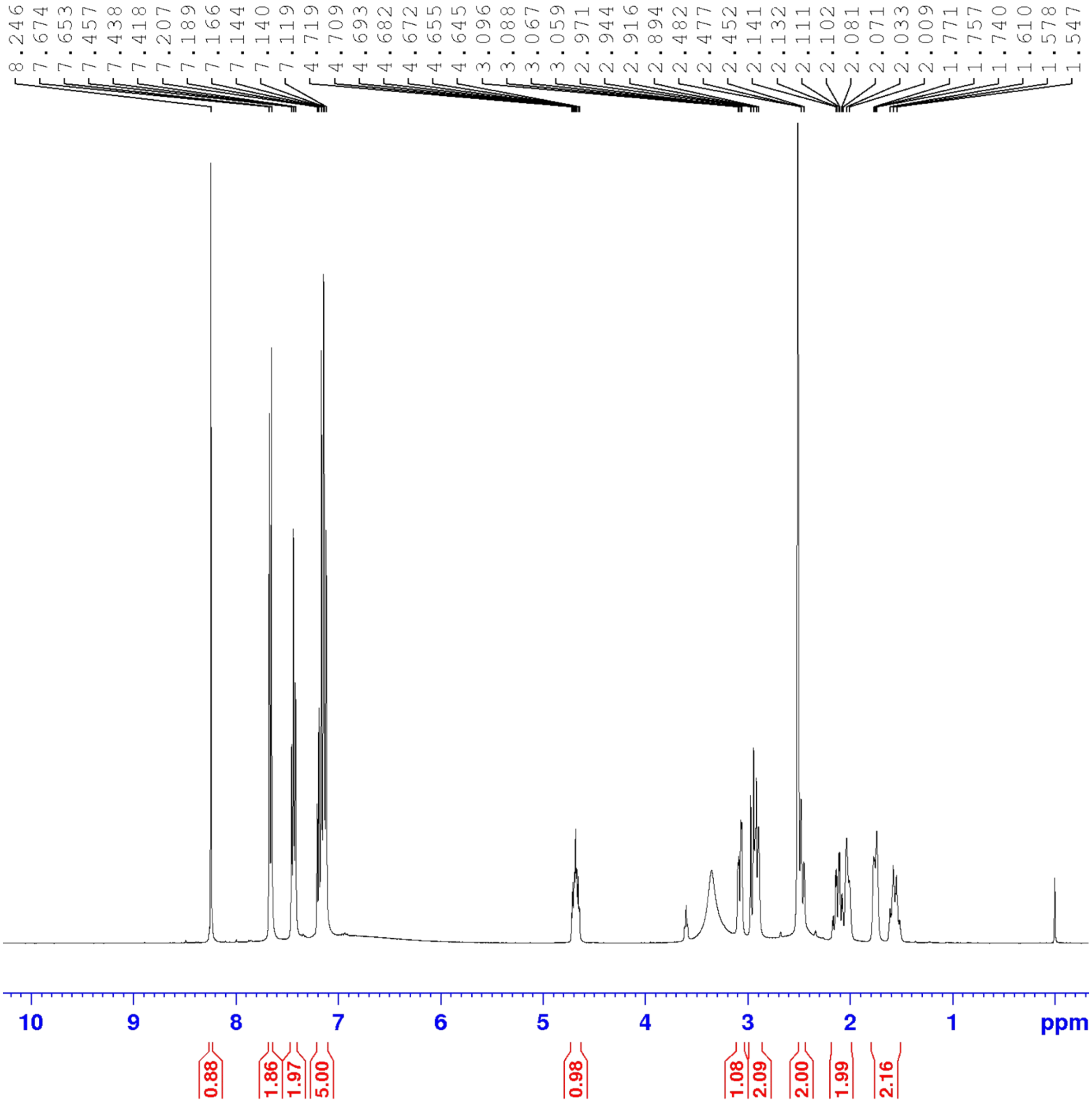

Figure 134. ^{1}H NMR full spectrum of **8**

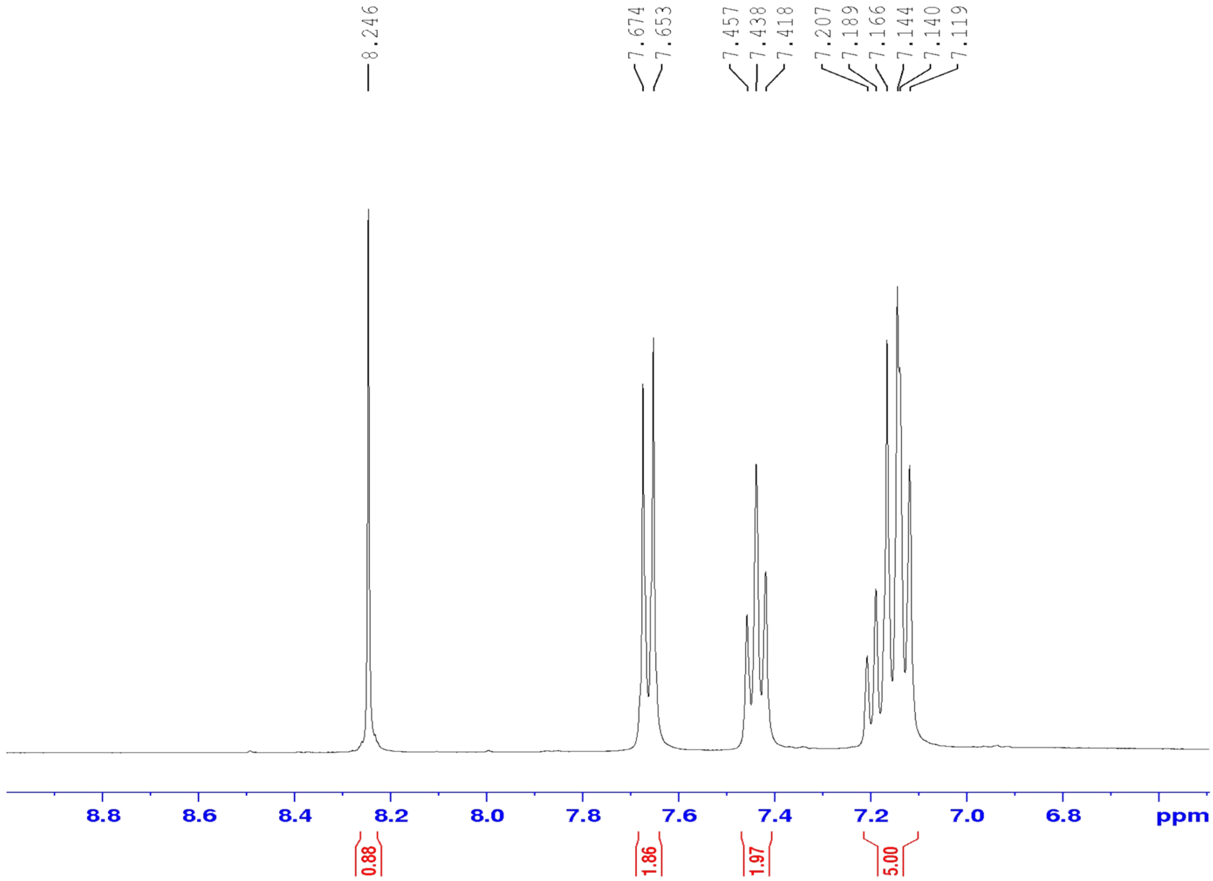

Figure 135. Aromatic region of the ^{1}H NMR spectrum for **8**

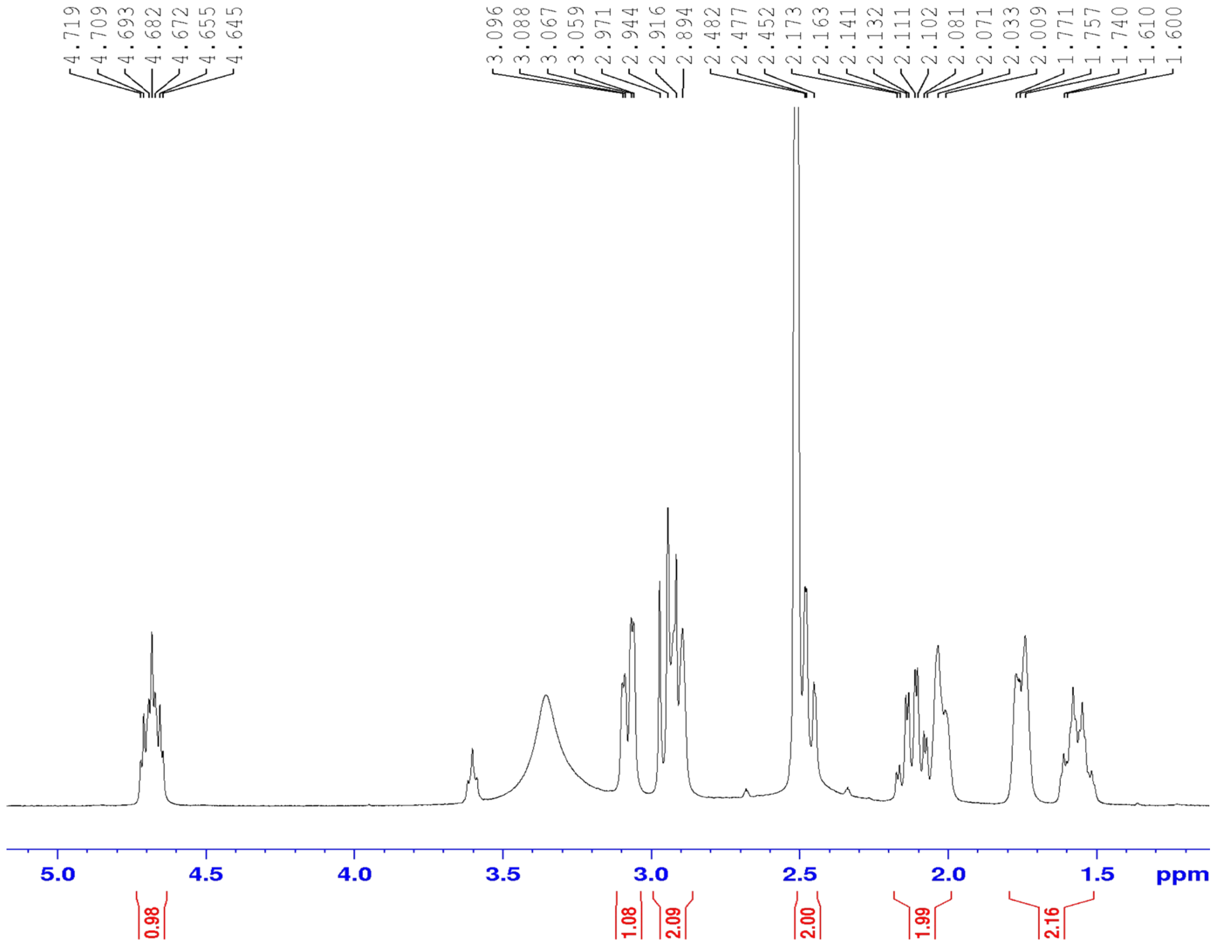

Figure 136. Aliphatic region of the ^{1}H NMR spectrum for **8**

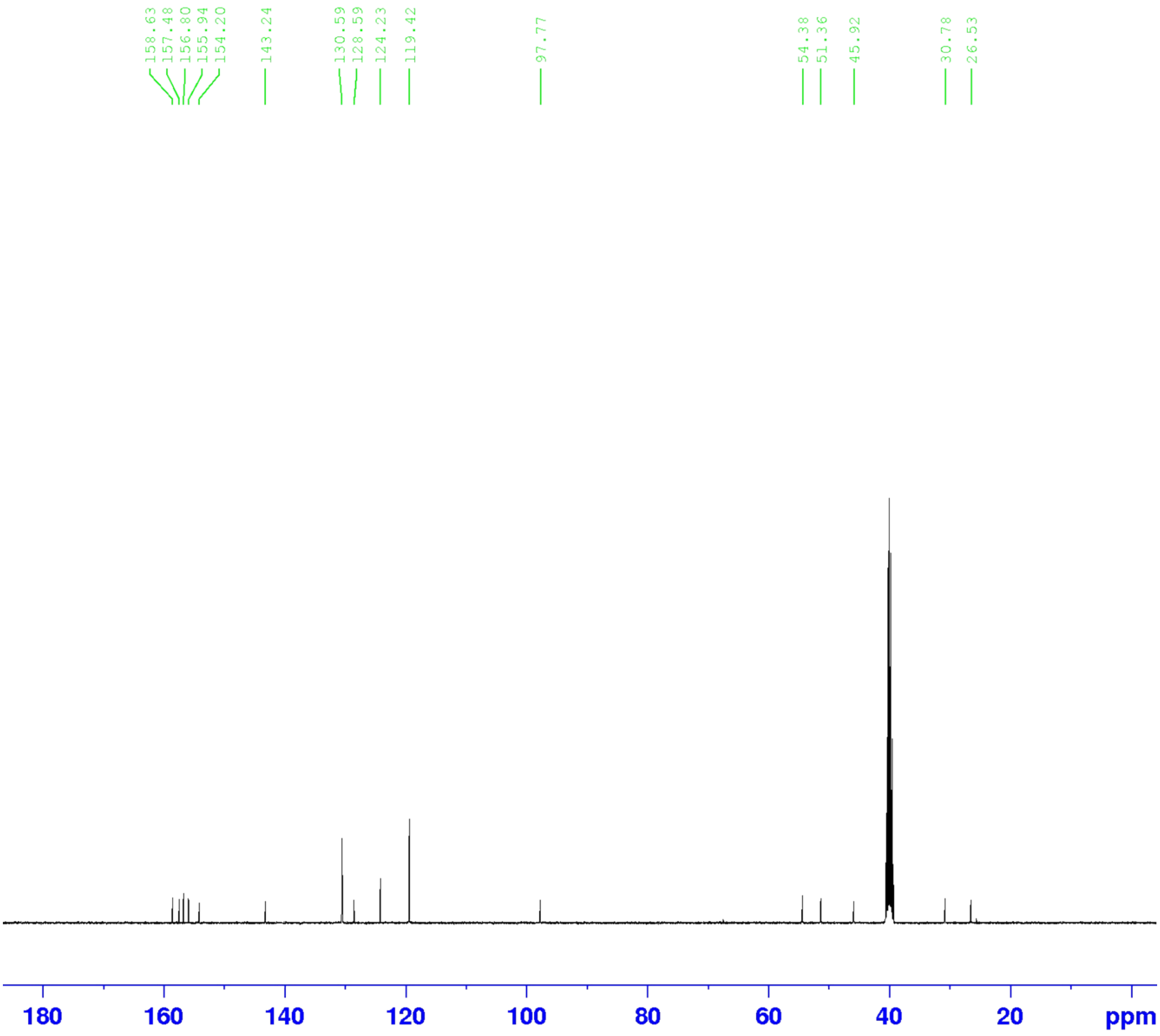

Figure 137. ^{13}C-NMR spectrum of **8**

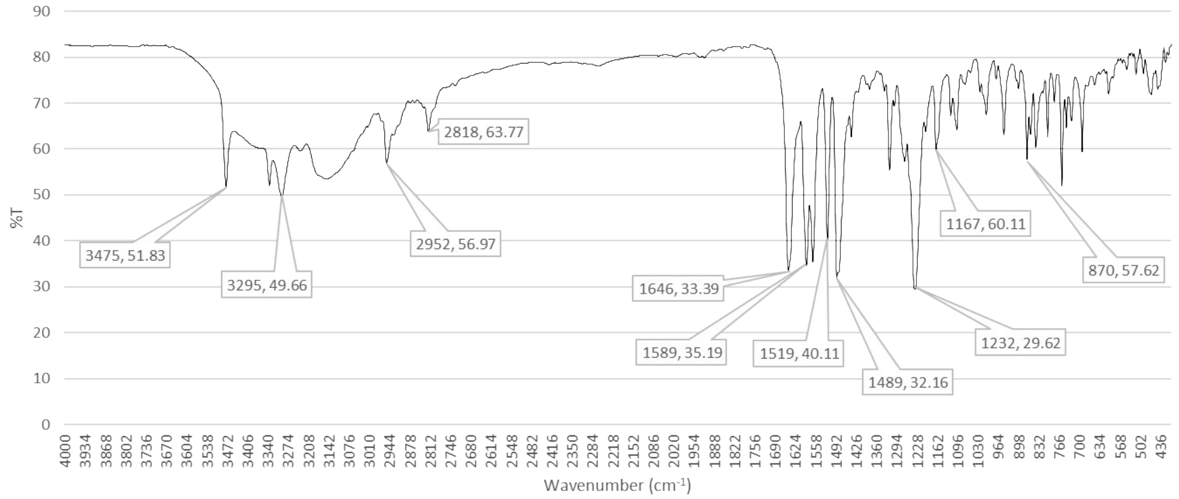

Figure 138. FT-IR spectrum of **8**

***In-vitro* anti-cancer activity against NCI 60 cell line panel – five dose study and NCI COMPARE. (Compound 12c)**

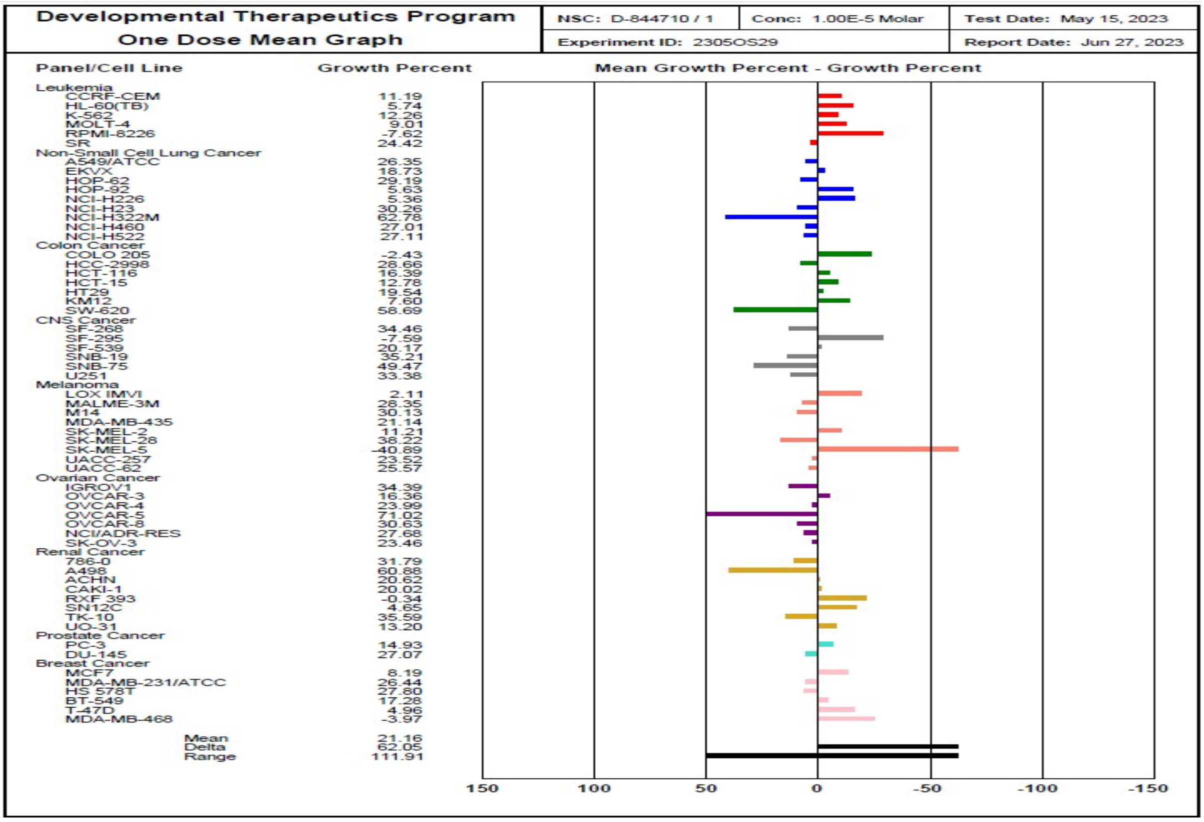

Figure 139. NCI report for growth inhibitory activity of sample **12c** at 10µM concentration

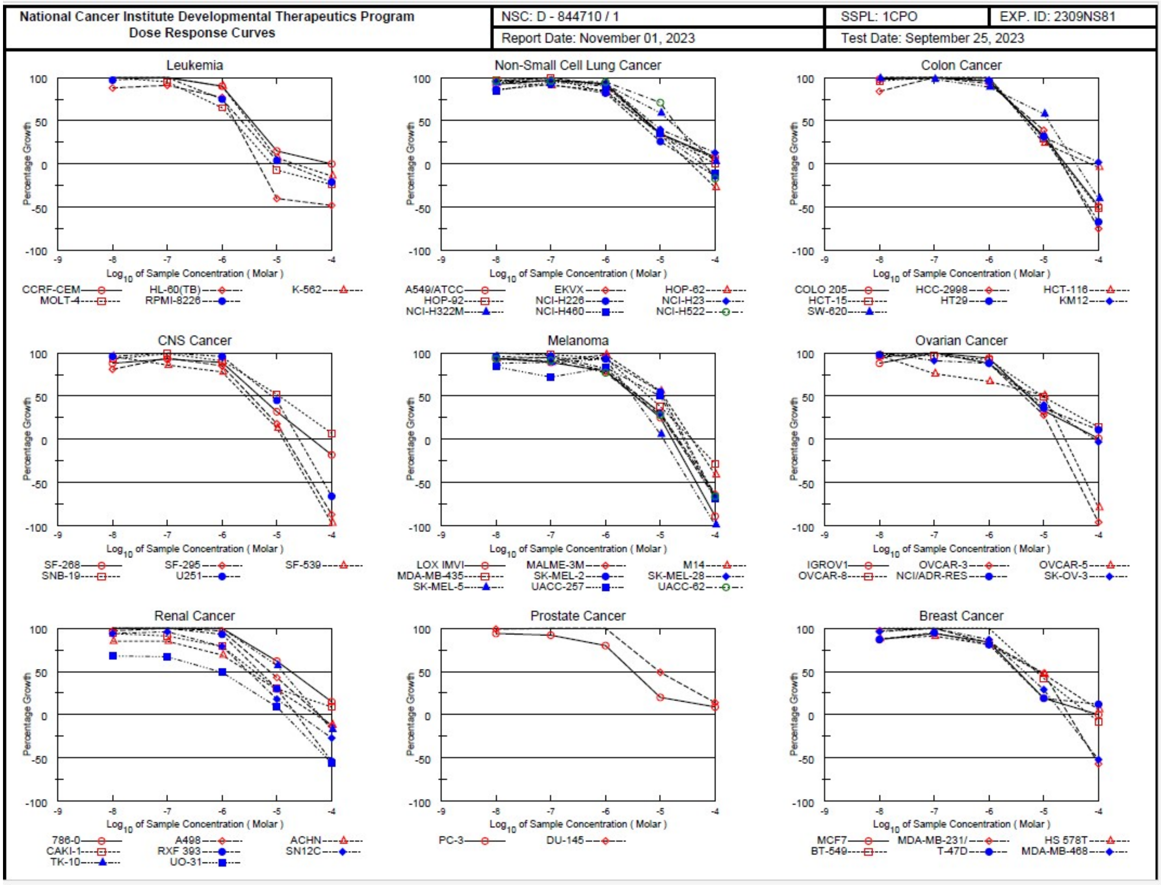

Figure 140. Dose response curves for compound **12c** in individual cell lines

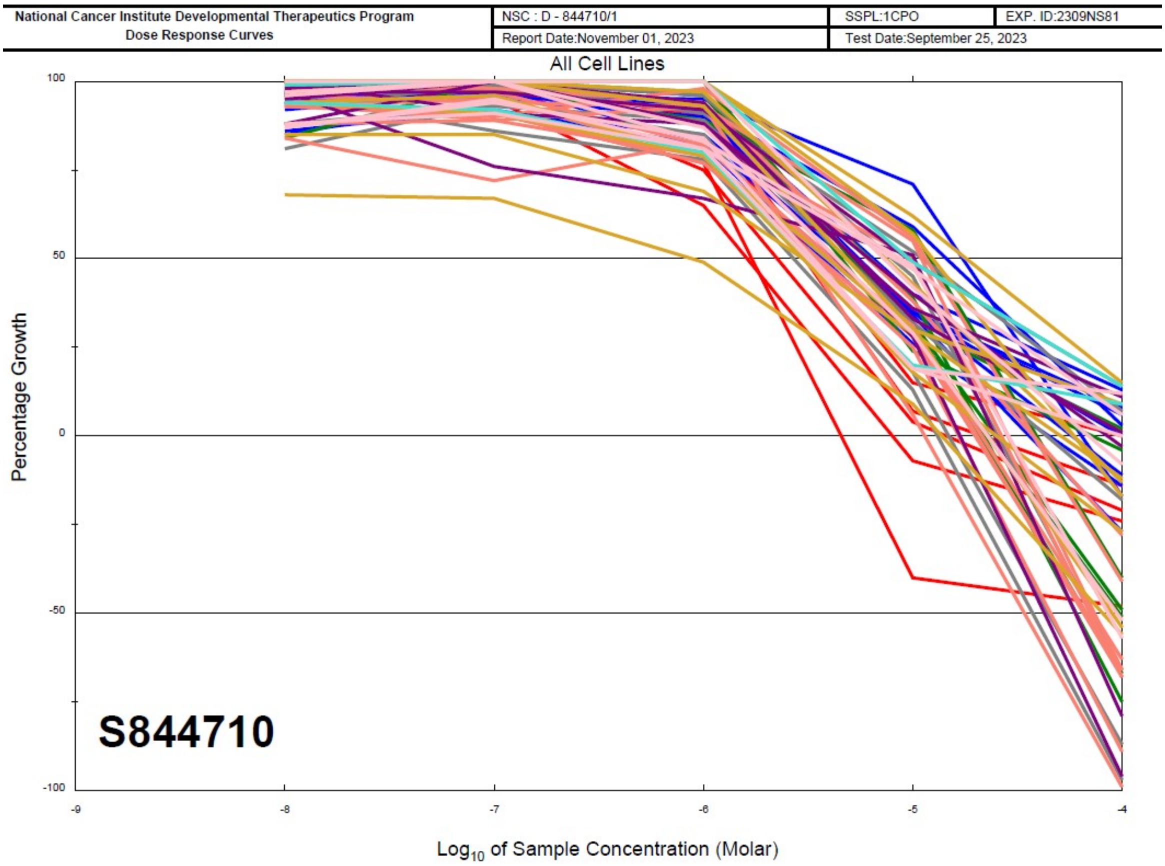

Figure 141. Dose response curves for compound **12c** in all cell lines

National Cancer Institute Developmental Therapeutics Program
In-Vitro Testing Results

NSC : D - 844710 / 1	Experiment ID : 2309NS81	Test Type : 08	Units : Molar
Report Date : November 01, 2023	Test Date : September 25, 2023	QNS :	MC :
COMI : 037	Stain Reagent : SRB Dual-Pass Related	SSPL : 1CPO	

			Log10 Concentration												
	Time		Mean Optical Densities					Percent Growth							
Panel/Cell Line	Zero	Ctrl	-8.0	-7.0	-6.0	-5.0	-4.0	-8.0	-7.0	-6.0	-5.0	-4.0	GI50	TGI	LC50
Leukemia															
CCRF-CEM	0.494	2.709	2.709	2.702	2.495	0.828	0.505	100	100	90	15	0	3.44E-6	> 1.00E-4	> 1.00E-4
HL-60(TB)	0.589	2.620	2.377	2.436	2.152	0.352	0.307	88	91	77	-40	-48	1.70E-6	4.53E-6	> 1.00E-4
K-562	0.236	2.277	2.372	2.332	2.064	0.384	0.203	105	103	90	7	-14	3.02E-6	2.20E-5	> 1.00E-4
MOLT-4	0.508	2.611	2.643	2.509	1.869	0.471	0.387	102	95	65	-7	-24	1.60E-6	7.92E-6	> 1.00E-4
RPMI-8226	0.797	2.610	2.553	2.612	2.162	0.866	0.634	97	100	75	4	-21	2.26E-6	1.43E-5	> 1.00E-4
Non-Small Cell Lung Cancer															
A549/ATCC	0.421	2.352	2.189	2.302	2.211	1.089	0.582	92	97	93	35	8	5.43E-6	> 1.00E-4	> 1.00E-4
EKVX	0.945	2.328	2.266	2.330	2.204	1.429	1.033	96	100	91	35	6	5.39E-6	> 1.00E-4	> 1.00E-4
HOP-62	0.538	1.908	1.824	1.789	1.705	1.007	0.394	94	91	85	34	-27	4.90E-6	3.63E-5	> 1.00E-4
HOP-92	0.834	1.612	1.592	1.606	1.483	1.105	0.833	97	99	83	35	0	4.86E-6	9.88E-5	> 1.00E-4
NCI-H226	1.201	2.411	2.245	2.313	2.191	1.521	1.032	86	92	82	26	-14	3.75E-6	4.48E-5	> 1.00E-4
NCI-H23	0.595	2.080	2.012	2.040	1.973	1.187	0.788	95	97	93	40	13	6.44E-6	> 1.00E-4	> 1.00E-4
NCI-H322M	0.916	2.506	2.410	2.436	2.376	1.847	0.962	94	96	92	59	3	1.42E-5	> 1.00E-4	> 1.00E-4
NCI-H460	0.288	2.644	2.288	2.530	2.397	1.124	0.257	85	95	90	35	-11	5.38E-6	5.85E-5	> 1.00E-4
NCI-H522	2.068	3.388	3.331	3.336	3.322	3.003	1.722	96	96	95	71	-17	1.73E-5	6.44E-5	> 1.00E-4
Colon Cancer															
COLO 205	0.645	2.754	2.705	2.777	2.664	1.316	0.328	98	101	96	32	-49	5.19E-6	2.47E-5	> 1.00E-4
HCC-2998	0.669	2.643	2.320	2.638	2.509	1.431	0.170	84	100	93	39	-75	6.18E-6	2.19E-5	6.06E-5
HCT-116	0.251	2.422	2.464	2.633	2.511	0.782	0.240	102	110	104	24	-4	4.78E-6	7.05E-5	> 1.00E-4
HCT-15	0.409	2.779	2.694	2.793	2.769	1.087	0.202	96	101	100	29	-51	4.99E-6	2.29E-5	9.79E-5
HT29	0.402	2.541	2.682	2.545	2.463	1.089	0.131	107	100	96	32	-67	5.27E-6	2.10E-5	6.68E-5
KM12	0.767	3.254	3.263	3.273	3.180	1.548	0.827	100	101	97	31	2	5.20E-6	> 1.00E-4	> 1.00E-4
SW-620	0.336	2.042	2.024	2.011	1.850	1.329	0.202	99	98	89	58	-40	1.21E-5	3.92E-5	> 1.00E-4
CNS Cancer															
SF-268	0.793	2.277	2.092	2.167	2.118	1.265	0.649	88	93	89	32	-18	4.82E-6	4.32E-5	> 1.00E-4
SF-295	0.734	2.481	2.152	2.374	2.226	1.057	0.094	81	94	85	18	-87	3.38E-6	1.50E-5	4.44E-5
SF-539	0.969	2.670	2.600	2.438	2.300	1.184	0.025	96	86	78	13	-97	2.69E-6	1.30E-5	3.71E-5
SNB-19	0.609	2.164	2.061	2.143	2.020	1.417	0.724	93	99	91	52	7	1.11E-5	> 1.00E-4	> 1.00E-4
U251	0.390	1.765	1.708	1.763	1.706	1.005	0.132	96	100	96	45	-66	7.88E-6	2.53E-5	7.15E-5
Melanoma															
LOX IMVI	0.390	2.408	2.291	2.180	1.986	0.898	0.041	94	89	79	25	-89	3.46E-6	1.66E-5	4.52E-5
MALME-3M	0.698	1.425	1.376	1.391	1.272	0.921	0.255	93	95	79	31	-63	3.97E-6	2.12E-5	7.19E-5
M14	0.488	1.935	1.830	1.797	1.901	1.292	0.289	93	90	98	56	-41	1.14E-5	3.77E-5	> 1.00E-4
MDA-MB-435	0.589	2.346	2.359	2.319	2.235	1.262	0.422	101	98	94	38	-28	6.15E-6	3.76E-5	> 1.00E-4
SK-MEL-2	1.482	2.874	2.817	2.785	2.782	2.246	0.498	96	94	93	55	-66	1.10E-5	2.83E-5	7.32E-5
SK-MEL-28	0.408	1.441	1.477	1.405	1.257	0.716	0.130	104	97	82	30	-68	4.12E-6	2.02E-5	6.53E-5
SK-MEL-5	1.218	3.323	3.076	3.098	3.176	1.340	0.015	88	89	93	6	-99	3.11E-6	1.14E-5	3.41E-5
UACC-257	1.356	2.780	2.547	2.381	2.544	2.071	0.431	84	72	83	50	-68	1.00E-5	2.65E-5	7.02E-5
UACC-62	0.888	2.966	2.820	2.773	2.491	1.479	0.302	93	91	77	28	-66	3.61E-6	2.00E-5	6.76E-5
Ovarian Cancer															
IGROV1	0.805	2.519	2.313	2.523	2.424	1.367	0.824	88	100	94	33	1	5.26E-6	> 1.00E-4	> 1.00E-4
OVCAR-3	0.583	1.890	1.831	1.894	1.738	0.953	0.023	95	100	88	28	-96	4.35E-6	1.69E-5	4.26E-5
OVCAR-5	0.675	1.571	1.544	1.356	1.280	1.133	0.143	97	76	67	51	-79	1.02E-5	2.47E-5	6.00E-5
OVCAR-8	0.556	2.563	2.499	2.508	2.405	1.539	0.840	97	97	92	49	14	9.47E-6	> 1.00E-4	> 1.00E-4
NCI/ADR-RES	0.477	1.706	1.680	1.706	1.554	0.920	0.609	98	100	88	36	11	5.36E-6	> 1.00E-4	> 1.00E-4
SK-OV-3	0.834	1.938	1.954	1.842	1.809	1.278	0.807	101	91	88	40	-3	6.26E-6	8.40E-5	> 1.00E-4
Renal Cancer															
786-0	0.639	2.852	2.904	2.973	2.856	2.019	0.976	102	105	100	62	15	1.83E-5	> 1.00E-4	> 1.00E-4
A498	1.292	2.242	2.218	2.296	2.210	1.702	1.127	97	106	97	43	-13	7.45E-6	5.90E-5	> 1.00E-4
ACHN	0.288	1.374	1.214	1.208	1.035	0.602	0.253	85	85	69	29	-12	2.95E-6	5.02E-5	> 1.00E-4
CAKI-1	0.603	2.459	2.349	2.301	2.072	1.157	0.775	94	91	79	30	9	3.90E-6	> 1.00E-4	> 1.00E-4
RXF 393	0.815	1.577	1.596	1.640	1.526	1.044	0.379	103	108	93	30	-54	4.84E-6	2.29E-5	9.07E-5
SN12C	0.457	2.074	1.977	2.003	1.733	0.752	0.336	94	96	79	18	-27	3.00E-6	2.55E-5	> 1.00E-4
TK-10	1.329	2.169	2.173	2.308	2.571	1.810	1.104	101	117	148	57	-17	1.25E-5	5.91E-5	> 1.00E-4
UO-31	0.668	2.149	1.682	1.658	1.392	0.796	0.297	68	67	49	9	-56	8.64E-7	1.36E-5	8.20E-5
Prostate Cancer															
PC-3	0.559	2.258	2.157	2.130	1.918	0.901	0.706	94	92	80	20	9	3.17E-6	> 1.00E-4	> 1.00E-4
DU-145	0.359	1.639	1.630	1.656	1.702	0.980	0.541	99	101	105	49	14	9.41E-6	> 1.00E-4	> 1.00E-4
Breast Cancer															
MCF7	0.625	2.614	2.352	2.491	2.303	1.000	0.630	87	94	84	19	0	3.34E-6	> 1.00E-4	> 1.00E-4
MDA-MB-231/ATCC	0.495	1.176	1.156	1.175	1.061	0.824	0.211	97	100	83	48	-57	8.89E-6	2.86E-5	8.51E-5
HS 578T	0.868	2.112	1.965	1.995	1.876	1.447	0.947	88	91	81	47	6	7.93E-6	> 1.00E-4	> 1.00E-4
BT-549	1.079	2.435	2.465	2.581	2.459	1.647	0.990	102	111	102	42	-8	7.31E-6	6.84E-5	> 1.00E-4
T-47D	0.750	1.964	1.803	1.905	1.737	0.977	0.894	87	95	81	19	12	3.16E-6	> 1.00E-4	> 1.00E-4
MDA-MB-468	0.959	2.002	1.965	2.028	1.863	1.262	0.461	96	103	87	29	-52	4.33E-6	2.28E-5	9.47E-5

Figure 142. In-vitro five dose testing results for compound **12c**

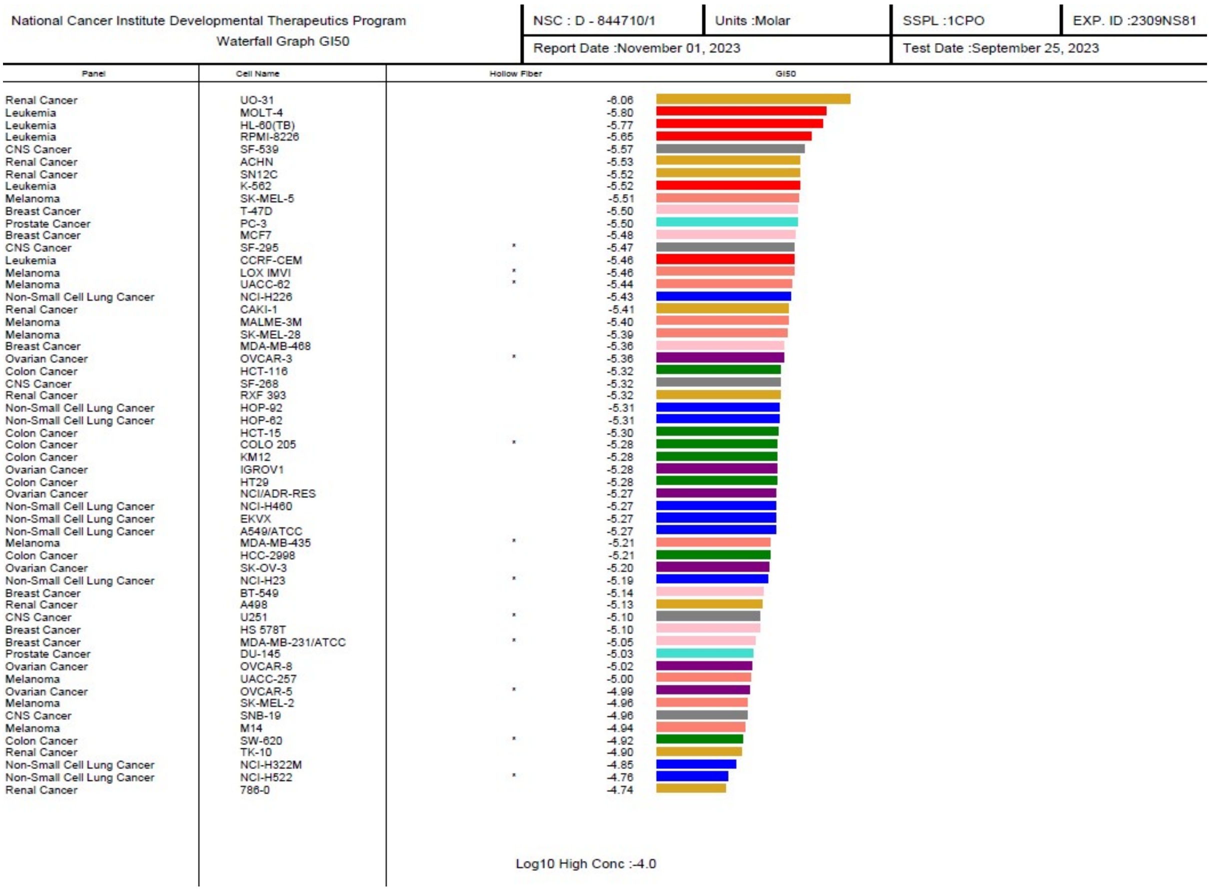

Figure 143. IC$_{50}$ values depicted in the waterfall graph for compound **12c**

In-vitro **anti-cancer activity against NCI 60 cell line panel – five dose study and NCI COMPARE. (Compound 12d)**

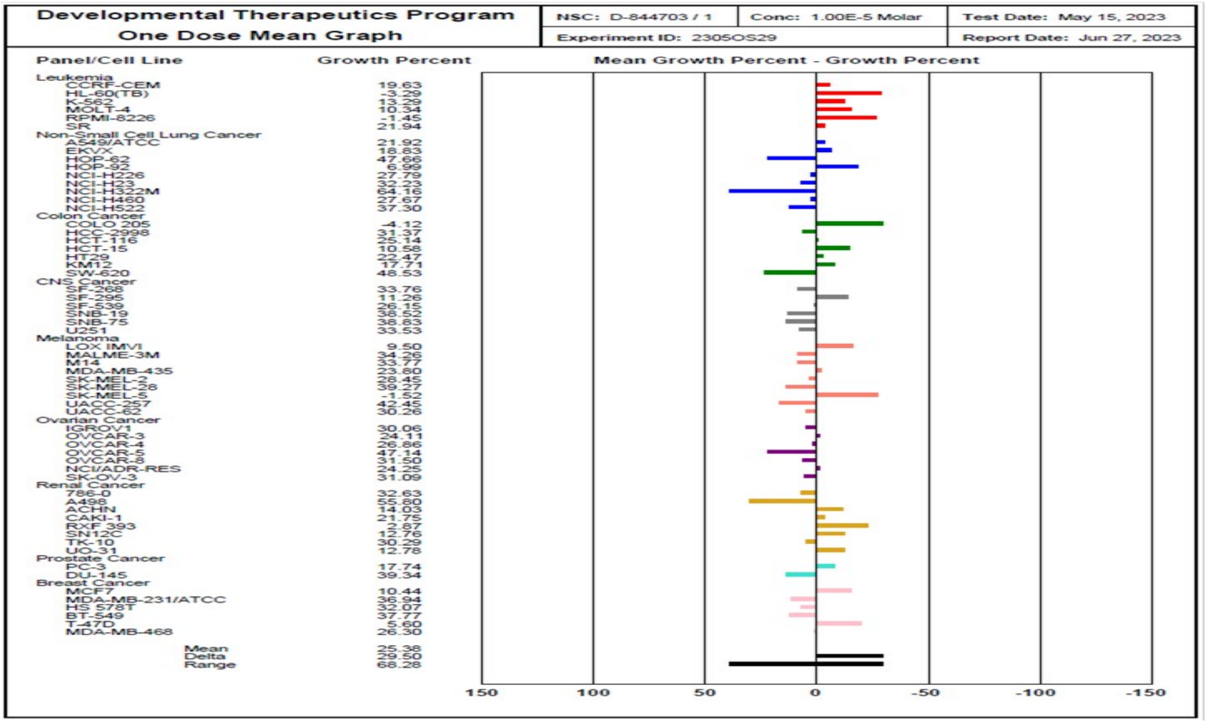

Figure 144. NCI report for growth inhibitory activity of sample **12d** at 10µM concentration

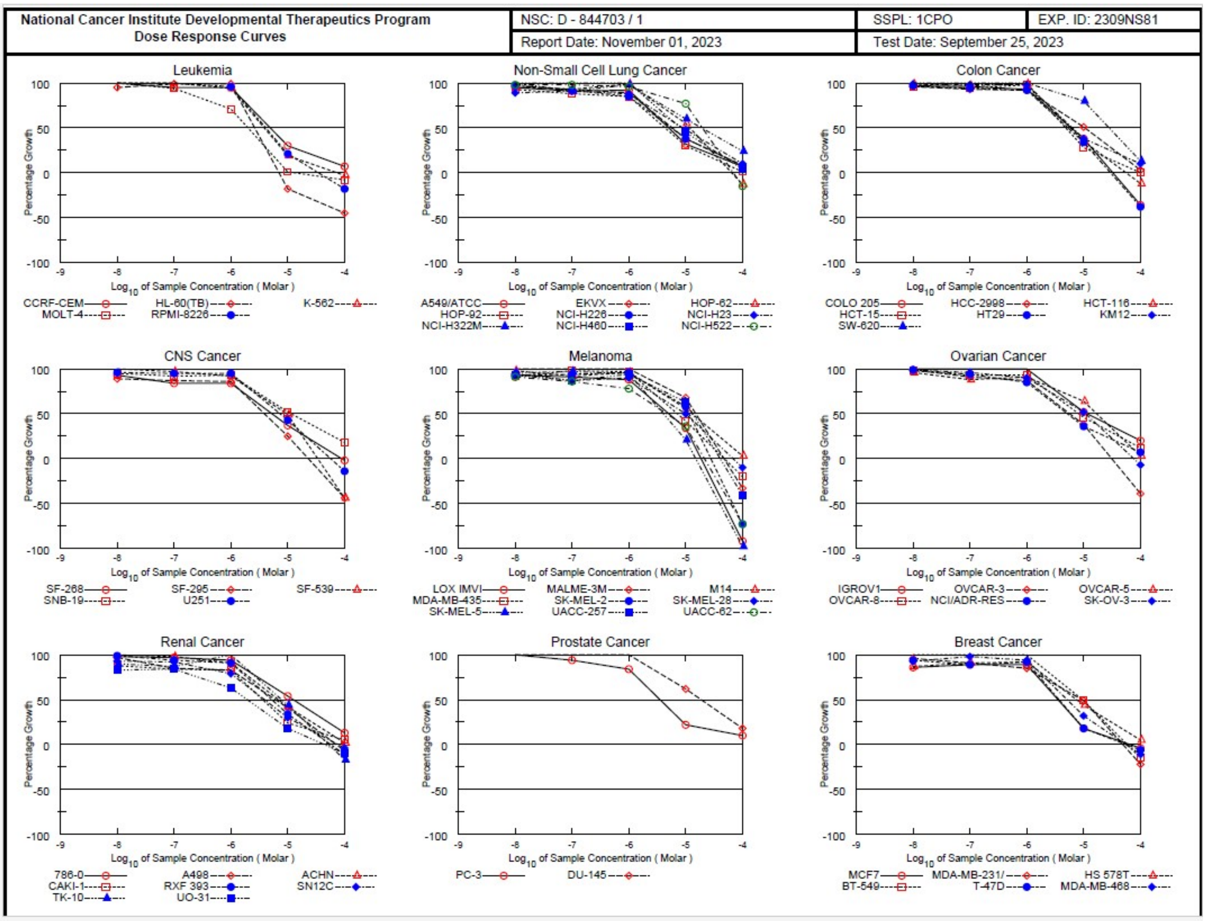

Figure 145. Dose response curves for compound **12d** in individual cell lines

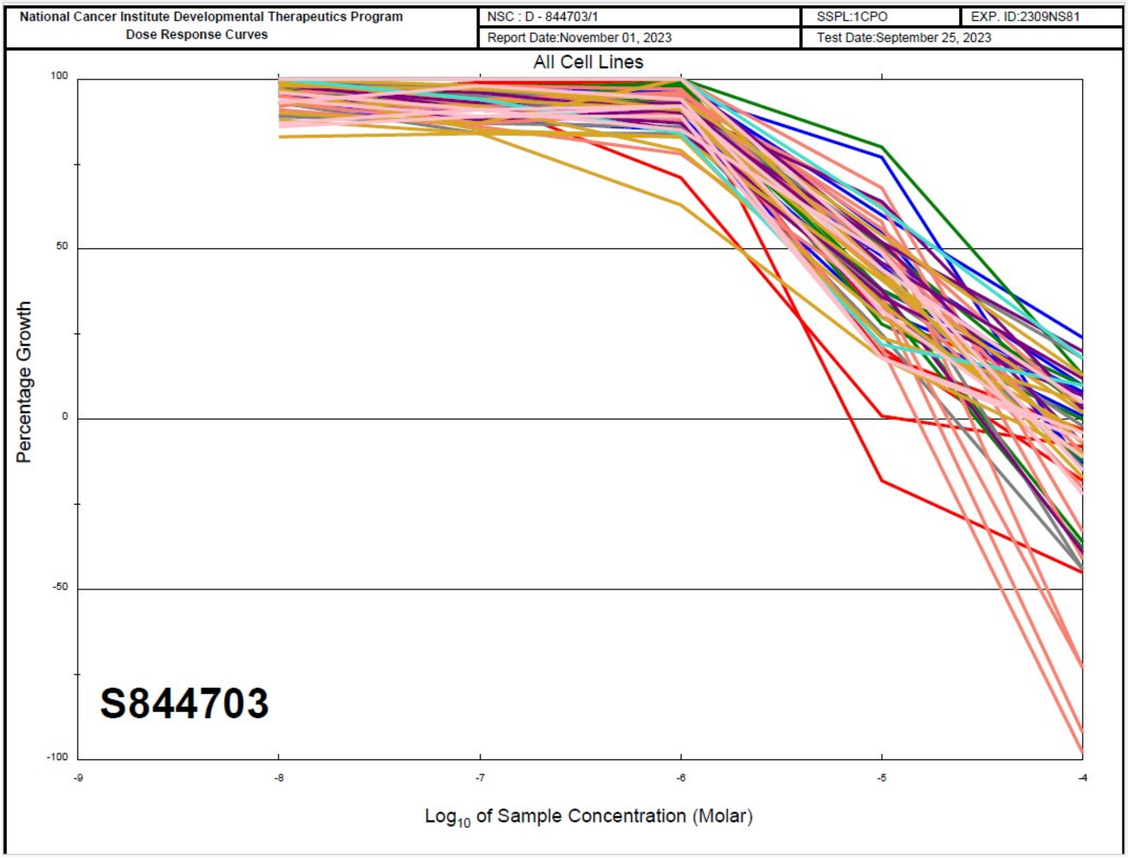

Figure 146. Dose response curves for compound **12d** in all cell lines

National Cancer Institute Developmental Therapeutics Program<br>In-Vitro Testing Results			
NSC : D - 844703 / 1	Experiment ID : 2309NS81	Test Type : 08	Units : Molar
Report Date : November 01, 2023	Test Date : September 25, 2023	QNS :	MC :
COMI : 038	Stain Reagent : SRB Dual-Pass Related	SSPL : 1CPO	

			Log10 Concentration												
	Time		Mean Optical Densities					Percent Growth							
Panel/Cell Line	Zero	Ctrl	-8.0	-7.0	-6.0	-5.0	-4.0	-8.0	-7.0	-6.0	-5.0	-4.0	GI50	TGI	LC50
Leukemia															
CCRF-CEM	0.494	2.711	2.715	2.603	2.601	1.153	0.660	100	95	95	30	7	4.89E-6	> 1.00E-4	> 1.00E-4
HL-60(TB)	0.589	2.595	2.496	2.585	2.578	0.485	0.325	95	99	99	-18	-45	2.63E-6	7.05E-6	> 1.00E-4
K-562	0.236	2.385	2.411	2.443	2.248	0.638	0.230	101	103	94	19	-3	3.82E-6	7.59E-5	> 1.00E-4
MOLT-4	0.508	2.453	2.444	2.344	1.886	0.535	0.466	100	94	71	1	-8	2.00E-6	1.39E-5	> 1.00E-4
RPMI-8226	0.797	2.574	2.648	2.733	2.510	1.162	0.657	104	109	96	21	-18	4.08E-6	3.45E-5	> 1.00E-4
Non-Small Cell Lung Cancer															
A549/ATCC	0.421	2.275	2.187	2.107	2.126	1.017	0.548	95	91	92	32	7	5.03E-6	> 1.00E-4	> 1.00E-4
EKVX	0.945	2.313	2.279	2.215	2.167	1.466	1.057	97	93	89	38	8	5.85E-6	> 1.00E-4	> 1.00E-4
HOP-62	0.538	1.897	1.831	1.808	1.885	1.286	0.468	95	93	99	55	-13	1.19E-5	6.42E-5	> 1.00E-4
HOP-92	0.834	1.663	1.603	1.561	1.541	1.084	0.845	93	88	85	30	1	4.36E-6	> 1.00E-4	> 1.00E-4
NCI-H226	1.201	2.405	2.365	2.304	2.228	1.656	1.303	97	92	85	38	8	5.53E-6	> 1.00E-4	> 1.00E-4
NCI-H23	0.595	2.105	1.943	1.972	1.931	1.322	0.749	89	91	88	48	10	8.98E-6	> 1.00E-4	> 1.00E-4
NCI-H322M	0.916	2.504	2.575	2.368	2.466	1.865	1.291	104	91	98	60	24	1.86E-5	> 1.00E-4	> 1.00E-4
NCI-H460	0.288	2.502	2.509	2.803	2.603	1.293	0.370	100	114	105	45	4	8.36E-6	> 1.00E-4	> 1.00E-4
NCI-H522	2.068	3.366	3.341	3.334	3.317	3.073	1.759	98	98	96	77	-15	1.98E-5	6.89E-5	> 1.00E-4
Colon Cancer															
COLO 205	0.645	2.793	2.739	2.708	2.647	1.458	0.416	97	96	93	38	-36	6.03E-6	3.28E-5	> 1.00E-4
HCC-2998	0.669	2.596	2.521	2.455	2.450	1.647	0.732	96	93	92	51	3	1.04E-5	> 1.00E-4	> 1.00E-4
HCT-116	0.251	2.392	2.368	2.332	2.380	1.004	0.222	99	97	99	35	-12	5.88E-6	5.62E-5	> 1.00E-4
HCT-15	0.409	2.729	2.640	2.597	2.676	1.056	0.416	96	94	98	28	0	4.82E-6	> 1.00E-4	> 1.00E-4
HT29	0.402	2.496	2.434	2.381	2.322	1.107	0.251	97	94	92	34	-38	5.23E-6	2.97E-5	> 1.00E-4
KM12	0.767	3.292	3.268	3.250	3.233	1.714	1.021	99	98	98	38	10	6.20E-6	> 1.00E-4	> 1.00E-4
SW-620	0.336	2.077	2.294	2.092	2.184	1.724	0.557	112	101	106	80	13	2.77E-5	> 1.00E-4	> 1.00E-4
CNS Cancer															
SF-268	0.793	2.323	2.213	2.071	2.082	1.355	0.776	93	84	84	37	-2	5.25E-6	8.81E-5	> 1.00E-4
SF-295	0.734	2.356	2.179	2.150	2.135	1.138	0.413	89	87	86	25	-44	3.90E-6	2.30E-5	> 1.00E-4
SF-539	0.969	2.877	2.889	2.812	2.716	1.912	0.540	101	97	92	49	-44	9.67E-6	3.37E-5	> 1.00E-4
SNB-19	0.609	2.083	2.016	1.959	1.977	1.370	0.869	95	92	93	52	18	1.12E-5	> 1.00E-4	> 1.00E-4
U251	0.390	1.770	1.722	1.703	1.695	0.981	0.334	96	95	95	43	-14	7.27E-6	5.61E-5	> 1.00E-4
Melanoma															
LOX IMVI	0.390	2.451	2.312	2.275	2.211	1.083	0.032	93	91	88	34	-92	5.02E-6	1.85E-5	4.64E-5
MALME-3M	0.698	1.406	1.526	1.594	1.480	1.178	0.466	117	127	111	68	-33	1.50E-5	4.68E-5	> 1.00E-4
M14	0.488	1.921	1.892	1.805	1.859	1.285	0.530	98	92	96	56	3	1.28E-5	> 1.00E-4	> 1.00E-4
MDA-MB-435	0.589	2.263	2.123	2.225	2.216	1.298	0.469	92	98	97	42	-20	7.25E-6	4.72E-5	> 1.00E-4
SK-MEL-2	1.482	2.843	2.746	2.763	2.786	2.272	0.400	93	94	96	58	-73	1.15E-5	2.77E-5	6.67E-5
SK-MEL-28	0.408	1.596	1.532	1.446	1.480	1.003	0.366	95	87	90	50	-10	1.00E-5	6.75E-5	> 1.00E-4
SK-MEL-5	1.218	3.352	3.275	3.286	3.244	1.663	0.021	96	97	95	21	-98	4.04E-6	1.50E-5	3.93E-5
UACC-257	1.356	2.814	2.715	2.618	2.732	2.273	0.803	93	87	94	63	-41	1.33E-5	4.04E-5	> 1.00E-4
UACC-62	0.888	2.918	2.740	2.637	2.473	1.609	0.241	91	86	78	36	-73	4.57E-6	2.13E-5	6.15E-5
Ovarian Cancer															
IGROV1	0.805	2.364	2.579	2.625	2.545	1.613	1.110	114	117	112	52	20	1.14E-5	> 1.00E-4	> 1.00E-4
OVCAR-3	0.583	1.898	1.884	1.777	1.731	1.087	0.358	99	91	87	38	-39	5.77E-6	3.15E-5	> 1.00E-4
OVCAR-5	0.675	1.768	1.723	1.642	1.669	1.375	0.709	96	88	91	64	3	1.70E-5	> 1.00E-4	> 1.00E-4
OVCAR-8	0.556	2.546	2.510	2.409	2.405	1.477	0.794	98	93	93	46	12	8.32E-6	> 1.00E-4	> 1.00E-4
NCI/ADR-RES	0.477	1.717	1.702	1.642	1.531	0.919	0.559	99	94	85	36	7	5.11E-6	> 1.00E-4	> 1.00E-4
SK-OV-3	0.834	1.968	1.960	1.928	1.858	1.419	0.774	99	96	90	52	-7	1.06E-5	7.54E-5	> 1.00E-4
Renal Cancer															
786-0	0.639	2.818	2.783	2.752	2.697	1.810	0.932	98	97	94	54	13	1.24E-5	> 1.00E-4	> 1.00E-4
A498	1.292	2.272	2.245	2.125	2.101	1.693	1.199	97	85	83	41	-7	6.05E-6	7.07E-5	> 1.00E-4
ACHN	0.288	1.502	1.512	1.477	1.391	0.796	0.317	101	98	91	42	2	6.81E-6	> 1.00E-4	> 1.00E-4
CAKI-1	0.603	2.530	2.295	2.219	2.203	1.069	0.698	88	84	83	24	5	3.64E-6	> 1.00E-4	> 1.00E-4
RXF 393	0.815	1.570	1.560	1.523	1.501	1.070	0.725	99	94	91	34	-11	5.20E-6	5.66E-5	> 1.00E-4
SN12C	0.457	2.002	1.896	1.871	1.680	0.915	0.441	93	92	79	30	-4	3.88E-6	7.79E-5	> 1.00E-4
TK-10	1.329	2.137	2.057	2.028	2.240	1.685	1.107	90	87	113	44	-17	8.20E-6	5.31E-5	> 1.00E-4
UO-31	0.668	2.105	1.856	1.873	1.573	0.934	0.599	83	84	63	18	-10	1.96E-6	4.36E-5	> 1.00E-4
Prostate Cancer															
PC-3	0.559	2.292	2.292	2.186	2.018	0.949	0.732	100	94	84	22	10	3.58E-6	> 1.00E-4	> 1.00E-4
DU-145	0.359	1.606	1.632	1.615	1.629	1.127	0.584	102	101	102	62	18	1.85E-5	> 1.00E-4	> 1.00E-4
Breast Cancer															
MCF7	0.625	2.551	2.277	2.336	2.348	0.979	0.602	86	89	89	18	-4	3.59E-6	6.81E-5	> 1.00E-4
MDA-MB-231/ATCC	0.495	1.216	1.190	1.153	1.107	0.847	0.387	96	91	85	49	-22	9.29E-6	4.91E-5	> 1.00E-4
HS 578T	0.868	2.131	1.965	2.022	2.026	1.425	0.934	87	91	92	44	5	7.50E-6	> 1.00E-4	> 1.00E-4
BT-549	1.079	2.355	2.358	2.384	2.378	1.703	0.916	100	102	102	49	-15	9.53E-6	5.80E-5	> 1.00E-4
T-47D	0.750	1.843	1.780	1.725	1.751	0.952	0.705	94	89	92	18	-6	3.70E-6	5.68E-5	> 1.00E-4
MDA-MB-468	0.959	1.988	1.921	1.965	1.923	1.291	0.850	93	98	94	32	-11	5.15E-6	5.48E-5	> 1.00E-4

Figure 147. In-vitro five dose testing results for compound **12d**

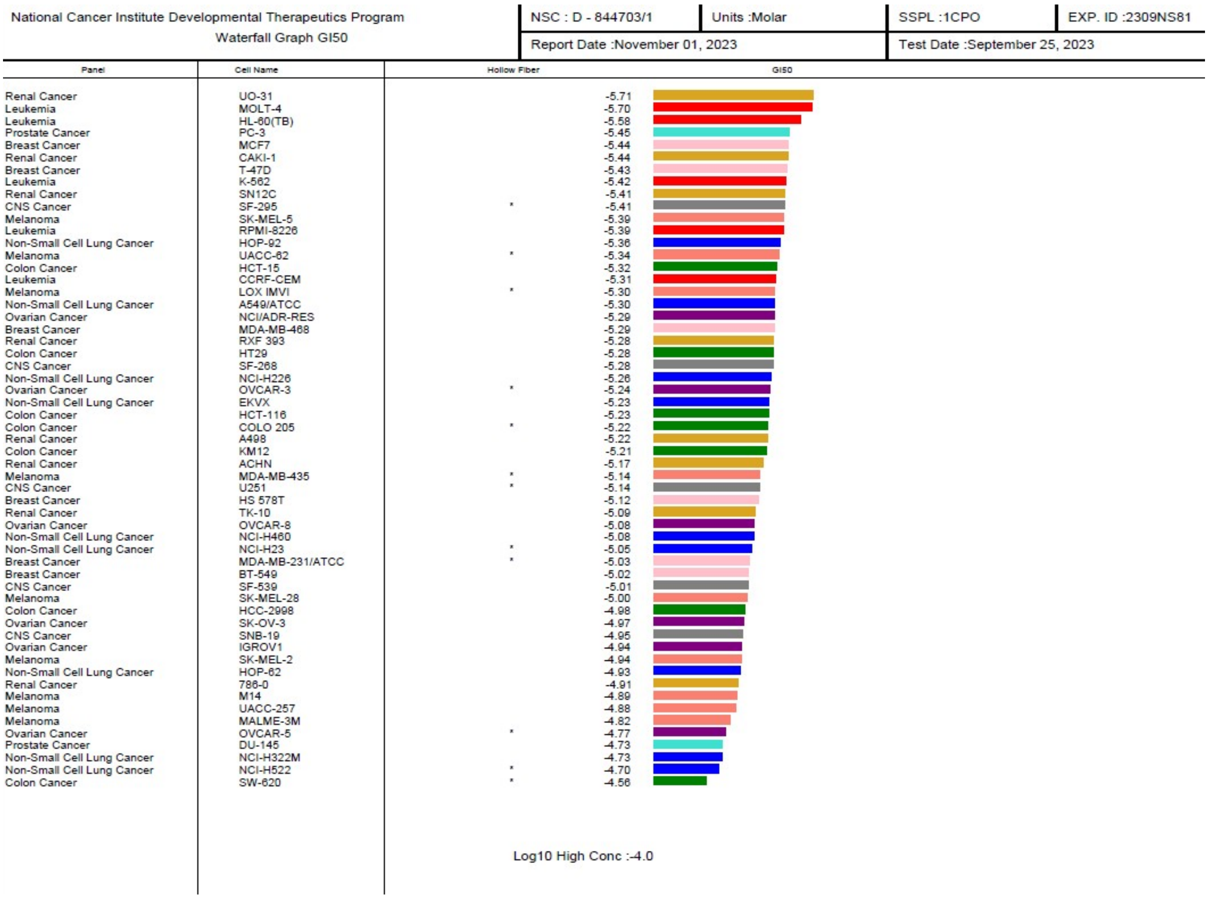

Figure 148. IC_{50} values depicted in the waterfall graph for compound **12d**

In-vitro anti-cancer activity against NCI 60 cell line panel – five dose study and NCI COMPARE. (Compound 12f)

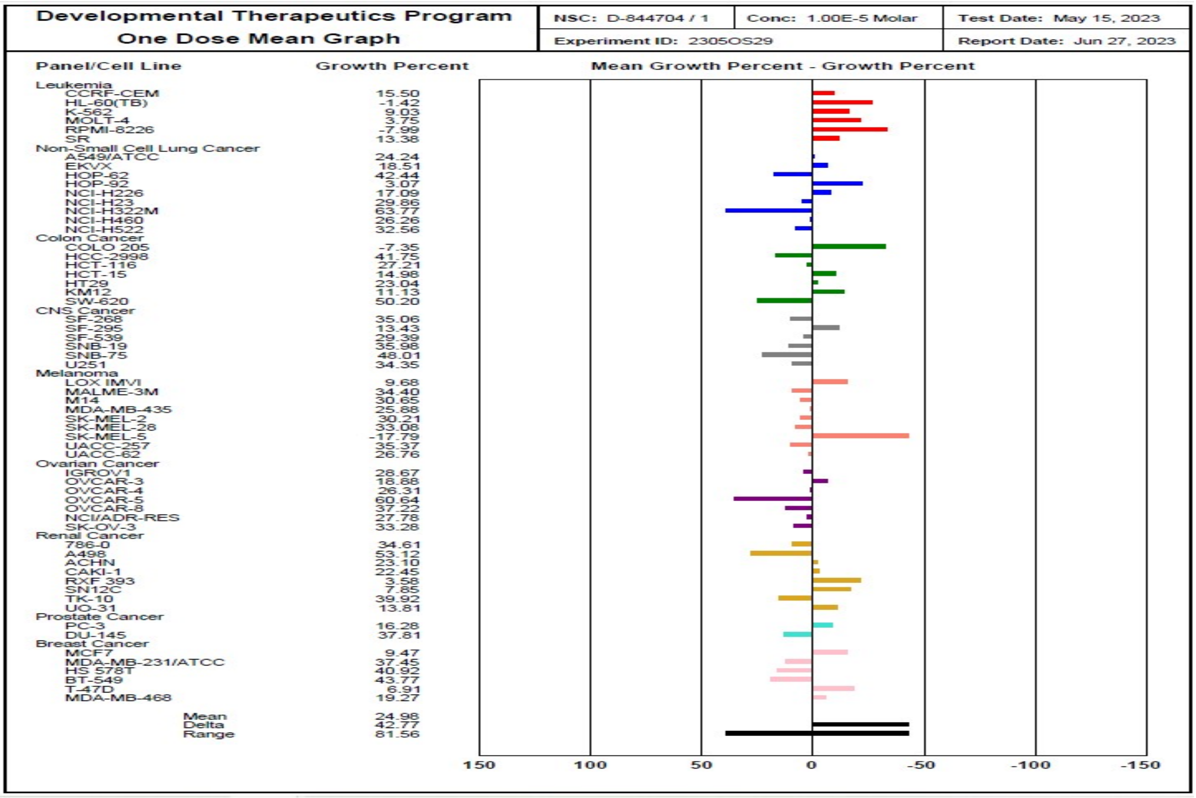

Figure 149. NCI report for growth inhibitory activity of sample **12f** at 10µM concentration

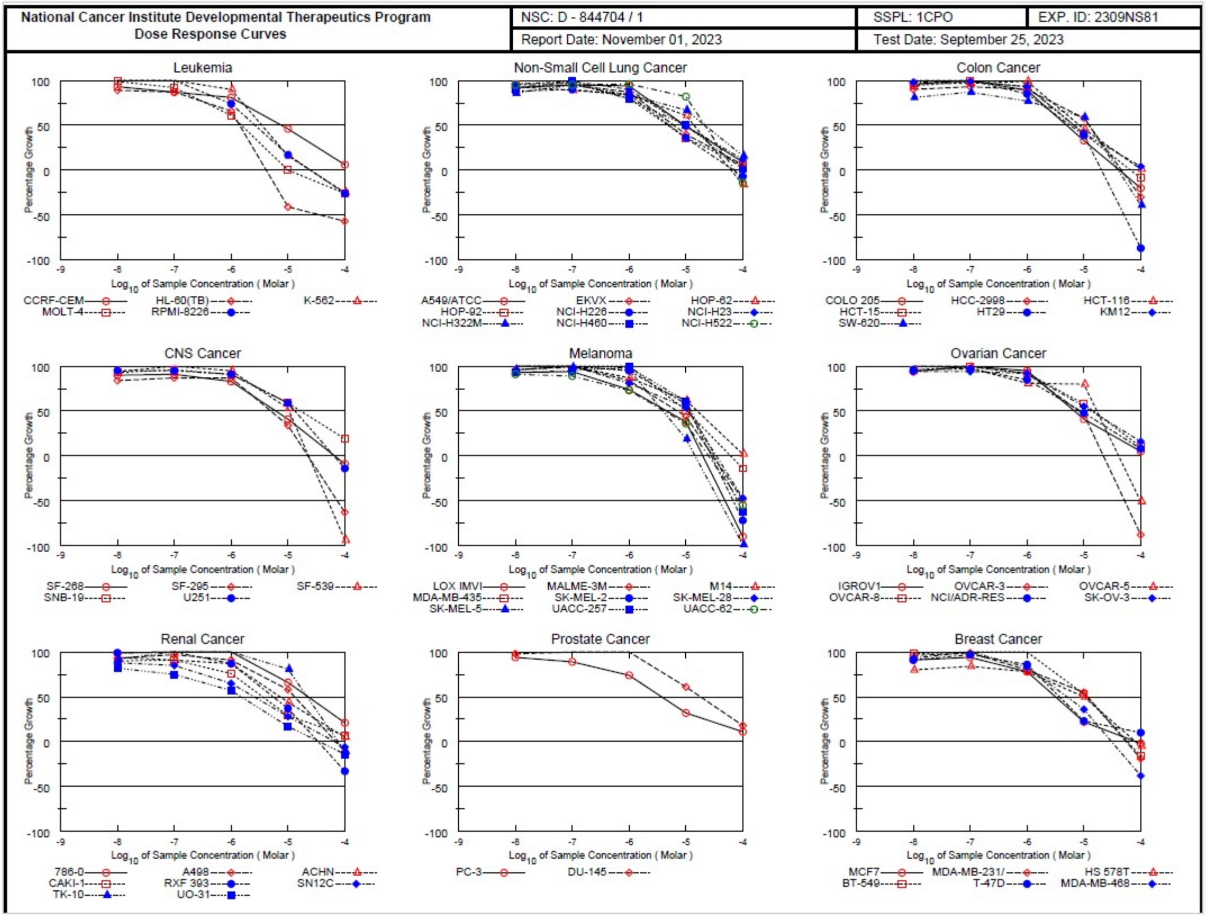

Figure 150. Dose response curves for compound **12f** in individual cell lines

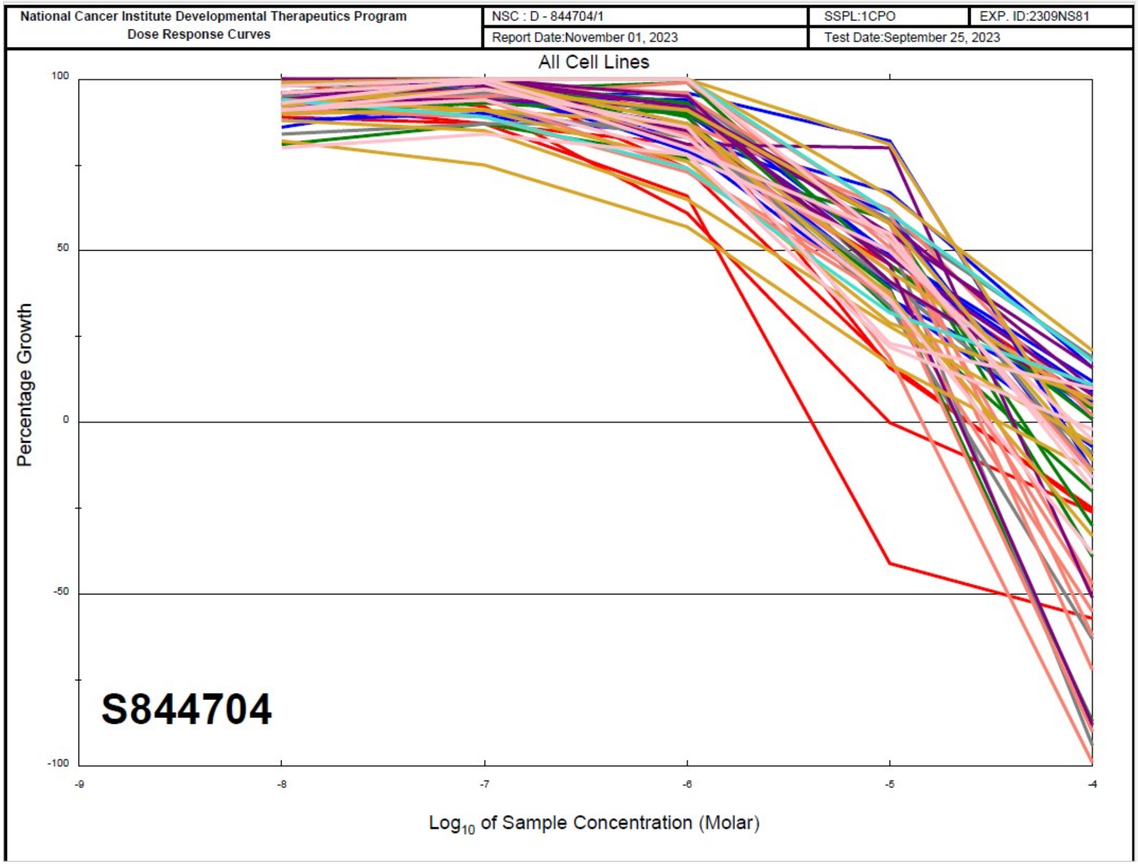

Figure 151. Dose response curves for compound **12f** in all cell lines

National Cancer Institute Developmental Therapeutics Program
In-Vitro Testing Results

NSC : D - 844704 / 1	Experiment ID : 2309NS81	Test Type : 08	Units : Molar
Report Date : November 01, 2023	Test Date : September 25, 2023	QNS :	MC :
COMI : 040	Stain Reagent : SRB Dual-Pass Related	SSPL : 1CPO	

			Log10 Concentration												
	Time		Mean Optical Densities					Percent Growth							
Panel/Cell Line	Zero	Ctrl	-8.0	-7.0	-6.0	-5.0	-4.0	-8.0	-7.0	-6.0	-5.0	-4.0	GI50	TGI	LC50
Leukemia															
CCRF-CEM	0.494	2.711	2.549	2.426	2.296	1.504	0.617	93	87	81	46	6	7.50E-6	> 1.00E-4	> 1.00E-4
HL-60(TB)	0.589	2.595	2.379	2.343	1.904	0.346	0.256	89	87	66	-41	-57	1.40E-6	4.10E-6	3.71E-5
K-562	0.236	2.385	2.423	2.463	2.177	0.574	0.178	102	104	90	16	-25	3.47E-6	2.45E-5	> 1.00E-4
MOLT-4	0.508	2.453	2.435	2.298	1.688	0.514	0.376	99	92	61	0	-26	1.50E-6	1.03E-5	> 1.00E-4
RPMI-8226	0.797	2.574	2.644	2.597	2.115	1.105	0.588	104	101	74	17	-26	2.66E-6	2.50E-5	> 1.00E-4
Non-Small Cell Lung Cancer															
A549/ATCC	0.421	2.275	2.130	2.184	2.156	1.325	0.580	92	95	94	49	9	9.37E-6	> 1.00E-4	> 1.00E-4
EKVX	0.945	2.313	2.207	2.248	2.137	1.496	1.029	92	95	87	40	6	6.20E-6	> 1.00E-4	> 1.00E-4
HOP-62	0.538	1.897	1.805	1.751	1.680	1.365	0.454	93	89	84	61	-16	1.38E-5	6.23E-5	> 1.00E-4
HOP-92	0.834	1.663	1.627	1.639	1.494	1.136	0.877	96	97	80	36	5	4.83E-6	> 1.00E-4	> 1.00E-4
NCI-H226	1.201	2.405	2.263	2.290	2.214	1.631	1.119	88	90	84	36	-7	5.06E-6	6.89E-5	> 1.00E-4
NCI-H23	0.595	2.105	2.051	2.093	1.950	1.342	0.770	96	99	90	49	12	9.71E-6	> 1.00E-4	> 1.00E-4
NCI-H322M	0.916	2.504	2.284	2.429	2.247	1.987	1.163	86	95	84	67	16	2.17E-5	> 1.00E-4	> 1.00E-4
NCI-H460	0.288	2.502	2.293	2.482	2.046	1.399	0.301	91	99	79	50	1	1.01E-5	> 1.00E-4	> 1.00E-4
NCI-H522	2.068	3.366	3.299	3.300	3.310	3.138	1.780	95	95	96	82	-14	2.17E-5	7.17E-5	> 1.00E-4
Colon Cancer															
COLO 205	0.645	2.793	2.716	2.790	2.549	1.348	0.514	96	100	89	33	-20	4.91E-6	4.14E-5	> 1.00E-4
HCC-2998	0.669	2.596	2.412	2.461	2.397	1.793	0.465	90	93	90	58	-30	1.24E-5	4.54E-5	> 1.00E-4
HCT-116	0.251	2.392	2.312	2.318	2.369	1.238	0.268	96	97	99	46	1	8.43E-6	> 1.00E-4	> 1.00E-4
HCT-15	0.409	2.729	2.594	2.708	2.577	1.361	0.374	94	99	93	41	-9	6.74E-6	6.72E-5	> 1.00E-4
HT29	0.402	2.496	2.506	2.492	2.178	1.213	0.053	100	100	85	39	-87	5.69E-6	2.03E-5	5.09E-5
KM12	0.767	3.292	3.245	3.262	3.104	1.800	0.858	98	99	93	41	4	6.66E-6	> 1.00E-4	> 1.00E-4
SW-620	0.336	2.077	1.740	1.851	1.680	1.366	0.204	81	87	77	59	-39	1.24E-5	3.99E-5	> 1.00E-4
CNS Cancer															
SF-268	0.793	2.323	2.172	2.186	2.062	1.425	0.719	90	91	83	41	-9	6.18E-6	6.54E-5	> 1.00E-4
SF-295	0.734	2.356	2.098	2.152	2.145	1.293	0.270	84	87	87	34	-63	5.06E-6	2.25E-5	7.31E-5
SF-539	0.969	2.877	2.762	2.917	2.777	1.979	0.054	94	102	95	53	-94	1.05E-5	2.29E-5	4.99E-5
SNB-19	0.609	2.083	1.975	2.021	1.946	1.486	0.889	93	96	91	59	19	1.72E-5	> 1.00E-4	> 1.00E-4
U251	0.390	1.770	1.701	1.708	1.641	1.203	0.337	95	95	91	59	-14	1.33E-5	6.49E-5	> 1.00E-4
Melanoma															
LOX IMVI	0.390	2.451	2.300	2.321	1.921	1.183	0.038	93	94	74	38	-90	4.76E-6	1.99E-5	4.87E-5
MALME-3M	0.698	1.406	1.376	1.430	1.294	1.007	0.356	96	103	84	44	-49	6.98E-6	2.96E-5	> 1.00E-4
M14	0.488	1.921	1.859	1.932	1.742	1.370	0.510	96	101	87	62	2	1.56E-5	> 1.00E-4	> 1.00E-4
MDA-MB-435	0.589	2.263	2.292	2.397	2.269	1.440	0.505	102	108	100	51	-14	1.03E-5	6.02E-5	> 1.00E-4
SK-MEL-2	1.482	2.843	2.843	2.837	2.773	2.269	0.413	100	100	95	58	-72	1.15E-5	2.79E-5	6.76E-5
SK-MEL-28	0.408	1.596	1.679	1.672	1.372	1.048	0.216	107	106	81	54	-47	1.09E-5	3.42E-5	> 1.00E-4
SK-MEL-5	1.218	3.352	3.266	3.325	3.265	1.624	0.018	96	99	96	19	-99	3.96E-6	1.45E-5	3.86E-5
UACC-257	1.356	2.814	2.713	2.742	2.796	2.253	0.521	93	95	99	61	-62	1.24E-5	3.16E-5	8.05E-5
UACC-62	0.888	2.918	2.738	2.702	2.373	1.623	0.400	91	89	73	36	-55	4.24E-6	2.50E-5	8.82E-5
Ovarian Cancer															
IGROV1	0.805	2.364	2.276	2.371	2.285	1.448	0.880	94	100	95	41	5	6.87E-6	> 1.00E-4	> 1.00E-4
OVCAR-3	0.583	1.898	1.893	1.935	1.791	1.189	0.070	100	103	92	46	-88	8.21E-6	2.21E-5	5.21E-5
OVCAR-5	0.675	1.768	1.822	1.747	1.564	1.553	0.333	105	98	81	80	-51	1.70E-5	4.10E-5	9.87E-5
OVCAR-8	0.556	2.546	2.468	2.517	2.380	1.707	0.752	96	99	92	58	10	1.46E-5	> 1.00E-4	> 1.00E-4
NCI/ADR-RES	0.477	1.717	1.665	1.683	1.528	1.072	0.575	96	97	85	48	8	8.81E-6	> 1.00E-4	> 1.00E-4
SK-OV-3	0.834	1.968	1.896	1.896	1.875	1.462	1.021	94	94	92	55	16	1.38E-5	> 1.00E-4	> 1.00E-4
Renal Cancer															
786-0	0.639	2.818	2.788	2.871	2.819	2.082	1.088	99	102	100	66	21	2.26E-5	> 1.00E-4	> 1.00E-4
A498	1.292	2.272	2.204	2.245	2.183	1.865	1.144	93	97	91	58	-11	1.32E-5	6.86E-5	> 1.00E-4
ACHN	0.288	1.502	1.429	1.390	1.342	0.818	0.349	94	91	87	44	5	7.14E-6	> 1.00E-4	> 1.00E-4
CAKI-1	0.603	2.530	2.329	2.350	2.064	1.170	0.730	90	91	76	29	7	3.60E-6	> 1.00E-4	> 1.00E-4
RXF 393	0.815	1.570	1.564	1.607	1.469	1.097	0.545	99	105	87	37	-33	5.52E-6	3.38E-5	> 1.00E-4
SN12C	0.457	2.002	1.823	1.768	1.465	0.884	0.431	88	85	65	28	-6	2.54E-6	6.75E-5	> 1.00E-4
TK-10	1.329	2.137	2.071	2.194	2.458	1.981	1.177	92	107	140	81	-11	2.15E-5	7.51E-5	> 1.00E-4
UO-31	0.668	2.105	1.844	1.752	1.482	0.916	0.576	82	75	57	17	-14	1.47E-6	3.58E-5	> 1.00E-4
Prostate Cancer															
PC-3	0.559	2.292	2.192	2.110	1.846	1.121	0.754	94	89	74	32	11	3.80E-6	> 1.00E-4	> 1.00E-4
DU-145	0.359	1.606	1.581	1.662	1.643	1.124	0.586	98	105	103	61	18	1.83E-5	> 1.00E-4	> 1.00E-4
Breast Cancer															
MCF7	0.625	2.551	2.376	2.429	2.129	1.051	0.615	91	94	78	22	-2	3.18E-6	8.50E-5	> 1.00E-4
MDA-MB-231/ATCC	0.495	1.216	1.185	1.208	1.075	0.895	0.399	96	99	80	55	-19	1.18E-5	5.49E-5	> 1.00E-4
HS 578T	0.868	2.131	1.876	1.929	1.854	1.497	0.823	80	84	78	50	-5	9.80E-6	8.03E-5	> 1.00E-4
BT-549	1.079	2.355	2.331	2.531	2.353	1.754	0.902	98	114	100	53	-16	1.10E-5	5.79E-5	> 1.00E-4
T-47D	0.750	1.843	1.745	1.810	1.689	0.997	0.863	91	97	86	23	10	3.69E-6	> 1.00E-4	> 1.00E-4
MDA-MB-468	0.959	1.988	1.917	1.991	1.816	1.326	0.593	93	100	83	36	-38	4.99E-6	3.04E-5	> 1.00E-4

Figure 152. In-vitro five dose testing results for compound **12f**

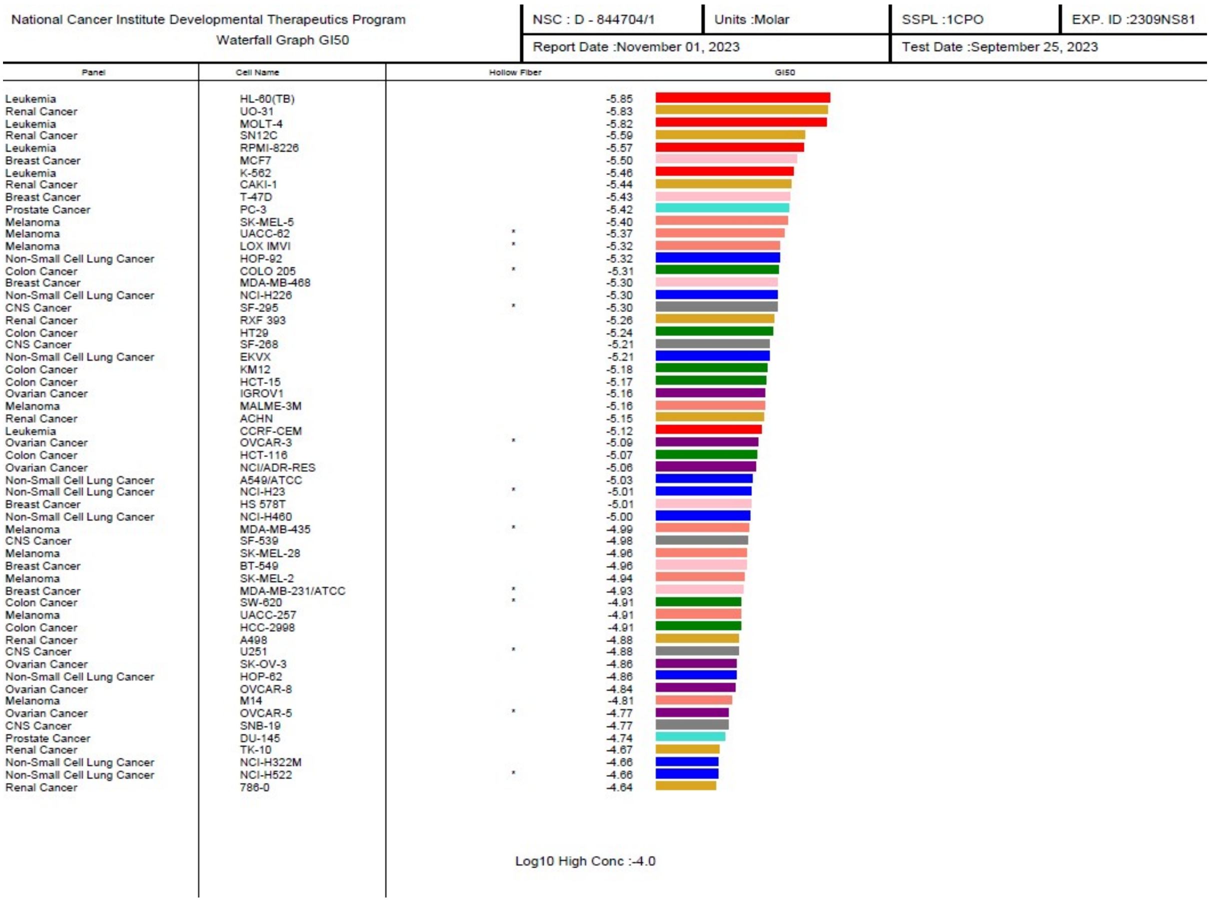

Figure 153. IC$_{50}$ values depicted in the waterfall graph for compound **12f**

In-vitro anti-cancer activity against NCI 60 cell line panel – five dose study and NCI COMPARE. (Compound 12j)

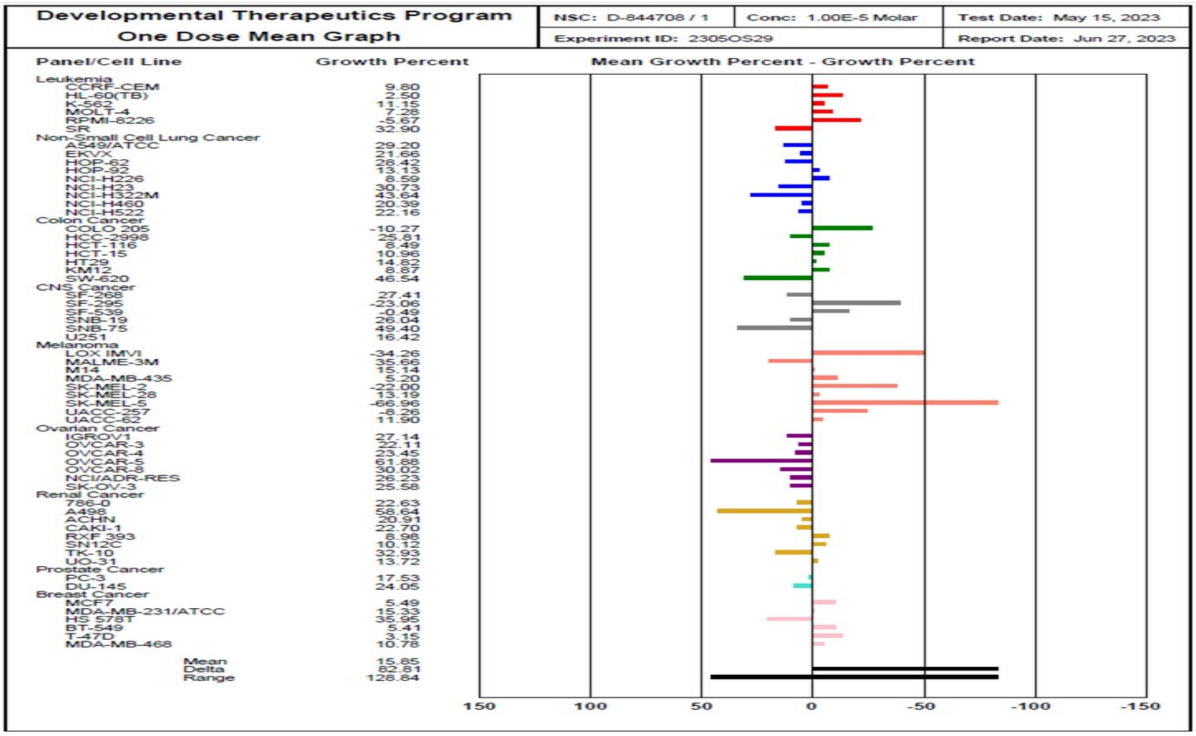

Figure 154. NCI report for growth inhibitory activity of sample **12j** at 10µM concentration

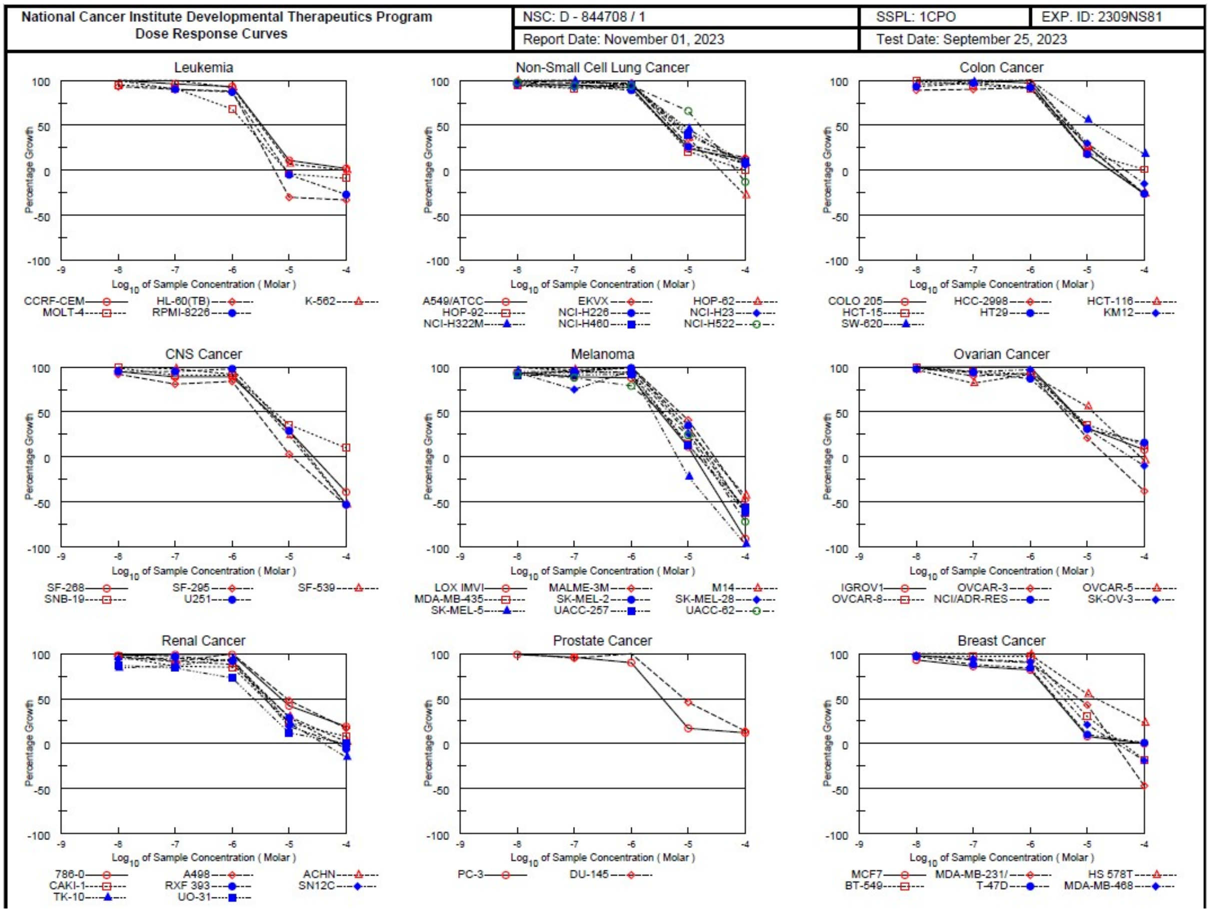

Figure 155. Dose response curves for compound **12j** in individual cell lines

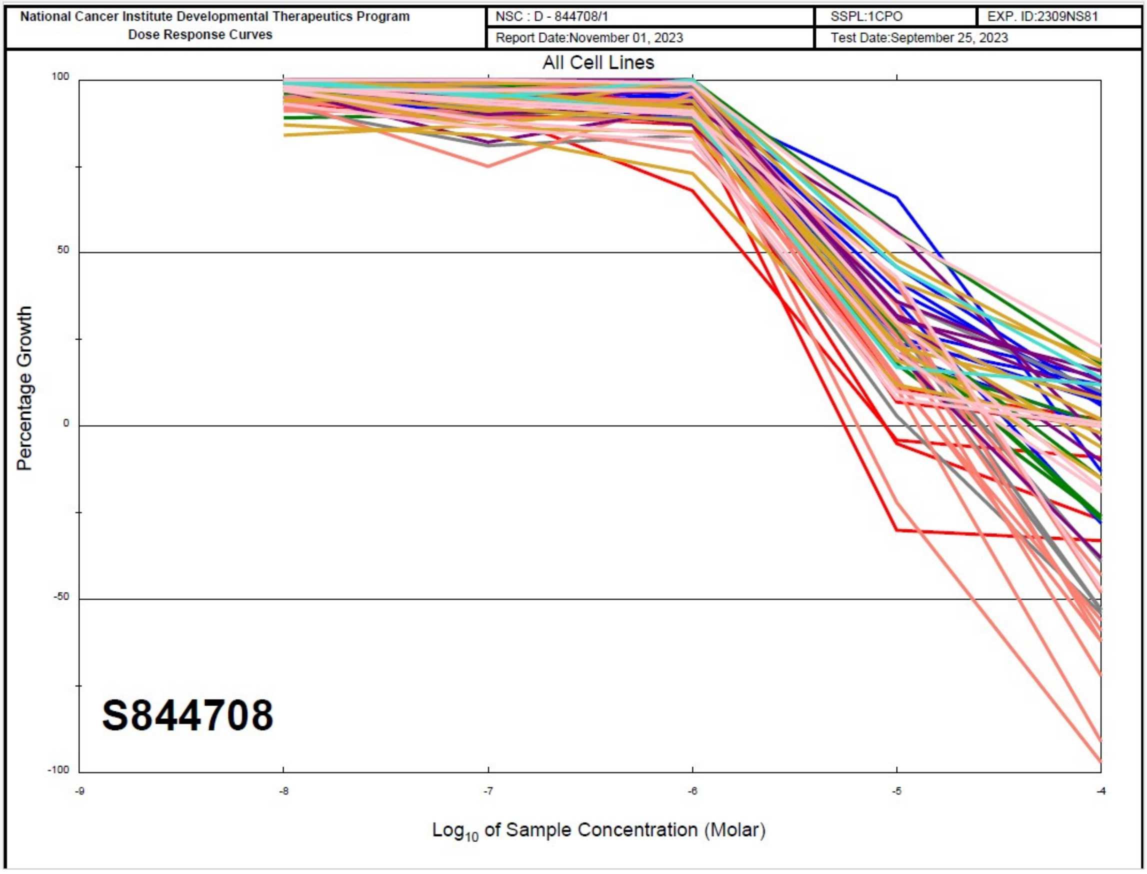

Figure 156. Dose response curves for compound **12j** in all cell lines

National Cancer Institute Developmental Therapeutics Program
In-Vitro Testing Results

NSC : D - 844708 / 1	Experiment ID : 2309NS81	Test Type : 08	Units : Molar
Report Date : November 01, 2023	Test Date : September 25, 2023	QNS :	MC :
COMI : 044	Stain Reagent : SRB Dual-Pass Related	SSPL : 1CPO	

			Log10 Concentration												
	Time		Mean Optical Densities					Percent Growth							
Panel/Cell Line	Zero	Ctrl	-8.0	-7.0	-6.0	-5.0	-4.0	-8.0	-7.0	-6.0	-5.0	-4.0	GI50	TGI	LC50
**Leukemia**															
CCRF-CEM	0.494	2.709	2.725	2.610	2.547	0.730	0.531	101	96	93	11	2	3.31E-6	> 1.00E-4	> 1.00E-4
HL-60(TB)	0.589	2.620	2.471	2.415	2.376	0.411	0.394	93	90	88	-30	-33	2.10E-6	5.55E-6	> 1.00E-4
K-562	0.236	2.277	2.340	2.308	2.117	0.373	0.238	103	101	92	7	0	3.11E-6	> 1.00E-4	> 1.00E-4
MOLT-4	0.508	2.611	2.508	2.413	1.946	0.490	0.461	95	91	68	-4	-9	1.80E-6	8.93E-6	> 1.00E-4
RPMI-8226	0.797	2.610	2.633	2.428	2.381	0.755	0.584	101	90	87	-5	-27	2.53E-6	8.76E-6	> 1.00E-4
**Non-Small Cell Lung Cancer**															
A549/ATCC	0.421	2.352	2.252	2.265	2.205	0.881	0.645	95	95	92	24	12	4.15E-6	> 1.00E-4	> 1.00E-4
EKVX	0.945	2.328	2.319	2.293	2.282	1.335	1.142	99	97	97	28	14	4.80E-6	> 1.00E-4	> 1.00E-4
HOP-62	0.538	1.908	1.889	1.951	1.837	1.037	0.390	99	103	95	36	-28	5.85E-6	3.71E-5	> 1.00E-4
HOP-92	0.834	1.612	1.565	1.539	1.551	0.998	0.827	94	91	92	21	0	3.91E-6	9.10E-5	> 1.00E-4
NCI-H226	1.201	2.411	2.371	2.340	2.273	1.519	1.290	97	94	89	26	7	4.16E-6	> 1.00E-4	> 1.00E-4
NCI-H23	0.595	2.080	2.012	1.964	2.020	1.226	0.686	95	92	96	42	6	7.23E-6	> 1.00E-4	> 1.00E-4
NCI-H322M	0.916	2.506	2.505	2.491	2.410	1.652	1.037	100	99	94	46	8	8.35E-6	> 1.00E-4	> 1.00E-4
NCI-H460	0.288	2.644	2.708	2.782	2.522	1.214	0.493	103	106	95	39	9	6.41E-6	> 1.00E-4	> 1.00E-4
NCI-H522	2.068	3.388	3.367	3.315	3.304	2.933	1.798	98	94	94	66	-13	1.58E-5	6.82E-5	> 1.00E-4
**Colon Cancer**															
COLO 205	0.645	2.754	2.792	2.767	2.684	1.029	0.480	102	101	97	18	-26	3.93E-6	2.60E-5	> 1.00E-4
HCC-2998	0.669	2.643	2.427	2.452	2.490	1.196	0.488	89	90	92	27	-27	4.41E-6	3.13E-5	> 1.00E-4
HCT-116	0.251	2.422	2.343	2.367	2.466	0.846	0.185	96	97	102	27	-26	4.98E-6	3.22E-5	> 1.00E-4
HCT-15	0.409	2.779	2.748	2.665	2.562	0.857	0.445	99	95	91	19	1	3.70E-6	> 1.00E-4	> 1.00E-4
HT29	0.402	2.541	2.381	2.467	2.365	0.781	0.296	93	97	92	18	-26	3.66E-6	2.51E-5	> 1.00E-4
KM12	0.767	3.254	3.306	3.249	3.273	1.525	0.650	102	100	101	30	-15	5.27E-6	4.64E-5	> 1.00E-4
SW-620	0.336	2.042	2.136	2.010	2.189	1.291	0.637	106	98	109	56	18	1.43E-5	> 1.00E-4	> 1.00E-4
**CNS Cancer**															
SF-268	0.793	2.277	2.205	2.110	2.109	1.230	0.481	95	89	89	29	-39	4.49E-6	2.68E-5	> 1.00E-4
SF-295	0.734	2.481	2.344	2.147	2.204	0.792	0.339	92	81	84	3	-54	2.65E-6	1.14E-5	8.57E-5
SF-539	0.969	2.670	2.745	2.631	2.533	1.383	0.459	104	98	92	24	-53	4.17E-6	2.07E-5	9.23E-5
SNB-19	0.609	2.164	2.143	2.023	2.024	1.169	0.758	99	91	91	36	10	5.57E-6	> 1.00E-4	> 1.00E-4
U251	0.390	1.765	1.702	1.697	1.733	0.789	0.183	95	95	98	29	-53	4.95E-6	2.25E-5	9.14E-5
**Melanoma**															
LOX IMVI	0.390	2.408	2.292	2.190	2.161	0.611	0.036	94	89	88	11	-91	3.10E-6	1.28E-5	3.97E-5
MALME-3M	0.698	1.425	1.374	1.392	1.462	0.993	0.361	93	95	105	41	-48	7.13E-6	2.86E-5	> 1.00E-4
M14	0.488	1.935	1.930	1.891	1.972	0.902	0.276	100	97	103	29	-43	5.14E-6	2.49E-5	> 1.00E-4
MDA-MB-435	0.589	2.346	2.230	2.243	2.235	0.835	0.225	93	94	94	14	-62	3.53E-6	1.53E-5	6.99E-5
SK-MEL-2	1.482	2.874	2.873	2.809	2.858	1.969	0.571	100	95	99	35	-62	5.81E-6	2.30E-5	7.60E-5
SK-MEL-28	0.408	1.441	1.377	1.178	1.397	0.675	0.169	94	75	96	26	-59	4.51E-6	2.02E-5	7.91E-5
SK-MEL-5	1.218	3.323	3.222	3.217	3.193	0.951	0.036	95	95	94	-22	-97	2.39E-6	6.46E-6	2.36E-5
UACC-257	1.356	2.780	2.646	2.652	2.664	1.548	0.599	91	91	92	13	-56	3.42E-6	1.56E-5	8.24E-5
UACC-62	0.888	2.966	2.792	2.720	2.524	1.390	0.249	92	88	79	24	-72	3.36E-6	1.78E-5	5.91E-5
**Ovarian Cancer**															
IGROV1	0.805	2.519	2.556	2.564	2.604	1.351	0.949	102	103	105	32	8	5.64E-6	> 1.00E-4	> 1.00E-4
OVCAR-3	0.583	1.890	1.915	1.754	1.795	0.859	0.362	102	90	93	21	-38	3.95E-6	2.28E-5	> 1.00E-4
OVCAR-5	0.675	1.571	1.541	1.407	1.495	1.173	0.650	97	82	92	56	-4	1.24E-5	8.66E-5	> 1.00E-4
OVCAR-8	0.556	2.563	2.542	2.455	2.408	1.277	0.809	99	95	92	36	13	5.62E-6	> 1.00E-4	> 1.00E-4
NCI/ADR-RES	0.477	1.706	1.685	1.629	1.553	0.852	0.673	98	94	87	31	16	4.55E-6	> 1.00E-4	> 1.00E-4
SK-OV-3	0.834	1.938	1.935	1.881	1.900	1.179	0.750	100	95	97	31	-10	5.16E-6	5.71E-5	> 1.00E-4
**Renal Cancer**															
786-0	0.639	2.852	2.798	2.823	2.828	1.573	1.057	98	99	99	42	19	7.29E-6	> 1.00E-4	> 1.00E-4
A498	1.292	2.242	2.211	2.157	2.316	1.747	1.456	97	91	108	48	17	9.21E-6	> 1.00E-4	> 1.00E-4
ACHN	0.288	1.374	1.408	1.290	1.246	0.618	0.309	103	92	88	30	2	4.58E-6	> 1.00E-4	> 1.00E-4
CAKI-1	0.603	2.459	2.399	2.206	2.175	1.025	0.750	97	86	85	23	8	3.63E-6	> 1.00E-4	> 1.00E-4
RXF 393	0.815	1.577	1.603	1.554	1.514	1.033	0.765	103	97	92	29	-6	4.59E-6	6.64E-5	> 1.00E-4
SN12C	0.457	2.074	1.984	1.990	1.941	0.771	0.447	94	95	92	19	-2	3.78E-6	7.84E-5	> 1.00E-4
TK-10	1.329	2.169	2.039	2.056	2.117	1.520	1.128	84	87	94	23	-15	4.13E-6	3.98E-5	> 1.00E-4
UO-31	0.668	2.149	1.960	1.910	1.747	0.850	0.671	87	84	73	12	0	2.38E-6	> 1.00E-4	> 1.00E-4
**Prostate Cancer**															
PC-3	0.559	2.258	2.243	2.183	2.080	0.847	0.762	99	96	90	17	12	3.50E-6	> 1.00E-4	> 1.00E-4
DU-145	0.359	1.639	1.689	1.581	1.664	0.946	0.543	104	95	102	46	14	8.43E-6	> 1.00E-4	> 1.00E-4
**Breast Cancer**															
MCF7	0.625	2.614	2.474	2.339	2.258	0.783	0.619	93	86	82	8	0	2.71E-6	7.80E-5	> 1.00E-4
MDA-MB-231/ATCC	0.495	1.176	1.162	1.127	1.106	0.790	0.263	98	93	90	43	-47	7.15E-6	3.01E-5	> 1.00E-4
HS 578T	0.868	2.112	2.140	2.180	2.095	1.551	1.148	102	105	99	55	23	1.42E-5	> 1.00E-4	> 1.00E-4
BT-549	1.079	2.435	2.389	2.392	2.401	1.481	0.881	97	97	97	30	-18	5.01E-6	4.14E-5	> 1.00E-4
T-47D	0.750	1.964	1.930	1.820	1.767	0.867	0.759	97	88	84	10	1	2.85E-6	> 1.00E-4	> 1.00E-4
MDA-MB-468	0.959	2.002	1.977	1.942	1.912	1.176	0.774	98	94	91	21	-19	3.86E-6	3.30E-5	> 1.00E-4

Figure 157. In-vitro five dose testing results for compound **12j**

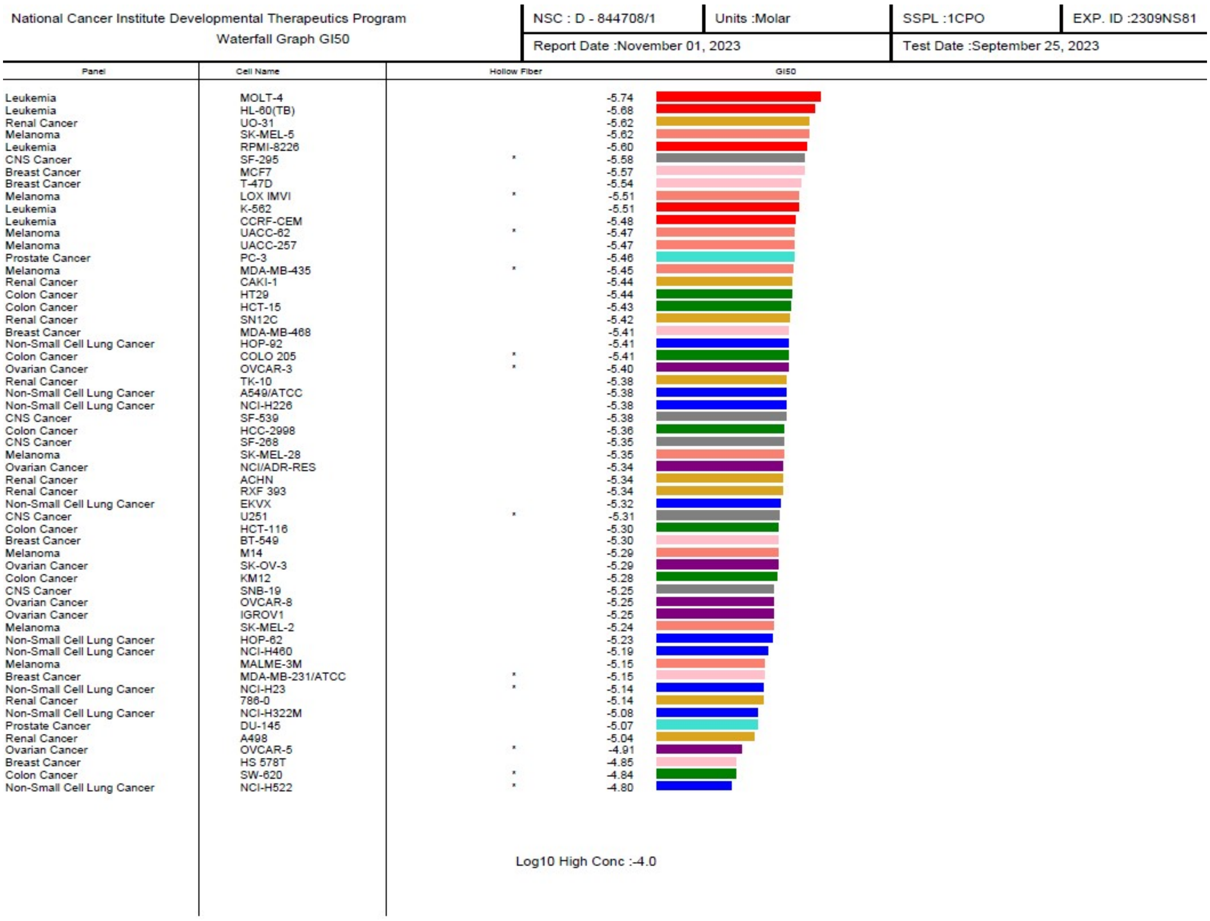

Figure 158. IC_{50} values depicted in the waterfall graph for compound **12j**

In-vitro anti-cancer activity against NCI 60 cell line panel – single dose and five dose study and NCI COMPARE. (Compound 11)

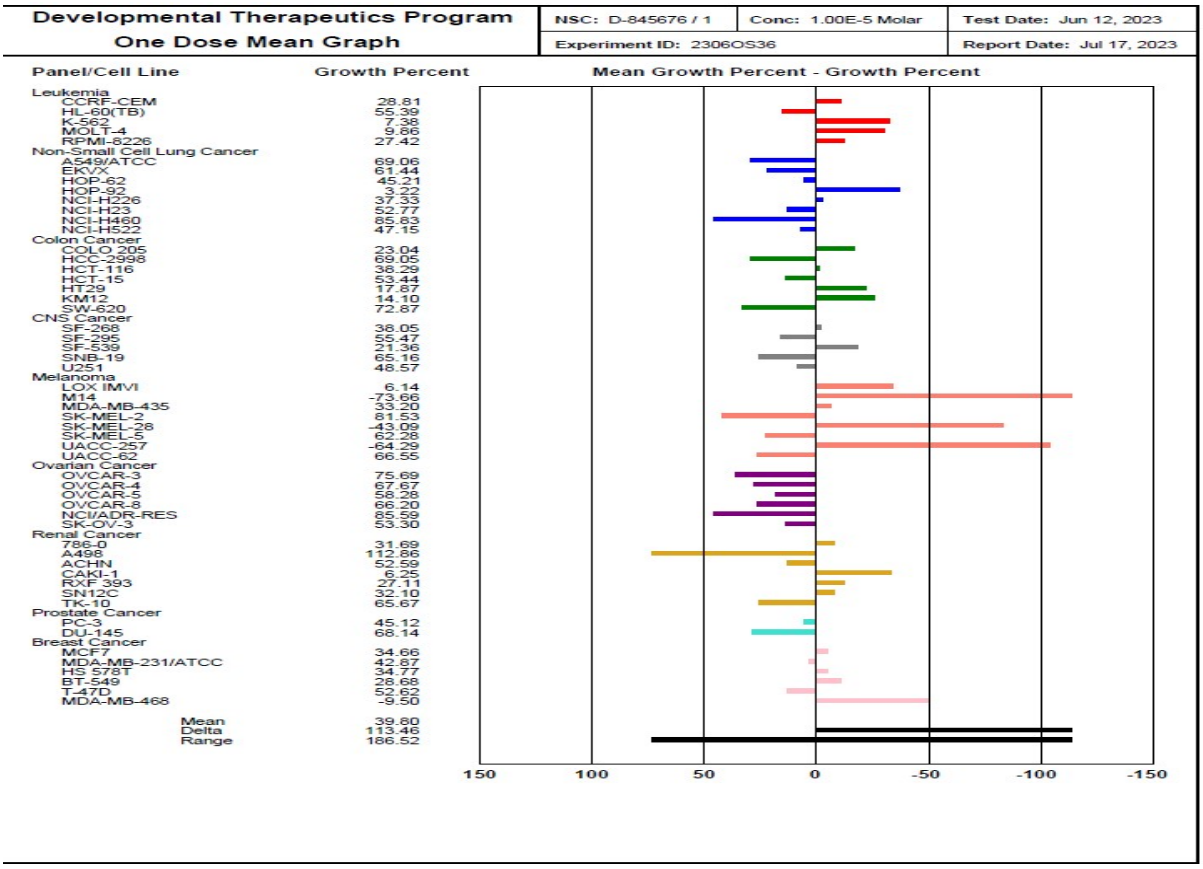

Figure 159. NCI report for growth inhibitory activity of sample **11** at 10µM concentration

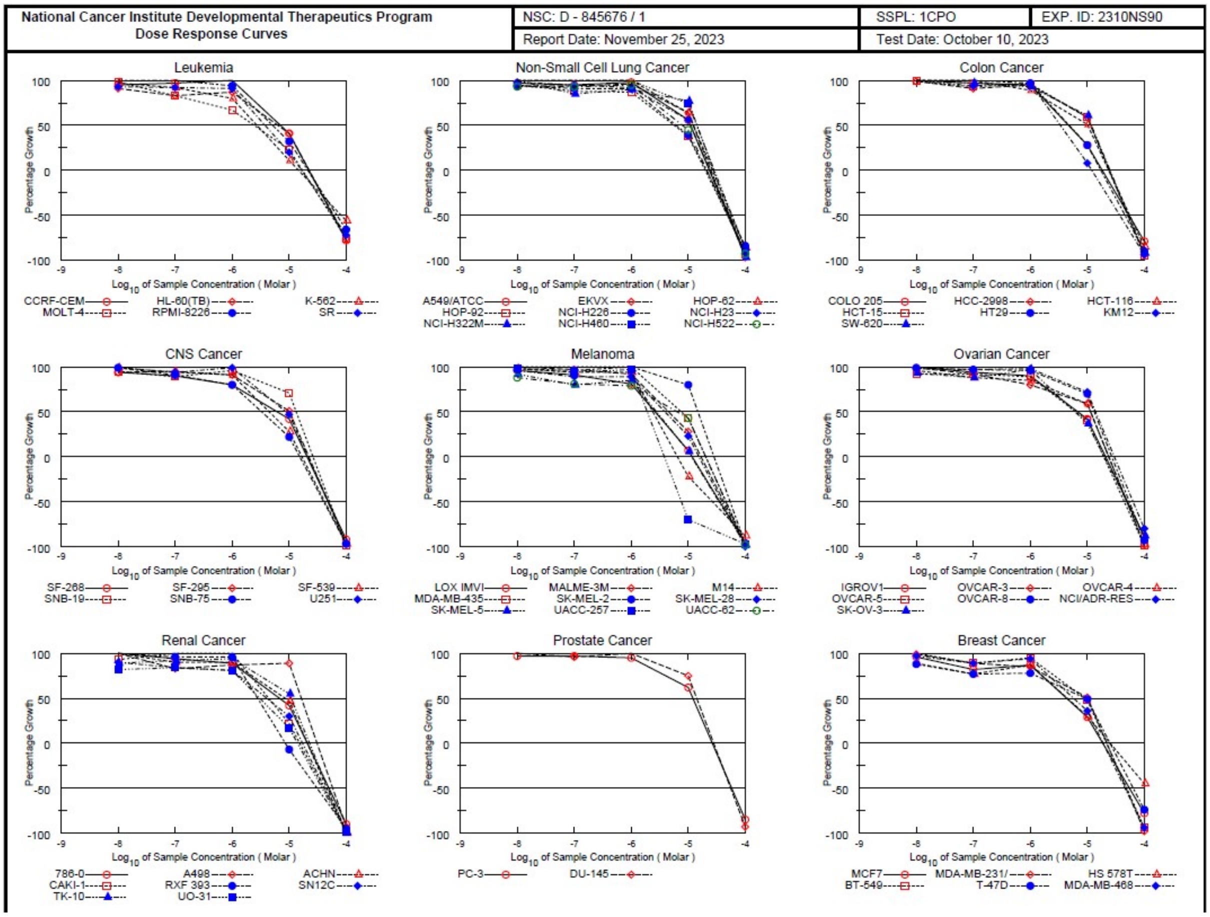

Figure 160. Dose response curves for compound **11** in individual cell lines

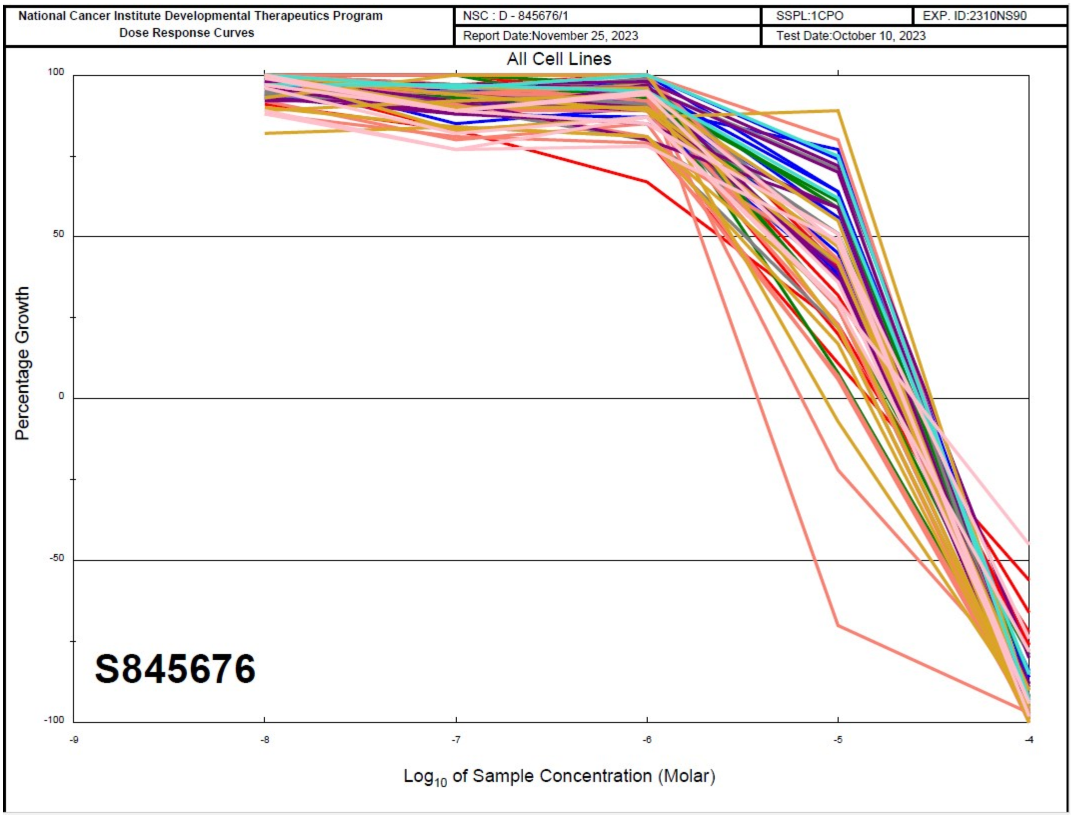

Figure 161. Dose response curves for compound **11** in all cell lines

National Cancer Institute Developmental Therapeutics Program
In-Vitro Testing Results

NSC : D - 845676 / 1	Experiment ID : 2310NS90	Test Type : 08	Units : Molar
Report Date : November 25, 2023	Test Date : October 10, 2023	QNS :	MC :
COMI : AND-052	Stain Reagent : SRB Dual-Pass Related	SSPL : 1CPO	

			Log10 Concentration												
	Time		Mean Optical Densities					Percent Growth							
Panel/Cell Line	Zero	Ctrl	-8.0	-7.0	-6.0	-5.0	-4.0	-8.0	-7.0	-6.0	-5.0	-4.0	GI50	TGI	LC50
Leukemia															
CCRF-CEM	0.316	1.413	1.363	1.385	1.415	0.770	0.070	95	97	100	41	-78	7.14E-6	2.22E-5	5.83E-5
HL-60(TB)	0.474	2.095	1.952	1.826	1.879	1.133	0.114	91	83	87	41	-76	6.25E-6	2.23E-5	5.98E-5
K-562	0.135	1.086	1.062	1.013	0.900	0.239	0.059	97	92	80	11	-56	2.74E-6	1.45E-5	8.06E-5
MOLT-4	0.532	2.420	2.389	2.108	1.799	0.974	0.127	98	83	67	23	-76	2.47E-6	1.72E-5	5.46E-5
RPMI-8226	0.801	2.417	2.515	2.429	2.312	1.310	0.271	106	101	94	32	-66	5.03E-6	2.10E-5	6.83E-5
SR	0.510	2.230	2.103	2.085	2.079	0.855	0.144	93	92	91	20	-72	3.80E-6	1.65E-5	5.79E-5
Non-Small Cell Lung Cancer															
A549/ATCC	0.316	1.600	1.563	1.541	1.572	1.035	0.015	97	95	98	56	-95	1.10E-5	2.34E-5	5.01E-5
EKVX	0.746	2.027	1.950	1.952	1.980	1.563	0.019	94	94	96	64	-97	1.22E-5	2.49E-5	5.08E-5
HOP-62	0.472	1.410	1.507	1.477	1.457	1.076	0.065	110	107	105	64	-86	1.25E-5	2.68E-5	5.75E-5
HOP-92	1.030	1.793	1.758	1.702	1.697	1.318	0.108	95	88	87	38	-90	5.65E-6	1.98E-5	4.89E-5
NCI-H226	1.191	2.425	2.351	2.317	2.314	1.670	0.192	94	91	91	39	-84	6.10E-6	2.07E-5	5.29E-5
NCI-H23	0.671	2.122	2.094	2.048	2.006	1.482	0.051	98	95	92	56	-92	1.10E-5	2.38E-5	5.17E-5
NCI-H322M	0.838	2.251	2.255	2.042	2.115	1.924	0.026	100	85	90	77	-97	1.43E-5	2.77E-5	5.37E-5
NCI-H460	0.323	1.880	2.089	1.943	1.956	1.474	0.040	113	104	105	74	-88	1.41E-5	2.86E-5	5.84E-5
NCI-H522	0.964	2.471	2.372	2.364	2.390	1.648	0.071	93	93	95	45	-93	8.05E-6	2.13E-5	4.91E-5
Colon Cancer															
COLO 205	0.530	1.773	1.829	1.738	1.715	0.880	0.111	105	97	95	28	-79	4.72E-6	1.83E-5	5.36E-5
HCC-2998	1.164	3.148	3.123	2.966	3.055	2.329	0.070	99	91	95	59	-94	1.14E-5	2.42E-5	5.15E-5
HCT-116	0.194	2.196	2.232	2.205	1.981	1.216	0.030	102	100	89	51	-85	1.02E-5	2.38E-5	5.56E-5
HCT-15	0.361	2.341	2.313	2.210	2.261	1.525	0.020	99	93	96	59	-95	1.14E-5	2.42E-5	5.12E-5
HT29	0.203	1.072	1.070	1.154	1.042	0.446	0.020	100	109	97	28	-90	4.77E-6	1.72E-5	4.57E-5
KM12	0.662	1.985	2.373	1.901	1.950	0.768	0.042	129	94	97	8	-94	3.39E-6	1.20E-5	3.72E-5
SW-620	0.309	1.387	1.405	1.352	1.316	0.962	0.025	102	97	93	61	-92	1.17E-5	2.49E-5	5.30E-5
CNS Cancer															
SF-268	0.932	2.356	2.266	2.209	2.070	1.532	0.075	94	90	80	42	-92	6.18E-6	2.06E-5	4.86E-5
SF-295	1.028	2.880	2.774	2.794	2.712	1.975	0.034	94	95	91	51	-97	1.02E-5	2.22E-5	4.83E-5
SF-539	0.944	2.347	2.329	2.262	2.217	1.335	0.025	99	94	91	28	-97	4.45E-6	1.67E-5	4.19E-5
SNB-19	0.787	2.067	2.003	1.924	2.019	1.695	0.004	95	89	96	71	-99	1.33E-5	2.61E-5	5.12E-5
SNB-75	1.063	1.693	1.687	1.637	1.564	1.199	0.034	99	91	80	22	-97	3.23E-6	1.52E-5	4.02E-5
U251	0.232	1.171	1.163	1.118	1.158	0.677	0.010	99	94	99	47	-96	8.88E-6	2.14E-5	4.78E-5
Melanoma															
LOX IMVI	0.425	2.123	2.060	1.966	1.795	0.539	0.017	96	91	81	7	-96	2.60E-6	1.16E-5	3.56E-5
MALME-3M	0.650	1.432	1.532	1.407	1.370	0.872	0.022	113	97	92	28	-97	4.58E-6	1.69E-5	4.23E-5
M14	0.408	1.522	1.497	1.454	1.460	0.319	0.051	98	94	94	-22	-88	2.41E-6	6.49E-6	2.69E-5
MDA-MB-435	0.636	1.914	2.277	2.043	1.977	1.185	0.020	128	110	105	43	-97	7.69E-6	2.03E-5	4.62E-5
SK-MEL-2	1.172	2.290	2.266	2.250	2.312	2.062	0.041	98	96	102	80	-97	1.47E-5	2.83E-5	5.44E-5
SK-MEL-28	0.693	2.002	1.952	1.853	1.862	0.990	0.002	96	89	89	23	-100	3.89E-6	1.53E-5	3.92E-5
SK-MEL-5	1.062	2.954	2.794	2.569	2.677	1.169	0.018	92	80	85	6	-98	2.78E-6	1.13E-5	3.43E-5
UACC-257	1.151	2.565	2.539	2.479	2.518	0.348	0.031	98	94	97	-70	-97	1.91E-6	3.81E-6	7.61E-6
UACC-62	0.704	2.232	2.046	1.944	1.915	1.365	0.016	88	81	79	43	-98	6.49E-6	2.03E-5	4.58E-5
Ovarian Cancer															
IGROV1	0.591	1.992	2.063	1.909	1.852	1.180	0.066	105	94	90	42	-89	6.81E-6	2.09E-5	5.04E-5
OVCAR-3	0.858	2.212	2.195	2.110	1.947	1.659	0.017	99	92	80	59	-98	1.14E-5	2.38E-5	4.95E-5
OVCAR-4	0.794	1.976	1.926	1.828	1.794	1.497	0.019	96	88	85	59	-98	1.15E-5	2.39E-5	4.98E-5
OVCAR-5	0.643	1.367	1.309	1.312	1.285	0.929	0.006	92	92	89	40	-99	6.12E-6	1.93E-5	4.43E-5
OVCAR-8	0.495	2.142	2.127	2.098	2.080	1.650	0.033	99	97	96	70	-93	1.33E-5	2.68E-5	5.43E-5
NCI/ADR-RES	0.372	1.264	1.245	1.233	1.246	1.011	0.076	98	97	98	72	-80	1.39E-5	2.98E-5	6.38E-5
SK-OV-3	0.624	1.547	1.483	1.440	1.517	0.965	0.076	93	88	97	37	-88	6.03E-6	1.97E-5	4.97E-5
Renal Cancer															
786-0	0.440	2.192	2.221	2.093	2.008	1.169	0.044	102	94	89	42	-90	6.68E-6	2.07E-5	4.97E-5
A498	1.307	2.187	2.183	2.034	2.071	2.089	0.092	100	83	87	89	-93	1.64E-5	3.08E-5	5.80E-5
ACHN	0.426	1.620	1.618	1.500	1.496	0.983	-0.001	100	90	90	47	-100	8.34E-6	2.08E-5	4.56E-5
CAKI-1	0.462	1.361	1.302	1.601	1.524	0.658	0.013	93	127	118	22	-97	5.09E-6	1.52E-5	4.01E-5
RXF 393	1.053	1.637	1.665	1.613	1.615	0.980	0.052	105	96	96	-7	-95	2.80E-6	8.56E-6	3.08E-5
SN12C	0.552	2.035	1.885	1.805	1.756	1.003	0.010	90	84	81	30	-98	4.11E-6	1.72E-5	4.22E-5
TK-10	0.990	2.125	1.999	2.037	2.053	1.617	0.013	89	92	94	55	-99	1.08E-5	2.28E-5	4.83E-5
UO-31	0.704	1.996	1.764	1.784	1.755	0.924	0.007	82	84	81	17	-99	3.07E-6	1.40E-5	3.78E-5
Prostate Cancer															
PC-3	0.414	1.696	1.664	1.653	1.637	1.207	0.063	97	97	95	62	-85	1.20E-5	2.64E-5	5.79E-5
DU-145	0.339	1.227	1.229	1.189	1.240	1.001	0.025	100	96	101	75	-93	1.40E-5	2.79E-5	5.56E-5
Breast Cancer															
MCF7	0.420	1.943	1.885	1.675	1.742	0.854	0.092	96	82	87	29	-78	4.28E-6	1.85E-5	5.44E-5
MDA-MB-231/ATCC	0.549	1.120	1.116	1.056	1.035	0.839	0.014	99	89	85	51	-98	1.01E-5	2.20E-5	4.78E-5
HS 578T	1.661	2.743	2.623	2.498	2.598	1.988	0.915	89	77	87	30	-45	4.45E-6	2.52E-5	> 1.00E-4
BT-549	1.156	2.218	2.233	2.105	2.160	1.664	0.065	101	89	95	48	-94	8.97E-6	2.17E-5	4.87E-5
T-47D	0.631	1.276	1.199	1.127	1.133	0.948	0.167	88	77	78	49	-74	9.28E-6	2.51E-5	6.42E-5
MDA-MB-468	1.202	1.975	1.950	1.887	1.925	1.482	0.074	97	89	94	36	-94	5.75E-6	1.90E-5	4.60E-5

Figure 162. In-vitro five dose testing results for compound **11**

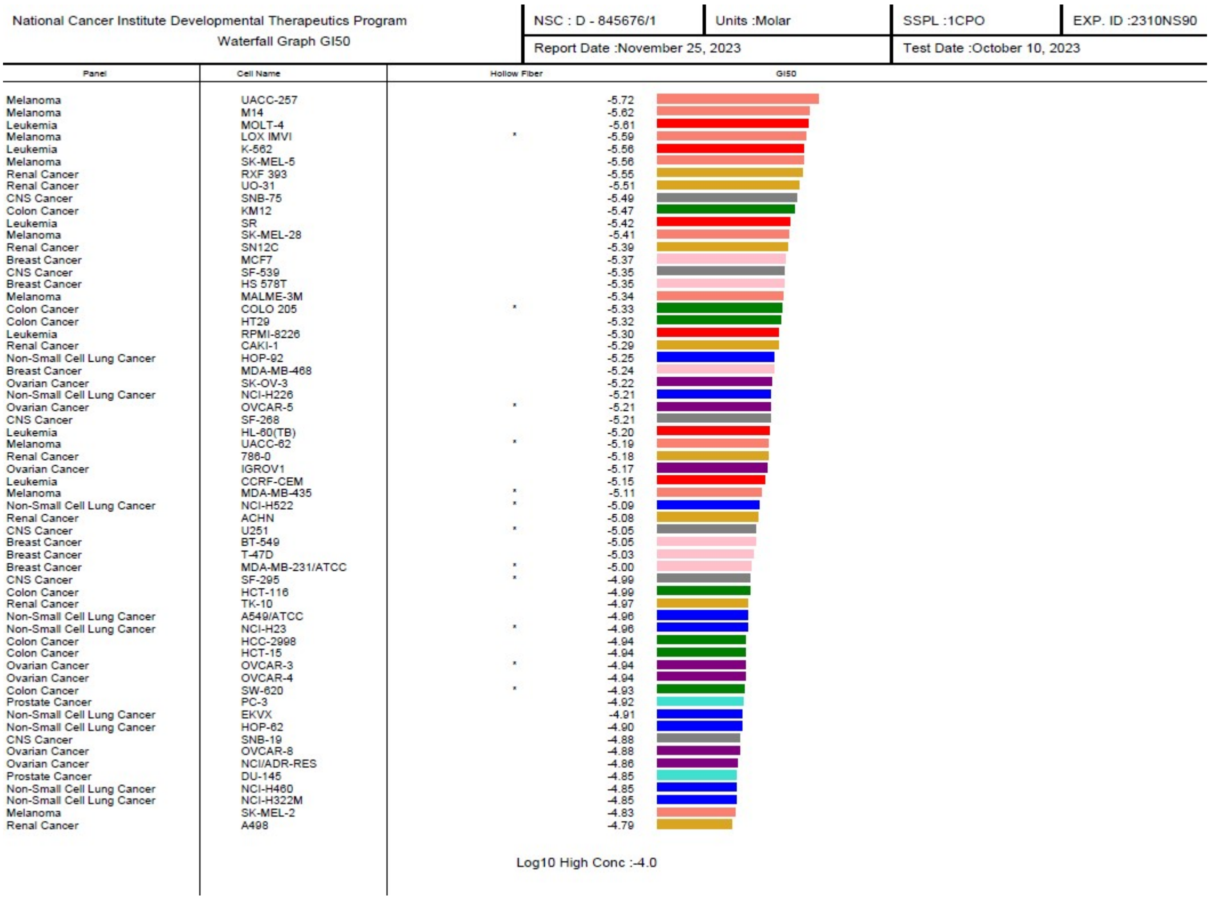

Figure 163. IC_{50} values depicted in the waterfall graph for compound **11**
